# Modifiable risk factors of major depressive disorder: A Mendelian randomization study

**DOI:** 10.1371/journal.pone.0289419

**Published:** 2023-08-03

**Authors:** Xiaofei Zheng, Li Tong, Chong Zhang, Chaoyang Zhang, Chao Zhang, Bangbei Wan

**Affiliations:** 1 Department of General Surgery, The First Affiliated Hospital of Anhui Medical University, Hefei, China; 2 Reproductive Medical Center, Hainan Women and Children’s Medical Center, Haikou, China; 3 Department of General Surgery, The Second Affiliated Hospital, School of Medicine, Zhejiang University, Hangzhou, China; Universita Cattolica del Sacro Cuore Sede di Roma, ITALY

## Abstract

Identifying modifiable risk factors early on is essential to prevent major depressive disorder (MDD). This study systematically investigated the causal relationship between 19 modifiable risk factors and MDD. Single-nucleotide polymorphisms (SNPs) associated with 19 potentially modifiable risk factors were screened via the genome-wide association study (GWAS) enrolling individuals of European descent. Summary-level data for MDD (59,851 cases and 113,154 controls) were extracted from the UK Biobank. The inverse-variance-weighted (IVW) method was utilized as the primary analysis. Sensitivity analyses were performed using the MR-Egger method, the Maximum likelihood method, the MR-pleiotropy residual sum outlier (MR-PRESSO) method, and MR-robust adjusted profile score (MR-RAPS) method. MR-Egger regression, heterogeneity tests, pleiotropy tests, and leave-one-out tests were also performed to analyze sensitivity. The MR Steiger test was used to verify the directionality of the exposure to the outcome. Genetically predicted smoking initiation increased the risk of MDD (*P* = 6.00E-09), while smoking status: never and past tobacco smoking decreased the risk of MDD (all *P* < 0.01). In addition, education level was inversely associated with MDD risk (all *P* < 0.01). Genetically instrumented sleeplessness/insomnia, daytime naps, and nap during the day were positively related to the risk of MDD (all *P* < 0.01). Personal feelings, including guilt, hurt, tension, and worry too long after an embarrassing experience, had a suggestive increased risk for MDD (all *P* < 0.000). The remaining five modifiable risk factors were all causally associated with the risk of MDD, including neuroticism, neuroticism scores, body mass index (BMI), average total household income before tax, and types of physical activity in the last 4 weeks (all *P* < 0.01). All 19 potentially modifiable risk factors were causally associated with the risk of MDD. The main hypothesis of this MR study was that identifying and intervening in these 19 potentially modifiable risk factors could be beneficial to the prevention and treatment of MDD and further reduce mortality and economic burden.

## Introduction

Major depressive disorder (MDD) is a worldwide disease characterized by persistent low mood. MDD has an estimated prevalence of 4.4% worldwide, characterized by high mortality and disability rates [1–3]. In addition, MDD also imposes a substantial economic burden on the United States [4]. Compared with healthy people, patients with MDD have a higher risk for suicide and more serious medical problems, thus causing a huge social burden. MDD is influenced by multiple environmental and genetic factors, such as sexual, regional brain volumes and physical or emotional abuse during childhood, which are difficult to change [5]. Necessarily, further exploration of risk factors, which are easily controlled and prevented, for MDD can help design and implement effective prevention strategies or novel treatments.

Several personal and sociodemographic factors are related to MDD in traditional research, such as physical activity [6, 7], smoking [8, 9], education level [10], sleeping [11, 12], and BMI [13]. Cannabis has been, over the past two decades, one of the most widely used illegal psychoactive substances, particularly among adolescents and young adults. The association between cannabis use, mental illness, and suicidal behavior has been frequently reported, although this link’s exact nature is still poorly investigated. The paper’s results showed that Cannabis use was a relevant risk factor associated with suicidal attempts and behaviors in psychotic and non-psychotic samples [14]. In addition to depression, substance use disorders, particularly cannabis use, are recognized as significant risk factors for suicidal behavior in clinical and community populations [14]. Moreover, some epidemiological researchers suggest that personal feelings, including neuroticism items, may exacerbate MDD [15–22]. Besides, immune-inflammatory changes are important in the pathophysiology of major depression and suicidal behavior [23]. It is well known that traditional observation research is subjected to residual confounding factors and cannot establish a causal relationship. Therefore, elucidating the causal role of these controllable risk factors in MDD is very important to guide clinicians in preventing and treating MDD.

As a novel method, Mendelian randomization (MR), which treats heritable variation as a natural experiment, has been used to assess the potential causal association between multiple diseases [24]. Genetic variants are randomly allocated before birth, never change after birth, and unaffected by environmental factors; thus, consider making up for the lack of observation of study-residual confounding and reverse causation. Some MR studies have demonstrated the association between risk factors and MDD, but their exposure or outcome was single and not detailed enough [25–27]. Here, we demonstrate whether there is a causal relationship between 19 modifiable risk factors and MDD through the two-sample MR method using the latest and more extensive data from IEU Open GWAS database.

## Materials and methods

### Assumption of Mendelian randomization study

**Fig 1:**
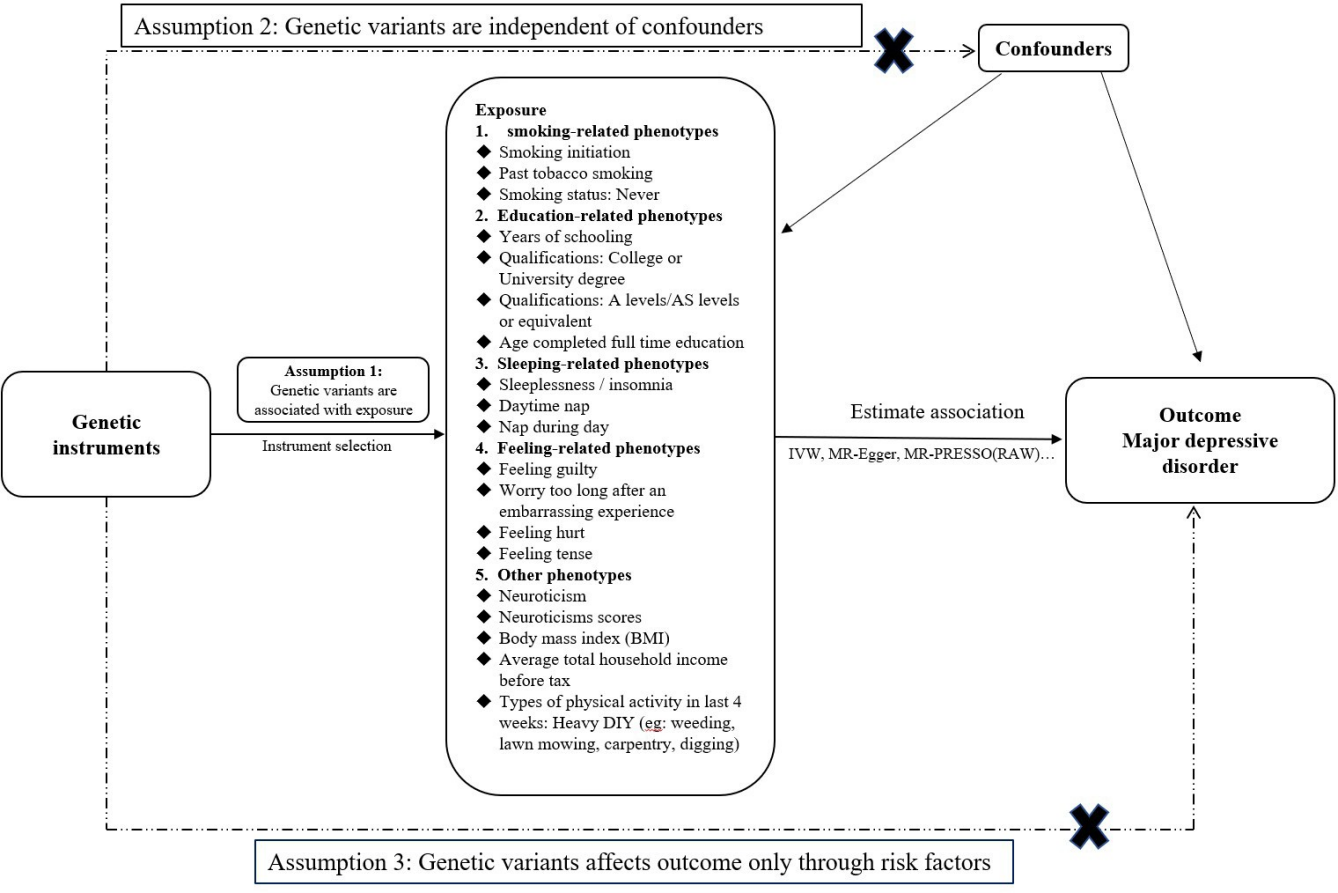
Mendelian randomization (MR) model. The MR research is based on the following principles:(i) Genetic instrument variables (GIVs) are significantly associated with exposures (19 potentially modifiable risk factors); (ii) GIVs are independent of any residual confounding factors; (iii) The affection of GIVs on the outcome only through risk factors without horizontal pleiotropy.

The MR research is based on the following principles:(i) Genetic instrument variables (GIVs) are significantly associated with exposures (19 potentially modifiable risk factors). The *P* value (*P* < 5 × 10−^8^) of the GIVs and the clumping method to exclude linkage disequilibrium (LD) were used to ensure the above assumption; (ii) GIVs are independent of any residual confounding factors; (iii) The affection of GIVs on the outcome (MDD) only through risk factors without horizontal pleiotropy [28, 29] (Fig 1). 19 controllable risk factors of our study were divided into five sections: smoking-related phenotypes, education-related phenotypes, sleeping-related phenotypes, personal feeling-related phenotypes, and other phenotypes.

### Exposure and outcome data

The data supporting this study’s findings are openly available in IEU Open GWAS database at https://gwas.mrcieu.ac.uk/.

Based on wide phenotypic screening, modifiable and significant risk factors were selected for inclusion in the study. 19 potential modifiable risk factors used in our study were classified into five categories: (i) smoking-related phenotypes, including smoking initiation, past tobacco smoking, and smoking status: never; (ii) education-related phenotypes, including years of schooling, qualifications: college or university degree, qualifications: A levels/AS levels or equivalent and age completed full-time education; (iii) sleeping-related phenotypes, including sleeplessness/insomnia, daytime nap, and nap during day; (iv) personal feeling-related phenotypes, including feeling guilty, worry too long after an embarrassing experience, feeling hurt and feeling tense; (v) other phenotypes, including neuroticism, neuroticisms scores, body mass index (BMI), average total housed income before tax and types of physical activity in last 4 weeks: heavy DIY (e.g., weeding, lawn mowing, carpentry, digging) (S1 Table).

The outcome data was MDD (59,851 cases vs. 113,154 controls, ieu-1188). Participants in the 19 potentially modifiable risk factors were not screened for MDD.

### Two-sample MR

#### SNP selection

Based on the GWAS summary-level data of 19 potentially modifiable risk factors, SNPs with a lower P value (P < 5 × 10–8), longer physical distance (≥ 10,000 kb), and a lower likelihood of LD (r2 < 0.001) were maintained. Data of all GIVs used in this work are shown in S2 Table. The F statistic estimating the instrument strength of each SNP in a two-sample MR study was obtained from the published article [30, 31].

#### Primary analyses

The multiplicative random effects inverse-variance-weighted (IVW) as the primary method was used to calculate the combined effects of all SNPs [32].

#### Sensitivity analyses

We also performed the MR-Egger method [33], the maximum likelihood method [34], the MR-pleiotropy residual sum outlier method(MR-PRESSO) method [35], and the MR-robust adjusted profile score (MR-RAPS) [36] to evaluate the reliability and stability of the results. The IVW and MR-Egger methods were performed to estimate the heterogeneity, where the *P* value greater than 0.05 indicated no heterogeneity. The MR-PRESSO, MR-Egger, and IVW methods were employed to identify and remove outliers. The intercept of the MR-Egger model was used to test the pleiotropy, where a deviation from 0 denotes the presence of directional pleiotropy. What’s more, the leave-one-out method was implemented to assess the influence of a single SNP on the total effect of IVW. An available website MR method, referenced in the published article, was used to calculate the statistical power of MR analysis, where a power greater than 80% was considered an excellent value [37]. When the *P* value of the MR Steiget test was lower than 0.05, the causal direction of exposure-causing the outcome was statistically significant [38].

All the MR analyses were implemented via the TwoSampleMR [39] and MRPRESSO packages [35] in R software (V 4.2.2).

## Results

### Baseline information

The essential features of the 19 potentially modifiable risk factors are summarized in S1 Table. 9–262 SNPs were associated with the 19 modifiable risk factors (all *P* < 5 × 10−^8^). In addition, there was no weak instrument bias in the two-sample MR study (all F statistics > 10) (Table 1).

**Table 1:** Results of the MR analysis of the 19 modifiable risk factors for MDD. MR, Mendelian randomization; SNPs, single nucleotide polymorphisms; OR, odds ratio; GWAS, genome-wide association studies; IVW, inverse-variance-weighted; MR-PRESSO, MR-pleiotropy residual sum outlier; MR-RAPS, MR-robust adjusted profile score.

Exposure	Method	SNPs	OR (95% CI)	*P*	*P*_het	*P*_intercept	*P_* Steiger	F_ Statistic	R^2^	Power
**Smoking-related phenotypes**										
Smoking initiation	MR-Egger	67	1.612 (0.901–2.886)	1.13E-01	2.21E-05	6.25E-01	5.69E-48	74.563	8.24E-03	1.000
	**IVW**	**67**	**1.397 (1.248–1.564)**	**6.00E-09**	2.76E-05					
	Maximum likelihood	67	1.404 (1.289–1.530)	7.17E-15						
	MR-PRESSO(RAW)	67	1.397 (1.232–1.585)	1.92E-07						
	MR-RAPS	67	1.418 (1.303–1.544)	6.66E-16						
Smoking status: never	MR-Egger	62	0.200 (0.041–0.978	5.14E-02	1.74E-04	4.98E-01	3.70E-91	60.636	1.04E-02	1.000
	**IVW**	**62**	**0.342 (0.238–0.491)**	**6.61E-09**	1.96E-04					
	Maximum likelihood	62	0.331 (0.249–0.439)	1.59E-14						
	MR-PRESSO(RAW)	62	0.342 (0.227–0.514)	2.52E-07						
	MR-RAPS	62	0.326 (0.246–0.431)	4.44E-15						
Past tobacco smoking	MR-Egger	81	0.768 (0.366–1.612)	4.87E-01	8.12E-06	8.71E-01	4.20E-102	50.777	9.67E-03	1.000
	**IVW**	**81**	**0.723 (0.627–0.834)**	**8.16E-06**	1.11E-05					
	Maximum likelihood	81	0.715 (0.641–0.797)	1.54E-09						
	MR-PRESSO(RAW)	81	0.726 (0.622–0.841)	2.64E-05						
	MR-RAPS	81	0.712 (0.639–0.793)	6.84E-10						
**Education-related phenotypes**										
Years of schooling	MR-Egger	251	0.632 (0.393–1.015)	5.86E-02	4.96E-13	6.92E-01	2.41E-182	23.712	7.76E-03	1.000
	**IVW**	**251**	**0.693 (0.620–0.776)**	**1.61E-10**	6.25E-13					
	Maximum likelihood	251	0.691 (0.634–0.753)	3.95E-17						
	MR-PRESSO(RAW)	251	0.693 (0.617–0.779)	7.87E-10						
	MR-RAPS	251	0.684 (0.628–0.745)	0.00E+00						
Qualifications: college or university degree	MR-Egger	151	0.709 (0.248–2.026)	5.22E-01	2.62E-09	6.08E-01	5.32E-288	24.790	1.12E-02	1.000
	**IVW**	**151**	**0.542 (0.431–0.682)**	**1.66E-07**	3.19E-09					
	Maximum likelihood	151	0.546 (0.459–0.650)	9.17E-12						
	MR-PRESSO(RAW)	151	0.542 (0.427–0.689)	5.49E-07						
	MR-RAPS	151	0.528 (0.445–0.628)	4.57E-13						
Qualifications: A levels/AS levels or equivalent	MR-Egger	50	0.251 (0.018–3.459)	3.07E-01	1.08E-04	7.05E-01	2.11E-75	72.693	1.09E-02	1.000
	**IVW**	**50**	**0.414 (0.265–0.648)**	**1.15E-04**	1.44E-04					
	Maximum likelihood	50	0.430 (0.308–0.601)	7.99E-07						
	MR-PRESSO(RAW)	50	0.414 (0.256–0.671)	3.36E-04						
	MR-RAPS	50	0.397 (0.285–0.553)	4.86E-08						
Age completed full time education	MR-Egger	34	0.837 (0.177–3.959)	8.24E-01	7.86E-04	7.18E-01	1.05E-49	99.354	1.09E-02	1.000
	**IVW**	**34**	**0.631 (0.468–0.850)**	**2.48E-03**	1.07E-03					
	Maximum likelihood	34	0.617 (0.494–0.770)	1.87E-05						
	MR-PRESSO(RAW)	34	0.631 (0.458–0.869)	4.78E-03						
	MR-RAPS	34	0.617 (0.495–0.769)	1.64E-05						
**Sleeping-related phenotypes**										
Sleeplessness/ insomnia	MR-Egger	24	0.887 (0.311–2.525)	8.24E-01	5.85E-02	1.25E-01	5.44E-28	149.165	1.06E-02	1.000
	**IVW**	**24**	**1.973 (1.366–2.849)**	**2.89E-04**	3.19E-02					
	Maximum likelihood	24	2.004 (1.488–2.697)	4.64E-06						
	MR-PRESSO(RAW)	24	1.973 (1.300–2.995)	1.42E-03						
	MR-RAPS	24	2.026 (1.503–2.731)	3.53E-06						
Daytime nap	MR-Egger	79	1.917 (0.779–4.721)	1.61E-01	8.97E-04	6.94E-01	3.90E-128	56.272	9.84E-03	1.000
	**IVW**	**79**	**1.612 (1.233–2.108)**	**4.78E-04**	1.10E-03					
	Maximum likelihood	79	1.639 (1.317–2.039)	9.43E-06						
	MR-PRESSO(RAW)	79	1.612 (1.220–2.131)	7.90E-04						
	MR-RAPS	79	1.637 (1.316–2.036)	9.61E-06						
Nap during day	MR-Egger	77	1.941 (0.727–5.183)	1.89E-01	6.20E-04	6.64E-01	4.44E-101	57.773	9.63E-03	1.000
	**IVW**	**77**	**1.574 (1.195–2.072)**	**1.23E-03**	7.56E-04					
	Maximum likelihood	77	1.590 (1.273–1.985)	4.34E-05						
	MR-PRESSO(RAW)	77	1.574 (1.183–2.093)	1.82E-03						
	MR-RAPS	77	1.599 (1.281–1.996)	3.29E-05						
**Feeling-related phenotypes**										
Feeling guilty	MR-Egger	9	1.521(0.003–14.767)	9.00E-01	3.68E-01	9.01E-01	2.76E-13	425.543	1.02E-02	1.000
	**IVW**	**9**	**2.294 (1.570–3.353)**	**1.80E-05**	4.70E-01					
	Maximum likelihood	9	2.346 (1.579–3.486)	2.41E-05						
	MR-PRESSO(RAW)	9	2.294 (1.345–3.914)	2.32E-03						
	MR-RAPS	9	2.334 (1.561–3.489)	3.64E-05						
Worry too long after an embarrassing experience	MR-Egger	15	0.868 (0.112–6.733)	8.94E-01	1.37E-01	3.65E-01	1.43E-17	248.288	1.01E-02	1.000
	**IVW**	**15**	**2.284 (1.642–3.176)**	**9.15E-07**	1.36E-01					
	Maximum likelihood	15	2.357 (1.762–3.153)	7.47E-09						
	MR-PRESSO(RAW)	15	2.284 (1.472–3.544)	2.30E-04						
	MR-RAPS	15	2.348 (1.753–3.145)	1.05E-08						
Feeling hurt	MR-Egger	24	2.428 (0.703–8.385)	1.75E-01	2.76E-03	9.23E-01	7.92E-23	156.628	1.01E-02	1.000
	**IVW**	**24**	**2.287 (1.683–3.108)**	**1.25E-07**	4.10E-03					
	Maximum likelihood	24	2.388 (1.893–3.012)	2.00E-13						
	MR-PRESSO(RAW)	24	2.287 (1.559–3.354)	2.30E-05						
	MR-RAPS	24	2.376 (1.888–2.991)	1.70E-13						
Feeling tense	MR-Egger	17	1.551(0.145–16.547)	7.21E-01	2.04E-01	8.00E-01	1.03E-19	213.370	9.76E-03	1.000
	**IVW**	**17**	**2.111 (1.563–2.851)**	**1.09E-06**	2.54E-01					
	Maximum likelihood	17	2.167 (1.631–2.878)	9.44E-08						
	MR-PRESSO(RAW)	17	2.111 (1.430–3.116)	1.69E-04						
	MR-RAPS	17	2.160 (1.619–2.880)	1.58E-07						
**Other phenotypes**										
Neuroticism	MR-Egger	65	0.957 (0.543–1.685)	8.79E-01	2.80E-05	1.72E-01	1.10E-71	55.624	1.09E-02	1.000
	**IVW**	**65**	**1.420 (1.306–1.544)**	**1.86E-16**	1.59E-05					
	Maximum likelihood	65	1.433 (1.345–1.527)	7.54E-29						
	MR-PRESSO(RAW)	65	1.420 (1.283–1.572)	1.26E-11						
	MR-RAPS	65	1.442 (1.354–1.537)	0.00E+00						
Neuroticisms scores	MR-Egger	56	1.397 (1.049–1.860)	2.62E-02	9.89E-05	6.31E-01	2.72E-70	57.591	1.18E-02	1.000
	**IVW**	**56**	**1.303 (1.236–1.375)**	**2.34E-22**	1.24E-04					
	Maximum likelihood	56	1.322 (1.268–1.379)	1.26E-38						
	MR-PRESSO(RAW)	56	1.303 (1.215–1.398)	1.50E-13						
	MR-RAPS	56	1.319 (1.265–1.376)	0.00E+00						
Body mass index	MR-Egger	262	1.058 (0.873–1.283)	5.67E-01	9.75E-11	1.14E-01	0.00E+00	15.919	1.24E-02	1.000
	**IVW**	**262**	**1.224 (1.143–1.311)**	**7.19E-09**	5.38E-11					
	Maximum likelihood	262	1.228 (1.164–1.296)	7.24E-14						
	MR-PRESSO(RAW)	262	1.224 (1.140–1.313)	2.05E-08						
	MR-RAPS	262	1.231 (1.167–1.299)	2.91E-14						
Average total household income before tax	MR-Egger	40	1.060 (0.490–2.292)	8.84E-01	4.12E-02	2.52E-01	1.83E-50	100.269	1.01E-02	0.997
	**IVW**	**40**	**0.679 (0.569–0.810)**	**1.71E-05**	3.57E-02					
	Maximum likelihood	40	0.684 (0.588–0.795)	7.86E-07						
	MR-PRESSO(RAW)	40	0.679 (0.557–0.826)	1.11E-04						
	MR-RAPS	40	0.669 (0.575–0.779)	1.98E-07						
Types of physical activity in last 4 weeks: Heavy DIY	MR-Egger	15	2.227(0.035–41.168)	7.11E-01	8.23E-01	4.27E-01	3.23E-1	265.182	8.62E-03	1.000
	**IVW**	**15**	**0.402 (0.200–0.810)**	**1.07E-02**	8.32E-01					
	Maximum likelihood	15	0.406 (0.199–0.826)	1.29E-02						
	MR-PRESSO(RAW)	15	0.402 (0.209–0.776)	6.61E-03						
	MR-RAPS	15	0.396 (0.191–0.821)	1.28E-02						

Otherwise, the statistical power for outcome MDD was approximately equal to 100% (Table 1). S2 Table summarizes the details of all SNPs (S2 Table).

No evidence of directional pleiotropy was found based on the intercept of MR-Egger. (Table 1). The results of the leave-one-out analysis showed that no single SNP influenced the overall estimates (S3 Table). The influence of the modifiable risk factors on MDD risk was the correct causal direction by the MR Steiger test (all *P* < 0.01) (Table 1).

### Smoking-related phenotypes for the risk of MDD

As for the smoking-related phenotypes in our MR study, smoking initiation tends to increase the risk of genetically developing MDD. Under the IVW method, the odds ratio (OR) (95% CI) was 1.386 (1.225–1.568; *P* = 6.00E-09) (Table 1). Other MR methods, including Maximum likelihood, MR-RAPS, and MR-PRESSO, showed the same sensitivity (Table 1, S1A and S2A Figs).

In addition, smoking status: never and past tobacco smoking were all inversely and genetically related to MDD, where the OR was 0.342 (0.238–0.491; *P* = 6.61E-09) and 0.723 (0.627–0.834; *P* = 8.16E-06) via the IVW method (Table 1). More importantly, the Maximum likelihood, MR-RAPS, and MR-PRESSO methods significantly showed the same sensitivity, indicating the results were reliable and stable (Table 1, S1B, S1C, S2B and S2C Figs).

### Education-related phenotypes for the risk of MDD

Regarding the education-related phenotypes, they all genetically and protectively decreased the risk of MDD. The OR was 0.693 (0.620–0.776; *P* = 1.61E-10), 0.542 (0.431–0.682; *P* = 1.66E-07), 0.414 (0.265–0.648; *P* = 1.15E-04) and 0.631 (0.468–0.850; *P* = 2.48E-03), respectively (Table 1). We found the same protective association via the Maximum likelihood, MR-PRESSO, and MR-RAPS methods, indicating the results were reliable and stable (Table 1, S1D–S1G, S2D–S2G Figs).

### Sleeping-related phenotypes for the risk of MDD

Genetic predisposition to sleeping-related phenotypes was significantly related to MDD under the IVW method (Table 1). Those were supported by the Maximum Likelihood, MR-PRESSO, and MR-RAPS methods, although the MR-Egger method did not reach statistical significance (Table 1, S1H–S1J, S2H–S2J Figs).

### Feeling-related phenotypes for the risk of MDD

To our primary MR method, the IVW method genetically showed that personal feeling-related phenotypes were all associated with the overall risk of advanced MDD (Table 1). Maximum Likelihood, MR-PREEO method, and MR-RAPS method for the association of feeling-related risk factors with outcome MDD were broadly similar to our primary MR method (Table 1, S1K–S1N, S2K–S2N Figs), indicating that our results were reliable and stable.

### Other phenotypes for the risk of MDD

As for the remaining phenotypes, there were suggestive associations between three risk factors (neuroticism, neuroticism scores, and BMI) and MDD. The results of the IVW method are shown in Table 1. Other MR methods used in our study showed the same sensitivity (Table 1, S1O–S1Q, S2O–S2Q Figs).

Regarding average total household income before tax and types of physical activity in the last 4 weeks: heavy DIY (e.g., weeding, lawn mowing, carpentry, digging), we found an inverse association between the two risk factors and MDD under the IVW method (OR = 0.679, *P* = 1.71E-05; OR = 0.402, *P* = 1.07E-02). The sensitivity of Maximum likelihood, MR-PREEO, and MR-RAPS methods for the association of the two factors with MDD were broadly similar to the IVW method (Table 1, S1R, S1S, S2R and S2S Figs), indicating that our results were reliable.

## Discussion

Genetically, the results suggested that smoking, low education level, poor sleep quality, and negative personal emotion were all positively and significantly related to the risk of MDD. Moreover, among the 19 personal and sociodemographic controllable risk factors, the remaining factors had significant casual relationships with MDD. The main hypothesis of this MR study was that identifying and intervening in these 19 potentially modifiable risk factors could be beneficial to the prevention and treatment of MDD and further reduce mortality and economic burden.MDD is a complex, pervasive and burdensome disorder with links to multiple factors [40], including smoking behaviour [41]. The results from some traditional researches have reported that smoking-related phenotypes are related to MDD [8, 9, 42], agreeing with the results of our two-sample MR study assessing the causal direction (*P* < 0.01). Conversely, a recent MR study genetically predicated smoking as a protective factor for MDD [43]. They just analyzed the pregnant women, while our outcome data consisted of men and women and was more representative. Some MR studies also explored the relationship between smoking and MDD, but their exposure or outcome was single and not detailed enough [25–27]. On the contrary, we studied multiple smoking phenotypes (smoking initiation, past tobacco smoking, and smoking status: never smoking) and MDD, one of the sub-categories of depression, and the data of our MR study is the latest release and more extensive than others. Genetically, our findings remind the public that reducing or not smoking may reduce the risk of MDD.

Epidemiological and observational investigation and review articles have repeatedly explored the relationship between sleep-related phenotypes and MDD [11, 12, 44–47]. Although some investigators accounted for confounding factors and repeated participants, residual confounding and reverse causality are still unavoidable in epidemiological and observational investigations [48]. Therefore, the MR analyses seem critical in addressing the above problems. Our MR study genetically predicted that sleeplessness/insomnia, nap during the day, and daytime nap aggravated MDD, which indicated that improved sleep quality was beneficial for preventing and treating MDD. Another two MR studies genetically reported that sleep-related phenotypes were not associated with MDD [49, 50]. It should be pointed out that the sample size of exposure and outcome of our MR study was more significant than the above MR study, so our results are more credible, making the public more aware of the importance of increasing sleep quality in reducing the risk of MDD.

Educational level is a crucial sociodemographic determinant of health and influences many diseases and disorders. In a Chinese study, Gan and colleagues discovered that education level was positively related to the risk of MDD, regardless of men or other races [51]. Their results did not support the straightforward claim made in publications from Europe and the US that higher levels of education were linked to reduced prevalence of MDD [52]. Reports from Finland showed no discernible differences across various educational groupings [53]. Another study linked illiteracy among older adults with higher incidence and more severe depression [54]. The above evidence for conflicting results of the effect of education on MDD came from observational studies and tended to suffer from residual confounding. We explored the genetic association between different education-related phenotypes and the risk of MDD. The results suggested that increasing personal and educational levels may decrease the risk of MDD, making the public aware of the importance of raising the education level and living to learn.

Guilty is one of the human emotions and is fascinating and complex. Feeling guilty has negative and positive health-related outcomes and incredibly contradictory empirical results in studies of depression [22, 55, 56]. Some research posited guilty as a positive, healthy construct [57, 58]. Conversely, some researchers reported that feeling guilty was significantly associated with major depression [22, 59–64]. However, the casual direction was not revealed and suffered from residual confounding. We were the first to evaluate the relationship between guilty and MDD using the mendelian randomization method. We genetically suggested that by using several MR methods, feeling guilty was related to a higher risk for MDD. The trauma experienced by people with severe mental diseases extends beyond physical and sexual abuse. The results in a group of female patients having urodynamics revealed that depression was linked to more severe shame. [21]. One interpretive phenomenological research concluded that MDD patients had increased relative frequencies of embarrassment. [65].

On the contrary, Louis et al. reported that depressive symptoms might amplify embarrassment in essential tremors [66]. Our study showed that worrying too long after an embarrassing experience was related to an increased risk for MDD. Fewer studies explored whether feeling tense and hurt concerning MDD, and the results were equivocal and influenced by other factors [15–20]. Our work showed that genetically predicated feeling hurt and tense was associated with an increased risk for MDD. All the above findings about personal emotions remind the public to pay attention to controlling their emotions in daily life.

A precursor to mental illness, neuroticism is a propensity for unpleasant feelings such as melancholy, worry, and emotional instability. Many studies showed that high neuroticism was a strong vulnerability marker for developing MDD and was common among those with MDD [67–70]. In an MR study, they found neuroticism and its items were genetically associated with the risk for MDD, but they did not show the statistical power and the causal direction. As for our MR study, we found neuroticism, its items (feeling guilty, worrying too long after an embarrassing experience, feeling hurt), and neuroticism scores were connected to a higher risk of MDD. Additionally, the MR Steiger test confirmed the casual assumption that neuroticism or its items correlate with MDD, and the outcome supported the idea. (*P* < 0.01)). The power of our investigation was around 1. Moreover, the exposure data were from different sources, illustrating the association between Neuroticism and MDD, and the result was more convincing. The significant conclusion of our results is that lowering neuroticism would be advantageous in reducing the risk of MDD.

The growing body of research showing a link between physical activity and a lower risk of depression offers a potentially versatile option for prevention.[6, 7, 71]. Uncertainty exists over the association’s direction and causation, though. According to an MR investigation, Accelerometer-based exercise appeared to protect against MDD. Self-reported activity and MDD did not significantly relate, indicating that different physical activities may impact MDD [72]. Another MR study genetically suggested that physical activity is prospectively associated with depression [73]. Regarding our MR study, different physical activity from the above exposure was genetically associated with the reduced risk for MDD, which further demonstrated that physical activity is essential in lowering MDD and reminded people to increase DIY activity to prevent MDD. Regarding BMI, our MR findings revealed an inverse relationship between BMI and MDD, indicating that the general public should maintain a healthy BMI to lower their chance of developing MDD.

The primary significance of our research is that we first conclude 19 modifiable risk factors and divide them into five categories, which are genetically associated with the risk for MDD, with stringent quality control conditions and analysis methodologies and produce dependable and steady research results. Unlike other MR studies, the risk factors of our MR study are more detailed and multifaceted, making the results more reliable. In addition, the sample size of our research is larger and latest than other studies. The results benefit the prevention and treatment of MDD by paying attention to the 19 risk factors.

We must admit that our study has some flaws. The results of our study may not apply to people of different origins because we only included participants of European ancestry. Thus, it is important to proceed with caution when interpreting the implications of our findings. Second, it would be intriguing to determine SNPs linked to the severity of MDD and analyses the relationship between modifiable risk variables and MDD severity. Since there are no publications on the associations between certain SNPs and the severity of MDD, we cannot do this association analysis. Thirdly, there is variability in the instrumental variables, possibly due to real causality rather than a failure to uphold the instrument variable premise. As for heterogeneity, we used the IVW as the primary analysis method. Fourth, other factors, such as other diseases, may confound our MR results, but avoiding them isn’t easy.

## Conclusions

There is a causal relationship between 19 modifiable risk factors and MDD through the two-sample MR method using the latest and more extensive data from IEU Open GWAS database, and identifying and intervening in these 19 potentially modifiable risk factors could be beneficial to the prevention and treatment of MDD and further reduce mortality and economic burden.

## Supporting information

S1 Fig Density plots of the MR study. Density plot of the MR results of 19 modifiable risk factors for MDD. Represent the results of heterogeneity analysis from 19 modifiable risk factors. MR, Mendelian randomization; MDD, major depressive disorder; SNP, single nucleotide polymorphisms; IVW, inverse-variance-weighted; MR-PRESSO, MR-pleiotropy residual sum outlier; MR-RAPS, MR-robust adjusted profile score.
(TIF)

S2 Fig Scatter plots of the MR study. On a log-odds scale, a scatter plot shows how each risk factor-related SNP affects MDD. MR, Mendelian randomization; MDD, major depressive disorder; SNP, single nucleotide polymorphisms; IVW, inverse-variance-weighted; MR-PRESSO, MR-pleiotropy residual sum outlier; MR-RAPS, MR-robust adjusted profile score.
(TIF)

S1 File Raw data. (XLSX)

S1 Table Data characteristics from the GWAS summary. SNP = single nucleotide polymorphism; GWAS, genome-wide association studies.
(DOCX)

S2 Table SNPs strongly associated with 19 risk factors and their F statistic. SNPs, single nucleotide polymorphisms; EAF, Effect allele frequency.
(DOCX)

S3 Table Leave-one-out results of the MR study. SNPs, single nucleotide polymorphisms.
(DOCX)
