# Supplementary figures and images for: Modifiable risk factors of major depressive disorder: A Mendelian randomization study
Source: PLoS One. 2023 Aug 3;18(8):e0289419. doi: 10.1371/journal.pone.0289419 (PMC10399902; doi:10.1371/journal.pone.0289419)

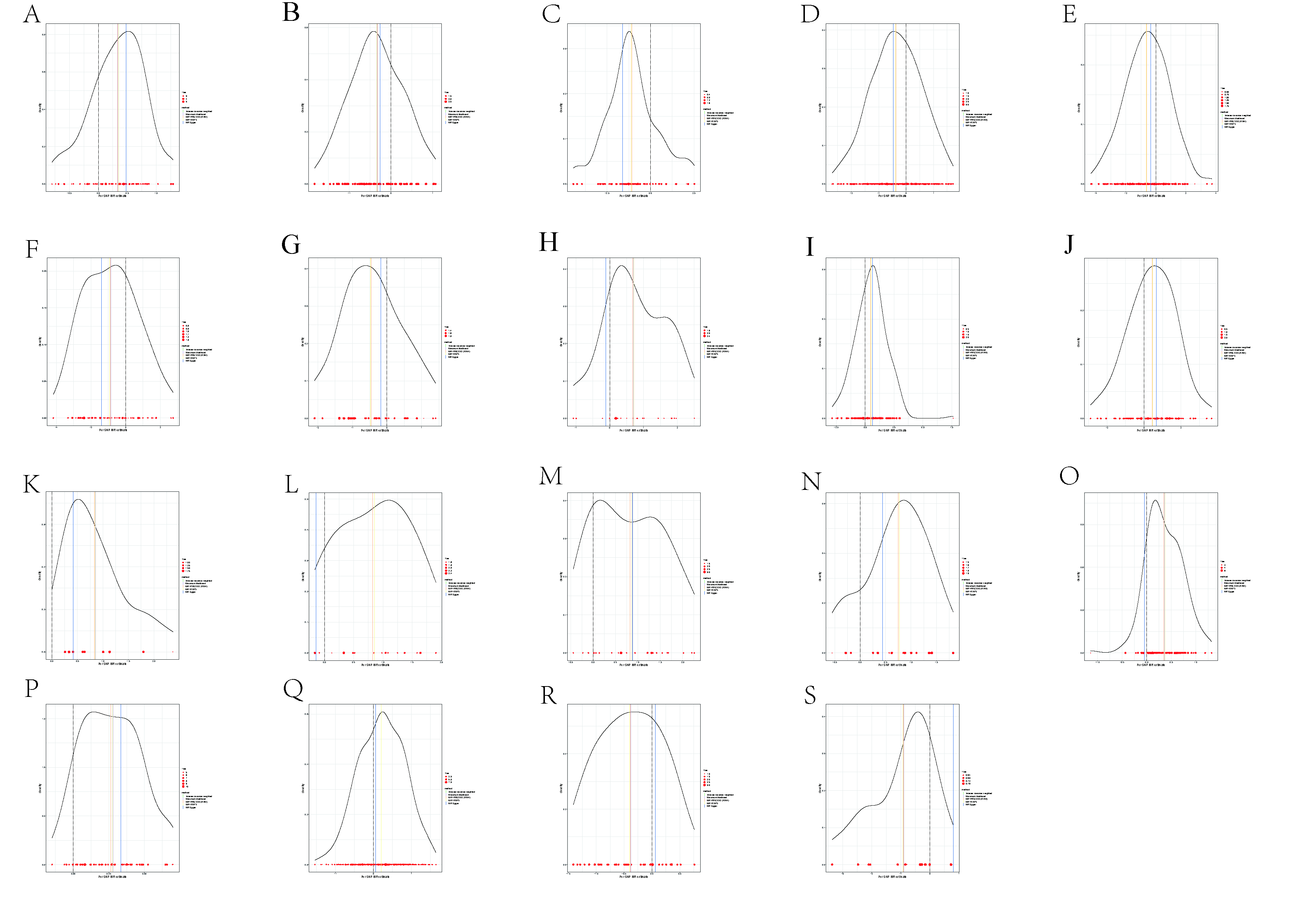

Supplement: S1 Fig — Density plot of the MR results of 19 modifiable risk factors for MDD. Represent the results of heterogeneity analysis from 19 modifiable risk factors. MR, Mendelian randomization; MDD, major depressive disorder; SNP, single nucleotide polymorphisms; IVW, inverse-variance-weighted; MR-PRESSO, MR-pleiotropy residual sum outlier; MR-RAPS, MR-robust adjusted profile score. (TIF) [file pone.0289419.s001.tif]

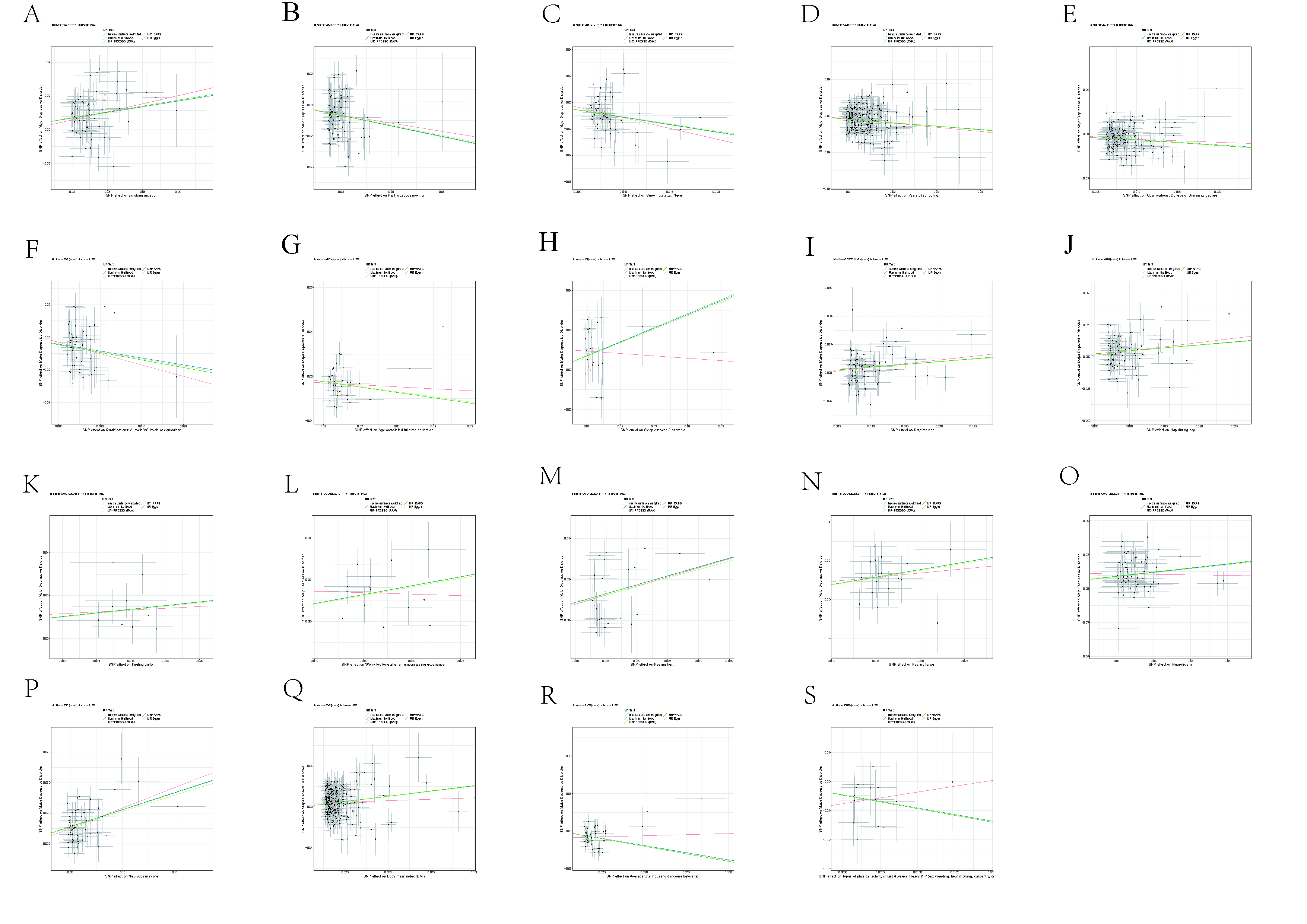

Supplement: S2 Fig — On a log-odds scale, a scatter plot shows how each risk factor-related SNP affects MDD. MR, Mendelian randomization; MDD, major depressive disorder; SNP, single nucleotide polymorphisms; IVW, inverse-variance-weighted; MR-PRESSO, MR-pleiotropy residual sum outlier; MR-RAPS, MR-robust adjusted profile score. (TIF) [file pone.0289419.s002.tif]
