# Supplementary material for: Modifiable risk factors of major depressive disorder: A Mendelian randomization study
Source: PLoS One. 2023 Aug 3;18(8):e0289419. doi: 10.1371/journal.pone.0289419 (PMC10399902; doi:10.1371/journal.pone.0289419)
Supplement: S1 Table — SNP = single nucleotide polymorphism; GWAS, genome-wide association studies. (DOCX) [file pone.0289419.s004.docx]

| **Table S1. characteristics of the GWAS summary data.** | | | | | |
| --- | --- | --- | --- | --- | --- |
| **Exposure** | **Ethnicity** | **GWAS ID** | **Total populations** | **PMID** | **SNPs** |
| **Smoking-related phenotypes** |  |  |  |  |  |
| Smoking initiation | European | ieu-b-4877 | 607,291 | 30643251 | 11,802,365 |
| Past tobacco smoking | European | ukb-b-2134 | 424,960 | NA | 9,851,867 |
| Smoking status: never | European | ukb-d-20116_0 | 359,706 | NA | 13,586,591 |
| **Education-related phenotypes** |  |  |  |  |  |
| Years of schooling | European | ieu-a-1239 | 766,345 | 30038396 | 10,101,242 |
| Qualifications: college or university degree | European | ukb-a-397 | 334,070 | NA | 10,894,596 |
| Qualifications: A levels/AS levels or equivalent | European | ukb-a-399 | 334,070 | NA | 10,894,596 |
| Age completed full time education | European | ukb-b-6134 | 307,897 | NA | 9,851,867 |
| **Sleeping-related phenotypes** |  |  |  |  |  |
| Sleeplessness / insomnia | European | ukb-a-13 | 336,965 | NA | 10,894,596 |
| Daytime nap | European | ebi-a-GCST011494 | 452,633 | 33568662 | 13,024,091 |
| Nap during day | European | ukb-b-4616 | 462,400 | NA | 9,851,867 |
| **Feeling-related phenotypes** |  |  |  |  |  |
| Feeling guilty | European | ebi-a-GCST006945 | 373,380 | 29500382 | 10,824,730 |
| Worry too long after an embarrassing experience | European | ebi-a-GCST006946 | 367,725 | 29500382 | 10,824,870 |
| Feeling hurt | European | ebi-a-GCST006951 | 372,047 | 29500382 | 10,824,675 |
| Feeling tense | European | ebi-a-GCST006952 | 371,318 | 29500382 | 10,824,652 |
| **Other phenotypes** |  |  |  |  |  |
| Neuroticism | European | ebi-a-GCST005232 | 329,821 | 29255261 | 18,436,568 |
| Neuroticisms scores | European | ukb-a-230 | 274,108 | NA | 10,894,596 |
| Body mass index (BMI) | European | ukb-a-248 | 336,107 | NA | 10,894,596 |
| Average total household income before tax | European | ukb-b-7408 | 397,751 | NA | 9,851,867 |
| Types of physical activity in last 4 weeks: heavy DIY | European | ukb-b-13184 | 397,751 | NA | 9,851,867 |
