# Supplementary material for: Modifiable risk factors of major depressive disorder: A Mendelian randomization study
Source: PLoS One. 2023 Aug 3;18(8):e0289419. doi: 10.1371/journal.pone.0289419 (PMC10399902; doi:10.1371/journal.pone.0289419)
Supplement: S2 Table — SNPs, single nucleotide polymorphisms; EAF, Effect allele frequency. (DOCX) [file pone.0289419.s005.docx]

**Table S2. SNPs strongly associated with risk factors and its F statistic.**

| Exposure | SNP | Effect allele | Other allele | EAF | Beta | Beta.se |
| --- | --- | --- | --- | --- | --- | --- |
| smoking initiation \|\| id:ieu-b-4877 | rs10001365 | A | G | 0.405 | -0.0249918 | 0.00364155 |
| smoking initiation \|\| id:ieu-b-4877 | rs10114490 | A | G | 0.198 | -0.0255148 | 0.00453171 |
| smoking initiation \|\| id:ieu-b-4877 | rs10233018 | G | A | 0.503 | 0.027069 | 0.00355741 |
| smoking initiation \|\| id:ieu-b-4877 | rs10260968 | A | G | 0.597 | -0.0203218 | 0.00360938 |
| smoking initiation \|\| id:ieu-b-4877 | rs10279261 | A | G | 0.619 | -0.0214194 | 0.00366263 |
| smoking initiation \|\| id:ieu-b-4877 | rs10498846 | T | C | 0.473 | 0.0206103 | 0.00355561 |
| smoking initiation \|\| id:ieu-b-4877 | rs1050847 | T | C | 0.505 | -0.0216231 | 0.00358893 |
| smoking initiation \|\| id:ieu-b-4877 | rs10905461 | C | T | 0.718 | -0.0239554 | 0.00414505 |
| smoking initiation \|\| id:ieu-b-4877 | rs11057005 | G | A | 0.43 | -0.0209298 | 0.00357892 |
| smoking initiation \|\| id:ieu-b-4877 | rs11078713 | G | A | 0.454 | -0.0201721 | 0.00360561 |
| smoking initiation \|\| id:ieu-b-4877 | rs1154693 | G | A | 0.856 | 0.0326217 | 0.00491232 |
| smoking initiation \|\| id:ieu-b-4877 | rs11658881 | G | A | 0.418 | 0.0201357 | 0.00361066 |
| smoking initiation \|\| id:ieu-b-4877 | rs11712680 | C | A | 0.174 | -0.0270476 | 0.00457843 |
| smoking initiation \|\| id:ieu-b-4877 | rs117143374 | C | T | 0.12 | 0.0292897 | 0.00526909 |
| smoking initiation \|\| id:ieu-b-4877 | rs11872397 | A | G | 0.252 | -0.0247725 | 0.00409477 |
| smoking initiation \|\| id:ieu-b-4877 | rs12042107 | C | T | 0.527 | -0.0222834 | 0.0035682 |
| smoking initiation \|\| id:ieu-b-4877 | rs12186738 | T | G | 0.154 | -0.0332644 | 0.00502051 |
| smoking initiation \|\| id:ieu-b-4877 | rs12333760 | C | T | 0.204 | -0.0290467 | 0.00480127 |
| smoking initiation \|\| id:ieu-b-4877 | rs12441907 | A | C | 0.186 | -0.0292051 | 0.00452262 |
| smoking initiation \|\| id:ieu-b-4877 | rs12474587 | T | G | 0.404 | 0.0276329 | 0.00358234 |
| smoking initiation \|\| id:ieu-b-4877 | rs12545053 | G | A | 0.397 | 0.0202808 | 0.00363668 |
| smoking initiation \|\| id:ieu-b-4877 | rs12632110 | G | A | 0.647 | -0.0233768 | 0.00375292 |
| smoking initiation \|\| id:ieu-b-4877 | rs13030994 | A | G | 0.485 | 0.0360925 | 0.0035563 |
| smoking initiation \|\| id:ieu-b-4877 | rs13261666 | T | G | 0.522 | -0.0268946 | 0.00355604 |
| smoking initiation \|\| id:ieu-b-4877 | rs134529 | C | T | 0.349 | -0.019984 | 0.00366078 |
| smoking initiation \|\| id:ieu-b-4877 | rs1385108 | T | C | 0.239 | 0.0246617 | 0.00415673 |
| smoking initiation \|\| id:ieu-b-4877 | rs1435741 | A | G | 0.425 | 0.0294151 | 0.00359095 |
| smoking initiation \|\| id:ieu-b-4877 | rs1445649 | C | T | 0.525 | 0.0239932 | 0.00356484 |
| smoking initiation \|\| id:ieu-b-4877 | rs1869243 | C | T | 0.481 | 0.0197411 | 0.00356289 |
| smoking initiation \|\| id:ieu-b-4877 | rs1899896 | T | C | 0.286 | 0.0264481 | 0.00388691 |
| smoking initiation \|\| id:ieu-b-4877 | rs1971318 | T | C | 0.141 | 0.0285074 | 0.00492533 |
| smoking initiation \|\| id:ieu-b-4877 | rs2046850 | T | C | 0.187 | -0.0248139 | 0.00447842 |
| smoking initiation \|\| id:ieu-b-4877 | rs2140114 | T | C | 0.518 | -0.0232591 | 0.00373402 |
| smoking initiation \|\| id:ieu-b-4877 | rs2378662 | A | G | 0.556 | 0.0209482 | 0.00356645 |
| smoking initiation \|\| id:ieu-b-4877 | rs240963 | C | T | 0.836 | -0.0410444 | 0.00483712 |
| smoking initiation \|\| id:ieu-b-4877 | rs2631024 | G | A | 0.737 | -0.0229645 | 0.00402823 |
| smoking initiation \|\| id:ieu-b-4877 | rs266047 | A | G | 0.529 | -0.0305098 | 0.00373855 |
| smoking initiation \|\| id:ieu-b-4877 | rs3001723 | A | G | 0.321 | 0.0335118 | 0.0038983 |
| smoking initiation \|\| id:ieu-b-4877 | rs35702515 | T | G | 0.162 | 0.0252442 | 0.00423093 |
| smoking initiation \|\| id:ieu-b-4877 | rs3800227 | G | A | 0.701 | 0.0228121 | 0.00405809 |
| smoking initiation \|\| id:ieu-b-4877 | rs3801289 | C | A | 0.351 | -0.0220618 | 0.00373982 |
| smoking initiation \|\| id:ieu-b-4877 | rs3904512 | A | G | 0.429 | -0.0211589 | 0.0035765 |
| smoking initiation \|\| id:ieu-b-4877 | rs4044321 | G | A | 0.642 | -0.0278417 | 0.00371058 |
| smoking initiation \|\| id:ieu-b-4877 | rs4236259 | G | T | 0.499 | -0.0247689 | 0.00355661 |
| smoking initiation \|\| id:ieu-b-4877 | rs4352629 | T | C | 0.492 | -0.0275284 | 0.00356881 |
| smoking initiation \|\| id:ieu-b-4877 | rs4523689 | G | A | 0.408 | -0.0206091 | 0.00364321 |
| smoking initiation \|\| id:ieu-b-4877 | rs4543592 | C | T | 0.468 | 0.0219314 | 0.00356244 |
| smoking initiation \|\| id:ieu-b-4877 | rs4674993 | G | A | 0.207 | -0.0252122 | 0.00443619 |
| smoking initiation \|\| id:ieu-b-4877 | rs4781977 | C | T | 0.205 | -0.0238668 | 0.00436473 |
| smoking initiation \|\| id:ieu-b-4877 | rs6265 | T | C | 0.203 | -0.0317863 | 0.00457843 |
| smoking initiation \|\| id:ieu-b-4877 | rs6433897 | C | T | 0.754 | 0.0224483 | 0.00405809 |
| smoking initiation \|\| id:ieu-b-4877 | rs66680800 | T | G | 0.397 | -0.0202716 | 0.00365268 |
| smoking initiation \|\| id:ieu-b-4877 | rs6669839 | T | C | 0.204 | 0.0260041 | 0.0043955 |
| smoking initiation \|\| id:ieu-b-4877 | rs6728726 | C | T | 0.829 | 0.0354486 | 0.00473279 |
| smoking initiation \|\| id:ieu-b-4877 | rs6893752 | G | A | 0.766 | -0.0240995 | 0.00407356 |
| smoking initiation \|\| id:ieu-b-4877 | rs7197072 | T | C | 0.238 | -0.0247672 | 0.0041686 |
| smoking initiation \|\| id:ieu-b-4877 | rs7224742 | T | C | 0.595 | -0.0207099 | 0.00365532 |
| smoking initiation \|\| id:ieu-b-4877 | rs72789632 | T | C | 0.12 | -0.0328856 | 0.00528628 |
| smoking initiation \|\| id:ieu-b-4877 | rs7322872 | T | C | 0.782 | -0.0255713 | 0.00433474 |
| smoking initiation \|\| id:ieu-b-4877 | rs76214862 | C | A | 0.202 | -0.0249903 | 0.00454745 |
| smoking initiation \|\| id:ieu-b-4877 | rs76608582 | A | C | 0.0389 | -0.0495575 | 0.00825958 |
| smoking initiation \|\| id:ieu-b-4877 | rs7929518 | G | A | 0.765 | 0.0242377 | 0.00428466 |
| smoking initiation \|\| id:ieu-b-4877 | rs7938812 | G | T | 0.424 | 0.0437914 | 0.00363668 |
| smoking initiation \|\| id:ieu-b-4877 | rs7969559 | G | A | 0.688 | -0.0243756 | 0.00395946 |
| smoking initiation \|\| id:ieu-b-4877 | rs9401770 | A | G | 0.273 | 0.0277307 | 0.003986 |
| smoking initiation \|\| id:ieu-b-4877 | rs962625 | G | A | 0.24 | 0.0237181 | 0.00403803 |
| smoking initiation \|\| id:ieu-b-4877 | rs993700 | C | T | 0.766 | -0.025928 | 0.00429163 |
| Past tobacco smoking \|\| id:ukb-b-2134 | rs1017998 | G | A | 0.620358 | -0.0151907 | 0.00277488 |
| Past tobacco smoking \|\| id:ukb-b-2134 | rs10474278 | G | A | 0.746763 | -0.0184912 | 0.00311705 |
| Past tobacco smoking \|\| id:ukb-b-2134 | rs10914684 | A | G | 0.325484 | 0.0165925 | 0.00287399 |
| Past tobacco smoking \|\| id:ukb-b-2134 | rs10956808 | G | T | 0.422089 | 0.0189115 | 0.00273732 |
| Past tobacco smoking \|\| id:ukb-b-2134 | rs10959442 | G | T | 0.46641 | -0.0171998 | 0.00269923 |
| Past tobacco smoking \|\| id:ukb-b-2134 | rs1109480 | A | G | 0.389103 | 0.0163393 | 0.0027867 |
| Past tobacco smoking \|\| id:ukb-b-2134 | rs11165623 | A | G | 0.504148 | -0.0158064 | 0.00269383 |
| Past tobacco smoking \|\| id:ukb-b-2134 | rs11613961 | C | T | 0.086782 | -0.0293599 | 0.00479699 |
| Past tobacco smoking \|\| id:ukb-b-2134 | rs1174864 | A | G | 0.54986 | -0.0153603 | 0.0027134 |
| Past tobacco smoking \|\| id:ukb-b-2134 | rs12209519 | G | A | 0.407575 | -0.0158856 | 0.0027537 |
| Past tobacco smoking \|\| id:ukb-b-2134 | rs12333760 | C | T | 0.165515 | 0.0213967 | 0.00363284 |
| Past tobacco smoking \|\| id:ukb-b-2134 | rs12450028 | T | C | 0.345053 | -0.0166053 | 0.0028331 |
| Past tobacco smoking \|\| id:ukb-b-2134 | rs1246265 | C | T | 0.695165 | -0.020922 | 0.00293474 |
| Past tobacco smoking \|\| id:ukb-b-2134 | rs12487411 | A | G | 0.470776 | 0.0167975 | 0.00270032 |
| Past tobacco smoking \|\| id:ukb-b-2134 | rs12608052 | T | C | 0.518925 | 0.0161105 | 0.0027009 |
| Past tobacco smoking \|\| id:ukb-b-2134 | rs12895462 | C | T | 0.191374 | 0.0200066 | 0.00344429 |
| Past tobacco smoking \|\| id:ukb-b-2134 | rs13009008 | G | A | 0.672659 | 0.0157552 | 0.00286663 |
| Past tobacco smoking \|\| id:ukb-b-2134 | rs139896 | C | T | 0.64784 | -0.0162135 | 0.00282014 |
| Past tobacco smoking \|\| id:ukb-b-2134 | rs147052174 | T | G | 0.018556 | -0.0603916 | 0.00997369 |
| Past tobacco smoking \|\| id:ukb-b-2134 | rs1499300 | C | A | 0.157852 | 0.0217944 | 0.00369422 |
| Past tobacco smoking \|\| id:ukb-b-2134 | rs1499976 | C | T | 0.852678 | -0.0314256 | 0.00381927 |
| Past tobacco smoking \|\| id:ukb-b-2134 | rs1559278 | C | T | 0.361217 | 0.0154864 | 0.0028052 |
| Past tobacco smoking \|\| id:ukb-b-2134 | rs1623003 | T | C | 0.664037 | -0.0184743 | 0.00286622 |
| Past tobacco smoking \|\| id:ukb-b-2134 | rs17503369 | C | T | 0.182208 | 0.0216357 | 0.00350324 |
| Past tobacco smoking \|\| id:ukb-b-2134 | rs1899689 | T | C | 0.389116 | -0.0154222 | 0.0027618 |
| Past tobacco smoking \|\| id:ukb-b-2134 | rs2289791 | T | G | 0.247319 | 0.0190853 | 0.00314571 |
| Past tobacco smoking \|\| id:ukb-b-2134 | rs2367724 | T | C | 0.673602 | 0.0160073 | 0.00287009 |
| Past tobacco smoking \|\| id:ukb-b-2134 | rs2433055 | G | T | 0.447651 | 0.0148366 | 0.00271458 |
| Past tobacco smoking \|\| id:ukb-b-2134 | rs2587507 | C | T | 0.505702 | 0.0155861 | 0.00267803 |
| Past tobacco smoking \|\| id:ukb-b-2134 | rs2797793 | C | T | 0.603851 | 0.0152706 | 0.00275684 |
| Past tobacco smoking \|\| id:ukb-b-2134 | rs2862465 | A | G | 0.409159 | 0.0158416 | 0.00273873 |
| Past tobacco smoking \|\| id:ukb-b-2134 | rs28647734 | A | G | 0.210012 | -0.0191736 | 0.00332696 |
| Past tobacco smoking \|\| id:ukb-b-2134 | rs2866724 | G | A | 0.266293 | -0.0188049 | 0.00305278 |
| Past tobacco smoking \|\| id:ukb-b-2134 | rs2917670 | C | T | 0.610217 | 0.0167548 | 0.00276245 |
| Past tobacco smoking \|\| id:ukb-b-2134 | rs2952251 | G | A | 0.739866 | -0.0175299 | 0.00307867 |
| Past tobacco smoking \|\| id:ukb-b-2134 | rs35445224 | C | T | 0.181004 | -0.0206228 | 0.00357585 |
| Past tobacco smoking \|\| id:ukb-b-2134 | rs35761479 | A | G | 0.120751 | 0.0229646 | 0.00413628 |
| Past tobacco smoking \|\| id:ukb-b-2134 | rs3808937 | T | C | 0.207824 | -0.020549 | 0.00332265 |
| Past tobacco smoking \|\| id:ukb-b-2134 | rs3811038 | C | T | 0.275964 | -0.0170544 | 0.00302893 |
| Past tobacco smoking \|\| id:ukb-b-2134 | rs3827592 | A | G | 0.350953 | 0.0177215 | 0.00282961 |
| Past tobacco smoking \|\| id:ukb-b-2134 | rs3857914 | C | T | 0.302135 | -0.0201533 | 0.00295853 |
| Past tobacco smoking \|\| id:ukb-b-2134 | rs3935790 | A | G | 0.416104 | -0.0154673 | 0.00273757 |
| Past tobacco smoking \|\| id:ukb-b-2134 | rs4708899 | G | A | 0.575572 | 0.0158982 | 0.00273541 |
| Past tobacco smoking \|\| id:ukb-b-2134 | rs528301 | A | G | 0.554258 | -0.0203355 | 0.00270534 |
| Past tobacco smoking \|\| id:ukb-b-2134 | rs540356 | A | C | 0.413629 | -0.0184455 | 0.00276048 |
| Past tobacco smoking \|\| id:ukb-b-2134 | rs56081685 | G | T | 0.31367 | 0.016504 | 0.00291156 |
| Past tobacco smoking \|\| id:ukb-b-2134 | rs56760958 | T | C | 0.390264 | 0.0172678 | 0.00275838 |
| Past tobacco smoking \|\| id:ukb-b-2134 | rs58400863 | A | G | 0.341566 | 0.0176273 | 0.00285288 |
| Past tobacco smoking \|\| id:ukb-b-2134 | rs597808 | G | A | 0.515766 | 0.0221177 | 0.00270078 |
| Past tobacco smoking \|\| id:ukb-b-2134 | rs6141314 | A | G | 0.241567 | -0.0191608 | 0.00316833 |
| Past tobacco smoking \|\| id:ukb-b-2134 | rs62022627 | G | A | 0.400649 | 0.0189626 | 0.00275549 |
| Past tobacco smoking \|\| id:ukb-b-2134 | rs6265 | T | C | 0.189039 | 0.0261462 | 0.00344035 |
| Past tobacco smoking \|\| id:ukb-b-2134 | rs6464024 | T | C | 0.427444 | 0.0175549 | 0.00272318 |
| Past tobacco smoking \|\| id:ukb-b-2134 | rs6588376 | A | G | 0.208312 | -0.0188335 | 0.00331846 |
| Past tobacco smoking \|\| id:ukb-b-2134 | rs67174662 | G | A | 0.375322 | 0.0166562 | 0.00278624 |
| Past tobacco smoking \|\| id:ukb-b-2134 | rs6751705 | G | T | 0.512137 | -0.0224698 | 0.00269257 |
| Past tobacco smoking \|\| id:ukb-b-2134 | rs71491831 | A | G | 0.075719 | 0.0305743 | 0.00510011 |
| Past tobacco smoking \|\| id:ukb-b-2134 | rs73229090 | A | C | 0.117859 | 0.0288784 | 0.0042275 |
| Past tobacco smoking \|\| id:ukb-b-2134 | rs7333559 | A | G | 0.788658 | 0.0199661 | 0.00332049 |
| Past tobacco smoking \|\| id:ukb-b-2134 | rs74676797 | A | G | 0.80811 | -0.0233457 | 0.00352676 |
| Past tobacco smoking \|\| id:ukb-b-2134 | rs7582445 | C | A | 0.587784 | -0.0172231 | 0.00273796 |
| Past tobacco smoking \|\| id:ukb-b-2134 | rs7600005 | A | G | 0.373357 | 0.0156078 | 0.00280252 |
| Past tobacco smoking \|\| id:ukb-b-2134 | rs7609050 | C | A | 0.525594 | -0.0157639 | 0.00269618 |
| Past tobacco smoking \|\| id:ukb-b-2134 | rs762995 | G | A | 0.535061 | 0.0148727 | 0.00270202 |
| Past tobacco smoking \|\| id:ukb-b-2134 | rs763053 | C | T | 0.226141 | 0.0194888 | 0.00323593 |
| Past tobacco smoking \|\| id:ukb-b-2134 | rs76608582 | A | C | 0.047264 | 0.0429722 | 0.00666659 |
| Past tobacco smoking \|\| id:ukb-b-2134 | rs77068442 | G | A | 0.107228 | -0.0239119 | 0.00435686 |
| Past tobacco smoking \|\| id:ukb-b-2134 | rs77304846 | C | T | 0.183007 | 0.0208584 | 0.00348154 |
| Past tobacco smoking \|\| id:ukb-b-2134 | rs7733542 | G | A | 0.629381 | -0.0156617 | 0.0028111 |
| Past tobacco smoking \|\| id:ukb-b-2134 | rs7901348 | G | T | 0.552343 | 0.0192949 | 0.00272924 |
| Past tobacco smoking \|\| id:ukb-b-2134 | rs7969559 | G | A | 0.720515 | 0.0173419 | 0.00300057 |
| Past tobacco smoking \|\| id:ukb-b-2134 | rs8034783 | T | C | 0.101047 | -0.0282475 | 0.0044944 |
| Past tobacco smoking \|\| id:ukb-b-2134 | rs8071295 | A | C | 0.157348 | 0.0225071 | 0.00371608 |
| Past tobacco smoking \|\| id:ukb-b-2134 | rs885011 | C | T | 0.501337 | 0.020272 | 0.00269531 |
| Past tobacco smoking \|\| id:ukb-b-2134 | rs899631 | T | G | 0.391204 | 0.0176508 | 0.00276804 |
| Past tobacco smoking \|\| id:ukb-b-2134 | rs911773 | C | A | 0.489521 | 0.0152558 | 0.00269417 |
| Past tobacco smoking \|\| id:ukb-b-2134 | rs9299331 | C | T | 0.526134 | -0.0174941 | 0.00269915 |
| Past tobacco smoking \|\| id:ukb-b-2134 | rs9375371 | A | G | 0.269381 | -0.0198988 | 0.00304394 |
| Past tobacco smoking \|\| id:ukb-b-2134 | rs9381919 | T | G | 0.103581 | 0.0270628 | 0.00442463 |
| Past tobacco smoking \|\| id:ukb-b-2134 | rs9542750 | C | T | 0.585734 | 0.0151549 | 0.00275108 |
| Past tobacco smoking \|\| id:ukb-b-2134 | rs963354 | A | C | 0.67342 | -0.0176444 | 0.00287718 |
| Smoking status: Never \|\| id:ukb-d-20116_0 | rs10193706 | C | A | 0.52527 | -0.0117667 | 0.00116951 |
| Smoking status: Never \|\| id:ukb-d-20116_0 | rs10233018 | G | A | 0.504194 | -0.00765512 | 0.00116273 |
| Smoking status: Never \|\| id:ukb-d-20116_0 | rs10274594 | G | T | 0.49589 | 0.00783264 | 0.00116705 |
| Smoking status: Never \|\| id:ukb-d-20116_0 | rs1029986 | T | C | 0.584921 | -0.00702078 | 0.00119529 |
| Smoking status: Never \|\| id:ukb-d-20116_0 | rs10774625 | G | A | 0.504146 | 0.00748682 | 0.00116086 |
| Smoking status: Never \|\| id:ukb-d-20116_0 | rs10813628 | C | T | 0.515133 | -0.00687611 | 0.00116854 |
| Smoking status: Never \|\| id:ukb-d-20116_0 | rs10897561 | C | T | 0.356457 | -0.00669175 | 0.0012233 |
| Smoking status: Never \|\| id:ukb-d-20116_0 | rs10905461 | C | T | 0.744192 | 0.00727309 | 0.00133206 |
| Smoking status: Never \|\| id:ukb-d-20116_0 | rs10914684 | A | G | 0.323978 | 0.00773564 | 0.00124102 |
| Smoking status: Never \|\| id:ukb-d-20116_0 | rs10956808 | G | T | 0.422245 | 0.00762466 | 0.00117999 |
| Smoking status: Never \|\| id:ukb-d-20116_0 | rs11103667 | T | C | 0.19203 | -0.00860468 | 0.00148601 |
| Smoking status: Never \|\| id:ukb-d-20116_0 | rs11127913 | C | T | 0.390179 | 0.00818013 | 0.00118974 |
| Smoking status: Never \|\| id:ukb-d-20116_0 | rs11611651 | A | G | 0.0877105 | -0.0119868 | 0.00205187 |
| Smoking status: Never \|\| id:ukb-d-20116_0 | rs11631530 | T | C | 0.118063 | -0.00998634 | 0.0017975 |
| Smoking status: Never \|\| id:ukb-d-20116_0 | rs11646575 | A | G | 0.438738 | -0.00824462 | 0.00117201 |
| Smoking status: Never \|\| id:ukb-d-20116_0 | rs12042107 | C | T | 0.549855 | 0.00717593 | 0.00116969 |
| Smoking status: Never \|\| id:ukb-d-20116_0 | rs12450028 | T | C | 0.34473 | -0.00706256 | 0.00122117 |
| Smoking status: Never \|\| id:ukb-d-20116_0 | rs12479064 | T | C | 0.208302 | -0.00803624 | 0.00146761 |
| Smoking status: Never \|\| id:ukb-d-20116_0 | rs12487411 | A | G | 0.472062 | 0.00750479 | 0.00116511 |
| Smoking status: Never \|\| id:ukb-d-20116_0 | rs12608052 | T | C | 0.51967 | 0.00675418 | 0.00116369 |
| Smoking status: Never \|\| id:ukb-d-20116_0 | rs12910916 | T | C | 0.212134 | -0.00901384 | 0.00141981 |
| Smoking status: Never \|\| id:ukb-d-20116_0 | rs1499982 | T | C | 0.851277 | -0.0114648 | 0.00164302 |
| Smoking status: Never \|\| id:ukb-d-20116_0 | rs1561195 | A | C | 0.522672 | -0.00789474 | 0.00118783 |
| Smoking status: Never \|\| id:ukb-d-20116_0 | rs16951001 | T | G | 0.418602 | -0.00660348 | 0.00117938 |
| Smoking status: Never \|\| id:ukb-d-20116_0 | rs17003752 | G | A | 0.138688 | 0.0098606 | 0.00169968 |
| Smoking status: Never \|\| id:ukb-d-20116_0 | rs17151637 | T | C | 0.281244 | 0.00751119 | 0.00129198 |
| Smoking status: Never \|\| id:ukb-d-20116_0 | rs1899896 | T | C | 0.297575 | -0.00799277 | 0.00127424 |
| Smoking status: Never \|\| id:ukb-d-20116_0 | rs2416770 | T | C | 0.53106 | -0.00648883 | 0.0011672 |
| Smoking status: Never \|\| id:ukb-d-20116_0 | rs2675609 | C | T | 0.627493 | 0.00815862 | 0.00120651 |
| Smoking status: Never \|\| id:ukb-d-20116_0 | rs2797116 | C | T | 0.267201 | 0.00791361 | 0.00131276 |
| Smoking status: Never \|\| id:ukb-d-20116_0 | rs2867749 | A | C | 0.318854 | 0.00694461 | 0.0012472 |
| Smoking status: Never \|\| id:ukb-d-20116_0 | rs299688 | T | G | 0.715841 | -0.00727214 | 0.00131154 |
| Smoking status: Never \|\| id:ukb-d-20116_0 | rs326341 | A | G | 0.476014 | 0.00658089 | 0.00116763 |
| Smoking status: Never \|\| id:ukb-d-20116_0 | rs35891966 | A | G | 0.0721131 | 0.0147752 | 0.00224378 |
| Smoking status: Never \|\| id:ukb-d-20116_0 | rs379525 | T | C | 0.48132 | -0.00649056 | 0.00119032 |
| Smoking status: Never \|\| id:ukb-d-20116_0 | rs42417 | T | C | 0.690191 | -0.00703306 | 0.00126258 |
| Smoking status: Never \|\| id:ukb-d-20116_0 | rs4566215 | C | A | 0.532626 | 0.00662191 | 0.00117234 |
| Smoking status: Never \|\| id:ukb-d-20116_0 | rs4910656 | C | T | 0.341569 | 0.00684378 | 0.00122755 |
| Smoking status: Never \|\| id:ukb-d-20116_0 | rs4957528 | C | A | 0.791955 | -0.00847499 | 0.00144577 |
| Smoking status: Never \|\| id:ukb-d-20116_0 | rs523528 | T | C | 0.585719 | 0.00807083 | 0.00119225 |
| Smoking status: Never \|\| id:ukb-d-20116_0 | rs528301 | A | G | 0.552033 | -0.00860082 | 0.00116774 |
| Smoking status: Never \|\| id:ukb-d-20116_0 | rs55921136 | C | T | 0.203088 | 0.00859503 | 0.00144804 |
| Smoking status: Never \|\| id:ukb-d-20116_0 | rs6141314 | A | G | 0.242466 | -0.00806157 | 0.00136376 |
| Smoking status: Never \|\| id:ukb-d-20116_0 | rs6265 | T | C | 0.188685 | 0.0101598 | 0.00148406 |
| Smoking status: Never \|\| id:ukb-d-20116_0 | rs6433897 | C | T | 0.736673 | -0.00723535 | 0.00132022 |
| Smoking status: Never \|\| id:ukb-d-20116_0 | rs6676022 | T | C | 0.121622 | 0.0115926 | 0.00177885 |
| Smoking status: Never \|\| id:ukb-d-20116_0 | rs6690680 | C | T | 0.157765 | 0.00884091 | 0.00159669 |
| Smoking status: Never \|\| id:ukb-d-20116_0 | rs72505558 | G | A | 0.39864 | 0.00674371 | 0.00118834 |
| Smoking status: Never \|\| id:ukb-d-20116_0 | rs72678864 | A | G | 0.173833 | 0.00975375 | 0.00153266 |
| Smoking status: Never \|\| id:ukb-d-20116_0 | rs7333559 | A | G | 0.790323 | 0.00805229 | 0.00143597 |
| Smoking status: Never \|\| id:ukb-d-20116_0 | rs748828 | T | C | 0.283101 | 0.00862128 | 0.00128896 |
| Smoking status: Never \|\| id:ukb-d-20116_0 | rs7528604 | A | G | 0.433285 | 0.00686585 | 0.00117661 |
| Smoking status: Never \|\| id:ukb-d-20116_0 | rs7567570 | C | T | 0.826905 | -0.00913239 | 0.00153323 |
| Smoking status: Never \|\| id:ukb-d-20116_0 | rs763053 | C | T | 0.224572 | 0.00806179 | 0.00139707 |
| Smoking status: Never \|\| id:ukb-d-20116_0 | rs76608582 | A | C | 0.0477092 | 0.0182891 | 0.00286008 |
| Smoking status: Never \|\| id:ukb-d-20116_0 | rs772921 | T | C | 0.343109 | 0.00727251 | 0.00122298 |
| Smoking status: Never \|\| id:ukb-d-20116_0 | rs7870475 | C | T | 0.474294 | -0.00718996 | 0.00116303 |
| Smoking status: Never \|\| id:ukb-d-20116_0 | rs7948789 | G | A | 0.386092 | -0.0161713 | 0.00119667 |
| Smoking status: Never \|\| id:ukb-d-20116_0 | rs883403 | C | T | 0.154118 | 0.00942401 | 0.00160739 |
| Smoking status: Never \|\| id:ukb-d-20116_0 | rs9375371 | A | G | 0.268873 | -0.00739626 | 0.00131224 |
| Smoking status: Never \|\| id:ukb-d-20116_0 | rs9381917 | A | G | 0.102086 | 0.0112569 | 0.00191907 |
| Smoking status: Never \|\| id:ukb-d-20116_0 | rs9487626 | T | C | 0.817783 | 0.0131029 | 0.00150447 |
| Years of schooling \|\| id:ieu-a-1239 | rs10073890 | G | A | 0.7364 | -0.01262 | 0.00196 |
| Years of schooling \|\| id:ieu-a-1239 | rs1008078 | T | C | 0.4099 | -0.01738 | 0.00173 |
| Years of schooling \|\| id:ieu-a-1239 | rs10189857 | G | A | 0.4184 | -0.01725 | 0.00171 |
| Years of schooling \|\| id:ieu-a-1239 | rs10191758 | G | A | 0.381 | 0.01631 | 0.00175 |
| Years of schooling \|\| id:ieu-a-1239 | rs10205801 | A | G | 0.5068 | -0.01053 | 0.00171 |
| Years of schooling \|\| id:ieu-a-1239 | rs10215082 | G | A | 0.5612 | 0.01303 | 0.00172 |
| Years of schooling \|\| id:ieu-a-1239 | rs10240905 | C | T | 0.6684 | 0.01167 | 0.00177 |
| Years of schooling \|\| id:ieu-a-1239 | rs10456918 | C | A | 0.182 | 0.01485 | 0.00224 |
| Years of schooling \|\| id:ieu-a-1239 | rs10460095 | A | G | 0.5867 | -0.01066 | 0.00171 |
| Years of schooling \|\| id:ieu-a-1239 | rs1051474 | C | T | 0.2738 | 0.01301 | 0.00188 |
| Years of schooling \|\| id:ieu-a-1239 | rs10765775 | A | G | 0.3963 | 0.01488 | 0.00176 |
| Years of schooling \|\| id:ieu-a-1239 | rs10798418 | T | C | 0.466 | -0.00957 | 0.00173 |
| Years of schooling \|\| id:ieu-a-1239 | rs10856785 | T | C | 0.7296 | -0.01132 | 0.00192 |
| Years of schooling \|\| id:ieu-a-1239 | rs10887801 | T | G | 0.4371 | 0.01087 | 0.00171 |
| Years of schooling \|\| id:ieu-a-1239 | rs10940921 | G | T | 0.5697 | -0.01089 | 0.00177 |
| Years of schooling \|\| id:ieu-a-1239 | rs10994777 | A | G | 0.1395 | 0.0146 | 0.00232 |
| Years of schooling \|\| id:ieu-a-1239 | rs11023749 | A | G | 0.6701 | 0.01132 | 0.0018 |
| Years of schooling \|\| id:ieu-a-1239 | rs1105307 | A | G | 0.2449 | -0.01173 | 0.00195 |
| Years of schooling \|\| id:ieu-a-1239 | rs1106090 | A | G | 0.6259 | 0.01173 | 0.00175 |
| Years of schooling \|\| id:ieu-a-1239 | rs11081529 | C | T | 0.2568 | -0.01311 | 0.00186 |
| Years of schooling \|\| id:ieu-a-1239 | rs11123818 | A | G | 0.3946 | 0.02081 | 0.00175 |
| Years of schooling \|\| id:ieu-a-1239 | rs111821073 | T | C | 0.1633 | 0.01385 | 0.00237 |
| Years of schooling \|\| id:ieu-a-1239 | rs112687095 | A | G | 0.165 | 0.01325 | 0.00238 |
| Years of schooling \|\| id:ieu-a-1239 | rs113182709 | A | G | 0.02381 | 0.03225 | 0.00567 |
| Years of schooling \|\| id:ieu-a-1239 | rs113520408 | A | G | 0.2857 | 0.01304 | 0.00192 |
| Years of schooling \|\| id:ieu-a-1239 | rs113615161 | T | C | 0.1395 | -0.01472 | 0.0025 |
| Years of schooling \|\| id:ieu-a-1239 | rs1143770 | T | C | 0.5918 | 0.01136 | 0.00172 |
| Years of schooling \|\| id:ieu-a-1239 | rs115454970 | T | G | 0.3044 | -0.01185 | 0.00199 |
| Years of schooling \|\| id:ieu-a-1239 | rs11601122 | G | A | 0.1497 | -0.01947 | 0.0023 |
| Years of schooling \|\| id:ieu-a-1239 | rs11620355 | A | G | 0.1156 | 0.01756 | 0.003 |
| Years of schooling \|\| id:ieu-a-1239 | rs11627087 | G | A | 0.08503 | -0.01788 | 0.00325 |
| Years of schooling \|\| id:ieu-a-1239 | rs11635092 | A | G | 0.3639 | -0.01231 | 0.00177 |
| Years of schooling \|\| id:ieu-a-1239 | rs11657342 | A | G | 0.3554 | 0.01404 | 0.00191 |
| Years of schooling \|\| id:ieu-a-1239 | rs11663602 | A | C | 0.2568 | -0.01213 | 0.0019 |
| Years of schooling \|\| id:ieu-a-1239 | rs11678980 | A | G | 0.4456 | -0.01744 | 0.00172 |
| Years of schooling \|\| id:ieu-a-1239 | rs11681861 | G | T | 0.1565 | -0.01435 | 0.00259 |
| Years of schooling \|\| id:ieu-a-1239 | rs11694904 | T | C | 0.3384 | 0.01215 | 0.00185 |
| Years of schooling \|\| id:ieu-a-1239 | rs11732657 | A | G | 0.7007 | -0.01274 | 0.00197 |
| Years of schooling \|\| id:ieu-a-1239 | rs117468730 | A | G | 0.0119 | -0.03521 | 0.00597 |
| Years of schooling \|\| id:ieu-a-1239 | rs11752914 | C | T | 0.1973 | -0.01208 | 0.00216 |
| Years of schooling \|\| id:ieu-a-1239 | rs11772580 | T | G | 0.2534 | -0.01199 | 0.00201 |
| Years of schooling \|\| id:ieu-a-1239 | rs11871429 | G | A | 0.2041 | -0.01425 | 0.00202 |
| Years of schooling \|\| id:ieu-a-1239 | rs12028010 | C | T | 0.2228 | -0.01696 | 0.00202 |
| Years of schooling \|\| id:ieu-a-1239 | rs12375949 | C | T | 0.5697 | 0.01447 | 0.00172 |
| Years of schooling \|\| id:ieu-a-1239 | rs12468040 | G | T | 0.6037 | -0.01432 | 0.00175 |
| Years of schooling \|\| id:ieu-a-1239 | rs12503522 | T | C | 0.2483 | -0.01125 | 0.00188 |
| Years of schooling \|\| id:ieu-a-1239 | rs12519073 | T | C | 0.2381 | -0.01221 | 0.00202 |
| Years of schooling \|\| id:ieu-a-1239 | rs12574281 | C | A | 0.3997 | 0.01077 | 0.00176 |
| Years of schooling \|\| id:ieu-a-1239 | rs12602286 | T | G | 0.8861 | 0.01701 | 0.00255 |
| Years of schooling \|\| id:ieu-a-1239 | rs12643771 | T | C | 0.3112 | 0.01518 | 0.00184 |
| Years of schooling \|\| id:ieu-a-1239 | rs12682775 | C | T | 0.2143 | 0.01187 | 0.00204 |
| Years of schooling \|\| id:ieu-a-1239 | rs12804787 | G | A | 0.06803 | -0.01814 | 0.00327 |
| Years of schooling \|\| id:ieu-a-1239 | rs1291818 | C | T | 0.5153 | -0.01085 | 0.0017 |
| Years of schooling \|\| id:ieu-a-1239 | rs12940014 | C | T | 0.5204 | 0.00936 | 0.0017 |
| Years of schooling \|\| id:ieu-a-1239 | rs13010566 | C | A | 0.5612 | 0.0106 | 0.0017 |
| Years of schooling \|\| id:ieu-a-1239 | rs13029509 | A | G | 0.4677 | -0.01049 | 0.0017 |
| Years of schooling \|\| id:ieu-a-1239 | rs13090388 | T | C | 0.3095 | 0.02852 | 0.00184 |
| Years of schooling \|\| id:ieu-a-1239 | rs13141210 | T | C | 0.5085 | 0.01361 | 0.00172 |
| Years of schooling \|\| id:ieu-a-1239 | rs13145650 | T | C | 0.90816 | -0.01918 | 0.00306 |
| Years of schooling \|\| id:ieu-a-1239 | rs13422673 | T | C | 0.4847 | -0.01201 | 0.0017 |
| Years of schooling \|\| id:ieu-a-1239 | rs1363862 | A | G | 0.2602 | -0.01171 | 0.00192 |
| Years of schooling \|\| id:ieu-a-1239 | rs1381247 | C | T | 0.2908 | -0.01013 | 0.00182 |
| Years of schooling \|\| id:ieu-a-1239 | rs1391438 | C | T | 0.6854 | -0.0167 | 0.00183 |
| Years of schooling \|\| id:ieu-a-1239 | rs1427298 | T | C | 0.4116 | 0.0102 | 0.00172 |
| Years of schooling \|\| id:ieu-a-1239 | rs1450782 | G | T | 0.602 | -0.00945 | 0.00173 |
| Years of schooling \|\| id:ieu-a-1239 | rs152603 | G | A | 0.3861 | 0.01019 | 0.00177 |
| Years of schooling \|\| id:ieu-a-1239 | rs1558727 | T | C | 0.4847 | -0.01069 | 0.0017 |
| Years of schooling \|\| id:ieu-a-1239 | rs1566085 | T | G | 0.5697 | 0.01645 | 0.00171 |
| Years of schooling \|\| id:ieu-a-1239 | rs1569092 | A | G | 0.182 | 0.01807 | 0.00234 |
| Years of schooling \|\| id:ieu-a-1239 | rs1584469 | T | C | 0.3112 | -0.01303 | 0.00185 |
| Years of schooling \|\| id:ieu-a-1239 | rs1595973 | T | C | 0.5595 | -0.01002 | 0.00173 |
| Years of schooling \|\| id:ieu-a-1239 | rs1618725 | T | C | 0.5204 | 0.01477 | 0.00174 |
| Years of schooling \|\| id:ieu-a-1239 | rs1671770 | C | A | 0.8061 | -0.01342 | 0.00223 |
| Years of schooling \|\| id:ieu-a-1239 | rs16846463 | G | A | 0.1173 | -0.02256 | 0.00283 |
| Years of schooling \|\| id:ieu-a-1239 | rs16854920 | C | T | 0.3554 | 0.01007 | 0.00181 |
| Years of schooling \|\| id:ieu-a-1239 | rs16995054 | T | C | 0.2007 | -0.0139 | 0.00208 |
| Years of schooling \|\| id:ieu-a-1239 | rs17048855 | A | G | 0.3248 | 0.01184 | 0.00179 |
| Years of schooling \|\| id:ieu-a-1239 | rs17110109 | C | T | 0.3776 | 0.01023 | 0.00175 |
| Years of schooling \|\| id:ieu-a-1239 | rs17126938 | C | T | 0.1207 | 0.01536 | 0.0025 |
| Years of schooling \|\| id:ieu-a-1239 | rs17425572 | G | A | 0.5425 | -0.01224 | 0.0017 |
| Years of schooling \|\| id:ieu-a-1239 | rs17489649 | G | A | 0.3265 | -0.0139 | 0.00181 |
| Years of schooling \|\| id:ieu-a-1239 | rs17551064 | G | A | 0.1599 | -0.01493 | 0.0023 |
| Years of schooling \|\| id:ieu-a-1239 | rs17563464 | A | C | 0.2041 | -0.01477 | 0.00212 |
| Years of schooling \|\| id:ieu-a-1239 | rs17565975 | A | G | 0.5306 | -0.01142 | 0.00171 |
| Years of schooling \|\| id:ieu-a-1239 | rs17598675 | C | T | 0.5187 | 0.01199 | 0.0017 |
| Years of schooling \|\| id:ieu-a-1239 | rs176218 | T | G | 0.2007 | 0.01883 | 0.00215 |
| Years of schooling \|\| id:ieu-a-1239 | rs1827540 | G | A | 0.466 | -0.0106 | 0.0017 |
| Years of schooling \|\| id:ieu-a-1239 | rs1866823 | A | G | 0.551 | 0.01009 | 0.00171 |
| Years of schooling \|\| id:ieu-a-1239 | rs192436652 | T | C | 0.02211 | -0.03497 | 0.00545 |
| Years of schooling \|\| id:ieu-a-1239 | rs1925576 | G | A | 0.4422 | 0.00997 | 0.00171 |
| Years of schooling \|\| id:ieu-a-1239 | rs1947114 | G | A | 0.2551 | 0.01071 | 0.00192 |
| Years of schooling \|\| id:ieu-a-1239 | rs1964927 | G | A | 0.6378 | -0.01423 | 0.00177 |
| Years of schooling \|\| id:ieu-a-1239 | rs2052285 | A | G | 0.5765 | 0.01123 | 0.00175 |
| Years of schooling \|\| id:ieu-a-1239 | rs2067854 | A | G | 0.182 | 0.01477 | 0.00209 |
| Years of schooling \|\| id:ieu-a-1239 | rs2179152 | C | T | 0.6429 | 0.01455 | 0.00176 |
| Years of schooling \|\| id:ieu-a-1239 | rs2182505 | C | T | 0.7364 | -0.01086 | 0.00192 |
| Years of schooling \|\| id:ieu-a-1239 | rs225291 | G | A | 0.8027 | -0.01205 | 0.00214 |
| Years of schooling \|\| id:ieu-a-1239 | rs2256965 | G | A | 0.5425 | -0.01128 | 0.00176 |
| Years of schooling \|\| id:ieu-a-1239 | rs2283076 | G | A | 0.2143 | -0.01143 | 0.00204 |
| Years of schooling \|\| id:ieu-a-1239 | rs2287838 | A | G | 0.534 | -0.01152 | 0.00171 |
| Years of schooling \|\| id:ieu-a-1239 | rs2302761 | T | C | 0.1905 | 0.01354 | 0.00209 |
| Years of schooling \|\| id:ieu-a-1239 | rs2347526 | C | T | 0.6378 | 0.01395 | 0.00179 |
| Years of schooling \|\| id:ieu-a-1239 | rs242093 | A | G | 0.5476 | -0.01031 | 0.00172 |
| Years of schooling \|\| id:ieu-a-1239 | rs2554835 | A | G | 0.398 | 0.00974 | 0.00175 |
| Years of schooling \|\| id:ieu-a-1239 | rs2570497 | T | C | 0.6735 | -0.01233 | 0.00177 |
| Years of schooling \|\| id:ieu-a-1239 | rs2725370 | C | T | 0.7109 | 0.01536 | 0.00187 |
| Years of schooling \|\| id:ieu-a-1239 | rs277828 | A | C | 0.2568 | -0.01091 | 0.00196 |
| Years of schooling \|\| id:ieu-a-1239 | rs2787101 | T | C | 0.6173 | 0.00968 | 0.00174 |
| Years of schooling \|\| id:ieu-a-1239 | rs2819336 | C | T | 0.6616 | -0.01828 | 0.00177 |
| Years of schooling \|\| id:ieu-a-1239 | rs2820314 | C | A | 0.3163 | -0.011 | 0.0018 |
| Years of schooling \|\| id:ieu-a-1239 | rs28513670 | G | A | 0.1531 | 0.01477 | 0.00225 |
| Years of schooling \|\| id:ieu-a-1239 | rs2885198 | G | A | 0.5017 | -0.01025 | 0.0017 |
| Years of schooling \|\| id:ieu-a-1239 | rs2901616 | A | G | 0.517 | 0.00941 | 0.00171 |
| Years of schooling \|\| id:ieu-a-1239 | rs2905426 | T | G | 0.6446 | 0.01037 | 0.00181 |
| Years of schooling \|\| id:ieu-a-1239 | rs2971970 | G | T | 0.7721 | 0.01654 | 0.00207 |
| Years of schooling \|\| id:ieu-a-1239 | rs2998315 | G | A | 0.5782 | 0.01269 | 0.00171 |
| Years of schooling \|\| id:ieu-a-1239 | rs3013014 | A | G | 0.6105 | -0.01024 | 0.00172 |
| Years of schooling \|\| id:ieu-a-1239 | rs301800 | C | T | 0.8197 | -0.01516 | 0.00224 |
| Years of schooling \|\| id:ieu-a-1239 | rs3026996 | C | A | 0.284 | -0.01537 | 0.00199 |
| Years of schooling \|\| id:ieu-a-1239 | rs31940 | A | G | 0.1344 | 0.01548 | 0.00246 |
| Years of schooling \|\| id:ieu-a-1239 | rs337637 | A | G | 0.3367 | 0.01123 | 0.00177 |
| Years of schooling \|\| id:ieu-a-1239 | rs34394051 | G | A | 0.1599 | 0.01392 | 0.0024 |
| Years of schooling \|\| id:ieu-a-1239 | rs34485537 | T | C | 0.3895 | 0.01075 | 0.00173 |
| Years of schooling \|\| id:ieu-a-1239 | rs35039375 | G | A | 0.09354 | -0.01983 | 0.00293 |
| Years of schooling \|\| id:ieu-a-1239 | rs35309068 | G | T | 0.466 | 0.01321 | 0.00171 |
| Years of schooling \|\| id:ieu-a-1239 | rs35316276 | T | C | 0.2942 | 0.01173 | 0.00194 |
| Years of schooling \|\| id:ieu-a-1239 | rs35417702 | T | C | 0.5765 | -0.01445 | 0.0017 |
| Years of schooling \|\| id:ieu-a-1239 | rs35475880 | T | G | 0.1837 | -0.01511 | 0.00208 |
| Years of schooling \|\| id:ieu-a-1239 | rs36083520 | C | T | 0.1667 | 0.01629 | 0.00223 |
| Years of schooling \|\| id:ieu-a-1239 | rs36119825 | A | G | 0.4694 | 0.01063 | 0.00171 |
| Years of schooling \|\| id:ieu-a-1239 | rs363096 | C | T | 0.5748 | 0.01363 | 0.00172 |
| Years of schooling \|\| id:ieu-a-1239 | rs3788556 | C | T | 0.5408 | -0.01138 | 0.00171 |
| Years of schooling \|\| id:ieu-a-1239 | rs3809634 | G | A | 0.335 | 0.01058 | 0.00185 |
| Years of schooling \|\| id:ieu-a-1239 | rs3890802 | A | G | 0.2687 | -0.01133 | 0.00191 |
| Years of schooling \|\| id:ieu-a-1239 | rs3897821 | G | A | 0.3503 | -0.01502 | 0.0018 |
| Years of schooling \|\| id:ieu-a-1239 | rs4073894 | A | G | 0.1769 | 0.01524 | 0.00211 |
| Years of schooling \|\| id:ieu-a-1239 | rs4328757 | T | C | 0.6514 | 0.01067 | 0.00174 |
| Years of schooling \|\| id:ieu-a-1239 | rs4352658 | T | C | 0.09014 | -0.0212 | 0.00308 |
| Years of schooling \|\| id:ieu-a-1239 | rs4369924 | A | G | 0.1684 | 0.01362 | 0.00234 |
| Years of schooling \|\| id:ieu-a-1239 | rs4382592 | G | T | 0.699 | 0.01636 | 0.00185 |
| Years of schooling \|\| id:ieu-a-1239 | rs4384309 | A | G | 0.4796 | 0.0109 | 0.00172 |
| Years of schooling \|\| id:ieu-a-1239 | rs4392737 | G | A | 0.3827 | -0.0097 | 0.00173 |
| Years of schooling \|\| id:ieu-a-1239 | rs4442732 | G | A | 0.5969 | -0.01063 | 0.00176 |
| Years of schooling \|\| id:ieu-a-1239 | rs4667025 | A | G | 0.3878 | 0.00957 | 0.00174 |
| Years of schooling \|\| id:ieu-a-1239 | rs4700393 | G | A | 0.5289 | 0.02086 | 0.0017 |
| Years of schooling \|\| id:ieu-a-1239 | rs4726070 | A | G | 0.6207 | 0.01251 | 0.00174 |
| Years of schooling \|\| id:ieu-a-1239 | rs4778058 | C | T | 0.5221 | 0.01017 | 0.0017 |
| Years of schooling \|\| id:ieu-a-1239 | rs4787457 | G | A | 0.3146 | -0.01741 | 0.00176 |
| Years of schooling \|\| id:ieu-a-1239 | rs4810227 | A | G | 0.6344 | 0.01272 | 0.00175 |
| Years of schooling \|\| id:ieu-a-1239 | rs4839155 | G | T | 0.25 | -0.01251 | 0.002 |
| Years of schooling \|\| id:ieu-a-1239 | rs4846724 | A | G | 0.4915 | 0.01018 | 0.0017 |
| Years of schooling \|\| id:ieu-a-1239 | rs4888746 | G | A | 0.3776 | -0.00952 | 0.00174 |
| Years of schooling \|\| id:ieu-a-1239 | rs4904523 | A | G | 0.4592 | -0.00936 | 0.0017 |
| Years of schooling \|\| id:ieu-a-1239 | rs4945424 | A | C | 0.415 | -0.00992 | 0.00171 |
| Years of schooling \|\| id:ieu-a-1239 | rs4964046 | G | A | 0.335 | 0.01053 | 0.00178 |
| Years of schooling \|\| id:ieu-a-1239 | rs4972400 | A | G | 0.352 | 0.01156 | 0.00181 |
| Years of schooling \|\| id:ieu-a-1239 | rs4984541 | G | A | 0.2415 | 0.01233 | 0.00207 |
| Years of schooling \|\| id:ieu-a-1239 | rs535307 | G | A | 0.6769 | -0.01004 | 0.00184 |
| Years of schooling \|\| id:ieu-a-1239 | rs56391344 | A | G | 0.2381 | 0.01571 | 0.00197 |
| Years of schooling \|\| id:ieu-a-1239 | rs575113 | A | G | 0.2772 | 0.01285 | 0.00186 |
| Years of schooling \|\| id:ieu-a-1239 | rs59123361 | A | G | 0.1054 | -0.02094 | 0.00291 |
| Years of schooling \|\| id:ieu-a-1239 | rs6122735 | T | C | 0.4133 | 0.0105 | 0.00174 |
| Years of schooling \|\| id:ieu-a-1239 | rs6123924 | G | A | 0.1599 | -0.01528 | 0.00235 |
| Years of schooling \|\| id:ieu-a-1239 | rs613872 | T | G | 0.8282 | -0.0175 | 0.00227 |
| Years of schooling \|\| id:ieu-a-1239 | rs62097985 | T | C | 0.4116 | -0.01288 | 0.00172 |
| Years of schooling \|\| id:ieu-a-1239 | rs62157915 | C | T | 0.05952 | 0.02091 | 0.00348 |
| Years of schooling \|\| id:ieu-a-1239 | rs62183776 | T | C | 0.1905 | -0.01308 | 0.00217 |
| Years of schooling \|\| id:ieu-a-1239 | rs62184480 | T | C | 0.2449 | -0.01528 | 0.00191 |
| Years of schooling \|\| id:ieu-a-1239 | rs622169 | T | C | 0.4677 | 0.00999 | 0.00178 |
| Years of schooling \|\| id:ieu-a-1239 | rs62439690 | A | G | 0.267 | -0.01087 | 0.00194 |
| Years of schooling \|\| id:ieu-a-1239 | rs62444881 | T | C | 0.1905 | 0.01815 | 0.00217 |
| Years of schooling \|\| id:ieu-a-1239 | rs6493265 | T | C | 0.3895 | -0.01385 | 0.00174 |
| Years of schooling \|\| id:ieu-a-1239 | rs6513959 | G | A | 0.2789 | -0.01177 | 0.00185 |
| Years of schooling \|\| id:ieu-a-1239 | rs6557171 | C | T | 0.7245 | 0.01567 | 0.00181 |
| Years of schooling \|\| id:ieu-a-1239 | rs66568921 | G | T | 0.3639 | 0.01565 | 0.00182 |
| Years of schooling \|\| id:ieu-a-1239 | rs6731373 | A | G | 0.3367 | -0.01256 | 0.00181 |
| Years of schooling \|\| id:ieu-a-1239 | rs67885444 | T | C | 0.1718 | 0.01406 | 0.00232 |
| Years of schooling \|\| id:ieu-a-1239 | rs67890737 | A | C | 0.3265 | -0.01141 | 0.00179 |
| Years of schooling \|\| id:ieu-a-1239 | rs6803651 | T | G | 0.415 | 0.01131 | 0.00172 |
| Years of schooling \|\| id:ieu-a-1239 | rs6805241 | C | T | 0.1973 | -0.01413 | 0.00203 |
| Years of schooling \|\| id:ieu-a-1239 | rs6938002 | A | G | 0.3963 | -0.01008 | 0.00173 |
| Years of schooling \|\| id:ieu-a-1239 | rs6959891 | G | A | 0.2959 | -0.01136 | 0.00189 |
| Years of schooling \|\| id:ieu-a-1239 | rs7012546 | T | C | 0.4201 | 0.01009 | 0.00172 |
| Years of schooling \|\| id:ieu-a-1239 | rs702606 | C | T | 0.1701 | -0.01427 | 0.0025 |
| Years of schooling \|\| id:ieu-a-1239 | rs7031698 | C | T | 0.7755 | 0.01248 | 0.00206 |
| Years of schooling \|\| id:ieu-a-1239 | rs710629 | A | G | 0.6565 | 0.01053 | 0.00177 |
| Years of schooling \|\| id:ieu-a-1239 | rs71646142 | T | C | 0.1735 | 0.01286 | 0.00217 |
| Years of schooling \|\| id:ieu-a-1239 | rs7233920 | A | G | 0.216 | -0.01315 | 0.00202 |
| Years of schooling \|\| id:ieu-a-1239 | rs7257460 | C | T | 0.2704 | -0.01145 | 0.00189 |
| Years of schooling \|\| id:ieu-a-1239 | rs72807818 | A | G | 0.1241 | 0.01915 | 0.00252 |
| Years of schooling \|\| id:ieu-a-1239 | rs72828517 | C | T | 0.1412 | 0.01836 | 0.00224 |
| Years of schooling \|\| id:ieu-a-1239 | rs72840994 | G | T | 0.182 | 0.01247 | 0.00216 |
| Years of schooling \|\| id:ieu-a-1239 | rs730384 | A | G | 0.4558 | 0.01016 | 0.00171 |
| Years of schooling \|\| id:ieu-a-1239 | rs7321274 | G | A | 0.1956 | -0.01275 | 0.00211 |
| Years of schooling \|\| id:ieu-a-1239 | rs73301698 | A | G | 0.2262 | -0.01291 | 0.00208 |
| Years of schooling \|\| id:ieu-a-1239 | rs7332724 | T | C | 0.2619 | -0.01149 | 0.00189 |
| Years of schooling \|\| id:ieu-a-1239 | rs73344830 | G | A | 0.602 | -0.0172 | 0.00172 |
| Years of schooling \|\| id:ieu-a-1239 | rs736282 | C | T | 0.5153 | -0.01082 | 0.0017 |
| Years of schooling \|\| id:ieu-a-1239 | rs73874335 | T | C | 0.05952 | -0.0199 | 0.00361 |
| Years of schooling \|\| id:ieu-a-1239 | rs743316 | C | T | 0.182 | -0.01185 | 0.00208 |
| Years of schooling \|\| id:ieu-a-1239 | rs74643044 | C | T | 0.02721 | 0.02323 | 0.00386 |
| Years of schooling \|\| id:ieu-a-1239 | rs74701752 | T | G | 0.09524 | 0.01591 | 0.00285 |
| Years of schooling \|\| id:ieu-a-1239 | rs7481514 | G | A | 0.665 | 0.01072 | 0.00178 |
| Years of schooling \|\| id:ieu-a-1239 | rs74998289 | G | T | 0.2398 | -0.01821 | 0.00213 |
| Years of schooling \|\| id:ieu-a-1239 | rs7594904 | C | T | 0.4184 | 0.00969 | 0.00173 |
| Years of schooling \|\| id:ieu-a-1239 | rs7603132 | A | G | 0.1548 | 0.01317 | 0.00215 |
| Years of schooling \|\| id:ieu-a-1239 | rs76076331 | T | C | 0.131 | 0.01873 | 0.00248 |
| Years of schooling \|\| id:ieu-a-1239 | rs7650602 | C | T | 0.4286 | 0.00939 | 0.00171 |
| Years of schooling \|\| id:ieu-a-1239 | rs76608582 | A | C | 0.04082 | 0.02798 | 0.00445 |
| Years of schooling \|\| id:ieu-a-1239 | rs77025239 | A | G | 0.1088 | -0.01422 | 0.00234 |
| Years of schooling \|\| id:ieu-a-1239 | rs77128898 | T | C | 0.02211 | -0.02769 | 0.00482 |
| Years of schooling \|\| id:ieu-a-1239 | rs77702622 | A | G | 0.07653 | -0.02447 | 0.00351 |
| Years of schooling \|\| id:ieu-a-1239 | rs77835879 | G | A | 0.09014 | -0.01601 | 0.00288 |
| Years of schooling \|\| id:ieu-a-1239 | rs7796203 | A | G | 0.5255 | -0.01074 | 0.00171 |
| Years of schooling \|\| id:ieu-a-1239 | rs7803932 | A | G | 0.1565 | 0.0143 | 0.00226 |
| Years of schooling \|\| id:ieu-a-1239 | rs7808399 | G | A | 0.5476 | 0.0107 | 0.00171 |
| Years of schooling \|\| id:ieu-a-1239 | rs7863447 | A | G | 0.8333 | 0.01678 | 0.00233 |
| Years of schooling \|\| id:ieu-a-1239 | rs78721320 | A | G | 0.2041 | 0.01307 | 0.00219 |
| Years of schooling \|\| id:ieu-a-1239 | rs790647 | A | C | 0.2347 | -0.01482 | 0.00202 |
| Years of schooling \|\| id:ieu-a-1239 | rs7924036 | T | G | 0.5391 | 0.01501 | 0.0017 |
| Years of schooling \|\| id:ieu-a-1239 | rs79265434 | G | A | 0.1173 | 0.02331 | 0.00262 |
| Years of schooling \|\| id:ieu-a-1239 | rs79269403 | A | G | 0.2228 | 0.01447 | 0.00204 |
| Years of schooling \|\| id:ieu-a-1239 | rs795230 | T | C | 0.4184 | 0.00952 | 0.00172 |
| Years of schooling \|\| id:ieu-a-1239 | rs79523955 | G | A | 0.09524 | -0.01802 | 0.00283 |
| Years of schooling \|\| id:ieu-a-1239 | rs7977614 | G | A | 0.3078 | 0.01325 | 0.00198 |
| Years of schooling \|\| id:ieu-a-1239 | rs7993663 | C | T | 0.3571 | 0.0118 | 0.00178 |
| Years of schooling \|\| id:ieu-a-1239 | rs80171383 | A | G | 0.1241 | 0.0145 | 0.00241 |
| Years of schooling \|\| id:ieu-a-1239 | rs8020034 | A | G | 0.2058 | 0.01782 | 0.00223 |
| Years of schooling \|\| id:ieu-a-1239 | rs818415 | G | T | 0.182 | 0.01235 | 0.00219 |
| Years of schooling \|\| id:ieu-a-1239 | rs837080 | C | T | 0.4932 | 0.01092 | 0.0017 |
| Years of schooling \|\| id:ieu-a-1239 | rs892612 | C | A | 0.8418 | 0.01464 | 0.00237 |
| Years of schooling \|\| id:ieu-a-1239 | rs894067 | A | G | 0.3929 | 0.01041 | 0.00175 |
| Years of schooling \|\| id:ieu-a-1239 | rs9289300 | C | T | 0.1837 | 0.01512 | 0.00234 |
| Years of schooling \|\| id:ieu-a-1239 | rs9320493 | G | A | 0.8639 | -0.01394 | 0.0024 |
| Years of schooling \|\| id:ieu-a-1239 | rs9342482 | T | G | 0.2908 | 0.01264 | 0.00197 |
| Years of schooling \|\| id:ieu-a-1239 | rs9349956 | C | A | 0.2398 | 0.01881 | 0.00225 |
| Years of schooling \|\| id:ieu-a-1239 | rs9372625 | A | G | 0.4133 | 0.02383 | 0.00176 |
| Years of schooling \|\| id:ieu-a-1239 | rs9384679 | T | C | 0.4082 | -0.00959 | 0.00176 |
| Years of schooling \|\| id:ieu-a-1239 | rs9386319 | G | A | 0.4269 | 0.00991 | 0.00174 |
| Years of schooling \|\| id:ieu-a-1239 | rs9436866 | C | A | 0.09524 | 0.01882 | 0.00289 |
| Years of schooling \|\| id:ieu-a-1239 | rs9503598 | A | G | 0.4388 | 0.01079 | 0.00171 |
| Years of schooling \|\| id:ieu-a-1239 | rs9556958 | T | C | 0.5289 | -0.0108 | 0.0017 |
| Years of schooling \|\| id:ieu-a-1239 | rs9616906 | A | G | 0.4235 | 0.01497 | 0.00172 |
| Years of schooling \|\| id:ieu-a-1239 | rs9679654 | C | T | 0.4847 | 0.01042 | 0.00172 |
| Years of schooling \|\| id:ieu-a-1239 | rs9704097 | A | C | 0.4728 | -0.0103 | 0.00171 |
| Years of schooling \|\| id:ieu-a-1239 | rs9882532 | C | T | 0.3639 | -0.01208 | 0.00177 |
| Years of schooling \|\| id:ieu-a-1239 | rs9914918 | A | G | 0.2823 | 0.01155 | 0.00189 |
| Years of schooling \|\| id:ieu-a-1239 | rs9933256 | G | A | 0.4082 | -0.01134 | 0.00172 |
| Years of schooling \|\| id:ieu-a-1239 | rs9936270 | T | C | 0.3078 | -0.0136 | 0.00198 |
| Years of schooling \|\| id:ieu-a-1239 | rs9964724 | T | C | 0.6599 | 0.01978 | 0.00183 |
| Years of schooling \|\| id:ieu-a-1239 | rs9995567 | A | G | 0.3827 | 0.00998 | 0.00178 |
| Qualifications: College or University degree \|\| id:ukb-a-397 | rs1007274 | T | C | 0.214362 | -0.0075876 | 0.00139038 |
| Qualifications: College or University degree \|\| id:ukb-a-397 | rs10088132 | T | C | 0.452808 | -0.00845573 | 0.0011481 |
| Qualifications: College or University degree \|\| id:ukb-a-397 | rs10129035 | C | T | 0.595399 | -0.0076557 | 0.00116439 |
| Qualifications: College or University degree \|\| id:ukb-a-397 | rs10138733 | G | A | 0.812068 | -0.00952756 | 0.00145634 |
| Qualifications: College or University degree \|\| id:ukb-a-397 | rs10189857 | G | A | 0.432196 | -0.00963462 | 0.00115092 |
| Qualifications: College or University degree \|\| id:ukb-a-397 | rs10431632 | A | G | 0.550863 | 0.00656235 | 0.00116797 |
| Qualifications: College or University degree \|\| id:ukb-a-397 | rs10515044 | T | C | 0.461186 | 0.00629586 | 0.00114633 |
| Qualifications: College or University degree \|\| id:ukb-a-397 | rs10518019 | G | A | 0.476713 | 0.00795861 | 0.00113893 |
| Qualifications: College or University degree \|\| id:ukb-a-397 | rs1073242 | A | G | 0.553801 | 0.0116733 | 0.00116724 |
| Qualifications: College or University degree \|\| id:ukb-a-397 | rs10752262 | T | C | 0.429844 | 0.00656451 | 0.00115172 |
| Qualifications: College or University degree \|\| id:ukb-a-397 | rs10795831 | T | G | 0.207444 | -0.00771068 | 0.00140833 |
| Qualifications: College or University degree \|\| id:ukb-a-397 | rs10801826 | T | C | 0.369813 | -0.0105964 | 0.00118043 |
| Qualifications: College or University degree \|\| id:ukb-a-397 | rs10942580 | T | C | 0.145551 | 0.00978101 | 0.00161219 |
| Qualifications: College or University degree \|\| id:ukb-a-397 | rs10950862 | G | T | 0.342609 | -0.00750882 | 0.00120555 |
| Qualifications: College or University degree \|\| id:ukb-a-397 | rs11080256 | A | G | 0.32723 | 0.00676667 | 0.00121421 |
| Qualifications: College or University degree \|\| id:ukb-a-397 | rs11155821 | C | T | 0.633353 | 0.00809818 | 0.00118203 |
| Qualifications: College or University degree \|\| id:ukb-a-397 | rs1140892 | T | C | 0.0309802 | 0.0197586 | 0.00343068 |
| Qualifications: College or University degree \|\| id:ukb-a-397 | rs114460989 | T | G | 0.037079 | -0.016819 | 0.00301331 |
| Qualifications: College or University degree \|\| id:ukb-a-397 | rs11678980 | A | G | 0.463274 | -0.00975602 | 0.0011807 |
| Qualifications: College or University degree \|\| id:ukb-a-397 | rs11703948 | G | A | 0.103004 | 0.0130102 | 0.00187045 |
| Qualifications: College or University degree \|\| id:ukb-a-397 | rs11711894 | T | C | 0.285109 | 0.00757568 | 0.00128561 |
| Qualifications: College or University degree \|\| id:ukb-a-397 | rs11771370 | T | C | 0.173678 | -0.00940699 | 0.00152418 |
| Qualifications: College or University degree \|\| id:ukb-a-397 | rs11793831 | T | G | 0.416435 | 0.0145667 | 0.00115549 |
| Qualifications: College or University degree \|\| id:ukb-a-397 | rs12028010 | C | T | 0.23372 | -0.0090857 | 0.00135301 |
| Qualifications: College or University degree \|\| id:ukb-a-397 | rs12203182 | A | G | 0.224567 | 0.0083675 | 0.00144601 |
| Qualifications: College or University degree \|\| id:ukb-a-397 | rs12221820 | T | C | 0.2695 | -0.00715158 | 0.00128832 |
| Qualifications: College or University degree \|\| id:ukb-a-397 | rs12359372 | C | T | 0.330439 | 0.00717644 | 0.00121858 |
| Qualifications: College or University degree \|\| id:ukb-a-397 | rs12375949 | C | T | 0.561019 | 0.00826335 | 0.00114573 |
| Qualifications: College or University degree \|\| id:ukb-a-397 | rs12523278 | G | A | 0.49888 | 0.010205 | 0.00114625 |
| Qualifications: College or University degree \|\| id:ukb-a-397 | rs12526814 | C | T | 0.569087 | 0.00697156 | 0.00118179 |
| Qualifications: College or University degree \|\| id:ukb-a-397 | rs12619354 | C | T | 0.368337 | -0.00760463 | 0.00119209 |
| Qualifications: College or University degree \|\| id:ukb-a-397 | rs12735232 | C | T | 0.157624 | 0.010903 | 0.00156294 |
| Qualifications: College or University degree \|\| id:ukb-a-397 | rs1275202 | C | T | 0.445163 | -0.0070062 | 0.00115333 |
| Qualifications: College or University degree \|\| id:ukb-a-397 | rs12991254 | C | T | 0.401187 | 0.0108549 | 0.00116281 |
| Qualifications: College or University degree \|\| id:ukb-a-397 | rs13033324 | G | A | 0.405879 | 0.00716236 | 0.00116035 |
| Qualifications: College or University degree \|\| id:ukb-a-397 | rs1325604 | A | G | 0.791412 | -0.00874418 | 0.0014063 |
| Qualifications: College or University degree \|\| id:ukb-a-397 | rs1333380 | A | G | 0.259209 | -0.00717863 | 0.00130453 |
| Qualifications: College or University degree \|\| id:ukb-a-397 | rs13397208 | A | C | 0.353417 | 0.00652972 | 0.00119255 |
| Qualifications: College or University degree \|\| id:ukb-a-397 | rs13426183 | A | G | 0.212539 | 0.00841567 | 0.00139566 |
| Qualifications: College or University degree \|\| id:ukb-a-397 | rs1391441 | A | G | 0.688546 | -0.00979383 | 0.00122854 |
| Qualifications: College or University degree \|\| id:ukb-a-397 | rs1455349 | G | A | 0.462483 | -0.00646588 | 0.00115045 |
| Qualifications: College or University degree \|\| id:ukb-a-397 | rs1536057 | T | C | 0.279212 | -0.00721315 | 0.00126895 |
| Qualifications: College or University degree \|\| id:ukb-a-397 | rs1612548 | A | G | 0.231221 | -0.00853195 | 0.00135956 |
| Qualifications: College or University degree \|\| id:ukb-a-397 | rs16828793 | G | T | 0.218696 | -0.00787843 | 0.00138969 |
| Qualifications: College or University degree \|\| id:ukb-a-397 | rs17235639 | G | A | 0.0750733 | -0.0131127 | 0.00230858 |
| Qualifications: College or University degree \|\| id:ukb-a-397 | rs1727302 | A | G | 0.754047 | -0.00906251 | 0.0013232 |
| Qualifications: College or University degree \|\| id:ukb-a-397 | rs17328524 | T | C | 0.555104 | -0.00660279 | 0.00114761 |
| Qualifications: College or University degree \|\| id:ukb-a-397 | rs17563464 | A | C | 0.225462 | -0.00945948 | 0.00138582 |
| Qualifications: College or University degree \|\| id:ukb-a-397 | rs1812587 | T | G | 0.45586 | -0.00725041 | 0.00114651 |
| Qualifications: College or University degree \|\| id:ukb-a-397 | rs1846228 | A | C | 0.513606 | -0.00693455 | 0.00114235 |
| Qualifications: College or University degree \|\| id:ukb-a-397 | rs1914391 | T | C | 0.442647 | -0.00757508 | 0.001147 |
| Qualifications: College or University degree \|\| id:ukb-a-397 | rs1919489 | A | G | 0.779903 | -0.00780238 | 0.00137636 |
| Qualifications: College or University degree \|\| id:ukb-a-397 | rs1998086 | G | A | 0.209584 | -0.00865503 | 0.00140083 |
| Qualifications: College or University degree \|\| id:ukb-a-397 | rs2061245 | A | G | 0.600778 | -0.00667869 | 0.001166 |
| Qualifications: College or University degree \|\| id:ukb-a-397 | rs2083440 | T | C | 0.677509 | 0.00730088 | 0.00121955 |
| Qualifications: College or University degree \|\| id:ukb-a-397 | rs2109661 | T | C | 0.333521 | -0.00665414 | 0.0012092 |
| Qualifications: College or University degree \|\| id:ukb-a-397 | rs2268991 | T | C | 0.0607564 | -0.0132875 | 0.00238824 |
| Qualifications: College or University degree \|\| id:ukb-a-397 | rs2271386 | A | G | 0.150909 | 0.00898778 | 0.00158849 |
| Qualifications: College or University degree \|\| id:ukb-a-397 | rs2341336 | T | C | 0.837563 | -0.00872244 | 0.00156597 |
| Qualifications: College or University degree \|\| id:ukb-a-397 | rs2447535 | G | A | 0.703153 | 0.00744316 | 0.00124782 |
| Qualifications: College or University degree \|\| id:ukb-a-397 | rs2545795 | C | A | 0.564096 | -0.0065099 | 0.00114882 |
| Qualifications: College or University degree \|\| id:ukb-a-397 | rs2661876 | C | A | 0.390397 | 0.00662406 | 0.00116634 |
| Qualifications: College or University degree \|\| id:ukb-a-397 | rs2670012 | T | C | 0.546523 | 0.00687354 | 0.00115126 |
| Qualifications: College or University degree \|\| id:ukb-a-397 | rs2725377 | A | G | 0.558446 | 0.00706858 | 0.00114827 |
| Qualifications: College or University degree \|\| id:ukb-a-397 | rs2735421 | G | T | 0.70162 | -0.00965554 | 0.00126114 |
| Qualifications: College or University degree \|\| id:ukb-a-397 | rs2762546 | T | C | 0.307437 | -0.00846641 | 0.00124029 |
| Qualifications: College or University degree \|\| id:ukb-a-397 | rs2858088 | G | A | 0.618532 | 0.00665401 | 0.00117399 |
| Qualifications: College or University degree \|\| id:ukb-a-397 | rs2860049 | T | G | 0.392728 | -0.00818723 | 0.00117048 |
| Qualifications: College or University degree \|\| id:ukb-a-397 | rs28624826 | T | C | 0.0580266 | 0.0135538 | 0.00243392 |
| Qualifications: College or University degree \|\| id:ukb-a-397 | rs28807201 | C | T | 0.286387 | 0.00703247 | 0.00126195 |
| Qualifications: College or University degree \|\| id:ukb-a-397 | rs2885198 | G | A | 0.466543 | -0.00687794 | 0.00115019 |
| Qualifications: College or University degree \|\| id:ukb-a-397 | rs2995803 | T | C | 0.321682 | 0.00917818 | 0.00121718 |
| Qualifications: College or University degree \|\| id:ukb-a-397 | rs303752 | A | G | 0.410146 | -0.00772102 | 0.00117262 |
| Qualifications: College or University degree \|\| id:ukb-a-397 | rs34811474 | A | G | 0.231875 | 0.00798638 | 0.00134727 |
| Qualifications: College or University degree \|\| id:ukb-a-397 | rs35617898 | T | C | 0.242314 | 0.00846832 | 0.00132661 |
| Qualifications: College or University degree \|\| id:ukb-a-397 | rs35649565 | A | G | 0.245566 | 0.00763705 | 0.00132533 |
| Qualifications: College or University degree \|\| id:ukb-a-397 | rs35811586 | T | C | 0.0569234 | 0.0158895 | 0.00245555 |
| Qualifications: College or University degree \|\| id:ukb-a-397 | rs360947 | A | G | 0.563393 | 0.00729305 | 0.00115405 |
| Qualifications: College or University degree \|\| id:ukb-a-397 | rs38857 | C | T | 0.7094 | 0.00695641 | 0.00126053 |
| Qualifications: College or University degree \|\| id:ukb-a-397 | rs3943093 | T | C | 0.321614 | -0.00850429 | 0.00122037 |
| Qualifications: College or University degree \|\| id:ukb-a-397 | rs4567409 | G | A | 0.498937 | -0.00630507 | 0.0011386 |
| Qualifications: College or University degree \|\| id:ukb-a-397 | rs4691576 | G | T | 0.462948 | 0.00785439 | 0.00114134 |
| Qualifications: College or University degree \|\| id:ukb-a-397 | rs4726070 | A | G | 0.608326 | 0.00671285 | 0.00116608 |
| Qualifications: College or University degree \|\| id:ukb-a-397 | rs4731366 | T | C | 0.740363 | -0.00742824 | 0.00130088 |
| Qualifications: College or University degree \|\| id:ukb-a-397 | rs4810227 | A | G | 0.618663 | 0.00866061 | 0.00117948 |
| Qualifications: College or University degree \|\| id:ukb-a-397 | rs4877151 | T | C | 0.552893 | 0.00634434 | 0.00114484 |
| Qualifications: College or University degree \|\| id:ukb-a-397 | rs4960987 | T | C | 0.529575 | 0.00761382 | 0.00114275 |
| Qualifications: College or University degree \|\| id:ukb-a-397 | rs4983187 | T | G | 0.798555 | -0.0106826 | 0.00142129 |
| Qualifications: College or University degree \|\| id:ukb-a-397 | rs549845 | A | G | 0.700309 | 0.0118559 | 0.00124213 |
| Qualifications: College or University degree \|\| id:ukb-a-397 | rs55677194 | G | A | 0.0743374 | 0.0128252 | 0.00216907 |
| Qualifications: College or University degree \|\| id:ukb-a-397 | rs56099375 | T | C | 0.243169 | 0.00731818 | 0.001332 |
| Qualifications: College or University degree \|\| id:ukb-a-397 | rs56151069 | T | C | 0.152326 | 0.00931609 | 0.00165766 |
| Qualifications: College or University degree \|\| id:ukb-a-397 | rs567203 | C | T | 0.676696 | 0.00860209 | 0.00123583 |
| Qualifications: College or University degree \|\| id:ukb-a-397 | rs5758922 | C | T | 0.488799 | -0.00648556 | 0.00113834 |
| Qualifications: College or University degree \|\| id:ukb-a-397 | rs580241 | A | G | 0.76303 | 0.00943416 | 0.00136885 |
| Qualifications: College or University degree \|\| id:ukb-a-397 | rs59123361 | A | G | 0.102511 | -0.0121115 | 0.00187598 |
| Qualifications: College or University degree \|\| id:ukb-a-397 | rs613872 | T | G | 0.825545 | -0.00934106 | 0.00150113 |
| Qualifications: College or University degree \|\| id:ukb-a-397 | rs61527214 | A | G | 0.405109 | 0.00661663 | 0.00116243 |
| Qualifications: College or University degree \|\| id:ukb-a-397 | rs62036613 | G | A | 0.403744 | -0.00830309 | 0.00115976 |
| Qualifications: College or University degree \|\| id:ukb-a-397 | rs62063281 | G | A | 0.221651 | -0.0096689 | 0.00137003 |
| Qualifications: College or University degree \|\| id:ukb-a-397 | rs62172156 | C | T | 0.374979 | 0.008909 | 0.00117732 |
| Qualifications: College or University degree \|\| id:ukb-a-397 | rs62182993 | A | G | 0.318613 | -0.00909335 | 0.00122256 |
| Qualifications: College or University degree \|\| id:ukb-a-397 | rs62246015 | T | C | 0.31917 | 0.00984658 | 0.00123387 |
| Qualifications: College or University degree \|\| id:ukb-a-397 | rs62507575 | T | C | 0.5335 | 0.00633671 | 0.00114003 |
| Qualifications: College or University degree \|\| id:ukb-a-397 | rs634546 | T | C | 0.35207 | -0.00714539 | 0.00119415 |
| Qualifications: College or University degree \|\| id:ukb-a-397 | rs6429911 | C | T | 0.915582 | 0.0119095 | 0.00204699 |
| Qualifications: College or University degree \|\| id:ukb-a-397 | rs6556982 | G | T | 0.597273 | 0.00642249 | 0.0011622 |
| Qualifications: College or University degree \|\| id:ukb-a-397 | rs6559365 | A | G | 0.496607 | -0.00687125 | 0.00113928 |
| Qualifications: College or University degree \|\| id:ukb-a-397 | rs6673646 | C | T | 0.540565 | -0.00641821 | 0.0011429 |
| Qualifications: College or University degree \|\| id:ukb-a-397 | rs6676960 | T | C | 0.116863 | 0.00983023 | 0.00178138 |
| Qualifications: College or University degree \|\| id:ukb-a-397 | rs6704703 | C | T | 0.927334 | 0.012289 | 0.00219514 |
| Qualifications: College or University degree \|\| id:ukb-a-397 | rs6707062 | C | T | 0.323554 | 0.00689489 | 0.00122052 |
| Qualifications: College or University degree \|\| id:ukb-a-397 | rs6735842 | C | T | 0.561833 | 0.00628153 | 0.00114811 |
| Qualifications: College or University degree \|\| id:ukb-a-397 | rs680767 | G | A | 0.731177 | -0.00718098 | 0.00128482 |
| Qualifications: College or University degree \|\| id:ukb-a-397 | rs6839304 | G | A | 0.313299 | 0.00777684 | 0.00123449 |
| Qualifications: College or University degree \|\| id:ukb-a-397 | rs723968 | C | T | 0.141959 | -0.0100379 | 0.00163909 |
| Qualifications: College or University degree \|\| id:ukb-a-397 | rs7260359 | T | C | 0.481116 | -0.00877871 | 0.00114215 |
| Qualifications: College or University degree \|\| id:ukb-a-397 | rs72801817 | T | C | 0.30155 | 0.00825356 | 0.00123903 |
| Qualifications: College or University degree \|\| id:ukb-a-397 | rs72829857 | G | A | 0.23183 | 0.00968349 | 0.00135231 |
| Qualifications: College or University degree \|\| id:ukb-a-397 | rs7526112 | G | T | 0.362046 | -0.0103173 | 0.00118348 |
| Qualifications: College or University degree \|\| id:ukb-a-397 | rs7561798 | G | A | 0.479847 | -0.00719502 | 0.00113949 |
| Qualifications: College or University degree \|\| id:ukb-a-397 | rs7577926 | T | C | 0.195809 | 0.008432 | 0.00143384 |
| Qualifications: College or University degree \|\| id:ukb-a-397 | rs7582977 | C | T | 0.0925866 | -0.010892 | 0.00197114 |
| Qualifications: College or University degree \|\| id:ukb-a-397 | rs76076331 | T | C | 0.123782 | 0.00992852 | 0.0017403 |
| Qualifications: College or University degree \|\| id:ukb-a-397 | rs7613360 | T | C | 0.397952 | -0.0143747 | 0.00116263 |
| Qualifications: College or University degree \|\| id:ukb-a-397 | rs77451029 | C | T | 0.0556526 | 0.0147144 | 0.00250399 |
| Qualifications: College or University degree \|\| id:ukb-a-397 | rs7757476 | A | G | 0.185024 | 0.00968205 | 0.00148921 |
| Qualifications: College or University degree \|\| id:ukb-a-397 | rs783562 | A | G | 0.368628 | -0.00707834 | 0.00118267 |
| Qualifications: College or University degree \|\| id:ukb-a-397 | rs7862560 | T | C | 0.342072 | 0.00666312 | 0.00121394 |
| Qualifications: College or University degree \|\| id:ukb-a-397 | rs7902 | G | A | 0.443731 | 0.00800297 | 0.00114525 |
| Qualifications: College or University degree \|\| id:ukb-a-397 | rs7921305 | A | G | 0.25517 | 0.00987456 | 0.00130576 |
| Qualifications: College or University degree \|\| id:ukb-a-397 | rs7965989 | T | G | 0.32466 | -0.00770939 | 0.0012196 |
| Qualifications: College or University degree \|\| id:ukb-a-397 | rs7966054 | C | T | 0.567525 | -0.00645699 | 0.00114745 |
| Qualifications: College or University degree \|\| id:ukb-a-397 | rs7988627 | A | G | 0.561403 | -0.00859004 | 0.00115884 |
| Qualifications: College or University degree \|\| id:ukb-a-397 | rs79918827 | A | G | 0.0321932 | -0.0183549 | 0.00322837 |
| Qualifications: College or University degree \|\| id:ukb-a-397 | rs8051058 | C | T | 0.754322 | -0.00793178 | 0.0013264 |
| Qualifications: College or University degree \|\| id:ukb-a-397 | rs8112975 | G | A | 0.271914 | -0.00815616 | 0.00129842 |
| Qualifications: College or University degree \|\| id:ukb-a-397 | rs818414 | G | A | 0.186722 | 0.00834779 | 0.00146015 |
| Qualifications: College or University degree \|\| id:ukb-a-397 | rs860815 | A | G | 0.211541 | -0.00927511 | 0.00139458 |
| Qualifications: College or University degree \|\| id:ukb-a-397 | rs9357004 | C | T | 0.484122 | 0.00971426 | 0.0011375 |
| Qualifications: College or University degree \|\| id:ukb-a-397 | rs9401593 | C | A | 0.485525 | 0.0143613 | 0.00114351 |
| Qualifications: College or University degree \|\| id:ukb-a-397 | rs9556958 | T | C | 0.526665 | -0.00710566 | 0.00114579 |
| Qualifications: College or University degree \|\| id:ukb-a-397 | rs9557427 | T | C | 0.308434 | -0.00739235 | 0.00124377 |
| Qualifications: College or University degree \|\| id:ukb-a-397 | rs9608438 | T | C | 0.268702 | 0.00750188 | 0.00129328 |
| Qualifications: College or University degree \|\| id:ukb-a-397 | rs962490 | A | G | 0.202544 | 0.00834479 | 0.00141679 |
| Qualifications: College or University degree \|\| id:ukb-a-397 | rs9826269 | C | A | 0.417007 | -0.00664674 | 0.00115472 |
| Qualifications: College or University degree \|\| id:ukb-a-397 | rs9891803 | T | C | 0.48637 | -0.0069969 | 0.00114271 |
| Qualifications: College or University degree \|\| id:ukb-a-397 | rs9934839 | G | A | 0.528336 | -0.00705692 | 0.00114181 |
| Qualifications: College or University degree \|\| id:ukb-a-397 | rs9960416 | G | A | 0.469009 | 0.00660204 | 0.00114021 |
| Qualifications: College or University degree \|\| id:ukb-a-397 | rs9964724 | T | C | 0.682676 | 0.0126355 | 0.00122476 |
| Qualifications: College or University degree \|\| id:ukb-a-397 | rs997779 | C | A | 0.208476 | -0.00849249 | 0.00140315 |
| Qualifications: A levels/AS levels or equivalent \|\| id:ukb-a-399 | rs10180461 | C | T | 0.392744 | 0.00718074 | 0.00112034 |
| Qualifications: A levels/AS levels or equivalent \|\| id:ukb-a-399 | rs10189857 | G | A | 0.432196 | -0.00874498 | 0.00110324 |
| Qualifications: A levels/AS levels or equivalent \|\| id:ukb-a-399 | rs10402747 | C | T | 0.480381 | -0.00630085 | 0.00109624 |
| Qualifications: A levels/AS levels or equivalent \|\| id:ukb-a-399 | rs10761254 | C | T | 0.666955 | 0.00640797 | 0.00116232 |
| Qualifications: A levels/AS levels or equivalent \|\| id:ukb-a-399 | rs11600296 | G | A | 0.497125 | -0.00665685 | 0.00109027 |
| Qualifications: A levels/AS levels or equivalent \|\| id:ukb-a-399 | rs11793831 | T | G | 0.416435 | 0.0088729 | 0.00110777 |
| Qualifications: A levels/AS levels or equivalent \|\| id:ukb-a-399 | rs12089622 | A | C | 0.775143 | 0.00950663 | 0.00130715 |
| Qualifications: A levels/AS levels or equivalent \|\| id:ukb-a-399 | rs12511976 | C | T | 0.0820687 | -0.0117473 | 0.00199041 |
| Qualifications: A levels/AS levels or equivalent \|\| id:ukb-a-399 | rs12546203 | G | T | 0.570574 | -0.00667394 | 0.00111297 |
| Qualifications: A levels/AS levels or equivalent \|\| id:ukb-a-399 | rs12614212 | A | G | 0.370837 | -0.00711907 | 0.00113297 |
| Qualifications: A levels/AS levels or equivalent \|\| id:ukb-a-399 | rs1291871 | C | T | 0.512026 | -0.0062873 | 0.00109688 |
| Qualifications: A levels/AS levels or equivalent \|\| id:ukb-a-399 | rs13034294 | A | G | 0.527929 | 0.0067324 | 0.00109453 |
| Qualifications: A levels/AS levels or equivalent \|\| id:ukb-a-399 | rs13240401 | C | T | 0.224465 | -0.00756449 | 0.00130847 |
| Qualifications: A levels/AS levels or equivalent \|\| id:ukb-a-399 | rs1424532 | G | A | 0.327858 | -0.00710864 | 0.00116788 |
| Qualifications: A levels/AS levels or equivalent \|\| id:ukb-a-399 | rs178165 | A | G | 0.6865 | -0.00702357 | 0.00118908 |
| Qualifications: A levels/AS levels or equivalent \|\| id:ukb-a-399 | rs17881016 | G | A | 0.271463 | 0.00710416 | 0.00123691 |
| Qualifications: A levels/AS levels or equivalent \|\| id:ukb-a-399 | rs187600727 | T | C | 0.0307252 | 0.0191709 | 0.00349559 |
| Qualifications: A levels/AS levels or equivalent \|\| id:ukb-a-399 | rs1881975 | G | A | 0.244743 | -0.0070599 | 0.00127622 |
| Qualifications: A levels/AS levels or equivalent \|\| id:ukb-a-399 | rs1931263 | T | G | 0.490185 | -0.00671373 | 0.00109229 |
| Qualifications: A levels/AS levels or equivalent \|\| id:ukb-a-399 | rs1966191 | C | A | 0.336975 | -0.00640565 | 0.00115977 |
| Qualifications: A levels/AS levels or equivalent \|\| id:ukb-a-399 | rs2179152 | C | T | 0.625158 | 0.00880171 | 0.00112617 |
| Qualifications: A levels/AS levels or equivalent \|\| id:ukb-a-399 | rs2325037 | G | A | 0.576716 | 0.00615543 | 0.00110663 |
| Qualifications: A levels/AS levels or equivalent \|\| id:ukb-a-399 | rs2352974 | T | C | 0.491722 | -0.0109587 | 0.00109515 |
| Qualifications: A levels/AS levels or equivalent \|\| id:ukb-a-399 | rs2479552 | A | C | 0.370191 | 0.00670453 | 0.00112924 |
| Qualifications: A levels/AS levels or equivalent \|\| id:ukb-a-399 | rs2568816 | T | G | 0.292986 | 0.00700302 | 0.00119809 |
| Qualifications: A levels/AS levels or equivalent \|\| id:ukb-a-399 | rs28704868 | A | G | 0.380357 | -0.00635712 | 0.00112549 |
| Qualifications: A levels/AS levels or equivalent \|\| id:ukb-a-399 | rs34334167 | A | G | 0.198543 | 0.00777537 | 0.00138668 |
| Qualifications: A levels/AS levels or equivalent \|\| id:ukb-a-399 | rs34954834 | C | T | 0.157368 | 0.0093024 | 0.0014962 |
| Qualifications: A levels/AS levels or equivalent \|\| id:ukb-a-399 | rs3943093 | T | C | 0.321614 | -0.00650046 | 0.00116983 |
| Qualifications: A levels/AS levels or equivalent \|\| id:ukb-a-399 | rs421853 | A | G | 0.708464 | -0.00673544 | 0.00120173 |
| Qualifications: A levels/AS levels or equivalent \|\| id:ukb-a-399 | rs4457002 | T | C | 0.523304 | 0.0079383 | 0.00109441 |
| Qualifications: A levels/AS levels or equivalent \|\| id:ukb-a-399 | rs4675248 | G | A | 0.605479 | 0.00654121 | 0.00111677 |
| Qualifications: A levels/AS levels or equivalent \|\| id:ukb-a-399 | rs4886901 | A | G | 0.441526 | 0.00609652 | 0.00109807 |
| Qualifications: A levels/AS levels or equivalent \|\| id:ukb-a-399 | rs4981692 | A | G | 0.808554 | -0.00787051 | 0.00138632 |
| Qualifications: A levels/AS levels or equivalent \|\| id:ukb-a-399 | rs518709 | T | C | 0.599204 | -0.00697809 | 0.00111214 |
| Qualifications: A levels/AS levels or equivalent \|\| id:ukb-a-399 | rs55853834 | C | A | 0.159671 | -0.00929624 | 0.00149673 |
| Qualifications: A levels/AS levels or equivalent \|\| id:ukb-a-399 | rs56219078 | C | T | 0.461022 | 0.00654082 | 0.00109457 |
| Qualifications: A levels/AS levels or equivalent \|\| id:ukb-a-399 | rs578096 | C | T | 0.839079 | -0.00835211 | 0.00148252 |
| Qualifications: A levels/AS levels or equivalent \|\| id:ukb-a-399 | rs58503743 | A | G | 0.309135 | 0.00646939 | 0.00118263 |
| Qualifications: A levels/AS levels or equivalent \|\| id:ukb-a-399 | rs604781 | T | C | 0.198468 | -0.00759082 | 0.00137602 |
| Qualifications: A levels/AS levels or equivalent \|\| id:ukb-a-399 | rs62139974 | A | G | 0.207966 | -0.00795572 | 0.0013474 |
| Qualifications: A levels/AS levels or equivalent \|\| id:ukb-a-399 | rs62183028 | T | G | 0.312781 | -0.00668001 | 0.00117993 |
| Qualifications: A levels/AS levels or equivalent \|\| id:ukb-a-399 | rs6714628 | T | G | 0.619573 | -0.00755929 | 0.00112307 |
| Qualifications: A levels/AS levels or equivalent \|\| id:ukb-a-399 | rs67589009 | G | A | 0.476984 | -0.00685544 | 0.00109818 |
| Qualifications: A levels/AS levels or equivalent \|\| id:ukb-a-399 | rs7203315 | T | G | 0.127202 | -0.0105704 | 0.0016444 |
| Qualifications: A levels/AS levels or equivalent \|\| id:ukb-a-399 | rs7242967 | G | T | 0.468848 | -0.00689586 | 0.00109528 |
| Qualifications: A levels/AS levels or equivalent \|\| id:ukb-a-399 | rs72829857 | G | A | 0.23183 | 0.00777682 | 0.0012963 |
| Qualifications: A levels/AS levels or equivalent \|\| id:ukb-a-399 | rs7325960 | T | G | 0.733549 | 0.00865723 | 0.00123749 |
| Qualifications: A levels/AS levels or equivalent \|\| id:ukb-a-399 | rs74619517 | A | G | 0.130804 | -0.00896409 | 0.00162003 |
| Qualifications: A levels/AS levels or equivalent \|\| id:ukb-a-399 | rs7896518 | G | A | 0.421706 | 0.00669039 | 0.00111516 |
| Age completed full time education \|\| id:ukb-b-6134 | rs10189857 | G | A | 0.435409 | -0.0124054 | 0.00208474 |
| Age completed full time education \|\| id:ukb-b-6134 | rs10200379 | A | C | 0.519841 | 0.0139597 | 0.00207137 |
| Age completed full time education \|\| id:ukb-b-6134 | rs10760199 | T | G | 0.551851 | 0.0115963 | 0.00208033 |
| Age completed full time education \|\| id:ukb-b-6134 | rs10953765 | A | G | 0.550745 | -0.0130933 | 0.002104 |
| Age completed full time education \|\| id:ukb-b-6134 | rs13064576 | T | C | 0.294739 | 0.0172696 | 0.00226489 |
| Age completed full time education \|\| id:ukb-b-6134 | rs13238996 | G | A | 0.242877 | -0.0133142 | 0.00240789 |
| Age completed full time education \|\| id:ukb-b-6134 | rs13274119 | A | C | 0.13803 | 0.0198214 | 0.00301673 |
| Age completed full time education \|\| id:ukb-b-6134 | rs13394374 | T | C | 0.030466 | 0.0335998 | 0.00611413 |
| Age completed full time education \|\| id:ukb-b-6134 | rs1462163 | T | C | 0.40086 | -0.0127222 | 0.00211969 |
| Age completed full time education \|\| id:ukb-b-6134 | rs1557341 | C | A | 0.663393 | 0.0129858 | 0.00220266 |
| Age completed full time education \|\| id:ukb-b-6134 | rs17563464 | A | C | 0.225571 | -0.0148391 | 0.00251248 |
| Age completed full time education \|\| id:ukb-b-6134 | rs178217 | C | T | 0.750378 | -0.0143669 | 0.00239746 |
| Age completed full time education \|\| id:ukb-b-6134 | rs2588962 | A | G | 0.458738 | 0.0150825 | 0.00207972 |
| Age completed full time education \|\| id:ukb-b-6134 | rs2709814 | C | T | 0.568651 | -0.0114761 | 0.0020926 |
| Age completed full time education \|\| id:ukb-b-6134 | rs34945223 | G | A | 0.278543 | -0.0136288 | 0.00230526 |
| Age completed full time education \|\| id:ukb-b-6134 | rs4557720 | G | A | 0.378226 | 0.0119687 | 0.00213393 |
| Age completed full time education \|\| id:ukb-b-6134 | rs4674403 | G | T | 0.718268 | -0.0127249 | 0.0023324 |
| Age completed full time education \|\| id:ukb-b-6134 | rs4731951 | T | G | 0.80058 | 0.0153215 | 0.00259236 |
| Age completed full time education \|\| id:ukb-b-6134 | rs57513571 | T | C | 0.200955 | -0.0153935 | 0.00258666 |
| Age completed full time education \|\| id:ukb-b-6134 | rs62039529 | A | C | 0.837847 | 0.0165739 | 0.00284063 |
| Age completed full time education \|\| id:ukb-b-6134 | rs6449503 | A | G | 0.491513 | 0.0123947 | 0.00208193 |
| Age completed full time education \|\| id:ukb-b-6134 | rs6508344 | A | G | 0.571312 | -0.0115957 | 0.00208724 |
| Age completed full time education \|\| id:ukb-b-6134 | rs6679399 | C | A | 0.025961 | 0.0426608 | 0.00677016 |
| Age completed full time education \|\| id:ukb-b-6134 | rs6729586 | A | G | 0.599259 | -0.0117953 | 0.00211341 |
| Age completed full time education \|\| id:ukb-b-6134 | rs6754311 | C | T | 0.257831 | 0.0150378 | 0.002367 |
| Age completed full time education \|\| id:ukb-b-6134 | rs68191270 | C | A | 0.778588 | 0.0166103 | 0.00248918 |
| Age completed full time education \|\| id:ukb-b-6134 | rs6931604 | T | C | 0.597011 | 0.0136265 | 0.0021131 |
| Age completed full time education \|\| id:ukb-b-6134 | rs7110786 | T | C | 0.418918 | 0.0127141 | 0.00209121 |
| Age completed full time education \|\| id:ukb-b-6134 | rs7768758 | C | T | 0.080585 | -0.0227349 | 0.00379371 |
| Age completed full time education \|\| id:ukb-b-6134 | rs7896518 | G | A | 0.425249 | 0.0145245 | 0.00211298 |
| Age completed full time education \|\| id:ukb-b-6134 | rs7975763 | T | C | 0.203454 | 0.0164068 | 0.00257604 |
| Age completed full time education \|\| id:ukb-b-6134 | rs9536961 | G | A | 0.346028 | 0.0139369 | 0.0022011 |
| Age completed full time education \|\| id:ukb-b-6134 | rs9655780 | A | G | 0.832553 | -0.0174579 | 0.00279646 |
| Age completed full time education \|\| id:ukb-b-6134 | rs9866630 | A | G | 0.504663 | -0.0126597 | 0.0020719 |
| Sleeplessness / insomnia \|\| id:ukb-a-13 | rs10087341 | C | T | 0.158749 | 0.0147121 | 0.00239204 |
| Sleeplessness / insomnia \|\| id:ukb-a-13 | rs10156602 | G | A | 0.363532 | -0.0101865 | 0.00182347 |
| Sleeplessness / insomnia \|\| id:ukb-a-13 | rs11152363 | A | G | 0.184703 | 0.0143109 | 0.00226276 |
| Sleeplessness / insomnia \|\| id:ukb-a-13 | rs113851554 | T | G | 0.0566594 | 0.0477769 | 0.00389018 |
| Sleeplessness / insomnia \|\| id:ukb-a-13 | rs11635495 | C | T | 0.513921 | 0.00973115 | 0.00174233 |
| Sleeplessness / insomnia \|\| id:ukb-a-13 | rs11804386 | A | G | 0.333176 | 0.0101714 | 0.00184599 |
| Sleeplessness / insomnia \|\| id:ukb-a-13 | rs13186678 | T | C | 0.307241 | 0.0110482 | 0.00188849 |
| Sleeplessness / insomnia \|\| id:ukb-a-13 | rs1547630 | A | G | 0.651732 | 0.0104415 | 0.00183342 |
| Sleeplessness / insomnia \|\| id:ukb-a-13 | rs2132083 | C | T | 0.660134 | -0.0101194 | 0.00184353 |
| Sleeplessness / insomnia \|\| id:ukb-a-13 | rs224071 | A | G | 0.550307 | 0.00972618 | 0.00174868 |
| Sleeplessness / insomnia \|\| id:ukb-a-13 | rs2863957 | A | C | 0.219489 | -0.0129917 | 0.00210431 |
| Sleeplessness / insomnia \|\| id:ukb-a-13 | rs2956278 | G | A | 0.214265 | 0.0119098 | 0.00212317 |
| Sleeplessness / insomnia \|\| id:ukb-a-13 | rs324017 | C | A | 0.70472 | -0.011717 | 0.0019116 |
| Sleeplessness / insomnia \|\| id:ukb-a-13 | rs3808937 | T | C | 0.207802 | -0.0143079 | 0.00214558 |
| Sleeplessness / insomnia \|\| id:ukb-a-13 | rs3817576 | G | A | 0.525328 | -0.01038 | 0.00174667 |
| Sleeplessness / insomnia \|\| id:ukb-a-13 | rs4688760 | T | C | 0.691885 | 0.011506 | 0.00188616 |
| Sleeplessness / insomnia \|\| id:ukb-a-13 | rs4886140 | G | A | 0.667148 | 0.0110768 | 0.00186185 |
| Sleeplessness / insomnia \|\| id:ukb-a-13 | rs4943439 | T | C | 0.386641 | 0.0104509 | 0.00178466 |
| Sleeplessness / insomnia \|\| id:ukb-a-13 | rs6690017 | G | T | 0.408391 | -0.00985431 | 0.00177148 |
| Sleeplessness / insomnia \|\| id:ukb-a-13 | rs6744461 | C | A | 0.680005 | 0.0108831 | 0.00188291 |
| Sleeplessness / insomnia \|\| id:ukb-a-13 | rs71373536 | A | G | 0.253453 | 0.0134498 | 0.00200376 |
| Sleeplessness / insomnia \|\| id:ukb-a-13 | rs7572387 | C | A | 0.429282 | 0.0109301 | 0.00176715 |
| Sleeplessness / insomnia \|\| id:ukb-a-13 | rs9815484 | G | A | 0.817741 | 0.0131678 | 0.00225968 |
| Sleeplessness / insomnia \|\| id:ukb-a-13 | rs9878792 | T | G | 0.0374927 | -0.0266984 | 0.00465798 |
| Daytime nap \|\| id:ebi-a-GCST011494 | rs1001817 | T | C | 0.492788 | -0.00775426 | 0.00121418 |
| Daytime nap \|\| id:ebi-a-GCST011494 | rs10149986 | G | T | 0.185198 | 0.0109384 | 0.00156608 |
| Daytime nap \|\| id:ebi-a-GCST011494 | rs10840017 | G | A | 0.232631 | -0.00894142 | 0.00148101 |
| Daytime nap \|\| id:ebi-a-GCST011494 | rs10875606 | A | C | 0.684233 | -0.00737707 | 0.0013077 |
| Daytime nap \|\| id:ebi-a-GCST011494 | rs10875622 | A | G | 0.575365 | 0.0104621 | 0.0012291 |
| Daytime nap \|\| id:ebi-a-GCST011494 | rs11071755 | A | G | 0.425078 | -0.00711401 | 0.00122819 |
| Daytime nap \|\| id:ebi-a-GCST011494 | rs11224896 | C | T | 0.110253 | -0.0111605 | 0.00193872 |
| Daytime nap \|\| id:ebi-a-GCST011494 | rs11258652 | A | C | 0.236815 | -0.0104004 | 0.00142918 |
| Daytime nap \|\| id:ebi-a-GCST011494 | rs11615756 | T | C | 0.404611 | 0.0182673 | 0.00123883 |
| Daytime nap \|\| id:ebi-a-GCST011494 | rs11682175 | C | T | 0.472864 | 0.0069583 | 0.0012166 |
| Daytime nap \|\| id:ebi-a-GCST011494 | rs11860072 | T | C | 0.447209 | -0.00897773 | 0.00123952 |
| Daytime nap \|\| id:ebi-a-GCST011494 | rs12042846 | C | T | 0.178874 | 0.00901497 | 0.00159014 |
| Daytime nap \|\| id:ebi-a-GCST011494 | rs12140153 | T | G | 0.095397 | -0.0247147 | 0.00211564 |
| Daytime nap \|\| id:ebi-a-GCST011494 | rs12346996 | C | T | 0.728071 | -0.00796106 | 0.00136459 |
| Daytime nap \|\| id:ebi-a-GCST011494 | rs12451365 | C | T | 0.204638 | 0.010628 | 0.00150388 |
| Daytime nap \|\| id:ebi-a-GCST011494 | rs12657723 | T | C | 0.321683 | 0.00818319 | 0.00129849 |
| Daytime nap \|\| id:ebi-a-GCST011494 | rs12992648 | G | A | 0.282932 | -0.00765323 | 0.00134659 |
| Daytime nap \|\| id:ebi-a-GCST011494 | rs13033444 | G | A | 0.282805 | 0.00974179 | 0.00135018 |
| Daytime nap \|\| id:ebi-a-GCST011494 | rs13150944 | G | A | 0.689386 | 0.00860076 | 0.0013218 |
| Daytime nap \|\| id:ebi-a-GCST011494 | rs13263535 | T | G | 0.468536 | -0.00687135 | 0.00121641 |
| Daytime nap \|\| id:ebi-a-GCST011494 | rs13284688 | C | T | 0.206572 | 0.0150717 | 0.00149894 |
| Daytime nap \|\| id:ebi-a-GCST011494 | rs13330072 | G | A | 0.286953 | 0.00791082 | 0.00134123 |
| Daytime nap \|\| id:ebi-a-GCST011494 | rs1546977 | G | A | 0.439655 | 0.00827718 | 0.00122321 |
| Daytime nap \|\| id:ebi-a-GCST011494 | rs1601440 | T | C | 0.721378 | -0.00912653 | 0.00135575 |
| Daytime nap \|\| id:ebi-a-GCST011494 | rs17158413 | A | G | 0.237279 | 0.00925014 | 0.00142955 |
| Daytime nap \|\| id:ebi-a-GCST011494 | rs17265513 | C | T | 0.199401 | 0.00913188 | 0.00151965 |
| Daytime nap \|\| id:ebi-a-GCST011494 | rs174541 | C | T | 0.360451 | 0.00988214 | 0.00126293 |
| Daytime nap \|\| id:ebi-a-GCST011494 | rs17502738 | C | T | 0.195889 | -0.00870459 | 0.00153004 |
| Daytime nap \|\| id:ebi-a-GCST011494 | rs1883048 | C | T | 0.523561 | 0.00787143 | 0.00122313 |
| Daytime nap \|\| id:ebi-a-GCST011494 | rs2033103 | T | C | 0.452115 | 0.00725728 | 0.00122022 |
| Daytime nap \|\| id:ebi-a-GCST011494 | rs2059639 | T | C | 0.520557 | -0.0066823 | 0.0012172 |
| Daytime nap \|\| id:ebi-a-GCST011494 | rs2099810 | G | A | 0.49693 | -0.00769192 | 0.00121447 |
| Daytime nap \|\| id:ebi-a-GCST011494 | rs2143792 | A | G | 0.432469 | -0.00713406 | 0.00123401 |
| Daytime nap \|\| id:ebi-a-GCST011494 | rs224111 | A | G | 0.387694 | -0.00798411 | 0.00124912 |
| Daytime nap \|\| id:ebi-a-GCST011494 | rs2250377 | G | A | 0.661117 | -0.0131561 | 0.00127975 |
| Daytime nap \|\| id:ebi-a-GCST011494 | rs2370926 | C | T | 0.366474 | -0.00813213 | 0.00126359 |
| Daytime nap \|\| id:ebi-a-GCST011494 | rs2653349 | G | A | 0.785223 | -0.0165942 | 0.00147827 |
| Daytime nap \|\| id:ebi-a-GCST011494 | rs2699869 | C | A | 0.545872 | -0.00678084 | 0.00121774 |
| Daytime nap \|\| id:ebi-a-GCST011494 | rs271057 | T | C | 0.751573 | -0.00866783 | 0.00140414 |
| Daytime nap \|\| id:ebi-a-GCST011494 | rs2769916 | A | G | 0.688617 | 0.00875328 | 0.00131344 |
| Daytime nap \|\| id:ebi-a-GCST011494 | rs2943023 | T | C | 0.419803 | -0.00710422 | 0.00123055 |
| Daytime nap \|\| id:ebi-a-GCST011494 | rs295278 | A | G | 0.394519 | -0.00787804 | 0.00124238 |
| Daytime nap \|\| id:ebi-a-GCST011494 | rs34262487 | A | C | 0.072003 | -0.0145107 | 0.00235463 |
| Daytime nap \|\| id:ebi-a-GCST011494 | rs35011311 | T | G | 0.265581 | -0.009082 | 0.00138249 |
| Daytime nap \|\| id:ebi-a-GCST011494 | rs35039375 | G | A | 0.091832 | 0.0136431 | 0.00210973 |
| Daytime nap \|\| id:ebi-a-GCST011494 | rs350785 | C | T | 0.884524 | -0.0126479 | 0.00190975 |
| Daytime nap \|\| id:ebi-a-GCST011494 | rs351776 | C | A | 0.549518 | 0.00755367 | 0.00122072 |
| Daytime nap \|\| id:ebi-a-GCST011494 | rs35851551 | G | A | 0.101504 | -0.011194 | 0.00202654 |
| Daytime nap \|\| id:ebi-a-GCST011494 | rs378421 | A | G | 0.41729 | 0.00697888 | 0.0012384 |
| Daytime nap \|\| id:ebi-a-GCST011494 | rs3810484 | G | A | 0.443696 | -0.00684147 | 0.001222 |
| Daytime nap \|\| id:ebi-a-GCST011494 | rs385199 | C | A | 0.227862 | -0.0209131 | 0.0014497 |
| Daytime nap \|\| id:ebi-a-GCST011494 | rs3935190 | A | G | 0.536252 | 0.00801432 | 0.00122354 |
| Daytime nap \|\| id:ebi-a-GCST011494 | rs4511908 | G | T | 0.580907 | 0.00726347 | 0.00128337 |
| Daytime nap \|\| id:ebi-a-GCST011494 | rs4604518 | A | G | 0.453526 | -0.00691205 | 0.00122091 |
| Daytime nap \|\| id:ebi-a-GCST011494 | rs467897 | A | G | 0.677609 | -0.00955698 | 0.00130107 |
| Daytime nap \|\| id:ebi-a-GCST011494 | rs60222088 | A | C | 0.147267 | -0.0112358 | 0.00172293 |
| Daytime nap \|\| id:ebi-a-GCST011494 | rs60920123 | A | G | 0.433192 | -0.00763778 | 0.00122697 |
| Daytime nap \|\| id:ebi-a-GCST011494 | rs614987 | C | A | 0.614205 | 0.0110217 | 0.00124925 |
| Daytime nap \|\| id:ebi-a-GCST011494 | rs62189006 | G | A | 0.092801 | -0.0120943 | 0.0020982 |
| Daytime nap \|\| id:ebi-a-GCST011494 | rs6452787 | G | A | 0.463847 | -0.00772931 | 0.0012169 |
| Daytime nap \|\| id:ebi-a-GCST011494 | rs6665690 | C | A | 0.170043 | -0.00923907 | 0.00161461 |
| Daytime nap \|\| id:ebi-a-GCST011494 | rs73817091 | T | C | 0.042467 | 0.0165127 | 0.00302224 |
| Daytime nap \|\| id:ebi-a-GCST011494 | rs7422655 | T | C | 0.736885 | -0.00783348 | 0.00137827 |
| Daytime nap \|\| id:ebi-a-GCST011494 | rs7423968 | G | A | 0.428827 | -0.00767586 | 0.00123216 |
| Daytime nap \|\| id:ebi-a-GCST011494 | rs75022160 | T | C | 0.137254 | -0.00988485 | 0.00176929 |
| Daytime nap \|\| id:ebi-a-GCST011494 | rs76257331 | A | G | 0.198266 | 0.00867666 | 0.0015218 |
| Daytime nap \|\| id:ebi-a-GCST011494 | rs76824303 | C | A | 0.099416 | -0.0117977 | 0.00207749 |
| Daytime nap \|\| id:ebi-a-GCST011494 | rs77154532 | G | A | 0.358326 | -0.00769324 | 0.0012705 |
| Daytime nap \|\| id:ebi-a-GCST011494 | rs7814873 | T | C | 0.617006 | -0.00720972 | 0.00126385 |
| Daytime nap \|\| id:ebi-a-GCST011494 | rs785145 | G | T | 0.431455 | 0.0069889 | 0.00122527 |
| Daytime nap \|\| id:ebi-a-GCST011494 | rs80163246 | C | T | 0.117455 | 0.0119129 | 0.00188438 |
| Daytime nap \|\| id:ebi-a-GCST011494 | rs9287862 | T | C | 0.913226 | -0.0122526 | 0.00215162 |
| Daytime nap \|\| id:ebi-a-GCST011494 | rs9309116 | T | C | 0.652831 | 0.00737914 | 0.00127277 |
| Daytime nap \|\| id:ebi-a-GCST011494 | rs936944 | A | G | 0.837821 | -0.00913528 | 0.00164374 |
| Daytime nap \|\| id:ebi-a-GCST011494 | rs9460110 | C | T | 0.369782 | 0.00735568 | 0.00125751 |
| Daytime nap \|\| id:ebi-a-GCST011494 | rs962247 | A | G | 0.476801 | -0.00796355 | 0.00122493 |
| Daytime nap \|\| id:ebi-a-GCST011494 | rs971415 | G | A | 0.122378 | -0.0110652 | 0.00185108 |
| Daytime nap \|\| id:ebi-a-GCST011494 | rs9939355 | T | C | 0.557671 | -0.00726404 | 0.00122438 |
| Daytime nap \|\| id:ebi-a-GCST011494 | rs9965170 | A | G | 0.423393 | -0.0136374 | 0.00122874 |
| Nap during day \|\| id:ukb-b-4616 | rs1001817 | T | C | 0.494347 | -0.00776666 | 0.00122507 |
| Nap during day \|\| id:ukb-b-4616 | rs1011024 | G | A | 0.154712 | -0.0102274 | 0.00169762 |
| Nap during day \|\| id:ukb-b-4616 | rs10150432 | G | A | 0.184721 | 0.0104521 | 0.00157996 |
| Nap during day \|\| id:ukb-b-4616 | rs10757347 | G | A | 0.222815 | 0.00810359 | 0.00147647 |
| Nap during day \|\| id:ukb-b-4616 | rs10764260 | A | G | 0.363097 | -0.00733254 | 0.00128128 |
| Nap during day \|\| id:ukb-b-4616 | rs10840017 | G | A | 0.233223 | -0.00866558 | 0.00149279 |
| Nap during day \|\| id:ukb-b-4616 | rs10868046 | A | G | 0.603704 | 0.00727374 | 0.00126066 |
| Nap during day \|\| id:ukb-b-4616 | rs10875622 | A | G | 0.575874 | 0.0102674 | 0.00124068 |
| Nap during day \|\| id:ukb-b-4616 | rs11071755 | A | G | 0.424644 | -0.00696004 | 0.00123874 |
| Nap during day \|\| id:ukb-b-4616 | rs11121194 | T | C | 0.633703 | 0.00706318 | 0.00126583 |
| Nap during day \|\| id:ukb-b-4616 | rs11125776 | G | T | 0.144182 | -0.0120412 | 0.00174757 |
| Nap during day \|\| id:ukb-b-4616 | rs11224896 | C | T | 0.110091 | -0.0111859 | 0.00195596 |
| Nap during day \|\| id:ukb-b-4616 | rs11252681 | A | G | 0.045481 | 0.016111 | 0.00293663 |
| Nap during day \|\| id:ukb-b-4616 | rs11258652 | A | C | 0.235891 | -0.0100132 | 0.0014441 |
| Nap during day \|\| id:ukb-b-4616 | rs113886333 | T | C | 0.036115 | 0.0182674 | 0.00329762 |
| Nap during day \|\| id:ukb-b-4616 | rs11615756 | T | C | 0.403516 | 0.0182385 | 0.00125078 |
| Nap during day \|\| id:ukb-b-4616 | rs12042846 | C | T | 0.178571 | 0.00905398 | 0.00160707 |
| Nap during day \|\| id:ukb-b-4616 | rs12140153 | T | G | 0.09424 | -0.0242712 | 0.0021468 |
| Nap during day \|\| id:ukb-b-4616 | rs12346996 | C | T | 0.728608 | -0.00832202 | 0.00137794 |
| Nap during day \|\| id:ukb-b-4616 | rs12451365 | C | T | 0.204133 | 0.0108452 | 0.00151874 |
| Nap during day \|\| id:ukb-b-4616 | rs12615434 | T | C | 0.11309 | 0.0116344 | 0.00193313 |
| Nap during day \|\| id:ukb-b-4616 | rs12657723 | T | C | 0.320683 | 0.0081807 | 0.00131152 |
| Nap during day \|\| id:ukb-b-4616 | rs12992648 | G | A | 0.281533 | -0.00762551 | 0.00136135 |
| Nap during day \|\| id:ukb-b-4616 | rs13033444 | G | A | 0.281934 | 0.0094407 | 0.00136451 |
| Nap during day \|\| id:ukb-b-4616 | rs13266972 | G | A | 0.698405 | -0.00729523 | 0.00133527 |
| Nap during day \|\| id:ukb-b-4616 | rs13284688 | C | T | 0.207055 | 0.0144228 | 0.00151104 |
| Nap during day \|\| id:ukb-b-4616 | rs1479116 | A | G | 0.351118 | 0.00796453 | 0.00129506 |
| Nap during day \|\| id:ukb-b-4616 | rs17158413 | A | G | 0.237392 | 0.00855728 | 0.00144202 |
| Nap during day \|\| id:ukb-b-4616 | rs17265513 | C | T | 0.198242 | 0.00888057 | 0.00153558 |
| Nap during day \|\| id:ukb-b-4616 | rs174541 | C | T | 0.35911 | 0.0101856 | 0.00127505 |
| Nap during day \|\| id:ukb-b-4616 | rs1883048 | C | T | 0.524834 | 0.0078482 | 0.00123337 |
| Nap during day \|\| id:ukb-b-4616 | rs2033103 | T | C | 0.450439 | 0.00753532 | 0.00123118 |
| Nap during day \|\| id:ukb-b-4616 | rs2099810 | G | A | 0.496352 | -0.00780818 | 0.0012254 |
| Nap during day \|\| id:ukb-b-4616 | rs224111 | A | G | 0.388797 | -0.00784245 | 0.0012587 |
| Nap during day \|\| id:ukb-b-4616 | rs2284016 | C | T | 0.399515 | 0.0070943 | 0.0012508 |
| Nap during day \|\| id:ukb-b-4616 | rs2370926 | C | T | 0.367305 | -0.00828326 | 0.00127397 |
| Nap during day \|\| id:ukb-b-4616 | rs2390669 | C | A | 0.128947 | -0.0112781 | 0.00183747 |
| Nap during day \|\| id:ukb-b-4616 | rs2653349 | G | A | 0.787137 | -0.0163076 | 0.0014949 |
| Nap during day \|\| id:ukb-b-4616 | rs2769916 | A | G | 0.688798 | 0.00877372 | 0.00132557 |
| Nap during day \|\| id:ukb-b-4616 | rs2786547 | T | C | 0.177277 | -0.0109884 | 0.00160158 |
| Nap during day \|\| id:ukb-b-4616 | rs34262487 | A | C | 0.072621 | -0.0146376 | 0.00236551 |
| Nap during day \|\| id:ukb-b-4616 | rs35011311 | T | G | 0.264996 | -0.00946899 | 0.00139578 |
| Nap during day \|\| id:ukb-b-4616 | rs351776 | C | A | 0.548043 | 0.00762604 | 0.00123034 |
| Nap during day \|\| id:ukb-b-4616 | rs35851551 | G | A | 0.101129 | -0.0119501 | 0.00204914 |
| Nap during day \|\| id:ukb-b-4616 | rs3810484 | G | A | 0.443064 | -0.00710613 | 0.00123272 |
| Nap during day \|\| id:ukb-b-4616 | rs3935190 | A | G | 0.536792 | 0.00826969 | 0.00123459 |
| Nap during day \|\| id:ukb-b-4616 | rs40005 | A | G | 0.76889 | 0.00807247 | 0.00145429 |
| Nap during day \|\| id:ukb-b-4616 | rs4402351 | G | A | 0.148069 | -0.0117649 | 0.00173327 |
| Nap during day \|\| id:ukb-b-4616 | rs4587762 | A | G | 0.604757 | -0.00694078 | 0.00125608 |
| Nap during day \|\| id:ukb-b-4616 | rs467897 | A | G | 0.68009 | -0.00930896 | 0.00131516 |
| Nap during day \|\| id:ukb-b-4616 | rs4692709 | T | C | 0.546706 | -0.00700067 | 0.00123617 |
| Nap during day \|\| id:ukb-b-4616 | rs4856536 | A | G | 0.728768 | -0.00859681 | 0.00138106 |
| Nap during day \|\| id:ukb-b-4616 | rs60920123 | A | G | 0.432894 | -0.00744881 | 0.00123803 |
| Nap during day \|\| id:ukb-b-4616 | rs614987 | C | A | 0.613566 | 0.0107556 | 0.00125938 |
| Nap during day \|\| id:ukb-b-4616 | rs62425620 | T | C | 0.369978 | 0.00775191 | 0.00127613 |
| Nap during day \|\| id:ukb-b-4616 | rs62560863 | T | C | 0.100702 | 0.0114884 | 0.00203914 |
| Nap during day \|\| id:ukb-b-4616 | rs6452787 | G | A | 0.466869 | -0.0072452 | 0.00122693 |
| Nap during day \|\| id:ukb-b-4616 | rs6483215 | G | A | 0.764113 | -0.00841794 | 0.00144207 |
| Nap during day \|\| id:ukb-b-4616 | rs6919087 | G | T | 0.311989 | -0.0103817 | 0.00132307 |
| Nap during day \|\| id:ukb-b-4616 | rs6942927 | A | G | 0.12313 | 0.0146516 | 0.00217116 |
| Nap during day \|\| id:ukb-b-4616 | rs7038206 | G | A | 0.608433 | 0.00738228 | 0.00125472 |
| Nap during day \|\| id:ukb-b-4616 | rs7191614 | G | A | 0.290789 | 0.00769805 | 0.00135296 |
| Nap during day \|\| id:ukb-b-4616 | rs7198121 | C | T | 0.538397 | -0.00681975 | 0.00123097 |
| Nap during day \|\| id:ukb-b-4616 | rs72781017 | G | A | 0.402575 | -0.00756808 | 0.00124775 |
| Nap during day \|\| id:ukb-b-4616 | rs7422655 | T | C | 0.736305 | -0.00805295 | 0.00139094 |
| Nap during day \|\| id:ukb-b-4616 | rs75022160 | T | C | 0.1366 | -0.00976296 | 0.00179002 |
| Nap during day \|\| id:ukb-b-4616 | rs75411336 | T | C | 0.054402 | -0.015756 | 0.0027004 |
| Nap during day \|\| id:ukb-b-4616 | rs7555990 | T | C | 0.13587 | -0.010372 | 0.00178981 |
| Nap during day \|\| id:ukb-b-4616 | rs77154532 | G | A | 0.359504 | -0.00724236 | 0.00128118 |
| Nap during day \|\| id:ukb-b-4616 | rs7752899 | T | C | 0.442806 | 0.00847983 | 0.00123126 |
| Nap during day \|\| id:ukb-b-4616 | rs7814873 | T | C | 0.616133 | -0.00704488 | 0.00127461 |
| Nap during day \|\| id:ukb-b-4616 | rs785145 | G | T | 0.431525 | 0.00706492 | 0.00123603 |
| Nap during day \|\| id:ukb-b-4616 | rs8050478 | A | G | 0.500601 | -0.00763479 | 0.0012248 |
| Nap during day \|\| id:ukb-b-4616 | rs903678 | A | G | 0.336999 | 0.013653 | 0.00129298 |
| Nap during day \|\| id:ukb-b-4616 | rs962247 | A | G | 0.475853 | -0.00817957 | 0.00123599 |
| Nap during day \|\| id:ukb-b-4616 | rs971415 | G | A | 0.122716 | -0.0108343 | 0.00186482 |
| Nap during day \|\| id:ukb-b-4616 | rs9965170 | A | G | 0.423866 | -0.014193 | 0.00123929 |
| Feeling guilty \|\| id:ebi-a-GCST006945 | rs10119773 | G | A | 0.534002 | 0.0157348 | 0.00237794 |
| Feeling guilty \|\| id:ebi-a-GCST006945 | rs12420205 | T | C | 0.677505 | 0.0149574 | 0.00250207 |
| Feeling guilty \|\| id:ebi-a-GCST006945 | rs12528131 | G | A | 0.4826 | 0.0149215 | 0.00232422 |
| Feeling guilty \|\| id:ebi-a-GCST006945 | rs1557339 | A | C | 0.738067 | -0.0166649 | 0.00265915 |
| Feeling guilty \|\| id:ebi-a-GCST006945 | rs2109648 | A | G | 0.63965 | 0.0141103 | 0.00241614 |
| Feeling guilty \|\| id:ebi-a-GCST006945 | rs34657012 | A | C | 0.279037 | 0.0154767 | 0.00258463 |
| Feeling guilty \|\| id:ebi-a-GCST006945 | rs55769038 | A | G | 0.578002 | -0.0160833 | 0.00235068 |
| Feeling guilty \|\| id:ebi-a-GCST006945 | rs681875 | A | C | 0.206279 | 0.0170061 | 0.00287168 |
| Feeling guilty \|\| id:ebi-a-GCST006945 | rs77804065 | T | C | 0.217158 | 0.0175056 | 0.00283353 |
| Worry too long after an embarrassing experience \|\| id:ebi-a-GCST006946 | rs10750866 | G | A | 0.272951 | 0.0179002 | 0.00263976 |
| Worry too long after an embarrassing experience \|\| id:ebi-a-GCST006946 | rs1983614 | T | C | 0.484257 | 0.0128425 | 0.00233458 |
| Worry too long after an embarrassing experience \|\| id:ebi-a-GCST006946 | rs2191130 | T | G | 0.678282 | 0.015113 | 0.00253872 |
| Worry too long after an embarrassing experience \|\| id:ebi-a-GCST006946 | rs2734837 | T | C | 0.594128 | -0.0144235 | 0.00238366 |
| Worry too long after an embarrassing experience \|\| id:ebi-a-GCST006946 | rs34588274 | T | C | 0.56477 | -0.0196466 | 0.00238169 |
| Worry too long after an embarrassing experience \|\| id:ebi-a-GCST006946 | rs35267052 | G | T | 0.101497 | 0.0217077 | 0.00387016 |
| Worry too long after an embarrassing experience \|\| id:ebi-a-GCST006946 | rs35327499 | A | G | 0.102548 | 0.0218721 | 0.00389184 |
| Worry too long after an embarrassing experience \|\| id:ebi-a-GCST006946 | rs3742021 | C | T | 0.302806 | 0.0157492 | 0.00254841 |
| Worry too long after an embarrassing experience \|\| id:ebi-a-GCST006946 | rs3777095 | A | G | 0.46041 | -0.0133434 | 0.00234919 |
| Worry too long after an embarrassing experience \|\| id:ebi-a-GCST006946 | rs3999543 | A | G | 0.305513 | 0.0145684 | 0.0025803 |
| Worry too long after an embarrassing experience \|\| id:ebi-a-GCST006946 | rs406204 | T | C | 0.206853 | -0.0157639 | 0.00289034 |
| Worry too long after an embarrassing experience \|\| id:ebi-a-GCST006946 | rs6439649 | T | G | 0.60374 | 0.0156954 | 0.00238677 |
| Worry too long after an embarrassing experience \|\| id:ebi-a-GCST006946 | rs72765272 | C | A | 0.1317 | -0.0212729 | 0.00345284 |
| Worry too long after an embarrassing experience \|\| id:ebi-a-GCST006946 | rs7987467 | A | G | 0.407619 | 0.0133593 | 0.00238048 |
| Worry too long after an embarrassing experience \|\| id:ebi-a-GCST006946 | rs9811585 | G | T | 0.384784 | 0.017108 | 0.00240179 |
| Feeling hurt \|\| id:ebi-a-GCST006951 | rs10210652 | A | G | 0.347252 | 0.0153477 | 0.00243807 |
| Feeling hurt \|\| id:ebi-a-GCST006951 | rs10511285 | G | T | 0.459246 | 0.0130829 | 0.0023484 |
| Feeling hurt \|\| id:ebi-a-GCST006951 | rs10850379 | T | C | 0.425983 | 0.0143923 | 0.00234593 |
| Feeling hurt \|\| id:ebi-a-GCST006951 | rs10891564 | A | G | 0.388119 | 0.0148506 | 0.00238143 |
| Feeling hurt \|\| id:ebi-a-GCST006951 | rs11663050 | G | T | 0.642196 | -0.0196964 | 0.00242357 |
| Feeling hurt \|\| id:ebi-a-GCST006951 | rs11767715 | T | C | 0.132418 | -0.0200211 | 0.00344657 |
| Feeling hurt \|\| id:ebi-a-GCST006951 | rs12028465 | A | G | 0.400161 | -0.0136625 | 0.00236908 |
| Feeling hurt \|\| id:ebi-a-GCST006951 | rs1231375 | C | T | 0.861237 | -0.019133 | 0.00336197 |
| Feeling hurt \|\| id:ebi-a-GCST006951 | rs12933611 | G | T | 0.373652 | 0.0131518 | 0.00240876 |
| Feeling hurt \|\| id:ebi-a-GCST006951 | rs145965565 | G | T | 0.0906455 | 0.0269671 | 0.00405155 |
| Feeling hurt \|\| id:ebi-a-GCST006951 | rs17532098 | T | C | 0.102583 | 0.0213159 | 0.00383035 |
| Feeling hurt \|\| id:ebi-a-GCST006951 | rs1978573 | T | C | 0.300324 | -0.0142756 | 0.00254784 |
| Feeling hurt \|\| id:ebi-a-GCST006951 | rs2027798 | T | C | 0.580897 | -0.013436 | 0.00236216 |
| Feeling hurt \|\| id:ebi-a-GCST006951 | rs2102923 | G | A | 0.627608 | 0.0139333 | 0.00241354 |
| Feeling hurt \|\| id:ebi-a-GCST006951 | rs219226 | C | T | 0.757344 | -0.0148161 | 0.00271755 |
| Feeling hurt \|\| id:ebi-a-GCST006951 | rs2488401 | T | C | 0.209268 | -0.0186452 | 0.00289027 |
| Feeling hurt \|\| id:ebi-a-GCST006951 | rs4652676 | A | G | 0.246938 | 0.0148069 | 0.0027005 |
| Feeling hurt \|\| id:ebi-a-GCST006951 | rs4702 | A | G | 0.561859 | -0.0140195 | 0.00233776 |
| Feeling hurt \|\| id:ebi-a-GCST006951 | rs4791774 | G | A | 0.463908 | -0.0128464 | 0.00234081 |
| Feeling hurt \|\| id:ebi-a-GCST006951 | rs4868774 | G | A | 0.177192 | -0.0172731 | 0.0030757 |
| Feeling hurt \|\| id:ebi-a-GCST006951 | rs545853 | A | G | 0.537648 | -0.0130672 | 0.00235276 |
| Feeling hurt \|\| id:ebi-a-GCST006951 | rs55657917 | G | T | 0.213403 | 0.0317281 | 0.00284429 |
| Feeling hurt \|\| id:ebi-a-GCST006951 | rs62035176 | A | G | 0.641118 | -0.013401 | 0.00242639 |
| Feeling hurt \|\| id:ebi-a-GCST006951 | rs73480560 | T | C | 0.260712 | 0.0150136 | 0.00268197 |
| Feeling tense \|\| id:ebi-a-GCST006952 | rs10767733 | A | G | 0.402825 | -0.0149582 | 0.00238567 |
| Feeling tense \|\| id:ebi-a-GCST006952 | rs11090045 | A | G | 0.30231 | 0.0164465 | 0.00257782 |
| Feeling tense \|\| id:ebi-a-GCST006952 | rs1147851 | G | A | 0.284644 | 0.0151053 | 0.00258077 |
| Feeling tense \|\| id:ebi-a-GCST006952 | rs11509880 | A | G | 0.344232 | 0.0148836 | 0.00244797 |
| Feeling tense \|\| id:ebi-a-GCST006952 | rs1450832 | A | G | 0.290096 | 0.0156599 | 0.00257946 |
| Feeling tense \|\| id:ebi-a-GCST006952 | rs2097247 | T | C | 0.0958248 | 0.0234116 | 0.00394467 |
| Feeling tense \|\| id:ebi-a-GCST006952 | rs28738966 | A | G | 0.217446 | 0.017533 | 0.00281791 |
| Feeling tense \|\| id:ebi-a-GCST006952 | rs3751855 | C | T | 0.393888 | -0.0134069 | 0.00237626 |
| Feeling tense \|\| id:ebi-a-GCST006952 | rs4129585 | C | A | 0.573268 | -0.0144294 | 0.00234739 |
| Feeling tense \|\| id:ebi-a-GCST006952 | rs4671330 | A | G | 0.664616 | 0.015168 | 0.00245915 |
| Feeling tense \|\| id:ebi-a-GCST006952 | rs4937872 | G | A | 0.417009 | 0.0156423 | 0.00238087 |
| Feeling tense \|\| id:ebi-a-GCST006952 | rs56084168 | T | C | 0.147994 | -0.0178945 | 0.00327438 |
| Feeling tense \|\| id:ebi-a-GCST006952 | rs7194615 | C | T | 0.551498 | 0.0131511 | 0.00236659 |
| Feeling tense \|\| id:ebi-a-GCST006952 | rs78379741 | C | T | 0.181714 | -0.0185314 | 0.00301176 |
| Feeling tense \|\| id:ebi-a-GCST006952 | rs79861172 | A | G | 0.0918161 | 0.0219756 | 0.0040219 |
| Feeling tense \|\| id:ebi-a-GCST006952 | rs9527336 | A | G | 0.722762 | -0.0152863 | 0.00259662 |
| Feeling tense \|\| id:ebi-a-GCST006952 | rs9811546 | A | G | 0.311971 | -0.0146051 | 0.00250602 |
| Neuroticism \|\| id:ebi-a-GCST005232 | rs10455007 | C | A | NA | -0.035297 | 0.0056489 |
| Neuroticism \|\| id:ebi-a-GCST005232 | rs10457809 | T | C | NA | 0.031524 | 0.0055964 |
| Neuroticism \|\| id:ebi-a-GCST005232 | rs10497655 | C | T | NA | -0.03172 | 0.0055758 |
| Neuroticism \|\| id:ebi-a-GCST005232 | rs1050846 | A | G | NA | -0.031983 | 0.0056288 |
| Neuroticism \|\| id:ebi-a-GCST005232 | rs10757410 | C | T | NA | -0.035061 | 0.0056377 |
| Neuroticism \|\| id:ebi-a-GCST005232 | rs10959926 | C | T | NA | -0.041389 | 0.0055145 |
| Neuroticism \|\| id:ebi-a-GCST005232 | rs11068870 | C | A | NA | 0.031666 | 0.005646 |
| Neuroticism \|\| id:ebi-a-GCST005232 | rs11082011 | T | C | NA | -0.047133 | 0.0056933 |
| Neuroticism \|\| id:ebi-a-GCST005232 | rs11090045 | A | G | NA | 0.039957 | 0.0055799 |
| Neuroticism \|\| id:ebi-a-GCST005232 | rs1109451 | A | G | NA | -0.031803 | 0.0055939 |
| Neuroticism \|\| id:ebi-a-GCST005232 | rs11605020 | A | G | NA | 0.031822 | 0.0056048 |
| Neuroticism \|\| id:ebi-a-GCST005232 | rs11759026 | G | A | NA | -0.031082 | 0.0056151 |
| Neuroticism \|\| id:ebi-a-GCST005232 | rs1282545 | C | T | NA | 0.03487 | 0.0056205 |
| Neuroticism \|\| id:ebi-a-GCST005232 | rs12896360 | C | A | NA | 0.040739 | 0.005607 |
| Neuroticism \|\| id:ebi-a-GCST005232 | rs13226841 | C | T | NA | 0.039546 | 0.0056143 |
| Neuroticism \|\| id:ebi-a-GCST005232 | rs13239186 | T | C | NA | 0.035111 | 0.0055773 |
| Neuroticism \|\| id:ebi-a-GCST005232 | rs1422192 | A | G | NA | 0.034587 | 0.0057392 |
| Neuroticism \|\| id:ebi-a-GCST005232 | rs1542212 | G | T | NA | 0.038547 | 0.0055875 |
| Neuroticism \|\| id:ebi-a-GCST005232 | rs169235 | G | A | NA | 0.033031 | 0.0056122 |
| Neuroticism \|\| id:ebi-a-GCST005232 | rs17522826 | A | G | NA | 0.033921 | 0.0055833 |
| Neuroticism \|\| id:ebi-a-GCST005232 | rs1870293 | C | T | NA | 0.035635 | 0.0056333 |
| Neuroticism \|\| id:ebi-a-GCST005232 | rs2042555 | A | G | NA | 0.039164 | 0.0055916 |
| Neuroticism \|\| id:ebi-a-GCST005232 | rs2071754 | T | C | NA | -0.038319 | 0.0057154 |
| Neuroticism \|\| id:ebi-a-GCST005232 | rs2149351 | G | T | NA | -0.035437 | 0.0055438 |
| Neuroticism \|\| id:ebi-a-GCST005232 | rs2244497 | T | C | NA | 0.031228 | 0.0056524 |
| Neuroticism \|\| id:ebi-a-GCST005232 | rs2269426 | A | G | NA | 0.037762 | 0.0055953 |
| Neuroticism \|\| id:ebi-a-GCST005232 | rs2380937 | C | T | NA | -0.035732 | 0.0055844 |
| Neuroticism \|\| id:ebi-a-GCST005232 | rs2678897 | A | G | NA | 0.032999 | 0.0055896 |
| Neuroticism \|\| id:ebi-a-GCST005232 | rs2921036 | C | T | NA | -0.058815 | 0.0055974 |
| Neuroticism \|\| id:ebi-a-GCST005232 | rs297346 | G | A | NA | -0.031816 | 0.0056023 |
| Neuroticism \|\| id:ebi-a-GCST005232 | rs34862781 | A | G | NA | -0.041242 | 0.0056308 |
| Neuroticism \|\| id:ebi-a-GCST005232 | rs3785232 | T | C | NA | 0.032548 | 0.0055886 |
| Neuroticism \|\| id:ebi-a-GCST005232 | rs3793577 | G | A | NA | 0.032741 | 0.005579 |
| Neuroticism \|\| id:ebi-a-GCST005232 | rs4140799 | A | G | NA | -0.033127 | 0.0056039 |
| Neuroticism \|\| id:ebi-a-GCST005232 | rs4362360 | C | T | NA | -0.032966 | 0.0056526 |
| Neuroticism \|\| id:ebi-a-GCST005232 | rs4585149 | C | T | NA | 0.030585 | 0.0055495 |
| Neuroticism \|\| id:ebi-a-GCST005232 | rs4653218 | C | T | NA | -0.038068 | 0.0056111 |
| Neuroticism \|\| id:ebi-a-GCST005232 | rs4673866 | G | A | NA | 0.032568 | 0.0056941 |
| Neuroticism \|\| id:ebi-a-GCST005232 | rs4841306 | T | C | NA | -0.037486 | 0.0056034 |
| Neuroticism \|\| id:ebi-a-GCST005232 | rs4899292 | G | A | NA | 0.031143 | 0.0055739 |
| Neuroticism \|\| id:ebi-a-GCST005232 | rs4911448 | T | C | NA | 0.031661 | 0.0056212 |
| Neuroticism \|\| id:ebi-a-GCST005232 | rs496939 | G | A | NA | 0.034727 | 0.0055986 |
| Neuroticism \|\| id:ebi-a-GCST005232 | rs56403421 | C | A | NA | 0.03675 | 0.0055758 |
| Neuroticism \|\| id:ebi-a-GCST005232 | rs59143394 | G | A | NA | 0.031861 | 0.0056759 |
| Neuroticism \|\| id:ebi-a-GCST005232 | rs60150206 | A | G | NA | 0.038299 | 0.0055872 |
| Neuroticism \|\| id:ebi-a-GCST005232 | rs60668206 | T | C | NA | 0.030238 | 0.0055416 |
| Neuroticism \|\| id:ebi-a-GCST005232 | rs6476086 | G | A | NA | -0.032466 | 0.0056448 |
| Neuroticism \|\| id:ebi-a-GCST005232 | rs6479494 | G | A | NA | 0.031329 | 0.0057252 |
| Neuroticism \|\| id:ebi-a-GCST005232 | rs6606710 | C | T | NA | 0.037417 | 0.0056155 |
| Neuroticism \|\| id:ebi-a-GCST005232 | rs7107356 | G | A | NA | 0.039555 | 0.0055923 |
| Neuroticism \|\| id:ebi-a-GCST005232 | rs7175083 | C | T | NA | -0.034027 | 0.0055905 |
| Neuroticism \|\| id:ebi-a-GCST005232 | rs7502590 | G | A | NA | -0.0371 | 0.0055642 |
| Neuroticism \|\| id:ebi-a-GCST005232 | rs7567451 | T | G | NA | 0.032477 | 0.0057035 |
| Neuroticism \|\| id:ebi-a-GCST005232 | rs7578651 | C | T | NA | 0.030648 | 0.0055918 |
| Neuroticism \|\| id:ebi-a-GCST005232 | rs76064345 | G | T | NA | 0.030503 | 0.00549 |
| Neuroticism \|\| id:ebi-a-GCST005232 | rs7696796 | A | G | NA | 0.038673 | 0.0056819 |
| Neuroticism \|\| id:ebi-a-GCST005232 | rs77484855 | G | T | NA | 0.030473 | 0.005491 |
| Neuroticism \|\| id:ebi-a-GCST005232 | rs77804065 | T | C | NA | 0.057242 | 0.0055267 |
| Neuroticism \|\| id:ebi-a-GCST005232 | rs802425 | T | C | NA | -0.032848 | 0.0056251 |
| Neuroticism \|\| id:ebi-a-GCST005232 | rs8063603 | A | G | NA | -0.032168 | 0.0057173 |
| Neuroticism \|\| id:ebi-a-GCST005232 | rs860626 | G | T | NA | -0.032304 | 0.0056439 |
| Neuroticism \|\| id:ebi-a-GCST005232 | rs877995 | A | G | NA | 0.032509 | 0.0055878 |
| Neuroticism \|\| id:ebi-a-GCST005232 | rs895941 | C | A | NA | 0.037512 | 0.0056427 |
| Neuroticism \|\| id:ebi-a-GCST005232 | rs9398586 | G | A | NA | 0.03467 | 0.0056479 |
| Neuroticism \|\| id:ebi-a-GCST005232 | rs9572015 | A | G | NA | 0.033687 | 0.005563 |
| Neuroticism score \|\| id:ukb-a-230 | rs10119773 | G | A | 0.53859 | 0.051675 | 0.008805 |
| Neuroticism score \|\| id:ukb-a-230 | rs10144845 | T | C | 0.683018 | 0.0653467 | 0.00932979 |
| Neuroticism score \|\| id:ukb-a-230 | rs10455007 | C | A | 0.629585 | -0.0575024 | 0.00901497 |
| Neuroticism score \|\| id:ukb-a-230 | rs10497655 | C | T | 0.320601 | -0.0520515 | 0.00930282 |
| Neuroticism score \|\| id:ukb-a-230 | rs10501696 | G | A | 0.507886 | -0.0503825 | 0.00892867 |
| Neuroticism score \|\| id:ukb-a-230 | rs11090045 | A | G | 0.308081 | 0.066709 | 0.00953335 |
| Neuroticism score \|\| id:ukb-a-230 | rs11509880 | A | G | 0.328208 | 0.0554247 | 0.00924105 |
| Neuroticism score \|\| id:ukb-a-230 | rs11665070 | A | G | 0.668381 | -0.07715 | 0.00926185 |
| Neuroticism score \|\| id:ukb-a-230 | rs11682716 | G | T | 0.577963 | -0.0484274 | 0.00882296 |
| Neuroticism score \|\| id:ukb-a-230 | rs117298864 | A | G | 0.0456452 | 0.115332 | 0.0211279 |
| Neuroticism score \|\| id:ukb-a-230 | rs12938775 | A | G | 0.501111 | -0.052341 | 0.00869703 |
| Neuroticism score \|\| id:ukb-a-230 | rs12969553 | T | C | 0.611729 | -0.0571743 | 0.00893034 |
| Neuroticism score \|\| id:ukb-a-230 | rs13226841 | C | T | 0.489644 | 0.0571868 | 0.00868951 |
| Neuroticism score \|\| id:ukb-a-230 | rs1442129 | G | A | 0.545061 | 0.0488135 | 0.00876688 |
| Neuroticism score \|\| id:ukb-a-230 | rs147861665 | A | C | 0.0317247 | 0.153071 | 0.026288 |
| Neuroticism score \|\| id:ukb-a-230 | rs1542212 | G | T | 0.392061 | 0.0568198 | 0.00893685 |
| Neuroticism score \|\| id:ukb-a-230 | rs1673931 | C | T | 0.377497 | 0.0495318 | 0.00898982 |
| Neuroticism score \|\| id:ukb-a-230 | rs1806153 | T | G | 0.230518 | 0.0683861 | 0.010337 |
| Neuroticism score \|\| id:ukb-a-230 | rs2102341 | C | T | 0.708061 | -0.062971 | 0.00959266 |
| Neuroticism score \|\| id:ukb-a-230 | rs2206544 | C | T | 0.428299 | -0.0517845 | 0.00877954 |
| Neuroticism score \|\| id:ukb-a-230 | rs2269426 | A | G | 0.359411 | 0.0633577 | 0.0090516 |
| Neuroticism score \|\| id:ukb-a-230 | rs2278609 | C | T | 0.217772 | 0.0618677 | 0.0105708 |
| Neuroticism score \|\| id:ukb-a-230 | rs2715147 | T | C | 0.50112 | 0.0474863 | 0.00868651 |
| Neuroticism score \|\| id:ukb-a-230 | rs2791459 | A | C | 0.410557 | -0.0505981 | 0.00883543 |
| Neuroticism score \|\| id:ukb-a-230 | rs28427480 | C | A | 0.0948446 | 0.0926109 | 0.0149015 |
| Neuroticism score \|\| id:ukb-a-230 | rs2921036 | C | T | 0.510045 | -0.0842536 | 0.00872912 |
| Neuroticism score \|\| id:ukb-a-230 | rs297346 | G | A | 0.639268 | -0.0508393 | 0.00907908 |
| Neuroticism score \|\| id:ukb-a-230 | rs34796300 | C | T | 0.5743 | -0.0524742 | 0.00879109 |
| Neuroticism score \|\| id:ukb-a-230 | rs3741475 | A | G | 0.194756 | 0.0694654 | 0.0109836 |
| Neuroticism score \|\| id:ukb-a-230 | rs3811489 | C | T | 0.398615 | 0.0494735 | 0.00886868 |
| Neuroticism score \|\| id:ukb-a-230 | rs3849470 | C | T | 0.523273 | -0.0496181 | 0.00869862 |
| Neuroticism score \|\| id:ukb-a-230 | rs4140799 | A | G | 0.530254 | -0.056799 | 0.0087249 |
| Neuroticism score \|\| id:ukb-a-230 | rs4632195 | T | C | 0.514595 | 0.0591632 | 0.00870816 |
| Neuroticism score \|\| id:ukb-a-230 | rs4738602 | A | G | 0.48331 | 0.047984 | 0.00874061 |
| Neuroticism score \|\| id:ukb-a-230 | rs56116032 | G | A | 0.221307 | -0.0585119 | 0.0104758 |
| Neuroticism score \|\| id:ukb-a-230 | rs56226325 | T | C | 0.153377 | -0.0693845 | 0.0120648 |
| Neuroticism score \|\| id:ukb-a-230 | rs57838764 | C | T | 0.114443 | 0.0806422 | 0.013657 |
| Neuroticism score \|\| id:ukb-a-230 | rs59970005 | T | C | 0.205064 | -0.0646473 | 0.0108066 |
| Neuroticism score \|\| id:ukb-a-230 | rs62062288 | A | G | 0.218845 | 0.0991406 | 0.0105772 |
| Neuroticism score \|\| id:ukb-a-230 | rs6743916 | A | G | 0.293242 | -0.0539999 | 0.00957646 |
| Neuroticism score \|\| id:ukb-a-230 | rs6916891 | T | G | 0.120549 | 0.0771723 | 0.0135009 |
| Neuroticism score \|\| id:ukb-a-230 | rs6976111 | A | C | 0.303121 | 0.0575258 | 0.00955301 |
| Neuroticism score \|\| id:ukb-a-230 | rs7107293 | A | G | 0.433741 | -0.0708265 | 0.00877379 |
| Neuroticism score \|\| id:ukb-a-230 | rs7107356 | G | A | 0.505562 | 0.0604683 | 0.00869253 |
| Neuroticism score \|\| id:ukb-a-230 | rs716508 | T | C | 0.691687 | -0.0519118 | 0.00943184 |
| Neuroticism score \|\| id:ukb-a-230 | rs7338774 | G | A | 0.336978 | 0.0577056 | 0.00922862 |
| Neuroticism score \|\| id:ukb-a-230 | rs7502590 | G | A | 0.148386 | -0.0761008 | 0.0121919 |
| Neuroticism score \|\| id:ukb-a-230 | rs7567451 | T | G | 0.732332 | 0.0555167 | 0.00982795 |
| Neuroticism score \|\| id:ukb-a-230 | rs7869969 | G | A | 0.332132 | -0.0550852 | 0.00922988 |
| Neuroticism score \|\| id:ukb-a-230 | rs7871494 | T | C | 0.716507 | -0.0551554 | 0.00968421 |
| Neuroticism score \|\| id:ukb-a-230 | rs8053004 | T | C | 0.302926 | -0.0534188 | 0.00952935 |
| Neuroticism score \|\| id:ukb-a-230 | rs8062719 | G | A | 0.634797 | 0.050548 | 0.00907586 |
| Neuroticism score \|\| id:ukb-a-230 | rs836927 | A | C | 0.426772 | 0.0486721 | 0.00885568 |
| Neuroticism score \|\| id:ukb-a-230 | rs9298995 | A | G | 0.400548 | -0.0528905 | 0.00887553 |
| Neuroticism score \|\| id:ukb-a-230 | rs9424100 | G | A | 0.263472 | -0.054389 | 0.00988857 |
| Neuroticism score \|\| id:ukb-a-230 | rs9462364 | G | A | 0.507229 | 0.052062 | 0.0087109 |
| Body mass index (BMI) \|\| id:ukb-a-248 | rs10100245 | A | G | 0.56644 | 0.0206373 | 0.00242306 |
| Body mass index (BMI) \|\| id:ukb-a-248 | rs10185199 | A | G | 0.27894 | -0.018708 | 0.00274904 |
| Body mass index (BMI) \|\| id:ukb-a-248 | rs10187101 | T | C | 0.359822 | -0.0157126 | 0.00250464 |
| Body mass index (BMI) \|\| id:ukb-a-248 | rs10404726 | T | C | 0.466784 | -0.0198595 | 0.0024074 |
| Body mass index (BMI) \|\| id:ukb-a-248 | rs10465231 | T | C | 0.5494 | 0.0171399 | 0.00242726 |
| Body mass index (BMI) \|\| id:ukb-a-248 | rs1064213 | A | G | 0.476968 | 0.016189 | 0.00240561 |
| Body mass index (BMI) \|\| id:ukb-a-248 | rs10788493 | T | C | 0.45676 | 0.0136921 | 0.00241992 |
| Body mass index (BMI) \|\| id:ukb-a-248 | rs10803762 | A | G | 0.678865 | 0.0154637 | 0.0025818 |
| Body mass index (BMI) \|\| id:ukb-a-248 | rs10805383 | A | G | 0.479558 | 0.0169488 | 0.00240894 |
| Body mass index (BMI) \|\| id:ukb-a-248 | rs10865612 | C | T | 0.354736 | -0.0230841 | 0.00251075 |
| Body mass index (BMI) \|\| id:ukb-a-248 | rs10898330 | T | C | 0.525392 | -0.0142865 | 0.00241661 |
| Body mass index (BMI) \|\| id:ukb-a-248 | rs10938397 | G | A | 0.434075 | 0.0289533 | 0.00242913 |
| Body mass index (BMI) \|\| id:ukb-a-248 | rs10995427 | A | G | 0.360827 | -0.0165308 | 0.00251566 |
| Body mass index (BMI) \|\| id:ukb-a-248 | rs11012732 | G | A | 0.331106 | 0.0238495 | 0.00255513 |
| Body mass index (BMI) \|\| id:ukb-a-248 | rs11084554 | A | G | 0.156586 | -0.021578 | 0.0033044 |
| Body mass index (BMI) \|\| id:ukb-a-248 | rs11099020 | T | C | 0.642739 | -0.0153203 | 0.00251168 |
| Body mass index (BMI) \|\| id:ukb-a-248 | rs11150745 | G | A | 0.319489 | -0.0209841 | 0.00257946 |
| Body mass index (BMI) \|\| id:ukb-a-248 | rs11223641 | C | T | 0.144615 | -0.0191007 | 0.00343166 |
| Body mass index (BMI) \|\| id:ukb-a-248 | rs112520079 | G | T | 0.198951 | 0.0204582 | 0.00301413 |
| Body mass index (BMI) \|\| id:ukb-a-248 | rs11264489 | G | A | 0.361994 | 0.0140849 | 0.00250707 |
| Body mass index (BMI) \|\| id:ukb-a-248 | rs112693590 | A | G | 0.0497066 | -0.0329198 | 0.00567991 |
| Body mass index (BMI) \|\| id:ukb-a-248 | rs1127100 | C | T | 0.648239 | 0.0164916 | 0.00252199 |
| Body mass index (BMI) \|\| id:ukb-a-248 | rs113182412 | A | G | 0.165919 | -0.0193709 | 0.00328432 |
| Body mass index (BMI) \|\| id:ukb-a-248 | rs113230003 | A | G | 0.260185 | -0.0197805 | 0.00275909 |
| Body mass index (BMI) \|\| id:ukb-a-248 | rs113603865 | T | C | 0.212428 | 0.0197498 | 0.00295729 |
| Body mass index (BMI) \|\| id:ukb-a-248 | rs11515071 | T | C | 0.365135 | -0.0228095 | 0.00250575 |
| Body mass index (BMI) \|\| id:ukb-a-248 | rs11642015 | T | C | 0.40236 | 0.0723688 | 0.00244736 |
| Body mass index (BMI) \|\| id:ukb-a-248 | rs11655587 | T | C | 0.359868 | -0.0196375 | 0.00251089 |
| Body mass index (BMI) \|\| id:ukb-a-248 | rs11742930 | T | C | 0.566428 | 0.0143076 | 0.00242869 |
| Body mass index (BMI) \|\| id:ukb-a-248 | rs11757278 | C | T | 0.305848 | -0.0157921 | 0.00260882 |
| Body mass index (BMI) \|\| id:ukb-a-248 | rs11761411 | T | C | 0.154361 | -0.0184757 | 0.00333986 |
| Body mass index (BMI) \|\| id:ukb-a-248 | rs117632017 | A | G | 0.0391416 | 0.0363818 | 0.00644665 |
| Body mass index (BMI) \|\| id:ukb-a-248 | rs11782074 | T | G | 0.385641 | 0.0144947 | 0.00250672 |
| Body mass index (BMI) \|\| id:ukb-a-248 | rs11856579 | A | G | 0.268192 | -0.0197908 | 0.00271147 |
| Body mass index (BMI) \|\| id:ukb-a-248 | rs12024554 | T | C | 0.236905 | -0.0162677 | 0.00282749 |
| Body mass index (BMI) \|\| id:ukb-a-248 | rs12042959 | G | A | 0.145777 | -0.0205562 | 0.00342514 |
| Body mass index (BMI) \|\| id:ukb-a-248 | rs12049202 | T | C | 0.198651 | 0.0218362 | 0.00300823 |
| Body mass index (BMI) \|\| id:ukb-a-248 | rs12140153 | T | G | 0.0967607 | -0.0318248 | 0.00416782 |
| Body mass index (BMI) \|\| id:ukb-a-248 | rs12144626 | C | T | 0.581821 | -0.0172144 | 0.00244388 |
| Body mass index (BMI) \|\| id:ukb-a-248 | rs12477385 | T | G | 0.2273 | -0.0172504 | 0.00287583 |
| Body mass index (BMI) \|\| id:ukb-a-248 | rs12479357 | G | A | 0.631081 | 0.0178651 | 0.00249872 |
| Body mass index (BMI) \|\| id:ukb-a-248 | rs12614861 | T | G | 0.34701 | 0.0152338 | 0.00252179 |
| Body mass index (BMI) \|\| id:ukb-a-248 | rs12622280 | G | T | 0.157075 | -0.0187009 | 0.00330533 |
| Body mass index (BMI) \|\| id:ukb-a-248 | rs12679106 | T | G | 0.711341 | -0.0220302 | 0.00266493 |
| Body mass index (BMI) \|\| id:ukb-a-248 | rs1286138 | G | T | 0.673439 | 0.0180512 | 0.00256405 |
| Body mass index (BMI) \|\| id:ukb-a-248 | rs12877270 | A | G | 0.43845 | 0.0169078 | 0.00243897 |
| Body mass index (BMI) \|\| id:ukb-a-248 | rs12881629 | G | A | 0.0821459 | 0.0239675 | 0.00436626 |
| Body mass index (BMI) \|\| id:ukb-a-248 | rs12885458 | G | T | 0.509982 | -0.0156111 | 0.00240414 |
| Body mass index (BMI) \|\| id:ukb-a-248 | rs1296328 | C | A | 0.559914 | -0.0189787 | 0.00243087 |
| Body mass index (BMI) \|\| id:ukb-a-248 | rs12977259 | G | A | 0.821338 | 0.0186259 | 0.00316073 |
| Body mass index (BMI) \|\| id:ukb-a-248 | rs12992672 | A | G | 0.828635 | 0.0507808 | 0.00318245 |
| Body mass index (BMI) \|\| id:ukb-a-248 | rs13062093 | G | T | 0.366444 | 0.01706 | 0.00249069 |
| Body mass index (BMI) \|\| id:ukb-a-248 | rs13135092 | G | A | 0.0832342 | 0.0500549 | 0.00437445 |
| Body mass index (BMI) \|\| id:ukb-a-248 | rs13174863 | G | A | 0.148208 | 0.0249793 | 0.00340131 |
| Body mass index (BMI) \|\| id:ukb-a-248 | rs1320903 | A | G | 0.318441 | 0.022008 | 0.00258125 |
| Body mass index (BMI) \|\| id:ukb-a-248 | rs1327259 | G | A | 0.38613 | -0.0140026 | 0.00247527 |
| Body mass index (BMI) \|\| id:ukb-a-248 | rs1342391 | T | G | 0.669963 | 0.0158687 | 0.00256148 |
| Body mass index (BMI) \|\| id:ukb-a-248 | rs13427822 | G | A | 0.272052 | -0.0198988 | 0.00272868 |
| Body mass index (BMI) \|\| id:ukb-a-248 | rs1411432 | C | A | 0.183895 | 0.0249455 | 0.00310861 |
| Body mass index (BMI) \|\| id:ukb-a-248 | rs1441264 | A | G | 0.591866 | 0.0206382 | 0.00249668 |
| Body mass index (BMI) \|\| id:ukb-a-248 | rs1446585 | G | A | 0.228006 | -0.0171122 | 0.00287668 |
| Body mass index (BMI) \|\| id:ukb-a-248 | rs1458156 | T | C | 0.488264 | 0.0137851 | 0.0024053 |
| Body mass index (BMI) \|\| id:ukb-a-248 | rs1477290 | C | T | 0.135362 | 0.0335389 | 0.00353705 |
| Body mass index (BMI) \|\| id:ukb-a-248 | rs147730268 | T | G | 0.0905299 | -0.0361121 | 0.00432936 |
| Body mass index (BMI) \|\| id:ukb-a-248 | rs1582931 | A | G | 0.472057 | -0.0148636 | 0.00242597 |
| Body mass index (BMI) \|\| id:ukb-a-248 | rs16846140 | G | A | 0.33821 | 0.0161898 | 0.0025442 |
| Body mass index (BMI) \|\| id:ukb-a-248 | rs16916303 | G | A | 0.119234 | -0.020719 | 0.00373905 |
| Body mass index (BMI) \|\| id:ukb-a-248 | rs16975459 | C | A | 0.121305 | 0.0239721 | 0.00368583 |
| Body mass index (BMI) \|\| id:ukb-a-248 | rs17014332 | C | T | 0.210609 | 0.0180292 | 0.00294421 |
| Body mass index (BMI) \|\| id:ukb-a-248 | rs17024393 | C | T | 0.0257993 | 0.0675077 | 0.00759831 |
| Body mass index (BMI) \|\| id:ukb-a-248 | rs17058884 | G | T | 0.0450046 | -0.0318261 | 0.00580849 |
| Body mass index (BMI) \|\| id:ukb-a-248 | rs17085463 | A | G | 0.317007 | -0.0148137 | 0.00259815 |
| Body mass index (BMI) \|\| id:ukb-a-248 | rs17149254 | C | T | 0.808564 | -0.0238078 | 0.00311607 |
| Body mass index (BMI) \|\| id:ukb-a-248 | rs17342242 | G | A | 0.229795 | -0.016161 | 0.00286561 |
| Body mass index (BMI) \|\| id:ukb-a-248 | rs17399739 | G | A | 0.0695257 | 0.026237 | 0.00472237 |
| Body mass index (BMI) \|\| id:ukb-a-248 | rs17716502 | T | C | 0.207243 | -0.0222998 | 0.00298602 |
| Body mass index (BMI) \|\| id:ukb-a-248 | rs1788808 | G | A | 0.497341 | -0.0183135 | 0.0024054 |
| Body mass index (BMI) \|\| id:ukb-a-248 | rs1805123 | G | T | 0.246217 | -0.0179911 | 0.00278844 |
| Body mass index (BMI) \|\| id:ukb-a-248 | rs1884897 | G | A | 0.628254 | 0.0213286 | 0.00249224 |
| Body mass index (BMI) \|\| id:ukb-a-248 | rs1901241 | G | A | 0.160137 | 0.0192919 | 0.00329019 |
| Body mass index (BMI) \|\| id:ukb-a-248 | rs1919243 | C | T | 0.487067 | 0.0138729 | 0.00243342 |
| Body mass index (BMI) \|\| id:ukb-a-248 | rs1941706 | G | A | 0.463511 | 0.0139405 | 0.00241073 |
| Body mass index (BMI) \|\| id:ukb-a-248 | rs1949204 | G | T | 0.761765 | 0.0166526 | 0.00281935 |
| Body mass index (BMI) \|\| id:ukb-a-248 | rs2035806 | A | G | 0.566354 | -0.0166309 | 0.00242835 |
| Body mass index (BMI) \|\| id:ukb-a-248 | rs2046002 | C | T | 0.639044 | -0.014571 | 0.00250777 |
| Body mass index (BMI) \|\| id:ukb-a-248 | rs2121058 | C | T | 0.228644 | -0.0238147 | 0.00286076 |
| Body mass index (BMI) \|\| id:ukb-a-248 | rs2155869 | C | T | 0.819163 | -0.0184462 | 0.00311144 |
| Body mass index (BMI) \|\| id:ukb-a-248 | rs215634 | G | A | 0.613873 | -0.0147674 | 0.00247575 |
| Body mass index (BMI) \|\| id:ukb-a-248 | rs217672 | C | A | 0.272013 | 0.0158661 | 0.00270563 |
| Body mass index (BMI) \|\| id:ukb-a-248 | rs2192649 | G | T | 0.497183 | 0.0136694 | 0.00241616 |
| Body mass index (BMI) \|\| id:ukb-a-248 | rs2234458 | T | C | 0.639151 | -0.020641 | 0.002496 |
| Body mass index (BMI) \|\| id:ukb-a-248 | rs2292238 | C | A | 0.408667 | -0.0176981 | 0.00244772 |
| Body mass index (BMI) \|\| id:ukb-a-248 | rs2307111 | C | T | 0.393183 | -0.0289516 | 0.00246183 |
| Body mass index (BMI) \|\| id:ukb-a-248 | rs2318543 | G | A | 0.782495 | -0.0194021 | 0.00292162 |
| Body mass index (BMI) \|\| id:ukb-a-248 | rs2384054 | C | T | 0.489674 | 0.0350265 | 0.00240007 |
| Body mass index (BMI) \|\| id:ukb-a-248 | rs2398861 | G | A | 0.256599 | 0.0220894 | 0.0027614 |
| Body mass index (BMI) \|\| id:ukb-a-248 | rs241460 | G | A | 0.68187 | -0.0205388 | 0.00257525 |
| Body mass index (BMI) \|\| id:ukb-a-248 | rs2425857 | G | A | 0.554635 | -0.0135947 | 0.00241624 |
| Body mass index (BMI) \|\| id:ukb-a-248 | rs2439823 | G | A | 0.547705 | 0.0216364 | 0.00241939 |
| Body mass index (BMI) \|\| id:ukb-a-248 | rs2450445 | A | G | 0.323973 | -0.0153656 | 0.00256262 |
| Body mass index (BMI) \|\| id:ukb-a-248 | rs245775 | G | A | 0.728826 | 0.0203304 | 0.0027049 |
| Body mass index (BMI) \|\| id:ukb-a-248 | rs2470392 | C | T | 0.287428 | 0.0145453 | 0.00265977 |
| Body mass index (BMI) \|\| id:ukb-a-248 | rs2474898 | T | C | 0.345803 | 0.0146104 | 0.00252319 |
| Body mass index (BMI) \|\| id:ukb-a-248 | rs2482704 | T | G | 0.423422 | -0.0138253 | 0.00243157 |
| Body mass index (BMI) \|\| id:ukb-a-248 | rs2616192 | T | G | 0.67249 | 0.0140763 | 0.00257234 |
| Body mass index (BMI) \|\| id:ukb-a-248 | rs2678204 | G | T | 0.341813 | 0.0280873 | 0.00253578 |
| Body mass index (BMI) \|\| id:ukb-a-248 | rs2711111 | G | A | 0.565043 | -0.0147156 | 0.00245734 |
| Body mass index (BMI) \|\| id:ukb-a-248 | rs2725371 | G | A | 0.696308 | -0.0180883 | 0.00262057 |
| Body mass index (BMI) \|\| id:ukb-a-248 | rs273505 | C | T | 0.419906 | 0.0188626 | 0.00243413 |
| Body mass index (BMI) \|\| id:ukb-a-248 | rs28447555 | T | C | 0.180987 | 0.0185076 | 0.00312798 |
| Body mass index (BMI) \|\| id:ukb-a-248 | rs28489620 | A | G | 0.290982 | -0.0147327 | 0.0026634 |
| Body mass index (BMI) \|\| id:ukb-a-248 | rs2861685 | C | T | 0.411371 | -0.017647 | 0.00243763 |
| Body mass index (BMI) \|\| id:ukb-a-248 | rs2962082 | A | G | 0.482549 | -0.0135377 | 0.00240754 |
| Body mass index (BMI) \|\| id:ukb-a-248 | rs2975693 | C | T | 0.109281 | 0.0238256 | 0.0038753 |
| Body mass index (BMI) \|\| id:ukb-a-248 | rs34045288 | T | C | 0.335903 | 0.0259978 | 0.00254511 |
| Body mass index (BMI) \|\| id:ukb-a-248 | rs34095326 | A | G | 0.114134 | -0.0244429 | 0.00379235 |
| Body mass index (BMI) \|\| id:ukb-a-248 | rs34236292 | T | G | 0.32772 | -0.0140997 | 0.002567 |
| Body mass index (BMI) \|\| id:ukb-a-248 | rs34373881 | A | G | 0.278276 | -0.0168863 | 0.00268678 |
| Body mass index (BMI) \|\| id:ukb-a-248 | rs34774377 | C | T | 0.120337 | -0.0208434 | 0.00369099 |
| Body mass index (BMI) \|\| id:ukb-a-248 | rs34811474 | A | G | 0.231875 | -0.0305977 | 0.00284335 |
| Body mass index (BMI) \|\| id:ukb-a-248 | rs34966008 | T | C | 0.408148 | -0.0199158 | 0.00244306 |
| Body mass index (BMI) \|\| id:ukb-a-248 | rs35193668 | T | C | 0.361915 | -0.0166761 | 0.00249998 |
| Body mass index (BMI) \|\| id:ukb-a-248 | rs35483388 | T | C | 0.379617 | 0.0140802 | 0.00248601 |
| Body mass index (BMI) \|\| id:ukb-a-248 | rs35626515 | A | C | 0.407965 | 0.0265699 | 0.00244547 |
| Body mass index (BMI) \|\| id:ukb-a-248 | rs35722922 | G | A | 0.385007 | -0.0164622 | 0.00247822 |
| Body mass index (BMI) \|\| id:ukb-a-248 | rs357501 | A | G | 0.377514 | 0.0154044 | 0.00249087 |
| Body mass index (BMI) \|\| id:ukb-a-248 | rs35851183 | G | A | 0.357777 | 0.0167614 | 0.00250852 |
| Body mass index (BMI) \|\| id:ukb-a-248 | rs35882248 | T | C | 0.314642 | 0.0192316 | 0.00258946 |
| Body mass index (BMI) \|\| id:ukb-a-248 | rs36007635 | A | G | 0.138403 | -0.0200004 | 0.00348146 |
| Body mass index (BMI) \|\| id:ukb-a-248 | rs362307 | T | C | 0.0760278 | 0.0313537 | 0.00458157 |
| Body mass index (BMI) \|\| id:ukb-a-248 | rs3759584 | C | T | 0.361958 | -0.0165842 | 0.00251394 |
| Body mass index (BMI) \|\| id:ukb-a-248 | rs3802858 | C | T | 0.427488 | -0.0164713 | 0.00243005 |
| Body mass index (BMI) \|\| id:ukb-a-248 | rs3803286 | G | A | 0.666379 | -0.0207912 | 0.00254468 |
| Body mass index (BMI) \|\| id:ukb-a-248 | rs3810291 | A | G | 0.677478 | 0.029685 | 0.00256755 |
| Body mass index (BMI) \|\| id:ukb-a-248 | rs3843540 | C | T | 0.148581 | -0.0241729 | 0.00338227 |
| Body mass index (BMI) \|\| id:ukb-a-248 | rs3861879 | G | A | 0.43683 | 0.0137348 | 0.00243136 |
| Body mass index (BMI) \|\| id:ukb-a-248 | rs3897102 | T | C | 0.412248 | 0.0147261 | 0.00245956 |
| Body mass index (BMI) \|\| id:ukb-a-248 | rs390192 | G | A | 0.522445 | -0.0154416 | 0.00242065 |
| Body mass index (BMI) \|\| id:ukb-a-248 | rs4246657 | T | C | 0.340914 | 0.0170056 | 0.00253421 |
| Body mass index (BMI) \|\| id:ukb-a-248 | rs4261944 | G | T | 0.362956 | 0.0154894 | 0.00250441 |
| Body mass index (BMI) \|\| id:ukb-a-248 | rs4402589 | G | T | 0.55278 | 0.0291524 | 0.00241668 |
| Body mass index (BMI) \|\| id:ukb-a-248 | rs4467770 | A | G | 0.730668 | 0.0156691 | 0.00271693 |
| Body mass index (BMI) \|\| id:ukb-a-248 | rs4474229 | A | G | 0.372338 | -0.0158775 | 0.00248334 |
| Body mass index (BMI) \|\| id:ukb-a-248 | rs4482463 | A | C | 0.924181 | -0.0340855 | 0.00454517 |
| Body mass index (BMI) \|\| id:ukb-a-248 | rs4502882 | T | C | 0.656893 | -0.0147181 | 0.0025291 |
| Body mass index (BMI) \|\| id:ukb-a-248 | rs4595495 | G | A | 0.422607 | 0.0137384 | 0.0024356 |
| Body mass index (BMI) \|\| id:ukb-a-248 | rs4648450 | A | C | 0.466133 | -0.0154374 | 0.00242211 |
| Body mass index (BMI) \|\| id:ukb-a-248 | rs4671328 | G | T | 0.552555 | -0.0217722 | 0.00243483 |
| Body mass index (BMI) \|\| id:ukb-a-248 | rs4687770 | C | T | 0.133375 | -0.019339 | 0.00353549 |
| Body mass index (BMI) \|\| id:ukb-a-248 | rs4718964 | T | G | 0.412194 | 0.0145653 | 0.00244679 |
| Body mass index (BMI) \|\| id:ukb-a-248 | rs4757144 | A | G | 0.593047 | 0.016524 | 0.00244585 |
| Body mass index (BMI) \|\| id:ukb-a-248 | rs4777541 | T | C | 0.765041 | 0.0196505 | 0.00284642 |
| Body mass index (BMI) \|\| id:ukb-a-248 | rs4790841 | T | C | 0.154867 | -0.0295249 | 0.00332942 |
| Body mass index (BMI) \|\| id:ukb-a-248 | rs4911382 | T | C | 0.584238 | 0.015559 | 0.00244287 |
| Body mass index (BMI) \|\| id:ukb-a-248 | rs491711 | C | A | 0.310988 | -0.0158403 | 0.00260937 |
| Body mass index (BMI) \|\| id:ukb-a-248 | rs4921301 | T | C | 0.209817 | -0.0182238 | 0.0029841 |
| Body mass index (BMI) \|\| id:ukb-a-248 | rs4929923 | C | T | 0.647328 | 0.0173844 | 0.00251068 |
| Body mass index (BMI) \|\| id:ukb-a-248 | rs525101 | C | T | 0.371963 | 0.0164956 | 0.00249029 |
| Body mass index (BMI) \|\| id:ukb-a-248 | rs539515 | C | A | 0.207529 | 0.0474871 | 0.00295985 |
| Body mass index (BMI) \|\| id:ukb-a-248 | rs55689274 | A | G | 0.285711 | -0.0148341 | 0.00266266 |
| Body mass index (BMI) \|\| id:ukb-a-248 | rs55726687 | A | G | 0.211138 | 0.0238631 | 0.00294249 |
| Body mass index (BMI) \|\| id:ukb-a-248 | rs55938344 | C | A | 0.242702 | -0.0168213 | 0.00281823 |
| Body mass index (BMI) \|\| id:ukb-a-248 | rs56212061 | T | C | 0.149969 | -0.0211123 | 0.00337336 |
| Body mass index (BMI) \|\| id:ukb-a-248 | rs56803094 | G | A | 0.227111 | -0.0188821 | 0.00287406 |
| Body mass index (BMI) \|\| id:ukb-a-248 | rs57636386 | C | T | 0.0834151 | -0.0427651 | 0.00435306 |
| Body mass index (BMI) \|\| id:ukb-a-248 | rs58862095 | T | C | 0.419757 | -0.0243799 | 0.00243968 |
| Body mass index (BMI) \|\| id:ukb-a-248 | rs588660 | A | G | 0.585856 | 0.0183012 | 0.00244095 |
| Body mass index (BMI) \|\| id:ukb-a-248 | rs59104534 | T | C | 0.301112 | 0.0149782 | 0.00262903 |
| Body mass index (BMI) \|\| id:ukb-a-248 | rs5995843 | G | A | 0.348993 | -0.0174411 | 0.00251927 |
| Body mass index (BMI) \|\| id:ukb-a-248 | rs6050446 | G | A | 0.966803 | 0.0420771 | 0.0067598 |
| Body mass index (BMI) \|\| id:ukb-a-248 | rs60654199 | A | C | 0.0666476 | 0.0312664 | 0.00481001 |
| Body mass index (BMI) \|\| id:ukb-a-248 | rs60764613 | T | G | 0.144857 | 0.023587 | 0.00342645 |
| Body mass index (BMI) \|\| id:ukb-a-248 | rs61813324 | T | C | 0.134883 | 0.0279187 | 0.00356314 |
| Body mass index (BMI) \|\| id:ukb-a-248 | rs61826867 | G | A | 0.109772 | 0.0251347 | 0.00384169 |
| Body mass index (BMI) \|\| id:ukb-a-248 | rs61871615 | T | C | 0.0905228 | -0.0280254 | 0.0043732 |
| Body mass index (BMI) \|\| id:ukb-a-248 | rs61903695 | G | A | 0.255999 | 0.0163585 | 0.00275201 |
| Body mass index (BMI) \|\| id:ukb-a-248 | rs61969510 | C | T | 0.284412 | 0.0155328 | 0.00269504 |
| Body mass index (BMI) \|\| id:ukb-a-248 | rs62106258 | C | T | 0.0484669 | -0.0906926 | 0.0055919 |
| Body mass index (BMI) \|\| id:ukb-a-248 | rs62147189 | G | T | 0.622 | -0.0170068 | 0.00251319 |
| Body mass index (BMI) \|\| id:ukb-a-248 | rs62246314 | A | G | 0.101276 | 0.0228001 | 0.00398064 |
| Body mass index (BMI) \|\| id:ukb-a-248 | rs6265 | T | C | 0.189726 | -0.0402275 | 0.00306191 |
| Body mass index (BMI) \|\| id:ukb-a-248 | rs6536575 | C | T | 0.518496 | 0.0140621 | 0.00240451 |
| Body mass index (BMI) \|\| id:ukb-a-248 | rs6575340 | A | G | 0.637416 | 0.0228776 | 0.00250397 |
| Body mass index (BMI) \|\| id:ukb-a-248 | rs6601527 | A | C | 0.587819 | -0.0217383 | 0.00244545 |
| Body mass index (BMI) \|\| id:ukb-a-248 | rs66679256 | T | C | 0.446142 | 0.0162973 | 0.00242165 |
| Body mass index (BMI) \|\| id:ukb-a-248 | rs6687953 | G | A | 0.391351 | 0.0158953 | 0.00246141 |
| Body mass index (BMI) \|\| id:ukb-a-248 | rs66922415 | G | A | 0.234362 | 0.0520958 | 0.00283196 |
| Body mass index (BMI) \|\| id:ukb-a-248 | rs6705567 | C | T | 0.3788 | -0.0136224 | 0.00249537 |
| Body mass index (BMI) \|\| id:ukb-a-248 | rs6722241 | C | T | 0.270908 | -0.0201712 | 0.00271361 |
| Body mass index (BMI) \|\| id:ukb-a-248 | rs6739755 | G | A | 0.602976 | -0.0209594 | 0.00246047 |
| Body mass index (BMI) \|\| id:ukb-a-248 | rs67609008 | C | T | 0.283597 | 0.0146011 | 0.00267444 |
| Body mass index (BMI) \|\| id:ukb-a-248 | rs67844506 | G | A | 0.183242 | -0.0259162 | 0.0031086 |
| Body mass index (BMI) \|\| id:ukb-a-248 | rs6789488 | C | T | 0.750784 | 0.0200094 | 0.00277743 |
| Body mass index (BMI) \|\| id:ukb-a-248 | rs6809307 | T | C | 0.25622 | 0.0157072 | 0.00276033 |
| Body mass index (BMI) \|\| id:ukb-a-248 | rs6831020 | A | C | 0.295229 | -0.0159366 | 0.00263095 |
| Body mass index (BMI) \|\| id:ukb-a-248 | rs6861649 | C | T | 0.607485 | 0.0143476 | 0.00246927 |
| Body mass index (BMI) \|\| id:ukb-a-248 | rs6950388 | A | G | 0.795004 | 0.0172281 | 0.00297615 |
| Body mass index (BMI) \|\| id:ukb-a-248 | rs7030732 | A | C | 0.607889 | -0.0151605 | 0.00245902 |
| Body mass index (BMI) \|\| id:ukb-a-248 | rs704061 | C | T | 0.452703 | 0.0158698 | 0.0024138 |
| Body mass index (BMI) \|\| id:ukb-a-248 | rs7094644 | A | G | 0.674176 | 0.0152821 | 0.0026106 |
| Body mass index (BMI) \|\| id:ukb-a-248 | rs7116641 | G | T | 0.316797 | 0.0244218 | 0.00258645 |
| Body mass index (BMI) \|\| id:ukb-a-248 | rs7124681 | A | C | 0.408057 | 0.0267462 | 0.00243997 |
| Body mass index (BMI) \|\| id:ukb-a-248 | rs7132908 | A | G | 0.38392 | 0.0285337 | 0.0024723 |
| Body mass index (BMI) \|\| id:ukb-a-248 | rs7138383 | A | G | 0.253485 | -0.0213808 | 0.0027649 |
| Body mass index (BMI) \|\| id:ukb-a-248 | rs7141420 | T | C | 0.514078 | 0.0208148 | 0.00241922 |
| Body mass index (BMI) \|\| id:ukb-a-248 | rs71495049 | A | G | 0.0840305 | 0.0265499 | 0.00432971 |
| Body mass index (BMI) \|\| id:ukb-a-248 | rs7183417 | T | C | 0.432569 | 0.0149003 | 0.0024276 |
| Body mass index (BMI) \|\| id:ukb-a-248 | rs7195386 | C | T | 0.506441 | -0.0158814 | 0.00240747 |
| Body mass index (BMI) \|\| id:ukb-a-248 | rs7201895 | A | G | 0.355984 | -0.017337 | 0.00252451 |
| Body mass index (BMI) \|\| id:ukb-a-248 | rs7218014 | C | T | 0.196595 | 0.0206989 | 0.00302709 |
| Body mass index (BMI) \|\| id:ukb-a-248 | rs72697614 | A | C | 0.320396 | 0.014951 | 0.00260626 |
| Body mass index (BMI) \|\| id:ukb-a-248 | rs72820274 | A | G | 0.41876 | 0.0160021 | 0.00243934 |
| Body mass index (BMI) \|\| id:ukb-a-248 | rs72892910 | T | G | 0.170442 | 0.0401658 | 0.00320426 |
| Body mass index (BMI) \|\| id:ukb-a-248 | rs72976986 | A | G | 0.191187 | -0.0240493 | 0.00308786 |
| Body mass index (BMI) \|\| id:ukb-a-248 | rs73050254 | A | G | 0.136682 | 0.0193216 | 0.00350204 |
| Body mass index (BMI) \|\| id:ukb-a-248 | rs73144053 | A | C | 0.329919 | -0.0153557 | 0.00256046 |
| Body mass index (BMI) \|\| id:ukb-a-248 | rs73169730 | G | A | 0.276291 | 0.0194632 | 0.00268664 |
| Body mass index (BMI) \|\| id:ukb-a-248 | rs7321331 | A | G | 0.741132 | 0.0175546 | 0.00275513 |
| Body mass index (BMI) \|\| id:ukb-a-248 | rs7331420 | A | G | 0.284027 | -0.0147324 | 0.00267011 |
| Body mass index (BMI) \|\| id:ukb-a-248 | rs7498044 | A | G | 0.218661 | -0.0176487 | 0.00293198 |
| Body mass index (BMI) \|\| id:ukb-a-248 | rs750090 | C | T | 0.356153 | -0.0156336 | 0.00253473 |
| Body mass index (BMI) \|\| id:ukb-a-248 | rs75499503 | T | C | 0.219955 | -0.0197843 | 0.00294617 |
| Body mass index (BMI) \|\| id:ukb-a-248 | rs7553158 | A | G | 0.562389 | -0.0174152 | 0.0024236 |
| Body mass index (BMI) \|\| id:ukb-a-248 | rs75557510 | G | A | 0.0614963 | -0.0377881 | 0.00510406 |
| Body mass index (BMI) \|\| id:ukb-a-248 | rs756717 | A | G | 0.398859 | -0.0146721 | 0.00248166 |
| Body mass index (BMI) \|\| id:ukb-a-248 | rs76040172 | A | G | 0.0544397 | -0.0407564 | 0.00530826 |
| Body mass index (BMI) \|\| id:ukb-a-248 | rs7701777 | G | T | 0.282759 | -0.0170279 | 0.00266777 |
| Body mass index (BMI) \|\| id:ukb-a-248 | rs7719067 | G | A | 0.573835 | -0.0162027 | 0.00242599 |
| Body mass index (BMI) \|\| id:ukb-a-248 | rs7723426 | C | T | 0.675017 | 0.0146685 | 0.00256587 |
| Body mass index (BMI) \|\| id:ukb-a-248 | rs7755574 | T | G | 0.281153 | 0.0154959 | 0.00267437 |
| Body mass index (BMI) \|\| id:ukb-a-248 | rs7774 | A | C | 0.309138 | 0.0166342 | 0.00261526 |
| Body mass index (BMI) \|\| id:ukb-a-248 | rs778094 | A | G | 0.577568 | -0.0148535 | 0.00243672 |
| Body mass index (BMI) \|\| id:ukb-a-248 | rs7852189 | G | A | 0.311364 | 0.0165539 | 0.00258881 |
| Body mass index (BMI) \|\| id:ukb-a-248 | rs78565420 | T | C | 0.0541861 | 0.0337314 | 0.00545431 |
| Body mass index (BMI) \|\| id:ukb-a-248 | rs79113395 | A | G | 0.265449 | -0.0209147 | 0.00273235 |
| Body mass index (BMI) \|\| id:ukb-a-248 | rs7933085 | G | A | 0.509195 | 0.0157387 | 0.00241521 |
| Body mass index (BMI) \|\| id:ukb-a-248 | rs7941828 | T | C | 0.360093 | -0.0156019 | 0.00249966 |
| Body mass index (BMI) \|\| id:ukb-a-248 | rs7952102 | C | T | 0.387339 | -0.0152263 | 0.00246547 |
| Body mass index (BMI) \|\| id:ukb-a-248 | rs7992832 | T | C | 0.276544 | -0.0175556 | 0.00269236 |
| Body mass index (BMI) \|\| id:ukb-a-248 | rs799449 | T | C | 0.558481 | 0.019902 | 0.00242699 |
| Body mass index (BMI) \|\| id:ukb-a-248 | rs8015400 | A | C | 0.678151 | 0.0210882 | 0.0025723 |
| Body mass index (BMI) \|\| id:ukb-a-248 | rs80330591 | A | G | 0.146844 | -0.0213608 | 0.00339342 |
| Body mass index (BMI) \|\| id:ukb-a-248 | rs8078135 | T | C | 0.488467 | -0.0194999 | 0.00241182 |
| Body mass index (BMI) \|\| id:ukb-a-248 | rs8134638 | C | T | 0.375763 | 0.0151694 | 0.00248078 |
| Body mass index (BMI) \|\| id:ukb-a-248 | rs815163 | C | T | 0.561603 | -0.0182459 | 0.00241971 |
| Body mass index (BMI) \|\| id:ukb-a-248 | rs845084 | A | G | 0.257551 | 0.02034 | 0.0027569 |
| Body mass index (BMI) \|\| id:ukb-a-248 | rs862320 | T | C | 0.410152 | -0.0228157 | 0.0024463 |
| Body mass index (BMI) \|\| id:ukb-a-248 | rs869400 | G | T | 0.815827 | 0.0307255 | 0.00310717 |
| Body mass index (BMI) \|\| id:ukb-a-248 | rs879620 | T | C | 0.615276 | 0.0256124 | 0.00247819 |
| Body mass index (BMI) \|\| id:ukb-a-248 | rs9267671 | A | G | 0.060402 | 0.0323708 | 0.00504153 |
| Body mass index (BMI) \|\| id:ukb-a-248 | rs9291822 | T | C | 0.515455 | -0.0136148 | 0.00242583 |
| Body mass index (BMI) \|\| id:ukb-a-248 | rs9320823 | C | T | 0.601901 | 0.0189398 | 0.00245759 |
| Body mass index (BMI) \|\| id:ukb-a-248 | rs9342196 | T | C | 0.189501 | 0.0167326 | 0.00306601 |
| Body mass index (BMI) \|\| id:ukb-a-248 | rs935166 | A | G | 0.506935 | -0.0154943 | 0.00240282 |
| Body mass index (BMI) \|\| id:ukb-a-248 | rs9402104 | A | G | 0.584134 | 0.0139016 | 0.00245189 |
| Body mass index (BMI) \|\| id:ukb-a-248 | rs946185 | G | A | 0.593276 | -0.0146913 | 0.00246179 |
| Body mass index (BMI) \|\| id:ukb-a-248 | rs9515455 | A | G | 0.416606 | 0.0179637 | 0.00244962 |
| Body mass index (BMI) \|\| id:ukb-a-248 | rs9527906 | A | G | 0.762395 | -0.0161813 | 0.0028336 |
| Body mass index (BMI) \|\| id:ukb-a-248 | rs9641499 | A | C | 0.434095 | -0.017102 | 0.00242262 |
| Body mass index (BMI) \|\| id:ukb-a-248 | rs9688977 | C | T | 0.146486 | 0.0239775 | 0.00340602 |
| Body mass index (BMI) \|\| id:ukb-a-248 | rs9843653 | C | T | 0.514555 | 0.0317261 | 0.00240405 |
| Body mass index (BMI) \|\| id:ukb-a-248 | rs9847186 | A | G | 0.427737 | -0.0142849 | 0.0024335 |
| Average total household income before tax \|\| id:ukb-b-7408 | rs10429582 | C | T | 0.416388 | 0.0270261 | 0.00268405 |
| Average total household income before tax \|\| id:ukb-b-7408 | rs10761035 | A | G | 0.188004 | 0.0186402 | 0.00338512 |
| Average total household income before tax \|\| id:ukb-b-7408 | rs11191116 | T | C | 0.351782 | -0.0163993 | 0.00277643 |
| Average total household income before tax \|\| id:ukb-b-7408 | rs11588857 | A | G | 0.209469 | 0.0213402 | 0.00324615 |
| Average total household income before tax \|\| id:ukb-b-7408 | rs11678501 | C | T | 0.024727 | -0.0517926 | 0.00851645 |
| Average total household income before tax \|\| id:ukb-b-7408 | rs11714337 | A | G | 0.431007 | 0.0153807 | 0.00267881 |
| Average total household income before tax \|\| id:ukb-b-7408 | rs11877758 | G | T | 0.312055 | -0.0203602 | 0.00286697 |
| Average total household income before tax \|\| id:ukb-b-7408 | rs11917431 | T | C | 0.300831 | 0.0227135 | 0.00288021 |
| Average total household income before tax \|\| id:ukb-b-7408 | rs1229984 | C | T | 0.972949 | -0.0491072 | 0.00804788 |
| Average total household income before tax \|\| id:ukb-b-7408 | rs12531825 | A | G | 0.122596 | -0.0258177 | 0.00406172 |
| Average total household income before tax \|\| id:ukb-b-7408 | rs12692596 | T | C | 0.3717 | -0.0154093 | 0.00273347 |
| Average total household income before tax \|\| id:ukb-b-7408 | rs12883788 | T | C | 0.459864 | -0.0188574 | 0.00266308 |
| Average total household income before tax \|\| id:ukb-b-7408 | rs1421334 | C | A | 0.549035 | 0.0162948 | 0.00267028 |
| Average total household income before tax \|\| id:ukb-b-7408 | rs2068428 | T | C | 0.239041 | 0.0169724 | 0.00309515 |
| Average total household income before tax \|\| id:ukb-b-7408 | rs2332719 | G | A | 0.278917 | -0.0183042 | 0.00295323 |
| Average total household income before tax \|\| id:ukb-b-7408 | rs2362523 | G | A | 0.339926 | 0.0158667 | 0.00279836 |
| Average total household income before tax \|\| id:ukb-b-7408 | rs2422859 | G | T | 0.471922 | 0.0163711 | 0.00264718 |
| Average total household income before tax \|\| id:ukb-b-7408 | rs2515919 | G | A | 0.369978 | -0.0159385 | 0.00273539 |
| Average total household income before tax \|\| id:ukb-b-7408 | rs2820314 | C | A | 0.338157 | -0.0165601 | 0.00278855 |
| Average total household income before tax \|\| id:ukb-b-7408 | rs32940 | C | T | 0.700979 | 0.0211431 | 0.00289645 |
| Average total household income before tax \|\| id:ukb-b-7408 | rs34473884 | A | G | 0.248977 | 0.017225 | 0.00305604 |
| Average total household income before tax \|\| id:ukb-b-7408 | rs387780 | C | T | 0.674498 | 0.0167299 | 0.00282732 |
| Average total household income before tax \|\| id:ukb-b-7408 | rs488786 | T | C | 0.164058 | 0.0206878 | 0.00356762 |
| Average total household income before tax \|\| id:ukb-b-7408 | rs5754738 | G | A | 0.709551 | -0.0162464 | 0.00292038 |
| Average total household income before tax \|\| id:ukb-b-7408 | rs6035877 | C | A | 0.469244 | -0.0145138 | 0.00265042 |
| Average total household income before tax \|\| id:ukb-b-7408 | rs62183028 | T | G | 0.310562 | -0.0189832 | 0.00286114 |
| Average total household income before tax \|\| id:ukb-b-7408 | rs6429636 | T | G | 0.719691 | 0.0192858 | 0.00293518 |
| Average total household income before tax \|\| id:ukb-b-7408 | rs6699397 | G | A | 0.369644 | -0.0190757 | 0.00274153 |
| Average total household income before tax \|\| id:ukb-b-7408 | rs6868457 | C | T | 0.478238 | 0.0209797 | 0.0026601 |
| Average total household income before tax \|\| id:ukb-b-7408 | rs71576284 | A | C | 0.008961 | -0.0838859 | 0.0151769 |
| Average total household income before tax \|\| id:ukb-b-7408 | rs73015322 | T | G | 0.076734 | -0.0271853 | 0.00495368 |
| Average total household income before tax \|\| id:ukb-b-7408 | rs75413320 | C | T | 0.108161 | -0.0261129 | 0.00426829 |
| Average total household income before tax \|\| id:ukb-b-7408 | rs7700107 | C | A | 0.138299 | -0.0228296 | 0.00382992 |
| Average total household income before tax \|\| id:ukb-b-7408 | rs77126132 | A | G | 0.093289 | 0.0267483 | 0.00457921 |
| Average total household income before tax \|\| id:ukb-b-7408 | rs784256 | A | G | 0.810934 | -0.0253813 | 0.00339684 |
| Average total household income before tax \|\| id:ukb-b-7408 | rs7896518 | G | A | 0.428029 | 0.014813 | 0.00270047 |
| Average total household income before tax \|\| id:ukb-b-7408 | rs9388490 | T | C | 0.439901 | 0.015105 | 0.00266861 |
| Average total household income before tax \|\| id:ukb-b-7408 | rs9556958 | T | C | 0.524062 | -0.0153409 | 0.0026641 |
| Average total household income before tax \|\| id:ukb-b-7408 | rs968050 | T | C | 0.483185 | 0.0223829 | 0.00265073 |
| Average total household income before tax \|\| id:ukb-b-7408 | rs9891103 | T | C | 0.229018 | -0.0234517 | 0.00314818 |
| Types of physical activity in last 4 weeks: Heavy DIY (eg: weeding, lawn mowing, carpentry, digging) \|\| id:ukb-b-13184 | rs10200379 | A | C | 0.521925 | 0.00571317 | 0.00100832 |
| Types of physical activity in last 4 weeks: Heavy DIY (eg: weeding, lawn mowing, carpentry, digging) \|\| id:ukb-b-13184 | rs11076320 | A | C | 0.603737 | 0.0057882 | 0.00103307 |
| Types of physical activity in last 4 weeks: Heavy DIY (eg: weeding, lawn mowing, carpentry, digging) \|\| id:ukb-b-13184 | rs11116540 | A | G | 0.05492 | 0.0123265 | 0.00221866 |
| Types of physical activity in last 4 weeks: Heavy DIY (eg: weeding, lawn mowing, carpentry, digging) \|\| id:ukb-b-13184 | rs11984231 | A | G | 0.268802 | -0.00702148 | 0.00114399 |
| Types of physical activity in last 4 weeks: Heavy DIY (eg: weeding, lawn mowing, carpentry, digging) \|\| id:ukb-b-13184 | rs12203592 | T | C | 0.219648 | -0.00860434 | 0.0011968 |
| Types of physical activity in last 4 weeks: Heavy DIY (eg: weeding, lawn mowing, carpentry, digging) \|\| id:ukb-b-13184 | rs1288124 | T | C | 0.513028 | 0.00575294 | 0.00103958 |
| Types of physical activity in last 4 weeks: Heavy DIY (eg: weeding, lawn mowing, carpentry, digging) \|\| id:ukb-b-13184 | rs17157586 | C | T | 0.157764 | -0.00775277 | 0.00137998 |
| Types of physical activity in last 4 weeks: Heavy DIY (eg: weeding, lawn mowing, carpentry, digging) \|\| id:ukb-b-13184 | rs19573 | A | C | 0.795782 | 0.00735552 | 0.00125146 |
| Types of physical activity in last 4 weeks: Heavy DIY (eg: weeding, lawn mowing, carpentry, digging) \|\| id:ukb-b-13184 | rs2529676 | C | A | 0.352311 | -0.00602002 | 0.00105244 |
| Types of physical activity in last 4 weeks: Heavy DIY (eg: weeding, lawn mowing, carpentry, digging) \|\| id:ukb-b-13184 | rs2661990 | C | A | 0.36198 | 0.00595191 | 0.00105047 |
| Types of physical activity in last 4 weeks: Heavy DIY (eg: weeding, lawn mowing, carpentry, digging) \|\| id:ukb-b-13184 | rs292026 | A | G | 0.270271 | 0.00635251 | 0.00113377 |
| Types of physical activity in last 4 weeks: Heavy DIY (eg: weeding, lawn mowing, carpentry, digging) \|\| id:ukb-b-13184 | rs35683183 | A | G | 0.207531 | 0.0073699 | 0.00124764 |
| Types of physical activity in last 4 weeks: Heavy DIY (eg: weeding, lawn mowing, carpentry, digging) \|\| id:ukb-b-13184 | rs62523388 | A | C | 0.333913 | 0.00686211 | 0.00107975 |
| Types of physical activity in last 4 weeks: Heavy DIY (eg: weeding, lawn mowing, carpentry, digging) \|\| id:ukb-b-13184 | rs6481128 | A | G | 0.548275 | 0.00588548 | 0.00101247 |
| Types of physical activity in last 4 weeks: Heavy DIY (eg: weeding, lawn mowing, carpentry, digging) \|\| id:ukb-b-13184 | rs79313673 | C | T | 0.209653 | 0.00719391 | 0.0012434 |
