# Supplementary material for: Modifiable risk factors of major depressive disorder: A Mendelian randomization study
Source: PLoS One. 2023 Aug 3;18(8):e0289419. doi: 10.1371/journal.pone.0289419 (PMC10399902; doi:10.1371/journal.pone.0289419)
Supplement: S3 Table — SNPs, single nucleotide polymorphisms. (DOCX) [file pone.0289419.s006.docx]

**Table S3. Leave-one-out results of 19** **modifiable risk factors with the risk of MDD.**

| Exposure | Outcome | Sample Size | SNP | b | se | *P* |
| --- | --- | --- | --- | --- | --- | --- |
| Neuroticism \|\| id:ebi-a-GCST005232 | Major Depressive Disorder \|\| id:ieu-a-1188 | 173005 | rs10455007 | 0.3562065 | 0.04303128 | 1.2542E-16 |
| Neuroticism \|\| id:ebi-a-GCST005232 | Major Depressive Disorder \|\| id:ieu-a-1188 | 173005 | rs10457809 | 0.34569073 | 0.04296906 | 8.6169E-16 |
| Neuroticism \|\| id:ebi-a-GCST005232 | Major Depressive Disorder \|\| id:ieu-a-1188 | 173005 | rs10497655 | 0.35222934 | 0.0432132 | 3.6103E-16 |
| Neuroticism \|\| id:ebi-a-GCST005232 | Major Depressive Disorder \|\| id:ieu-a-1188 | 173005 | rs1050846 | 0.35300713 | 0.04317948 | 2.9502E-16 |
| Neuroticism \|\| id:ebi-a-GCST005232 | Major Depressive Disorder \|\| id:ieu-a-1188 | 173005 | rs10757410 | 0.35358561 | 0.04323908 | 2.899E-16 |
| Neuroticism \|\| id:ebi-a-GCST005232 | Major Depressive Disorder \|\| id:ieu-a-1188 | 173005 | rs10959926 | 0.34232942 | 0.04254543 | 8.5398E-16 |
| Neuroticism \|\| id:ebi-a-GCST005232 | Major Depressive Disorder \|\| id:ieu-a-1188 | 173005 | rs11068870 | 0.34294088 | 0.04169508 | 1.9524E-16 |
| Neuroticism \|\| id:ebi-a-GCST005232 | Major Depressive Disorder \|\| id:ieu-a-1188 | 173005 | rs11082011 | 0.34288506 | 0.04323207 | 2.1692E-15 |
| Neuroticism \|\| id:ebi-a-GCST005232 | Major Depressive Disorder \|\| id:ieu-a-1188 | 173005 | rs11090045 | 0.35484396 | 0.04321467 | 2.19E-16 |
| Neuroticism \|\| id:ebi-a-GCST005232 | Major Depressive Disorder \|\| id:ieu-a-1188 | 173005 | rs1109451 | 0.3581034 | 0.04252248 | 3.7167E-17 |
| Neuroticism \|\| id:ebi-a-GCST005232 | Major Depressive Disorder \|\| id:ieu-a-1188 | 173005 | rs11605020 | 0.35130383 | 0.04328705 | 4.8307E-16 |
| Neuroticism \|\| id:ebi-a-GCST005232 | Major Depressive Disorder \|\| id:ieu-a-1188 | 173005 | rs11759026 | 0.34932368 | 0.04313163 | 5.5407E-16 |
| Neuroticism \|\| id:ebi-a-GCST005232 | Major Depressive Disorder \|\| id:ieu-a-1188 | 173005 | rs1282545 | 0.35913233 | 0.04263531 | 3.6585E-17 |
| Neuroticism \|\| id:ebi-a-GCST005232 | Major Depressive Disorder \|\| id:ieu-a-1188 | 173005 | rs12896360 | 0.3481939 | 0.04338889 | 1.0156E-15 |
| Neuroticism \|\| id:ebi-a-GCST005232 | Major Depressive Disorder \|\| id:ieu-a-1188 | 173005 | rs13226841 | 0.3569966 | 0.0431789 | 1.364E-16 |
| Neuroticism \|\| id:ebi-a-GCST005232 | Major Depressive Disorder \|\| id:ieu-a-1188 | 173005 | rs13239186 | 0.34400156 | 0.0427647 | 8.6914E-16 |
| Neuroticism \|\| id:ebi-a-GCST005232 | Major Depressive Disorder \|\| id:ieu-a-1188 | 173005 | rs1422192 | 0.34526761 | 0.04258914 | 5.191E-16 |
| Neuroticism \|\| id:ebi-a-GCST005232 | Major Depressive Disorder \|\| id:ieu-a-1188 | 173005 | rs1542212 | 0.35980763 | 0.04269842 | 3.5549E-17 |
| Neuroticism \|\| id:ebi-a-GCST005232 | Major Depressive Disorder \|\| id:ieu-a-1188 | 173005 | rs169235 | 0.34259826 | 0.04223392 | 4.9825E-16 |
| Neuroticism \|\| id:ebi-a-GCST005232 | Major Depressive Disorder \|\| id:ieu-a-1188 | 173005 | rs17522826 | 0.34684817 | 0.04290179 | 6.2317E-16 |
| Neuroticism \|\| id:ebi-a-GCST005232 | Major Depressive Disorder \|\| id:ieu-a-1188 | 173005 | rs1870293 | 0.35203714 | 0.04324445 | 3.9322E-16 |
| Neuroticism \|\| id:ebi-a-GCST005232 | Major Depressive Disorder \|\| id:ieu-a-1188 | 173005 | rs2042555 | 0.35794067 | 0.04301695 | 8.7283E-17 |
| Neuroticism \|\| id:ebi-a-GCST005232 | Major Depressive Disorder \|\| id:ieu-a-1188 | 173005 | rs2071754 | 0.34540446 | 0.04289047 | 8.0674E-16 |
| Neuroticism \|\| id:ebi-a-GCST005232 | Major Depressive Disorder \|\| id:ieu-a-1188 | 173005 | rs2149351 | 0.34476171 | 0.04275343 | 7.3866E-16 |
| Neuroticism \|\| id:ebi-a-GCST005232 | Major Depressive Disorder \|\| id:ieu-a-1188 | 173005 | rs2244497 | 0.35313513 | 0.04315398 | 2.7654E-16 |
| Neuroticism \|\| id:ebi-a-GCST005232 | Major Depressive Disorder \|\| id:ieu-a-1188 | 173005 | rs2269426 | 0.34410285 | 0.04298837 | 1.199E-15 |
| Neuroticism \|\| id:ebi-a-GCST005232 | Major Depressive Disorder \|\| id:ieu-a-1188 | 173005 | rs2380937 | 0.35549064 | 0.04313794 | 1.7108E-16 |
| Neuroticism \|\| id:ebi-a-GCST005232 | Major Depressive Disorder \|\| id:ieu-a-1188 | 173005 | rs2678897 | 0.34921383 | 0.04326956 | 6.992E-16 |
| Neuroticism \|\| id:ebi-a-GCST005232 | Major Depressive Disorder \|\| id:ieu-a-1188 | 173005 | rs2921036 | 0.3637686 | 0.04355395 | 6.7038E-17 |
| Neuroticism \|\| id:ebi-a-GCST005232 | Major Depressive Disorder \|\| id:ieu-a-1188 | 173005 | rs297346 | 0.35107944 | 0.0432647 | 4.8705E-16 |
| Neuroticism \|\| id:ebi-a-GCST005232 | Major Depressive Disorder \|\| id:ieu-a-1188 | 173005 | rs34862781 | 0.35698051 | 0.04322977 | 1.4845E-16 |
| Neuroticism \|\| id:ebi-a-GCST005232 | Major Depressive Disorder \|\| id:ieu-a-1188 | 173005 | rs3785232 | 0.35187323 | 0.04324195 | 4.0415E-16 |
| Neuroticism \|\| id:ebi-a-GCST005232 | Major Depressive Disorder \|\| id:ieu-a-1188 | 173005 | rs3793577 | 0.34721286 | 0.04315452 | 8.568E-16 |
| Neuroticism \|\| id:ebi-a-GCST005232 | Major Depressive Disorder \|\| id:ieu-a-1188 | 173005 | rs4140799 | 0.35320041 | 0.04325446 | 3.1973E-16 |
| Neuroticism \|\| id:ebi-a-GCST005232 | Major Depressive Disorder \|\| id:ieu-a-1188 | 173005 | rs4362360 | 0.34810775 | 0.04323602 | 8.1894E-16 |
| Neuroticism \|\| id:ebi-a-GCST005232 | Major Depressive Disorder \|\| id:ieu-a-1188 | 173005 | rs4585149 | 0.35456386 | 0.04280156 | 1.1921E-16 |
| Neuroticism \|\| id:ebi-a-GCST005232 | Major Depressive Disorder \|\| id:ieu-a-1188 | 173005 | rs4653218 | 0.34561069 | 0.04319754 | 1.2371E-15 |
| Neuroticism \|\| id:ebi-a-GCST005232 | Major Depressive Disorder \|\| id:ieu-a-1188 | 173005 | rs4673866 | 0.34599737 | 0.04258928 | 4.5088E-16 |
| Neuroticism \|\| id:ebi-a-GCST005232 | Major Depressive Disorder \|\| id:ieu-a-1188 | 173005 | rs4841306 | 0.34977441 | 0.04330254 | 6.6131E-16 |
| Neuroticism \|\| id:ebi-a-GCST005232 | Major Depressive Disorder \|\| id:ieu-a-1188 | 173005 | rs4899292 | 0.35497175 | 0.04300391 | 1.5266E-16 |
| Neuroticism \|\| id:ebi-a-GCST005232 | Major Depressive Disorder \|\| id:ieu-a-1188 | 173005 | rs4911448 | 0.35324069 | 0.04300693 | 2.147E-16 |
| Neuroticism \|\| id:ebi-a-GCST005232 | Major Depressive Disorder \|\| id:ieu-a-1188 | 173005 | rs496939 | 0.34662103 | 0.04316792 | 9.7793E-16 |
| Neuroticism \|\| id:ebi-a-GCST005232 | Major Depressive Disorder \|\| id:ieu-a-1188 | 173005 | rs56403421 | 0.34555586 | 0.04305978 | 1.015E-15 |
| Neuroticism \|\| id:ebi-a-GCST005232 | Major Depressive Disorder \|\| id:ieu-a-1188 | 173005 | rs59143394 | 0.35210744 | 0.04314555 | 3.3248E-16 |
| Neuroticism \|\| id:ebi-a-GCST005232 | Major Depressive Disorder \|\| id:ieu-a-1188 | 173005 | rs60150206 | 0.34422815 | 0.04209316 | 2.8914E-16 |
| Neuroticism \|\| id:ebi-a-GCST005232 | Major Depressive Disorder \|\| id:ieu-a-1188 | 173005 | rs60668206 | 0.35051484 | 0.04314544 | 4.5095E-16 |
| Neuroticism \|\| id:ebi-a-GCST005232 | Major Depressive Disorder \|\| id:ieu-a-1188 | 173005 | rs6476086 | 0.35309215 | 0.0431439 | 2.7445E-16 |
| Neuroticism \|\| id:ebi-a-GCST005232 | Major Depressive Disorder \|\| id:ieu-a-1188 | 173005 | rs6479494 | 0.34468421 | 0.04214648 | 2.8801E-16 |
| Neuroticism \|\| id:ebi-a-GCST005232 | Major Depressive Disorder \|\| id:ieu-a-1188 | 173005 | rs6606710 | 0.34971687 | 0.04333542 | 7.0297E-16 |
| Neuroticism \|\| id:ebi-a-GCST005232 | Major Depressive Disorder \|\| id:ieu-a-1188 | 173005 | rs7107356 | 0.34545628 | 0.04325865 | 1.3958E-15 |
| Neuroticism \|\| id:ebi-a-GCST005232 | Major Depressive Disorder \|\| id:ieu-a-1188 | 173005 | rs7175083 | 0.35151626 | 0.04332357 | 4.9085E-16 |
| Neuroticism \|\| id:ebi-a-GCST005232 | Major Depressive Disorder \|\| id:ieu-a-1188 | 173005 | rs7502590 | 0.35174789 | 0.04313268 | 3.4912E-16 |
| Neuroticism \|\| id:ebi-a-GCST005232 | Major Depressive Disorder \|\| id:ieu-a-1188 | 173005 | rs7567451 | 0.34851544 | 0.04307178 | 5.8933E-16 |
| Neuroticism \|\| id:ebi-a-GCST005232 | Major Depressive Disorder \|\| id:ieu-a-1188 | 173005 | rs7578651 | 0.34488585 | 0.04281798 | 7.9677E-16 |
| Neuroticism \|\| id:ebi-a-GCST005232 | Major Depressive Disorder \|\| id:ieu-a-1188 | 173005 | rs76064345 | 0.35367313 | 0.04218922 | 5.1587E-17 |
| Neuroticism \|\| id:ebi-a-GCST005232 | Major Depressive Disorder \|\| id:ieu-a-1188 | 173005 | rs7696796 | 0.364496 | 0.04127279 | 1.0339E-18 |
| Neuroticism \|\| id:ebi-a-GCST005232 | Major Depressive Disorder \|\| id:ieu-a-1188 | 173005 | rs77484855 | 0.35101503 | 0.04303368 | 3.4416E-16 |
| Neuroticism \|\| id:ebi-a-GCST005232 | Major Depressive Disorder \|\| id:ieu-a-1188 | 173005 | rs77804065 | 0.35810742 | 0.04315453 | 1.0565E-16 |
| Neuroticism \|\| id:ebi-a-GCST005232 | Major Depressive Disorder \|\| id:ieu-a-1188 | 173005 | rs802425 | 0.35103906 | 0.04330279 | 5.2049E-16 |
| Neuroticism \|\| id:ebi-a-GCST005232 | Major Depressive Disorder \|\| id:ieu-a-1188 | 173005 | rs8063603 | 0.34386486 | 0.04235483 | 4.7133E-16 |
| Neuroticism \|\| id:ebi-a-GCST005232 | Major Depressive Disorder \|\| id:ieu-a-1188 | 173005 | rs860626 | 0.34613505 | 0.04292261 | 7.3738E-16 |
| Neuroticism \|\| id:ebi-a-GCST005232 | Major Depressive Disorder \|\| id:ieu-a-1188 | 173005 | rs877995 | 0.35274008 | 0.04309648 | 2.7254E-16 |
| Neuroticism \|\| id:ebi-a-GCST005232 | Major Depressive Disorder \|\| id:ieu-a-1188 | 173005 | rs895941 | 0.35637083 | 0.04301473 | 1.1825E-16 |
| Neuroticism \|\| id:ebi-a-GCST005232 | Major Depressive Disorder \|\| id:ieu-a-1188 | 173005 | rs9398586 | 0.3556022 | 0.04261994 | 7.2084E-17 |
| Neuroticism \|\| id:ebi-a-GCST005232 | Major Depressive Disorder \|\| id:ieu-a-1188 | 173005 | rs9572015 | 0.35604189 | 0.04295402 | 1.1429E-16 |
| Neuroticism \|\| id:ebi-a-GCST005232 | Major Depressive Disorder \|\| id:ieu-a-1188 | 173005 | All | 0.35078018 | 0.04261825 | 1.8604E-16 |
| Feeling guilty \|\| id:ebi-a-GCST006945 | Major Depressive Disorder \|\| id:ieu-a-1188 | 173005 | rs10119773 | 0.77931995 | 0.21273958 | 0.00024903 |
| Feeling guilty \|\| id:ebi-a-GCST006945 | Major Depressive Disorder \|\| id:ieu-a-1188 | 173005 | rs12420205 | 0.79241554 | 0.19601908 | 5.2875E-05 |
| Feeling guilty \|\| id:ebi-a-GCST006945 | Major Depressive Disorder \|\| id:ieu-a-1188 | 173005 | rs12528131 | 0.80408882 | 0.21509028 | 0.00018521 |
| Feeling guilty \|\| id:ebi-a-GCST006945 | Major Depressive Disorder \|\| id:ieu-a-1188 | 173005 | rs1557339 | 0.69508851 | 0.20679371 | 0.00077586 |
| Feeling guilty \|\| id:ebi-a-GCST006945 | Major Depressive Disorder \|\| id:ieu-a-1188 | 173005 | rs2109648 | 0.85803015 | 0.21176628 | 5.083E-05 |
| Feeling guilty \|\| id:ebi-a-GCST006945 | Major Depressive Disorder \|\| id:ieu-a-1188 | 173005 | rs34657012 | 0.88505108 | 0.20631205 | 1.7878E-05 |
| Feeling guilty \|\| id:ebi-a-GCST006945 | Major Depressive Disorder \|\| id:ieu-a-1188 | 173005 | rs55769038 | 0.91611917 | 0.20978171 | 1.2596E-05 |
| Feeling guilty \|\| id:ebi-a-GCST006945 | Major Depressive Disorder \|\| id:ieu-a-1188 | 173005 | rs681875 | 0.85339088 | 0.21250721 | 5.9239E-05 |
| Feeling guilty \|\| id:ebi-a-GCST006945 | Major Depressive Disorder \|\| id:ieu-a-1188 | 173005 | rs77804065 | 0.89174454 | 0.20375841 | 1.2061E-05 |
| Feeling guilty \|\| id:ebi-a-GCST006945 | Major Depressive Disorder \|\| id:ieu-a-1188 | 173005 | All | 0.83035226 | 0.19365329 | 1.8043E-05 |
| Worry too long after an embarrassing experience \|\| id:ebi-a-GCST006946 | Major Depressive Disorder \|\| id:ieu-a-1188 | 173005 | rs10750866 | 0.75570116 | 0.16825728 | 7.0775E-06 |
| Worry too long after an embarrassing experience \|\| id:ebi-a-GCST006946 | Major Depressive Disorder \|\| id:ieu-a-1188 | 173005 | rs1983614 | 0.86195696 | 0.17319118 | 6.4607E-07 |
| Worry too long after an embarrassing experience \|\| id:ebi-a-GCST006946 | Major Depressive Disorder \|\| id:ieu-a-1188 | 173005 | rs2191130 | 0.84669179 | 0.1788835 | 2.2101E-06 |
| Worry too long after an embarrassing experience \|\| id:ebi-a-GCST006946 | Major Depressive Disorder \|\| id:ieu-a-1188 | 173005 | rs2734837 | 0.75264718 | 0.16137422 | 3.1014E-06 |
| Worry too long after an embarrassing experience \|\| id:ebi-a-GCST006946 | Major Depressive Disorder \|\| id:ieu-a-1188 | 173005 | rs34588274 | 0.89363664 | 0.17808953 | 5.2238E-07 |
| Worry too long after an embarrassing experience \|\| id:ebi-a-GCST006946 | Major Depressive Disorder \|\| id:ieu-a-1188 | 173005 | rs35267052 | 0.78310922 | 0.17191803 | 5.2353E-06 |
| Worry too long after an embarrassing experience \|\| id:ebi-a-GCST006946 | Major Depressive Disorder \|\| id:ieu-a-1188 | 173005 | rs35327499 | 0.88144455 | 0.16789278 | 1.5206E-07 |
| Worry too long after an embarrassing experience \|\| id:ebi-a-GCST006946 | Major Depressive Disorder \|\| id:ieu-a-1188 | 173005 | rs3742021 | 0.78658814 | 0.17578395 | 7.6503E-06 |
| Worry too long after an embarrassing experience \|\| id:ebi-a-GCST006946 | Major Depressive Disorder \|\| id:ieu-a-1188 | 173005 | rs3777095 | 0.81000565 | 0.17883314 | 5.9153E-06 |
| Worry too long after an embarrassing experience \|\| id:ebi-a-GCST006946 | Major Depressive Disorder \|\| id:ieu-a-1188 | 173005 | rs3999543 | 0.82471433 | 0.17955386 | 4.3665E-06 |
| Worry too long after an embarrassing experience \|\| id:ebi-a-GCST006946 | Major Depressive Disorder \|\| id:ieu-a-1188 | 173005 | rs406204 | 0.81683432 | 0.17915444 | 5.1303E-06 |
| Worry too long after an embarrassing experience \|\| id:ebi-a-GCST006946 | Major Depressive Disorder \|\| id:ieu-a-1188 | 173005 | rs6439649 | 0.80800169 | 0.18061822 | 7.6937E-06 |
| Worry too long after an embarrassing experience \|\| id:ebi-a-GCST006946 | Major Depressive Disorder \|\| id:ieu-a-1188 | 173005 | rs72765272 | 0.84922203 | 0.17872542 | 2.0187E-06 |
| Worry too long after an embarrassing experience \|\| id:ebi-a-GCST006946 | Major Depressive Disorder \|\| id:ieu-a-1188 | 173005 | rs7987467 | 0.80140974 | 0.17715949 | 6.0779E-06 |
| Worry too long after an embarrassing experience \|\| id:ebi-a-GCST006946 | Major Depressive Disorder \|\| id:ieu-a-1188 | 173005 | rs9811585 | 0.92006795 | 0.15975725 | 8.4532E-09 |
| Worry too long after an embarrassing experience \|\| id:ebi-a-GCST006946 | Major Depressive Disorder \|\| id:ieu-a-1188 | 173005 | All | 0.82580187 | 0.16822105 | 9.1529E-07 |
| Feeling hurt \|\| id:ebi-a-GCST006951 | Major Depressive Disorder \|\| id:ieu-a-1188 | 173005 | rs10210652 | 0.86903618 | 0.15782708 | 3.6655E-08 |
| Feeling hurt \|\| id:ebi-a-GCST006951 | Major Depressive Disorder \|\| id:ieu-a-1188 | 173005 | rs10511285 | 0.80235778 | 0.16018458 | 5.4726E-07 |
| Feeling hurt \|\| id:ebi-a-GCST006951 | Major Depressive Disorder \|\| id:ieu-a-1188 | 173005 | rs10850379 | 0.80160455 | 0.16125864 | 6.6634E-07 |
| Feeling hurt \|\| id:ebi-a-GCST006951 | Major Depressive Disorder \|\| id:ieu-a-1188 | 173005 | rs10891564 | 0.8224628 | 0.1634399 | 4.8487E-07 |
| Feeling hurt \|\| id:ebi-a-GCST006951 | Major Depressive Disorder \|\| id:ieu-a-1188 | 173005 | rs11663050 | 0.7909496 | 0.16342983 | 1.3004E-06 |
| Feeling hurt \|\| id:ebi-a-GCST006951 | Major Depressive Disorder \|\| id:ieu-a-1188 | 173005 | rs11767715 | 0.85395482 | 0.16043017 | 1.0212E-07 |
| Feeling hurt \|\| id:ebi-a-GCST006951 | Major Depressive Disorder \|\| id:ieu-a-1188 | 173005 | rs12028465 | 0.85814089 | 0.15936907 | 7.26E-08 |
| Feeling hurt \|\| id:ebi-a-GCST006951 | Major Depressive Disorder \|\| id:ieu-a-1188 | 173005 | rs1231375 | 0.85004259 | 0.15894881 | 8.898E-08 |
| Feeling hurt \|\| id:ebi-a-GCST006951 | Major Depressive Disorder \|\| id:ieu-a-1188 | 173005 | rs12933611 | 0.79805768 | 0.15910463 | 5.2778E-07 |
| Feeling hurt \|\| id:ebi-a-GCST006951 | Major Depressive Disorder \|\| id:ieu-a-1188 | 173005 | rs145965565 | 0.8033563 | 0.16356528 | 9.0367E-07 |
| Feeling hurt \|\| id:ebi-a-GCST006951 | Major Depressive Disorder \|\| id:ieu-a-1188 | 173005 | rs17532098 | 0.79737171 | 0.15926813 | 5.5436E-07 |
| Feeling hurt \|\| id:ebi-a-GCST006951 | Major Depressive Disorder \|\| id:ieu-a-1188 | 173005 | rs1978573 | 0.85463815 | 0.15981778 | 8.9139E-08 |
| Feeling hurt \|\| id:ebi-a-GCST006951 | Major Depressive Disorder \|\| id:ieu-a-1188 | 173005 | rs2027798 | 0.84158731 | 0.16194314 | 2.0274E-07 |
| Feeling hurt \|\| id:ebi-a-GCST006951 | Major Depressive Disorder \|\| id:ieu-a-1188 | 173005 | rs2102923 | 0.8583404 | 0.15942076 | 7.28E-08 |
| Feeling hurt \|\| id:ebi-a-GCST006951 | Major Depressive Disorder \|\| id:ieu-a-1188 | 173005 | rs219226 | 0.86126511 | 0.15731538 | 4.3809E-08 |
| Feeling hurt \|\| id:ebi-a-GCST006951 | Major Depressive Disorder \|\| id:ieu-a-1188 | 173005 | rs2488401 | 0.80381757 | 0.1619923 | 6.9745E-07 |
| Feeling hurt \|\| id:ebi-a-GCST006951 | Major Depressive Disorder \|\| id:ieu-a-1188 | 173005 | rs4652676 | 0.78720332 | 0.15564913 | 4.2468E-07 |
| Feeling hurt \|\| id:ebi-a-GCST006951 | Major Depressive Disorder \|\| id:ieu-a-1188 | 173005 | rs4702 | 0.77401255 | 0.15153073 | 3.2566E-07 |
| Feeling hurt \|\| id:ebi-a-GCST006951 | Major Depressive Disorder \|\| id:ieu-a-1188 | 173005 | rs4791774 | 0.8297936 | 0.1625234 | 3.296E-07 |
| Feeling hurt \|\| id:ebi-a-GCST006951 | Major Depressive Disorder \|\| id:ieu-a-1188 | 173005 | rs4868774 | 0.85308356 | 0.15983965 | 9.4434E-08 |
| Feeling hurt \|\| id:ebi-a-GCST006951 | Major Depressive Disorder \|\| id:ieu-a-1188 | 173005 | rs545853 | 0.83564704 | 0.16226176 | 2.605E-07 |
| Feeling hurt \|\| id:ebi-a-GCST006951 | Major Depressive Disorder \|\| id:ieu-a-1188 | 173005 | rs55657917 | 0.85877115 | 0.17101435 | 5.1234E-07 |
| Feeling hurt \|\| id:ebi-a-GCST006951 | Major Depressive Disorder \|\| id:ieu-a-1188 | 173005 | rs62035176 | 0.87014536 | 0.15466374 | 1.8439E-08 |
| Feeling hurt \|\| id:ebi-a-GCST006951 | Major Depressive Disorder \|\| id:ieu-a-1188 | 173005 | rs73480560 | 0.77963397 | 0.15300494 | 3.4785E-07 |
| Feeling hurt \|\| id:ebi-a-GCST006951 | Major Depressive Disorder \|\| id:ieu-a-1188 | 173005 | All | 0.82726093 | 0.15651152 | 1.2528E-07 |
| Feeling tense \|\| id:ebi-a-GCST006952 | Major Depressive Disorder \|\| id:ieu-a-1188 | 173005 | rs10767733 | 0.70370571 | 0.15798374 | 8.4171E-06 |
| Feeling tense \|\| id:ebi-a-GCST006952 | Major Depressive Disorder \|\| id:ieu-a-1188 | 173005 | rs11090045 | 0.77569667 | 0.16136554 | 1.5315E-06 |
| Feeling tense \|\| id:ebi-a-GCST006952 | Major Depressive Disorder \|\| id:ieu-a-1188 | 173005 | rs1147851 | 0.75492987 | 0.16298398 | 3.6228E-06 |
| Feeling tense \|\| id:ebi-a-GCST006952 | Major Depressive Disorder \|\| id:ieu-a-1188 | 173005 | rs11509880 | 0.67675866 | 0.14595196 | 3.5374E-06 |
| Feeling tense \|\| id:ebi-a-GCST006952 | Major Depressive Disorder \|\| id:ieu-a-1188 | 173005 | rs1450832 | 0.7387724 | 0.16321982 | 6.0042E-06 |
| Feeling tense \|\| id:ebi-a-GCST006952 | Major Depressive Disorder \|\| id:ieu-a-1188 | 173005 | rs2097247 | 0.72675833 | 0.16116747 | 6.5031E-06 |
| Feeling tense \|\| id:ebi-a-GCST006952 | Major Depressive Disorder \|\| id:ieu-a-1188 | 173005 | rs28738966 | 0.75587541 | 0.16350105 | 3.7812E-06 |
| Feeling tense \|\| id:ebi-a-GCST006952 | Major Depressive Disorder \|\| id:ieu-a-1188 | 173005 | rs3751855 | 0.74131575 | 0.16260101 | 5.1371E-06 |
| Feeling tense \|\| id:ebi-a-GCST006952 | Major Depressive Disorder \|\| id:ieu-a-1188 | 173005 | rs4129585 | 0.70732563 | 0.15814665 | 7.7277E-06 |
| Feeling tense \|\| id:ebi-a-GCST006952 | Major Depressive Disorder \|\| id:ieu-a-1188 | 173005 | rs4671330 | 0.81889991 | 0.14676614 | 2.4104E-08 |
| Feeling tense \|\| id:ebi-a-GCST006952 | Major Depressive Disorder \|\| id:ieu-a-1188 | 173005 | rs4937872 | 0.69682842 | 0.15684514 | 8.8804E-06 |
| Feeling tense \|\| id:ebi-a-GCST006952 | Major Depressive Disorder \|\| id:ieu-a-1188 | 173005 | rs56084168 | 0.75517367 | 0.16156853 | 2.9537E-06 |
| Feeling tense \|\| id:ebi-a-GCST006952 | Major Depressive Disorder \|\| id:ieu-a-1188 | 173005 | rs7194615 | 0.8002154 | 0.15173459 | 1.3364E-07 |
| Feeling tense \|\| id:ebi-a-GCST006952 | Major Depressive Disorder \|\| id:ieu-a-1188 | 173005 | rs78379741 | 0.72953078 | 0.16269394 | 7.3233E-06 |
| Feeling tense \|\| id:ebi-a-GCST006952 | Major Depressive Disorder \|\| id:ieu-a-1188 | 173005 | rs79861172 | 0.80303381 | 0.14527133 | 3.2423E-08 |
| Feeling tense \|\| id:ebi-a-GCST006952 | Major Depressive Disorder \|\| id:ieu-a-1188 | 173005 | rs9527336 | 0.73885568 | 0.16284047 | 5.698E-06 |
| Feeling tense \|\| id:ebi-a-GCST006952 | Major Depressive Disorder \|\| id:ieu-a-1188 | 173005 | rs9811546 | 0.77586442 | 0.15976233 | 1.1956E-06 |
| Feeling tense \|\| id:ebi-a-GCST006952 | Major Depressive Disorder \|\| id:ieu-a-1188 | 173005 | All | 0.74715372 | 0.15330044 | 1.0948E-06 |
| Daytime nap \|\| id:ebi-a-GCST011494 | Major Depressive Disorder \|\| id:ieu-a-1188 | 173005 | rs1001817 | 0.4673047 | 0.13794263 | 0.00070488 |
| Daytime nap \|\| id:ebi-a-GCST011494 | Major Depressive Disorder \|\| id:ieu-a-1188 | 173005 | rs10149986 | 0.49684192 | 0.13728567 | 0.0002957 |
| Daytime nap \|\| id:ebi-a-GCST011494 | Major Depressive Disorder \|\| id:ieu-a-1188 | 173005 | rs10840017 | 0.46588328 | 0.13746303 | 0.00070109 |
| Daytime nap \|\| id:ebi-a-GCST011494 | Major Depressive Disorder \|\| id:ieu-a-1188 | 173005 | rs10875606 | 0.45852941 | 0.13620577 | 0.00076143 |
| Daytime nap \|\| id:ebi-a-GCST011494 | Major Depressive Disorder \|\| id:ieu-a-1188 | 173005 | rs10875622 | 0.46776541 | 0.1388128 | 0.00075234 |
| Daytime nap \|\| id:ebi-a-GCST011494 | Major Depressive Disorder \|\| id:ieu-a-1188 | 173005 | rs11071755 | 0.45607966 | 0.13594832 | 0.00079422 |
| Daytime nap \|\| id:ebi-a-GCST011494 | Major Depressive Disorder \|\| id:ieu-a-1188 | 173005 | rs11224896 | 0.47741861 | 0.1382035 | 0.00055139 |
| Daytime nap \|\| id:ebi-a-GCST011494 | Major Depressive Disorder \|\| id:ieu-a-1188 | 173005 | rs11258652 | 0.4754625 | 0.13862958 | 0.00060417 |
| Daytime nap \|\| id:ebi-a-GCST011494 | Major Depressive Disorder \|\| id:ieu-a-1188 | 173005 | rs11615756 | 0.51807856 | 0.14074944 | 0.00023245 |
| Daytime nap \|\| id:ebi-a-GCST011494 | Major Depressive Disorder \|\| id:ieu-a-1188 | 173005 | rs11682175 | 0.45376185 | 0.1353493 | 0.00080078 |
| Daytime nap \|\| id:ebi-a-GCST011494 | Major Depressive Disorder \|\| id:ieu-a-1188 | 173005 | rs11860072 | 0.48649585 | 0.13796869 | 0.00042168 |
| Daytime nap \|\| id:ebi-a-GCST011494 | Major Depressive Disorder \|\| id:ieu-a-1188 | 173005 | rs12042846 | 0.48065827 | 0.13814923 | 0.00050278 |
| Daytime nap \|\| id:ebi-a-GCST011494 | Major Depressive Disorder \|\| id:ieu-a-1188 | 173005 | rs12140153 | 0.4439098 | 0.13876869 | 0.00137944 |
| Daytime nap \|\| id:ebi-a-GCST011494 | Major Depressive Disorder \|\| id:ieu-a-1188 | 173005 | rs12346996 | 0.47222314 | 0.13810806 | 0.00062799 |
| Daytime nap \|\| id:ebi-a-GCST011494 | Major Depressive Disorder \|\| id:ieu-a-1188 | 173005 | rs12451365 | 0.48291547 | 0.13846305 | 0.00048722 |
| Daytime nap \|\| id:ebi-a-GCST011494 | Major Depressive Disorder \|\| id:ieu-a-1188 | 173005 | rs12657723 | 0.50661327 | 0.1348551 | 0.00017215 |
| Daytime nap \|\| id:ebi-a-GCST011494 | Major Depressive Disorder \|\| id:ieu-a-1188 | 173005 | rs12992648 | 0.48466288 | 0.13796657 | 0.00044324 |
| Daytime nap \|\| id:ebi-a-GCST011494 | Major Depressive Disorder \|\| id:ieu-a-1188 | 173005 | rs13033444 | 0.47629806 | 0.13859393 | 0.00058897 |
| Daytime nap \|\| id:ebi-a-GCST011494 | Major Depressive Disorder \|\| id:ieu-a-1188 | 173005 | rs13150944 | 0.47615425 | 0.13842523 | 0.00058216 |
| Daytime nap \|\| id:ebi-a-GCST011494 | Major Depressive Disorder \|\| id:ieu-a-1188 | 173005 | rs13263535 | 0.4951492 | 0.13655573 | 0.00028786 |
| Daytime nap \|\| id:ebi-a-GCST011494 | Major Depressive Disorder \|\| id:ieu-a-1188 | 173005 | rs13284688 | 0.46471957 | 0.13936771 | 0.00085457 |
| Daytime nap \|\| id:ebi-a-GCST011494 | Major Depressive Disorder \|\| id:ieu-a-1188 | 173005 | rs13330072 | 0.48753909 | 0.13776101 | 0.00040161 |
| Daytime nap \|\| id:ebi-a-GCST011494 | Major Depressive Disorder \|\| id:ieu-a-1188 | 173005 | rs1546977 | 0.47281223 | 0.13843442 | 0.00063683 |
| Daytime nap \|\| id:ebi-a-GCST011494 | Major Depressive Disorder \|\| id:ieu-a-1188 | 173005 | rs1601440 | 0.49810718 | 0.13683668 | 0.00027247 |
| Daytime nap \|\| id:ebi-a-GCST011494 | Major Depressive Disorder \|\| id:ieu-a-1188 | 173005 | rs17158413 | 0.47032894 | 0.13821663 | 0.0006669 |
| Daytime nap \|\| id:ebi-a-GCST011494 | Major Depressive Disorder \|\| id:ieu-a-1188 | 173005 | rs17265513 | 0.47436406 | 0.13826096 | 0.00060151 |
| Daytime nap \|\| id:ebi-a-GCST011494 | Major Depressive Disorder \|\| id:ieu-a-1188 | 173005 | rs174541 | 0.45263066 | 0.13705902 | 0.00095844 |
| Daytime nap \|\| id:ebi-a-GCST011494 | Major Depressive Disorder \|\| id:ieu-a-1188 | 173005 | rs17502738 | 0.47539228 | 0.1381804 | 0.00058091 |
| Daytime nap \|\| id:ebi-a-GCST011494 | Major Depressive Disorder \|\| id:ieu-a-1188 | 173005 | rs1883048 | 0.49519948 | 0.13703522 | 0.0003019 |
| Daytime nap \|\| id:ebi-a-GCST011494 | Major Depressive Disorder \|\| id:ieu-a-1188 | 173005 | rs2033103 | 0.48003912 | 0.13827349 | 0.00051724 |
| Daytime nap \|\| id:ebi-a-GCST011494 | Major Depressive Disorder \|\| id:ieu-a-1188 | 173005 | rs2059639 | 0.4809258 | 0.13742497 | 0.00046604 |
| Daytime nap \|\| id:ebi-a-GCST011494 | Major Depressive Disorder \|\| id:ieu-a-1188 | 173005 | rs2099810 | 0.49287771 | 0.13741444 | 0.00033476 |
| Daytime nap \|\| id:ebi-a-GCST011494 | Major Depressive Disorder \|\| id:ieu-a-1188 | 173005 | rs2143792 | 0.49062398 | 0.13734062 | 0.00035384 |
| Daytime nap \|\| id:ebi-a-GCST011494 | Major Depressive Disorder \|\| id:ieu-a-1188 | 173005 | rs224111 | 0.46267531 | 0.13710601 | 0.00073928 |
| Daytime nap \|\| id:ebi-a-GCST011494 | Major Depressive Disorder \|\| id:ieu-a-1188 | 173005 | rs2250377 | 0.45477361 | 0.13884178 | 0.00105482 |
| Daytime nap \|\| id:ebi-a-GCST011494 | Major Depressive Disorder \|\| id:ieu-a-1188 | 173005 | rs2370926 | 0.51163579 | 0.13370744 | 0.00012996 |
| Daytime nap \|\| id:ebi-a-GCST011494 | Major Depressive Disorder \|\| id:ieu-a-1188 | 173005 | rs2653349 | 0.4693413 | 0.14004176 | 0.00080393 |
| Daytime nap \|\| id:ebi-a-GCST011494 | Major Depressive Disorder \|\| id:ieu-a-1188 | 173005 | rs2699869 | 0.47196568 | 0.138046 | 0.00062874 |
| Daytime nap \|\| id:ebi-a-GCST011494 | Major Depressive Disorder \|\| id:ieu-a-1188 | 173005 | rs271057 | 0.46710432 | 0.13788192 | 0.00070478 |
| Daytime nap \|\| id:ebi-a-GCST011494 | Major Depressive Disorder \|\| id:ieu-a-1188 | 173005 | rs2769916 | 0.47895404 | 0.13845554 | 0.00054166 |
| Daytime nap \|\| id:ebi-a-GCST011494 | Major Depressive Disorder \|\| id:ieu-a-1188 | 173005 | rs2943023 | 0.46105544 | 0.13689361 | 0.0007572 |
| Daytime nap \|\| id:ebi-a-GCST011494 | Major Depressive Disorder \|\| id:ieu-a-1188 | 173005 | rs295278 | 0.48466084 | 0.13675791 | 0.00039421 |
| Daytime nap \|\| id:ebi-a-GCST011494 | Major Depressive Disorder \|\| id:ieu-a-1188 | 173005 | rs34262487 | 0.45271729 | 0.13572228 | 0.00085111 |
| Daytime nap \|\| id:ebi-a-GCST011494 | Major Depressive Disorder \|\| id:ieu-a-1188 | 173005 | rs35011311 | 0.48976362 | 0.13722743 | 0.00035836 |
| Daytime nap \|\| id:ebi-a-GCST011494 | Major Depressive Disorder \|\| id:ieu-a-1188 | 173005 | rs35039375 | 0.45980104 | 0.13708232 | 0.00079596 |
| Daytime nap \|\| id:ebi-a-GCST011494 | Major Depressive Disorder \|\| id:ieu-a-1188 | 173005 | rs350785 | 0.46055757 | 0.13737348 | 0.00080058 |
| Daytime nap \|\| id:ebi-a-GCST011494 | Major Depressive Disorder \|\| id:ieu-a-1188 | 173005 | rs351776 | 0.47258335 | 0.13825482 | 0.00063036 |
| Daytime nap \|\| id:ebi-a-GCST011494 | Major Depressive Disorder \|\| id:ieu-a-1188 | 173005 | rs35851551 | 0.47986247 | 0.138083 | 0.00051052 |
| Daytime nap \|\| id:ebi-a-GCST011494 | Major Depressive Disorder \|\| id:ieu-a-1188 | 173005 | rs378421 | 0.47704749 | 0.13802839 | 0.00054794 |
| Daytime nap \|\| id:ebi-a-GCST011494 | Major Depressive Disorder \|\| id:ieu-a-1188 | 173005 | rs3810484 | 0.49075991 | 0.13673992 | 0.00033195 |
| Daytime nap \|\| id:ebi-a-GCST011494 | Major Depressive Disorder \|\| id:ieu-a-1188 | 173005 | rs385199 | 0.50728605 | 0.13958223 | 0.00027872 |
| Daytime nap \|\| id:ebi-a-GCST011494 | Major Depressive Disorder \|\| id:ieu-a-1188 | 173005 | rs3935190 | 0.4915903 | 0.13735469 | 0.00034493 |
| Daytime nap \|\| id:ebi-a-GCST011494 | Major Depressive Disorder \|\| id:ieu-a-1188 | 173005 | rs4511908 | 0.46602936 | 0.13376475 | 0.00049408 |
| Daytime nap \|\| id:ebi-a-GCST011494 | Major Depressive Disorder \|\| id:ieu-a-1188 | 173005 | rs4604518 | 0.45469869 | 0.1355301 | 0.00079375 |
| Daytime nap \|\| id:ebi-a-GCST011494 | Major Depressive Disorder \|\| id:ieu-a-1188 | 173005 | rs467897 | 0.48990038 | 0.13814603 | 0.00039076 |
| Daytime nap \|\| id:ebi-a-GCST011494 | Major Depressive Disorder \|\| id:ieu-a-1188 | 173005 | rs60222088 | 0.47378409 | 0.1384503 | 0.0006215 |
| Daytime nap \|\| id:ebi-a-GCST011494 | Major Depressive Disorder \|\| id:ieu-a-1188 | 173005 | rs60920123 | 0.48988911 | 0.13770238 | 0.00037427 |
| Daytime nap \|\| id:ebi-a-GCST011494 | Major Depressive Disorder \|\| id:ieu-a-1188 | 173005 | rs614987 | 0.48322827 | 0.13908408 | 0.00051207 |
| Daytime nap \|\| id:ebi-a-GCST011494 | Major Depressive Disorder \|\| id:ieu-a-1188 | 173005 | rs62189006 | 0.47461148 | 0.13821479 | 0.00059503 |
| Daytime nap \|\| id:ebi-a-GCST011494 | Major Depressive Disorder \|\| id:ieu-a-1188 | 173005 | rs6452787 | 0.47668588 | 0.13775198 | 0.00053924 |
| Daytime nap \|\| id:ebi-a-GCST011494 | Major Depressive Disorder \|\| id:ieu-a-1188 | 173005 | rs6665690 | 0.49709589 | 0.13629377 | 0.00026507 |
| Daytime nap \|\| id:ebi-a-GCST011494 | Major Depressive Disorder \|\| id:ieu-a-1188 | 173005 | rs73817091 | 0.48266411 | 0.13797454 | 0.00046839 |
| Daytime nap \|\| id:ebi-a-GCST011494 | Major Depressive Disorder \|\| id:ieu-a-1188 | 173005 | rs7422655 | 0.46805492 | 0.13778605 | 0.00068137 |
| Daytime nap \|\| id:ebi-a-GCST011494 | Major Depressive Disorder \|\| id:ieu-a-1188 | 173005 | rs7423968 | 0.48185829 | 0.1382874 | 0.00049311 |
| Daytime nap \|\| id:ebi-a-GCST011494 | Major Depressive Disorder \|\| id:ieu-a-1188 | 173005 | rs75022160 | 0.50733771 | 0.13341606 | 0.00014314 |
| Daytime nap \|\| id:ebi-a-GCST011494 | Major Depressive Disorder \|\| id:ieu-a-1188 | 173005 | rs76257331 | 0.47386079 | 0.13816907 | 0.00060453 |
| Daytime nap \|\| id:ebi-a-GCST011494 | Major Depressive Disorder \|\| id:ieu-a-1188 | 173005 | rs76824303 | 0.46187766 | 0.13696539 | 0.00074564 |
| Daytime nap \|\| id:ebi-a-GCST011494 | Major Depressive Disorder \|\| id:ieu-a-1188 | 173005 | rs77154532 | 0.47563534 | 0.13829805 | 0.00058343 |
| Daytime nap \|\| id:ebi-a-GCST011494 | Major Depressive Disorder \|\| id:ieu-a-1188 | 173005 | rs7814873 | 0.4837764 | 0.13793142 | 0.00045256 |
| Daytime nap \|\| id:ebi-a-GCST011494 | Major Depressive Disorder \|\| id:ieu-a-1188 | 173005 | rs785145 | 0.46774156 | 0.13776689 | 0.00068587 |
| Daytime nap \|\| id:ebi-a-GCST011494 | Major Depressive Disorder \|\| id:ieu-a-1188 | 173005 | rs80163246 | 0.45754456 | 0.13653451 | 0.00080483 |
| Daytime nap \|\| id:ebi-a-GCST011494 | Major Depressive Disorder \|\| id:ieu-a-1188 | 173005 | rs9287862 | 0.45899677 | 0.13642488 | 0.00076692 |
| Daytime nap \|\| id:ebi-a-GCST011494 | Major Depressive Disorder \|\| id:ieu-a-1188 | 173005 | rs9309116 | 0.47266745 | 0.13813873 | 0.00062234 |
| Daytime nap \|\| id:ebi-a-GCST011494 | Major Depressive Disorder \|\| id:ieu-a-1188 | 173005 | rs936944 | 0.49388764 | 0.13660718 | 0.0002999 |
| Daytime nap \|\| id:ebi-a-GCST011494 | Major Depressive Disorder \|\| id:ieu-a-1188 | 173005 | rs9460110 | 0.48336041 | 0.13806515 | 0.00046359 |
| Daytime nap \|\| id:ebi-a-GCST011494 | Major Depressive Disorder \|\| id:ieu-a-1188 | 173005 | rs962247 | 0.46581639 | 0.13787865 | 0.00072895 |
| Daytime nap \|\| id:ebi-a-GCST011494 | Major Depressive Disorder \|\| id:ieu-a-1188 | 173005 | rs971415 | 0.49122122 | 0.13752272 | 0.00035436 |
| Daytime nap \|\| id:ebi-a-GCST011494 | Major Depressive Disorder \|\| id:ieu-a-1188 | 173005 | rs9939355 | 0.48684364 | 0.1378746 | 0.0004139 |
| Daytime nap \|\| id:ebi-a-GCST011494 | Major Depressive Disorder \|\| id:ieu-a-1188 | 173005 | rs9965170 | 0.48733615 | 0.13999794 | 0.0004995 |
| Daytime nap \|\| id:ebi-a-GCST011494 | Major Depressive Disorder \|\| id:ieu-a-1188 | 173005 | All | 0.47755598 | 0.13671745 | 0.0004776 |
| Years of schooling \|\| id:ieu-a-1239 | Major Depressive Disorder \|\| id:ieu-a-1188 | 173005 | rs10073890 | -0.3697545 | 0.05735982 | 1.1467E-10 |
| Years of schooling \|\| id:ieu-a-1239 | Major Depressive Disorder \|\| id:ieu-a-1188 | 173005 | rs1008078 | -0.3611219 | 0.0575239 | 3.4346E-10 |
| Years of schooling \|\| id:ieu-a-1239 | Major Depressive Disorder \|\| id:ieu-a-1188 | 173005 | rs10189857 | -0.3725804 | 0.05746523 | 8.9571E-11 |
| Years of schooling \|\| id:ieu-a-1239 | Major Depressive Disorder \|\| id:ieu-a-1188 | 173005 | rs10191758 | -0.3633447 | 0.05756179 | 2.75E-10 |
| Years of schooling \|\| id:ieu-a-1239 | Major Depressive Disorder \|\| id:ieu-a-1188 | 173005 | rs10205801 | -0.3649786 | 0.05745915 | 2.1258E-10 |
| Years of schooling \|\| id:ieu-a-1239 | Major Depressive Disorder \|\| id:ieu-a-1188 | 173005 | rs10215082 | -0.3623117 | 0.05741745 | 2.788E-10 |
| Years of schooling \|\| id:ieu-a-1239 | Major Depressive Disorder \|\| id:ieu-a-1188 | 173005 | rs10240905 | -0.3607916 | 0.05721906 | 2.8737E-10 |
| Years of schooling \|\| id:ieu-a-1239 | Major Depressive Disorder \|\| id:ieu-a-1188 | 173005 | rs10456918 | -0.3643565 | 0.05746042 | 2.2828E-10 |
| Years of schooling \|\| id:ieu-a-1239 | Major Depressive Disorder \|\| id:ieu-a-1188 | 173005 | rs10460095 | -0.3673605 | 0.05745982 | 1.6229E-10 |
| Years of schooling \|\| id:ieu-a-1239 | Major Depressive Disorder \|\| id:ieu-a-1188 | 173005 | rs1051474 | -0.3623623 | 0.05737297 | 2.6858E-10 |
| Years of schooling \|\| id:ieu-a-1239 | Major Depressive Disorder \|\| id:ieu-a-1188 | 173005 | rs10765775 | -0.363796 | 0.05753093 | 2.5573E-10 |
| Years of schooling \|\| id:ieu-a-1239 | Major Depressive Disorder \|\| id:ieu-a-1188 | 173005 | rs10798418 | -0.3627706 | 0.057305 | 2.4433E-10 |
| Years of schooling \|\| id:ieu-a-1239 | Major Depressive Disorder \|\| id:ieu-a-1188 | 173005 | rs10856785 | -0.364843 | 0.05744788 | 2.1412E-10 |
| Years of schooling \|\| id:ieu-a-1239 | Major Depressive Disorder \|\| id:ieu-a-1188 | 173005 | rs10887801 | -0.3687971 | 0.05741537 | 1.3334E-10 |
| Years of schooling \|\| id:ieu-a-1239 | Major Depressive Disorder \|\| id:ieu-a-1188 | 173005 | rs10940921 | -0.3653572 | 0.05747444 | 2.0591E-10 |
| Years of schooling \|\| id:ieu-a-1239 | Major Depressive Disorder \|\| id:ieu-a-1188 | 173005 | rs10994777 | -0.3656259 | 0.05747872 | 2.0036E-10 |
| Years of schooling \|\| id:ieu-a-1239 | Major Depressive Disorder \|\| id:ieu-a-1188 | 173005 | rs11023749 | -0.3706631 | 0.05719796 | 9.1507E-11 |
| Years of schooling \|\| id:ieu-a-1239 | Major Depressive Disorder \|\| id:ieu-a-1188 | 173005 | rs1105307 | -0.367876 | 0.0574413 | 1.5098E-10 |
| Years of schooling \|\| id:ieu-a-1239 | Major Depressive Disorder \|\| id:ieu-a-1188 | 173005 | rs1106090 | -0.3699094 | 0.0573635 | 1.1295E-10 |
| Years of schooling \|\| id:ieu-a-1239 | Major Depressive Disorder \|\| id:ieu-a-1188 | 173005 | rs11081529 | -0.3680391 | 0.05747166 | 1.5152E-10 |
| Years of schooling \|\| id:ieu-a-1239 | Major Depressive Disorder \|\| id:ieu-a-1188 | 173005 | rs11123818 | -0.3598395 | 0.05762174 | 4.2419E-10 |
| Years of schooling \|\| id:ieu-a-1239 | Major Depressive Disorder \|\| id:ieu-a-1188 | 173005 | rs111821073 | -0.3639489 | 0.05741026 | 2.306E-10 |
| Years of schooling \|\| id:ieu-a-1239 | Major Depressive Disorder \|\| id:ieu-a-1188 | 173005 | rs112687095 | -0.3641939 | 0.05737594 | 2.1884E-10 |
| Years of schooling \|\| id:ieu-a-1239 | Major Depressive Disorder \|\| id:ieu-a-1188 | 173005 | rs113182709 | -0.3687863 | 0.05729819 | 1.2245E-10 |
| Years of schooling \|\| id:ieu-a-1239 | Major Depressive Disorder \|\| id:ieu-a-1188 | 173005 | rs113520408 | -0.3696736 | 0.0573849 | 1.1791E-10 |
| Years of schooling \|\| id:ieu-a-1239 | Major Depressive Disorder \|\| id:ieu-a-1188 | 173005 | rs113615161 | -0.3667406 | 0.05746327 | 1.7459E-10 |
| Years of schooling \|\| id:ieu-a-1239 | Major Depressive Disorder \|\| id:ieu-a-1188 | 173005 | rs1143770 | -0.3646538 | 0.05746822 | 2.2197E-10 |
| Years of schooling \|\| id:ieu-a-1239 | Major Depressive Disorder \|\| id:ieu-a-1188 | 173005 | rs115454970 | -0.363256 | 0.05736542 | 2.4151E-10 |
| Years of schooling \|\| id:ieu-a-1239 | Major Depressive Disorder \|\| id:ieu-a-1188 | 173005 | rs11601122 | -0.3650024 | 0.05754467 | 2.2542E-10 |
| Years of schooling \|\| id:ieu-a-1239 | Major Depressive Disorder \|\| id:ieu-a-1188 | 173005 | rs11620355 | -0.3603032 | 0.05704279 | 2.6778E-10 |
| Years of schooling \|\| id:ieu-a-1239 | Major Depressive Disorder \|\| id:ieu-a-1188 | 173005 | rs11627087 | -0.3694123 | 0.05733111 | 1.1676E-10 |
| Years of schooling \|\| id:ieu-a-1239 | Major Depressive Disorder \|\| id:ieu-a-1188 | 173005 | rs11635092 | -0.3722939 | 0.05715742 | 7.3427E-11 |
| Years of schooling \|\| id:ieu-a-1239 | Major Depressive Disorder \|\| id:ieu-a-1188 | 173005 | rs11657342 | -0.3717464 | 0.05724731 | 8.3758E-11 |
| Years of schooling \|\| id:ieu-a-1239 | Major Depressive Disorder \|\| id:ieu-a-1188 | 173005 | rs11663602 | -0.3593145 | 0.05700015 | 2.9054E-10 |
| Years of schooling \|\| id:ieu-a-1239 | Major Depressive Disorder \|\| id:ieu-a-1188 | 173005 | rs11678980 | -0.3680248 | 0.05758681 | 1.6504E-10 |
| Years of schooling \|\| id:ieu-a-1239 | Major Depressive Disorder \|\| id:ieu-a-1188 | 173005 | rs11681861 | -0.3710122 | 0.05714359 | 8.4351E-11 |
| Years of schooling \|\| id:ieu-a-1239 | Major Depressive Disorder \|\| id:ieu-a-1188 | 173005 | rs11694904 | -0.3645585 | 0.05746104 | 2.2323E-10 |
| Years of schooling \|\| id:ieu-a-1239 | Major Depressive Disorder \|\| id:ieu-a-1188 | 173005 | rs11732657 | -0.3661757 | 0.0574626 | 1.8607E-10 |
| Years of schooling \|\| id:ieu-a-1239 | Major Depressive Disorder \|\| id:ieu-a-1188 | 173005 | rs117468730 | -0.3635254 | 0.0573777 | 2.3633E-10 |
| Years of schooling \|\| id:ieu-a-1239 | Major Depressive Disorder \|\| id:ieu-a-1188 | 173005 | rs11752914 | -0.3663207 | 0.05745861 | 1.825E-10 |
| Years of schooling \|\| id:ieu-a-1239 | Major Depressive Disorder \|\| id:ieu-a-1188 | 173005 | rs11772580 | -0.3670086 | 0.05745563 | 1.6841E-10 |
| Years of schooling \|\| id:ieu-a-1239 | Major Depressive Disorder \|\| id:ieu-a-1188 | 173005 | rs11871429 | -0.3646261 | 0.05748833 | 2.259E-10 |
| Years of schooling \|\| id:ieu-a-1239 | Major Depressive Disorder \|\| id:ieu-a-1188 | 173005 | rs12028010 | -0.3714794 | 0.05740092 | 9.6931E-11 |
| Years of schooling \|\| id:ieu-a-1239 | Major Depressive Disorder \|\| id:ieu-a-1188 | 173005 | rs12375949 | -0.3678246 | 0.05754172 | 1.634E-10 |
| Years of schooling \|\| id:ieu-a-1239 | Major Depressive Disorder \|\| id:ieu-a-1188 | 173005 | rs12468040 | -0.3602949 | 0.05733058 | 3.2888E-10 |
| Years of schooling \|\| id:ieu-a-1239 | Major Depressive Disorder \|\| id:ieu-a-1188 | 173005 | rs12503522 | -0.369748 | 0.05732272 | 1.1164E-10 |
| Years of schooling \|\| id:ieu-a-1239 | Major Depressive Disorder \|\| id:ieu-a-1188 | 173005 | rs12519073 | -0.3709965 | 0.05719776 | 8.8025E-11 |
| Years of schooling \|\| id:ieu-a-1239 | Major Depressive Disorder \|\| id:ieu-a-1188 | 173005 | rs12574281 | -0.3675045 | 0.05745545 | 1.5915E-10 |
| Years of schooling \|\| id:ieu-a-1239 | Major Depressive Disorder \|\| id:ieu-a-1188 | 173005 | rs12602286 | -0.3683893 | 0.05740721 | 1.3887E-10 |
| Years of schooling \|\| id:ieu-a-1239 | Major Depressive Disorder \|\| id:ieu-a-1188 | 173005 | rs12643771 | -0.3702696 | 0.05742948 | 1.138E-10 |
| Years of schooling \|\| id:ieu-a-1239 | Major Depressive Disorder \|\| id:ieu-a-1188 | 173005 | rs12682775 | -0.3704405 | 0.05724618 | 9.7351E-11 |
| Years of schooling \|\| id:ieu-a-1239 | Major Depressive Disorder \|\| id:ieu-a-1188 | 173005 | rs12804787 | -0.3605453 | 0.05698888 | 2.5064E-10 |
| Years of schooling \|\| id:ieu-a-1239 | Major Depressive Disorder \|\| id:ieu-a-1188 | 173005 | rs1291818 | -0.3696149 | 0.05735421 | 1.1604E-10 |
| Years of schooling \|\| id:ieu-a-1239 | Major Depressive Disorder \|\| id:ieu-a-1188 | 173005 | rs12940014 | -0.3665439 | 0.05745378 | 1.7731E-10 |
| Years of schooling \|\| id:ieu-a-1239 | Major Depressive Disorder \|\| id:ieu-a-1188 | 173005 | rs13010566 | -0.3624575 | 0.05733284 | 2.5822E-10 |
| Years of schooling \|\| id:ieu-a-1239 | Major Depressive Disorder \|\| id:ieu-a-1188 | 173005 | rs13029509 | -0.3646973 | 0.05745296 | 2.1848E-10 |
| Years of schooling \|\| id:ieu-a-1239 | Major Depressive Disorder \|\| id:ieu-a-1188 | 173005 | rs13090388 | -0.3595235 | 0.05788465 | 5.2637E-10 |
| Years of schooling \|\| id:ieu-a-1239 | Major Depressive Disorder \|\| id:ieu-a-1188 | 173005 | rs13141210 | -0.3614679 | 0.05739198 | 3.0106E-10 |
| Years of schooling \|\| id:ieu-a-1239 | Major Depressive Disorder \|\| id:ieu-a-1188 | 173005 | rs13145650 | -0.3621908 | 0.05730363 | 2.6063E-10 |
| Years of schooling \|\| id:ieu-a-1239 | Major Depressive Disorder \|\| id:ieu-a-1188 | 173005 | rs13422673 | -0.3704735 | 0.05735537 | 1.0521E-10 |
| Years of schooling \|\| id:ieu-a-1239 | Major Depressive Disorder \|\| id:ieu-a-1188 | 173005 | rs1363862 | -0.3706155 | 0.05724642 | 9.5417E-11 |
| Years of schooling \|\| id:ieu-a-1239 | Major Depressive Disorder \|\| id:ieu-a-1188 | 173005 | rs1381247 | -0.370431 | 0.05718048 | 9.2776E-11 |
| Years of schooling \|\| id:ieu-a-1239 | Major Depressive Disorder \|\| id:ieu-a-1188 | 173005 | rs1391438 | -0.3737307 | 0.05731005 | 6.9744E-11 |
| Years of schooling \|\| id:ieu-a-1239 | Major Depressive Disorder \|\| id:ieu-a-1188 | 173005 | rs1427298 | -0.3680388 | 0.05742612 | 1.4656E-10 |
| Years of schooling \|\| id:ieu-a-1239 | Major Depressive Disorder \|\| id:ieu-a-1188 | 173005 | rs1450782 | -0.3669941 | 0.05744808 | 1.6777E-10 |
| Years of schooling \|\| id:ieu-a-1239 | Major Depressive Disorder \|\| id:ieu-a-1188 | 173005 | rs152603 | -0.3681945 | 0.05741224 | 1.4252E-10 |
| Years of schooling \|\| id:ieu-a-1239 | Major Depressive Disorder \|\| id:ieu-a-1188 | 173005 | rs1558727 | -0.3680329 | 0.05744493 | 1.4869E-10 |
| Years of schooling \|\| id:ieu-a-1239 | Major Depressive Disorder \|\| id:ieu-a-1188 | 173005 | rs1566085 | -0.3736721 | 0.0573228 | 7.0903E-11 |
| Years of schooling \|\| id:ieu-a-1239 | Major Depressive Disorder \|\| id:ieu-a-1188 | 173005 | rs1569092 | -0.3605019 | 0.05727374 | 3.0866E-10 |
| Years of schooling \|\| id:ieu-a-1239 | Major Depressive Disorder \|\| id:ieu-a-1188 | 173005 | rs1584469 | -0.3614931 | 0.05732392 | 2.8607E-10 |
| Years of schooling \|\| id:ieu-a-1239 | Major Depressive Disorder \|\| id:ieu-a-1188 | 173005 | rs1595973 | -0.3662865 | 0.05746405 | 1.8394E-10 |
| Years of schooling \|\| id:ieu-a-1239 | Major Depressive Disorder \|\| id:ieu-a-1188 | 173005 | rs1618725 | -0.3759657 | 0.05687034 | 3.8192E-11 |
| Years of schooling \|\| id:ieu-a-1239 | Major Depressive Disorder \|\| id:ieu-a-1188 | 173005 | rs1671770 | -0.3676949 | 0.05744995 | 1.5511E-10 |
| Years of schooling \|\| id:ieu-a-1239 | Major Depressive Disorder \|\| id:ieu-a-1188 | 173005 | rs16846463 | -0.3653671 | 0.05753664 | 2.1508E-10 |
| Years of schooling \|\| id:ieu-a-1239 | Major Depressive Disorder \|\| id:ieu-a-1188 | 173005 | rs16854920 | -0.3625279 | 0.05726535 | 2.441E-10 |
| Years of schooling \|\| id:ieu-a-1239 | Major Depressive Disorder \|\| id:ieu-a-1188 | 173005 | rs16995054 | -0.3653794 | 0.05749107 | 2.0787E-10 |
| Years of schooling \|\| id:ieu-a-1239 | Major Depressive Disorder \|\| id:ieu-a-1188 | 173005 | rs17048855 | -0.3626192 | 0.05737317 | 2.6095E-10 |
| Years of schooling \|\| id:ieu-a-1239 | Major Depressive Disorder \|\| id:ieu-a-1188 | 173005 | rs17110109 | -0.3668912 | 0.0574617 | 1.7143E-10 |
| Years of schooling \|\| id:ieu-a-1239 | Major Depressive Disorder \|\| id:ieu-a-1188 | 173005 | rs17126938 | -0.3691867 | 0.05738216 | 1.2443E-10 |
| Years of schooling \|\| id:ieu-a-1239 | Major Depressive Disorder \|\| id:ieu-a-1188 | 173005 | rs17425572 | -0.3639544 | 0.05747158 | 2.408E-10 |
| Years of schooling \|\| id:ieu-a-1239 | Major Depressive Disorder \|\| id:ieu-a-1188 | 173005 | rs17489649 | -0.3646025 | 0.05751148 | 2.3029E-10 |
| Years of schooling \|\| id:ieu-a-1239 | Major Depressive Disorder \|\| id:ieu-a-1188 | 173005 | rs17551064 | -0.3602327 | 0.05713366 | 2.8803E-10 |
| Years of schooling \|\| id:ieu-a-1239 | Major Depressive Disorder \|\| id:ieu-a-1188 | 173005 | rs17563464 | -0.3725782 | 0.05710613 | 6.8314E-11 |
| Years of schooling \|\| id:ieu-a-1239 | Major Depressive Disorder \|\| id:ieu-a-1188 | 173005 | rs17565975 | -0.3678012 | 0.057467 | 1.5516E-10 |
| Years of schooling \|\| id:ieu-a-1239 | Major Depressive Disorder \|\| id:ieu-a-1188 | 173005 | rs17598675 | -0.3657498 | 0.05750391 | 2.0119E-10 |
| Years of schooling \|\| id:ieu-a-1239 | Major Depressive Disorder \|\| id:ieu-a-1188 | 173005 | rs176218 | -0.3667765 | 0.05757007 | 1.8785E-10 |
| Years of schooling \|\| id:ieu-a-1239 | Major Depressive Disorder \|\| id:ieu-a-1188 | 173005 | rs1827540 | -0.3664281 | 0.05747796 | 1.8284E-10 |
| Years of schooling \|\| id:ieu-a-1239 | Major Depressive Disorder \|\| id:ieu-a-1188 | 173005 | rs1866823 | -0.3677224 | 0.05743648 | 1.5311E-10 |
| Years of schooling \|\| id:ieu-a-1239 | Major Depressive Disorder \|\| id:ieu-a-1188 | 173005 | rs192436652 | -0.3675944 | 0.05742668 | 1.5425E-10 |
| Years of schooling \|\| id:ieu-a-1239 | Major Depressive Disorder \|\| id:ieu-a-1188 | 173005 | rs1925576 | -0.3686267 | 0.05739275 | 1.3372E-10 |
| Years of schooling \|\| id:ieu-a-1239 | Major Depressive Disorder \|\| id:ieu-a-1188 | 173005 | rs1947114 | -0.3641026 | 0.05740034 | 2.2502E-10 |
| Years of schooling \|\| id:ieu-a-1239 | Major Depressive Disorder \|\| id:ieu-a-1188 | 173005 | rs1964927 | -0.3643767 | 0.05752288 | 2.3817E-10 |
| Years of schooling \|\| id:ieu-a-1239 | Major Depressive Disorder \|\| id:ieu-a-1188 | 173005 | rs2052285 | -0.3615346 | 0.05726469 | 2.7298E-10 |
| Years of schooling \|\| id:ieu-a-1239 | Major Depressive Disorder \|\| id:ieu-a-1188 | 173005 | rs2067854 | -0.3684526 | 0.05745761 | 1.4306E-10 |
| Years of schooling \|\| id:ieu-a-1239 | Major Depressive Disorder \|\| id:ieu-a-1188 | 173005 | rs2179152 | -0.3630586 | 0.05749777 | 2.7137E-10 |
| Years of schooling \|\| id:ieu-a-1239 | Major Depressive Disorder \|\| id:ieu-a-1188 | 173005 | rs2182505 | -0.3682336 | 0.05741163 | 1.4183E-10 |
| Years of schooling \|\| id:ieu-a-1239 | Major Depressive Disorder \|\| id:ieu-a-1188 | 173005 | rs225291 | -0.3631296 | 0.0573426 | 2.4101E-10 |
| Years of schooling \|\| id:ieu-a-1239 | Major Depressive Disorder \|\| id:ieu-a-1188 | 173005 | rs2256965 | -0.3708212 | 0.05727623 | 9.5278E-11 |
| Years of schooling \|\| id:ieu-a-1239 | Major Depressive Disorder \|\| id:ieu-a-1188 | 173005 | rs2283076 | -0.3647384 | 0.05742822 | 2.1364E-10 |
| Years of schooling \|\| id:ieu-a-1239 | Major Depressive Disorder \|\| id:ieu-a-1188 | 173005 | rs2287838 | -0.3684641 | 0.05744579 | 1.4164E-10 |
| Years of schooling \|\| id:ieu-a-1239 | Major Depressive Disorder \|\| id:ieu-a-1188 | 173005 | rs2302761 | -0.3598897 | 0.05709136 | 2.9053E-10 |
| Years of schooling \|\| id:ieu-a-1239 | Major Depressive Disorder \|\| id:ieu-a-1188 | 173005 | rs2347526 | -0.3609997 | 0.0573457 | 3.0711E-10 |
| Years of schooling \|\| id:ieu-a-1239 | Major Depressive Disorder \|\| id:ieu-a-1188 | 173005 | rs242093 | -0.3667306 | 0.05746548 | 1.7507E-10 |
| Years of schooling \|\| id:ieu-a-1239 | Major Depressive Disorder \|\| id:ieu-a-1188 | 173005 | rs2554835 | -0.3699778 | 0.05725819 | 1.0362E-10 |
| Years of schooling \|\| id:ieu-a-1239 | Major Depressive Disorder \|\| id:ieu-a-1188 | 173005 | rs2570497 | -0.3669538 | 0.05749521 | 1.7441E-10 |
| Years of schooling \|\| id:ieu-a-1239 | Major Depressive Disorder \|\| id:ieu-a-1188 | 173005 | rs2725370 | -0.3652339 | 0.05753962 | 2.1881E-10 |
| Years of schooling \|\| id:ieu-a-1239 | Major Depressive Disorder \|\| id:ieu-a-1188 | 173005 | rs277828 | -0.3666972 | 0.05745234 | 1.7407E-10 |
| Years of schooling \|\| id:ieu-a-1239 | Major Depressive Disorder \|\| id:ieu-a-1188 | 173005 | rs2787101 | -0.3632385 | 0.05734396 | 2.383E-10 |
| Years of schooling \|\| id:ieu-a-1239 | Major Depressive Disorder \|\| id:ieu-a-1188 | 173005 | rs2819336 | -0.3661276 | 0.05764843 | 2.1386E-10 |
| Years of schooling \|\| id:ieu-a-1239 | Major Depressive Disorder \|\| id:ieu-a-1188 | 173005 | rs2820314 | -0.3630073 | 0.05736287 | 2.4794E-10 |
| Years of schooling \|\| id:ieu-a-1239 | Major Depressive Disorder \|\| id:ieu-a-1188 | 173005 | rs28513670 | -0.3628631 | 0.0573869 | 2.5637E-10 |
| Years of schooling \|\| id:ieu-a-1239 | Major Depressive Disorder \|\| id:ieu-a-1188 | 173005 | rs2885198 | -0.3627479 | 0.05732864 | 2.4914E-10 |
| Years of schooling \|\| id:ieu-a-1239 | Major Depressive Disorder \|\| id:ieu-a-1188 | 173005 | rs2901616 | -0.3615844 | 0.05716694 | 2.5313E-10 |
| Years of schooling \|\| id:ieu-a-1239 | Major Depressive Disorder \|\| id:ieu-a-1188 | 173005 | rs2905426 | -0.3697303 | 0.05731341 | 1.111E-10 |
| Years of schooling \|\| id:ieu-a-1239 | Major Depressive Disorder \|\| id:ieu-a-1188 | 173005 | rs2971970 | -0.3684912 | 0.05750319 | 1.4726E-10 |
| Years of schooling \|\| id:ieu-a-1239 | Major Depressive Disorder \|\| id:ieu-a-1188 | 173005 | rs2998315 | -0.3630006 | 0.05743561 | 2.6134E-10 |
| Years of schooling \|\| id:ieu-a-1239 | Major Depressive Disorder \|\| id:ieu-a-1188 | 173005 | rs3013014 | -0.36217 | 0.0572772 | 2.5635E-10 |
| Years of schooling \|\| id:ieu-a-1239 | Major Depressive Disorder \|\| id:ieu-a-1188 | 173005 | rs301800 | -0.3690879 | 0.05742091 | 1.295E-10 |
| Years of schooling \|\| id:ieu-a-1239 | Major Depressive Disorder \|\| id:ieu-a-1188 | 173005 | rs3026996 | -0.3654805 | 0.05752175 | 2.1009E-10 |
| Years of schooling \|\| id:ieu-a-1239 | Major Depressive Disorder \|\| id:ieu-a-1188 | 173005 | rs31940 | -0.3662623 | 0.05747914 | 1.8647E-10 |
| Years of schooling \|\| id:ieu-a-1239 | Major Depressive Disorder \|\| id:ieu-a-1188 | 173005 | rs337637 | -0.3635224 | 0.05740715 | 2.4145E-10 |
| Years of schooling \|\| id:ieu-a-1239 | Major Depressive Disorder \|\| id:ieu-a-1188 | 173005 | rs34394051 | -0.3641825 | 0.05742318 | 2.2668E-10 |
| Years of schooling \|\| id:ieu-a-1239 | Major Depressive Disorder \|\| id:ieu-a-1188 | 173005 | rs34485537 | -0.3634919 | 0.05739461 | 2.4012E-10 |
| Years of schooling \|\| id:ieu-a-1239 | Major Depressive Disorder \|\| id:ieu-a-1188 | 173005 | rs35039375 | -0.3623617 | 0.05735202 | 2.6462E-10 |
| Years of schooling \|\| id:ieu-a-1239 | Major Depressive Disorder \|\| id:ieu-a-1188 | 173005 | rs35309068 | -0.3734031 | 0.05717907 | 6.5587E-11 |
| Years of schooling \|\| id:ieu-a-1239 | Major Depressive Disorder \|\| id:ieu-a-1188 | 173005 | rs35316276 | -0.3703213 | 0.05725758 | 9.9547E-11 |
| Years of schooling \|\| id:ieu-a-1239 | Major Depressive Disorder \|\| id:ieu-a-1188 | 173005 | rs35417702 | -0.3645418 | 0.05754604 | 2.3767E-10 |
| Years of schooling \|\| id:ieu-a-1239 | Major Depressive Disorder \|\| id:ieu-a-1188 | 173005 | rs35475880 | -0.3666017 | 0.057508 | 1.8321E-10 |
| Years of schooling \|\| id:ieu-a-1239 | Major Depressive Disorder \|\| id:ieu-a-1188 | 173005 | rs36083520 | -0.3634258 | 0.05744778 | 2.5128E-10 |
| Years of schooling \|\| id:ieu-a-1239 | Major Depressive Disorder \|\| id:ieu-a-1188 | 173005 | rs36119825 | -0.3631126 | 0.05738073 | 2.4816E-10 |
| Years of schooling \|\| id:ieu-a-1239 | Major Depressive Disorder \|\| id:ieu-a-1188 | 173005 | rs363096 | -0.3613842 | 0.05738767 | 3.0298E-10 |
| Years of schooling \|\| id:ieu-a-1239 | Major Depressive Disorder \|\| id:ieu-a-1188 | 173005 | rs3788556 | -0.3649762 | 0.05744838 | 2.11E-10 |
| Years of schooling \|\| id:ieu-a-1239 | Major Depressive Disorder \|\| id:ieu-a-1188 | 173005 | rs3809634 | -0.3681662 | 0.05741419 | 1.4319E-10 |
| Years of schooling \|\| id:ieu-a-1239 | Major Depressive Disorder \|\| id:ieu-a-1188 | 173005 | rs3890802 | -0.3678535 | 0.05743918 | 1.5113E-10 |
| Years of schooling \|\| id:ieu-a-1239 | Major Depressive Disorder \|\| id:ieu-a-1188 | 173005 | rs3897821 | -0.3651119 | 0.0575465 | 2.2295E-10 |
| Years of schooling \|\| id:ieu-a-1239 | Major Depressive Disorder \|\| id:ieu-a-1188 | 173005 | rs4073894 | -0.3654017 | 0.05749896 | 2.0852E-10 |
| Years of schooling \|\| id:ieu-a-1239 | Major Depressive Disorder \|\| id:ieu-a-1188 | 173005 | rs4328757 | -0.3666156 | 0.05746857 | 1.7777E-10 |
| Years of schooling \|\| id:ieu-a-1239 | Major Depressive Disorder \|\| id:ieu-a-1188 | 173005 | rs4352658 | -0.3639532 | 0.05744836 | 2.3686E-10 |
| Years of schooling \|\| id:ieu-a-1239 | Major Depressive Disorder \|\| id:ieu-a-1188 | 173005 | rs4369924 | -0.362438 | 0.05721308 | 2.3753E-10 |
| Years of schooling \|\| id:ieu-a-1239 | Major Depressive Disorder \|\| id:ieu-a-1188 | 173005 | rs4382592 | -0.3627202 | 0.05751942 | 2.8625E-10 |
| Years of schooling \|\| id:ieu-a-1239 | Major Depressive Disorder \|\| id:ieu-a-1188 | 173005 | rs4384309 | -0.3696464 | 0.05734309 | 1.1467E-10 |
| Years of schooling \|\| id:ieu-a-1239 | Major Depressive Disorder \|\| id:ieu-a-1188 | 173005 | rs4392737 | -0.3636389 | 0.05737776 | 2.3333E-10 |
| Years of schooling \|\| id:ieu-a-1239 | Major Depressive Disorder \|\| id:ieu-a-1188 | 173005 | rs4442732 | -0.3683777 | 0.05742159 | 1.4053E-10 |
| Years of schooling \|\| id:ieu-a-1239 | Major Depressive Disorder \|\| id:ieu-a-1188 | 173005 | rs4667025 | -0.368459 | 0.05739049 | 1.3609E-10 |
| Years of schooling \|\| id:ieu-a-1239 | Major Depressive Disorder \|\| id:ieu-a-1188 | 173005 | rs4700393 | -0.3734744 | 0.05761312 | 9.0242E-11 |
| Years of schooling \|\| id:ieu-a-1239 | Major Depressive Disorder \|\| id:ieu-a-1188 | 173005 | rs4726070 | -0.3657779 | 0.05751176 | 2.0168E-10 |
| Years of schooling \|\| id:ieu-a-1239 | Major Depressive Disorder \|\| id:ieu-a-1188 | 173005 | rs4778058 | -0.3666797 | 0.05746369 | 1.7586E-10 |
| Years of schooling \|\| id:ieu-a-1239 | Major Depressive Disorder \|\| id:ieu-a-1188 | 173005 | rs4787457 | -0.3720672 | 0.05747878 | 9.5998E-11 |
| Years of schooling \|\| id:ieu-a-1239 | Major Depressive Disorder \|\| id:ieu-a-1188 | 173005 | rs4810227 | -0.3692982 | 0.0574348 | 1.2772E-10 |
| Years of schooling \|\| id:ieu-a-1239 | Major Depressive Disorder \|\| id:ieu-a-1188 | 173005 | rs4839155 | -0.3626611 | 0.0573495 | 2.5537E-10 |
| Years of schooling \|\| id:ieu-a-1239 | Major Depressive Disorder \|\| id:ieu-a-1188 | 173005 | rs4846724 | -0.3664628 | 0.05747023 | 1.811E-10 |
| Years of schooling \|\| id:ieu-a-1239 | Major Depressive Disorder \|\| id:ieu-a-1188 | 173005 | rs4888746 | -0.3678855 | 0.05741383 | 1.4782E-10 |
| Years of schooling \|\| id:ieu-a-1239 | Major Depressive Disorder \|\| id:ieu-a-1188 | 173005 | rs4904523 | -0.3690298 | 0.05734831 | 1.2357E-10 |
| Years of schooling \|\| id:ieu-a-1239 | Major Depressive Disorder \|\| id:ieu-a-1188 | 173005 | rs4945424 | -0.3617042 | 0.05720942 | 2.5743E-10 |
| Years of schooling \|\| id:ieu-a-1239 | Major Depressive Disorder \|\| id:ieu-a-1188 | 173005 | rs4964046 | -0.3696885 | 0.05732964 | 1.1298E-10 |
| Years of schooling \|\| id:ieu-a-1239 | Major Depressive Disorder \|\| id:ieu-a-1188 | 173005 | rs4972400 | -0.3702645 | 0.05731094 | 1.0427E-10 |
| Years of schooling \|\| id:ieu-a-1239 | Major Depressive Disorder \|\| id:ieu-a-1188 | 173005 | rs4984541 | -0.366941 | 0.05746203 | 1.705E-10 |
| Years of schooling \|\| id:ieu-a-1239 | Major Depressive Disorder \|\| id:ieu-a-1188 | 173005 | rs535307 | -0.366817 | 0.0574479 | 1.7116E-10 |
| Years of schooling \|\| id:ieu-a-1239 | Major Depressive Disorder \|\| id:ieu-a-1188 | 173005 | rs56391344 | -0.3647165 | 0.05752429 | 2.2944E-10 |
| Years of schooling \|\| id:ieu-a-1239 | Major Depressive Disorder \|\| id:ieu-a-1188 | 173005 | rs575113 | -0.3740162 | 0.05695062 | 5.1209E-11 |
| Years of schooling \|\| id:ieu-a-1239 | Major Depressive Disorder \|\| id:ieu-a-1188 | 173005 | rs59123361 | -0.3649453 | 0.05748898 | 2.18E-10 |
| Years of schooling \|\| id:ieu-a-1239 | Major Depressive Disorder \|\| id:ieu-a-1188 | 173005 | rs6122735 | -0.3708307 | 0.05722896 | 9.186E-11 |
| Years of schooling \|\| id:ieu-a-1239 | Major Depressive Disorder \|\| id:ieu-a-1188 | 173005 | rs6123924 | -0.366826 | 0.05747952 | 1.7496E-10 |
| Years of schooling \|\| id:ieu-a-1239 | Major Depressive Disorder \|\| id:ieu-a-1188 | 173005 | rs613872 | -0.3577275 | 0.05703106 | 3.5529E-10 |
| Years of schooling \|\| id:ieu-a-1239 | Major Depressive Disorder \|\| id:ieu-a-1188 | 173005 | rs62097985 | -0.3569452 | 0.05689639 | 3.5279E-10 |
| Years of schooling \|\| id:ieu-a-1239 | Major Depressive Disorder \|\| id:ieu-a-1188 | 173005 | rs62157915 | -0.3654281 | 0.05746614 | 2.0305E-10 |
| Years of schooling \|\| id:ieu-a-1239 | Major Depressive Disorder \|\| id:ieu-a-1188 | 173005 | rs62183776 | -0.3688883 | 0.05738618 | 1.2915E-10 |
| Years of schooling \|\| id:ieu-a-1239 | Major Depressive Disorder \|\| id:ieu-a-1188 | 173005 | rs62184480 | -0.3605107 | 0.05733366 | 3.217E-10 |
| Years of schooling \|\| id:ieu-a-1239 | Major Depressive Disorder \|\| id:ieu-a-1188 | 173005 | rs622169 | -0.369993 | 0.05716695 | 9.6619E-11 |
| Years of schooling \|\| id:ieu-a-1239 | Major Depressive Disorder \|\| id:ieu-a-1188 | 173005 | rs62439690 | -0.3675712 | 0.05742667 | 1.5466E-10 |
| Years of schooling \|\| id:ieu-a-1239 | Major Depressive Disorder \|\| id:ieu-a-1188 | 173005 | rs62444881 | -0.3568242 | 0.0570413 | 3.9614E-10 |
| Years of schooling \|\| id:ieu-a-1239 | Major Depressive Disorder \|\| id:ieu-a-1188 | 173005 | rs6493265 | -0.3576735 | 0.05708512 | 3.7135E-10 |
| Years of schooling \|\| id:ieu-a-1239 | Major Depressive Disorder \|\| id:ieu-a-1188 | 173005 | rs6513959 | -0.3620771 | 0.0573123 | 2.6563E-10 |
| Years of schooling \|\| id:ieu-a-1239 | Major Depressive Disorder \|\| id:ieu-a-1188 | 173005 | rs6557171 | -0.3556524 | 0.05696457 | 4.2817E-10 |
| Years of schooling \|\| id:ieu-a-1239 | Major Depressive Disorder \|\| id:ieu-a-1188 | 173005 | rs66568921 | -0.374111 | 0.05723827 | 6.3173E-11 |
| Years of schooling \|\| id:ieu-a-1239 | Major Depressive Disorder \|\| id:ieu-a-1188 | 173005 | rs6731373 | -0.3678625 | 0.05747465 | 1.5494E-10 |
| Years of schooling \|\| id:ieu-a-1239 | Major Depressive Disorder \|\| id:ieu-a-1188 | 173005 | rs67885444 | -0.3675306 | 0.05744871 | 1.579E-10 |
| Years of schooling \|\| id:ieu-a-1239 | Major Depressive Disorder \|\| id:ieu-a-1188 | 173005 | rs67890737 | -0.3662067 | 0.05748335 | 1.8822E-10 |
| Years of schooling \|\| id:ieu-a-1239 | Major Depressive Disorder \|\| id:ieu-a-1188 | 173005 | rs6803651 | -0.3701647 | 0.05733285 | 1.0721E-10 |
| Years of schooling \|\| id:ieu-a-1239 | Major Depressive Disorder \|\| id:ieu-a-1188 | 173005 | rs6805241 | -0.3715004 | 0.05725888 | 8.6936E-11 |
| Years of schooling \|\| id:ieu-a-1239 | Major Depressive Disorder \|\| id:ieu-a-1188 | 173005 | rs6938002 | -0.3623873 | 0.0572892 | 2.5229E-10 |
| Years of schooling \|\| id:ieu-a-1239 | Major Depressive Disorder \|\| id:ieu-a-1188 | 173005 | rs6959891 | -0.3655182 | 0.05746643 | 2.0103E-10 |
| Years of schooling \|\| id:ieu-a-1239 | Major Depressive Disorder \|\| id:ieu-a-1188 | 173005 | rs7012546 | -0.3672935 | 0.05745277 | 1.627E-10 |
| Years of schooling \|\| id:ieu-a-1239 | Major Depressive Disorder \|\| id:ieu-a-1188 | 173005 | rs702606 | -0.3679456 | 0.05741845 | 1.473E-10 |
| Years of schooling \|\| id:ieu-a-1239 | Major Depressive Disorder \|\| id:ieu-a-1188 | 173005 | rs7031698 | -0.3594246 | 0.05697269 | 2.8136E-10 |
| Years of schooling \|\| id:ieu-a-1239 | Major Depressive Disorder \|\| id:ieu-a-1188 | 173005 | rs710629 | -0.3658578 | 0.05746716 | 1.9354E-10 |
| Years of schooling \|\| id:ieu-a-1239 | Major Depressive Disorder \|\| id:ieu-a-1188 | 173005 | rs71646142 | -0.3652859 | 0.05745595 | 2.0484E-10 |
| Years of schooling \|\| id:ieu-a-1239 | Major Depressive Disorder \|\| id:ieu-a-1188 | 173005 | rs7233920 | -0.3665456 | 0.05748568 | 1.8142E-10 |
| Years of schooling \|\| id:ieu-a-1239 | Major Depressive Disorder \|\| id:ieu-a-1188 | 173005 | rs7257460 | -0.3628065 | 0.05734556 | 2.505E-10 |
| Years of schooling \|\| id:ieu-a-1239 | Major Depressive Disorder \|\| id:ieu-a-1188 | 173005 | rs72807818 | -0.3642745 | 0.05750577 | 2.38E-10 |
| Years of schooling \|\| id:ieu-a-1239 | Major Depressive Disorder \|\| id:ieu-a-1188 | 173005 | rs72828517 | -0.3655579 | 0.05754658 | 2.12E-10 |
| Years of schooling \|\| id:ieu-a-1239 | Major Depressive Disorder \|\| id:ieu-a-1188 | 173005 | rs72840994 | -0.3692149 | 0.05734988 | 1.2111E-10 |
| Years of schooling \|\| id:ieu-a-1239 | Major Depressive Disorder \|\| id:ieu-a-1188 | 173005 | rs730384 | -0.3672498 | 0.05745526 | 1.6381E-10 |
| Years of schooling \|\| id:ieu-a-1239 | Major Depressive Disorder \|\| id:ieu-a-1188 | 173005 | rs7321274 | -0.3667784 | 0.05747084 | 1.748E-10 |
| Years of schooling \|\| id:ieu-a-1239 | Major Depressive Disorder \|\| id:ieu-a-1188 | 173005 | rs73301698 | -0.3647644 | 0.05745558 | 2.1724E-10 |
| Years of schooling \|\| id:ieu-a-1239 | Major Depressive Disorder \|\| id:ieu-a-1188 | 173005 | rs7332724 | -0.3635698 | 0.0573978 | 2.3856E-10 |
| Years of schooling \|\| id:ieu-a-1239 | Major Depressive Disorder \|\| id:ieu-a-1188 | 173005 | rs73344830 | -0.3687114 | 0.05759042 | 1.5305E-10 |
| Years of schooling \|\| id:ieu-a-1239 | Major Depressive Disorder \|\| id:ieu-a-1188 | 173005 | rs736282 | -0.3651097 | 0.05747102 | 2.1124E-10 |
| Years of schooling \|\| id:ieu-a-1239 | Major Depressive Disorder \|\| id:ieu-a-1188 | 173005 | rs73874335 | -0.3717417 | 0.05705172 | 7.2268E-11 |
| Years of schooling \|\| id:ieu-a-1239 | Major Depressive Disorder \|\| id:ieu-a-1188 | 173005 | rs743316 | -0.3602571 | 0.05701607 | 2.6408E-10 |
| Years of schooling \|\| id:ieu-a-1239 | Major Depressive Disorder \|\| id:ieu-a-1188 | 173005 | rs74643044 | -0.3641424 | 0.05738724 | 2.2191E-10 |
| Years of schooling \|\| id:ieu-a-1239 | Major Depressive Disorder \|\| id:ieu-a-1188 | 173005 | rs74701752 | -0.3673903 | 0.0574411 | 1.5955E-10 |
| Years of schooling \|\| id:ieu-a-1239 | Major Depressive Disorder \|\| id:ieu-a-1188 | 173005 | rs7481514 | -0.3688781 | 0.05738927 | 1.2959E-10 |
| Years of schooling \|\| id:ieu-a-1239 | Major Depressive Disorder \|\| id:ieu-a-1188 | 173005 | rs74998289 | -0.3664834 | 0.05751567 | 1.8671E-10 |
| Years of schooling \|\| id:ieu-a-1239 | Major Depressive Disorder \|\| id:ieu-a-1188 | 173005 | rs7594904 | -0.3637233 | 0.05738288 | 2.3196E-10 |
| Years of schooling \|\| id:ieu-a-1239 | Major Depressive Disorder \|\| id:ieu-a-1188 | 173005 | rs7603132 | -0.3673622 | 0.05745994 | 1.6228E-10 |
| Years of schooling \|\| id:ieu-a-1239 | Major Depressive Disorder \|\| id:ieu-a-1188 | 173005 | rs76076331 | -0.3647717 | 0.05749957 | 2.2402E-10 |
| Years of schooling \|\| id:ieu-a-1239 | Major Depressive Disorder \|\| id:ieu-a-1188 | 173005 | rs7650602 | -0.36569 | 0.05745083 | 1.9495E-10 |
| Years of schooling \|\| id:ieu-a-1239 | Major Depressive Disorder \|\| id:ieu-a-1188 | 173005 | rs76608582 | -0.3660654 | 0.0574719 | 1.8969E-10 |
| Years of schooling \|\| id:ieu-a-1239 | Major Depressive Disorder \|\| id:ieu-a-1188 | 173005 | rs77025239 | -0.3694784 | 0.05734887 | 1.1741E-10 |
| Years of schooling \|\| id:ieu-a-1239 | Major Depressive Disorder \|\| id:ieu-a-1188 | 173005 | rs77128898 | -0.3671083 | 0.05741581 | 1.6176E-10 |
| Years of schooling \|\| id:ieu-a-1239 | Major Depressive Disorder \|\| id:ieu-a-1188 | 173005 | rs77702622 | -0.3673769 | 0.05747583 | 1.6389E-10 |
| Years of schooling \|\| id:ieu-a-1239 | Major Depressive Disorder \|\| id:ieu-a-1188 | 173005 | rs77835879 | -0.3662999 | 0.05745313 | 1.8221E-10 |
| Years of schooling \|\| id:ieu-a-1239 | Major Depressive Disorder \|\| id:ieu-a-1188 | 173005 | rs7796203 | -0.3586876 | 0.05686979 | 2.8418E-10 |
| Years of schooling \|\| id:ieu-a-1239 | Major Depressive Disorder \|\| id:ieu-a-1188 | 173005 | rs7803932 | -0.3638648 | 0.05741262 | 2.332E-10 |
| Years of schooling \|\| id:ieu-a-1239 | Major Depressive Disorder \|\| id:ieu-a-1188 | 173005 | rs7808399 | -0.3682183 | 0.0574308 | 1.4408E-10 |
| Years of schooling \|\| id:ieu-a-1239 | Major Depressive Disorder \|\| id:ieu-a-1188 | 173005 | rs7863447 | -0.3662357 | 0.05748511 | 1.8784E-10 |
| Years of schooling \|\| id:ieu-a-1239 | Major Depressive Disorder \|\| id:ieu-a-1188 | 173005 | rs78721320 | -0.371461 | 0.05712469 | 7.8925E-11 |
| Years of schooling \|\| id:ieu-a-1239 | Major Depressive Disorder \|\| id:ieu-a-1188 | 173005 | rs790647 | -0.3629554 | 0.05743157 | 2.6192E-10 |
| Years of schooling \|\| id:ieu-a-1239 | Major Depressive Disorder \|\| id:ieu-a-1188 | 173005 | rs7924036 | -0.3620606 | 0.05748659 | 3.0117E-10 |
| Years of schooling \|\| id:ieu-a-1239 | Major Depressive Disorder \|\| id:ieu-a-1188 | 173005 | rs79265434 | -0.3738216 | 0.05713215 | 6.0255E-11 |
| Years of schooling \|\| id:ieu-a-1239 | Major Depressive Disorder \|\| id:ieu-a-1188 | 173005 | rs79269403 | -0.3673303 | 0.05748747 | 1.6616E-10 |
| Years of schooling \|\| id:ieu-a-1239 | Major Depressive Disorder \|\| id:ieu-a-1188 | 173005 | rs795230 | -0.3647081 | 0.05743171 | 2.1491E-10 |
| Years of schooling \|\| id:ieu-a-1239 | Major Depressive Disorder \|\| id:ieu-a-1188 | 173005 | rs79523955 | -0.3645678 | 0.05744994 | 2.2122E-10 |
| Years of schooling \|\| id:ieu-a-1239 | Major Depressive Disorder \|\| id:ieu-a-1188 | 173005 | rs7977614 | -0.3656968 | 0.05748629 | 1.9984E-10 |
| Years of schooling \|\| id:ieu-a-1239 | Major Depressive Disorder \|\| id:ieu-a-1188 | 173005 | rs7993663 | -0.3634234 | 0.0574192 | 2.4627E-10 |
| Years of schooling \|\| id:ieu-a-1239 | Major Depressive Disorder \|\| id:ieu-a-1188 | 173005 | rs80171383 | -0.3659851 | 0.05746542 | 1.9053E-10 |
| Years of schooling \|\| id:ieu-a-1239 | Major Depressive Disorder \|\| id:ieu-a-1188 | 173005 | rs8020034 | -0.374925 | 0.05707141 | 5.0518E-11 |
| Years of schooling \|\| id:ieu-a-1239 | Major Depressive Disorder \|\| id:ieu-a-1188 | 173005 | rs818415 | -0.3677839 | 0.05742993 | 1.5131E-10 |
| Years of schooling \|\| id:ieu-a-1239 | Major Depressive Disorder \|\| id:ieu-a-1188 | 173005 | rs837080 | -0.3669248 | 0.05745595 | 1.7007E-10 |
| Years of schooling \|\| id:ieu-a-1239 | Major Depressive Disorder \|\| id:ieu-a-1188 | 173005 | rs892612 | -0.3687346 | 0.0574076 | 1.3353E-10 |
| Years of schooling \|\| id:ieu-a-1239 | Major Depressive Disorder \|\| id:ieu-a-1188 | 173005 | rs894067 | -0.3635356 | 0.05739047 | 2.3823E-10 |
| Years of schooling \|\| id:ieu-a-1239 | Major Depressive Disorder \|\| id:ieu-a-1188 | 173005 | rs9289300 | -0.3735594 | 0.05696068 | 5.4462E-11 |
| Years of schooling \|\| id:ieu-a-1239 | Major Depressive Disorder \|\| id:ieu-a-1188 | 173005 | rs9320493 | -0.3707833 | 0.05720665 | 9.0832E-11 |
| Years of schooling \|\| id:ieu-a-1239 | Major Depressive Disorder \|\| id:ieu-a-1188 | 173005 | rs9342482 | -0.3670412 | 0.05747826 | 1.7057E-10 |
| Years of schooling \|\| id:ieu-a-1239 | Major Depressive Disorder \|\| id:ieu-a-1188 | 173005 | rs9349956 | -0.3699353 | 0.05747603 | 1.2239E-10 |
| Years of schooling \|\| id:ieu-a-1239 | Major Depressive Disorder \|\| id:ieu-a-1188 | 173005 | rs9372625 | -0.3726348 | 0.05773064 | 1.0842E-10 |
| Years of schooling \|\| id:ieu-a-1239 | Major Depressive Disorder \|\| id:ieu-a-1188 | 173005 | rs9384679 | -0.3696272 | 0.05729149 | 1.1061E-10 |
| Years of schooling \|\| id:ieu-a-1239 | Major Depressive Disorder \|\| id:ieu-a-1188 | 173005 | rs9386319 | -0.366302 | 0.05746222 | 1.8337E-10 |
| Years of schooling \|\| id:ieu-a-1239 | Major Depressive Disorder \|\| id:ieu-a-1188 | 173005 | rs9436866 | -0.3620782 | 0.05732232 | 2.675E-10 |
| Years of schooling \|\| id:ieu-a-1239 | Major Depressive Disorder \|\| id:ieu-a-1188 | 173005 | rs9503598 | -0.3657911 | 0.05747799 | 1.9654E-10 |
| Years of schooling \|\| id:ieu-a-1239 | Major Depressive Disorder \|\| id:ieu-a-1188 | 173005 | rs9556958 | -0.3667337 | 0.05747663 | 1.7643E-10 |
| Years of schooling \|\| id:ieu-a-1239 | Major Depressive Disorder \|\| id:ieu-a-1188 | 173005 | rs9616906 | -0.3730598 | 0.05730339 | 7.5022E-11 |
| Years of schooling \|\| id:ieu-a-1239 | Major Depressive Disorder \|\| id:ieu-a-1188 | 173005 | rs9679654 | -0.3664249 | 0.05747016 | 1.8187E-10 |
| Years of schooling \|\| id:ieu-a-1239 | Major Depressive Disorder \|\| id:ieu-a-1188 | 173005 | rs9704097 | -0.3662957 | 0.05747308 | 1.8495E-10 |
| Years of schooling \|\| id:ieu-a-1239 | Major Depressive Disorder \|\| id:ieu-a-1188 | 173005 | rs9882532 | -0.3694605 | 0.05740485 | 1.2262E-10 |
| Years of schooling \|\| id:ieu-a-1239 | Major Depressive Disorder \|\| id:ieu-a-1188 | 173005 | rs9914918 | -0.3645575 | 0.05744547 | 2.2077E-10 |
| Years of schooling \|\| id:ieu-a-1239 | Major Depressive Disorder \|\| id:ieu-a-1188 | 173005 | rs9933256 | -0.3654736 | 0.05745189 | 1.9994E-10 |
| Years of schooling \|\| id:ieu-a-1239 | Major Depressive Disorder \|\| id:ieu-a-1188 | 173005 | rs9936270 | -0.3700588 | 0.05736926 | 1.115E-10 |
| Years of schooling \|\| id:ieu-a-1239 | Major Depressive Disorder \|\| id:ieu-a-1188 | 173005 | rs9964724 | -0.3558614 | 0.05728546 | 5.2291E-10 |
| Years of schooling \|\| id:ieu-a-1239 | Major Depressive Disorder \|\| id:ieu-a-1188 | 173005 | rs9995567 | -0.3653639 | 0.05745091 | 2.023E-10 |
| Years of schooling \|\| id:ieu-a-1239 | Major Depressive Disorder \|\| id:ieu-a-1188 | 173005 | All | -0.3661831 | 0.05726788 | 1.6137E-10 |
| smoking initiation \|\| id:ieu-b-4877 | Major Depressive Disorder \|\| id:ieu-a-1188 | 173005 | rs10001365 | 0.33234733 | 0.05842163 | 1.2796E-08 |
| smoking initiation \|\| id:ieu-b-4877 | Major Depressive Disorder \|\| id:ieu-a-1188 | 173005 | rs10114490 | 0.34116716 | 0.05777616 | 3.5269E-09 |
| smoking initiation \|\| id:ieu-b-4877 | Major Depressive Disorder \|\| id:ieu-a-1188 | 173005 | rs10233018 | 0.31485733 | 0.0560809 | 1.9731E-08 |
| smoking initiation \|\| id:ieu-b-4877 | Major Depressive Disorder \|\| id:ieu-a-1188 | 173005 | rs10260968 | 0.32962282 | 0.05796344 | 1.2949E-08 |
| smoking initiation \|\| id:ieu-b-4877 | Major Depressive Disorder \|\| id:ieu-a-1188 | 173005 | rs10279261 | 0.33487837 | 0.05830056 | 9.2466E-09 |
| smoking initiation \|\| id:ieu-b-4877 | Major Depressive Disorder \|\| id:ieu-a-1188 | 173005 | rs10498846 | 0.33407529 | 0.05822322 | 9.5894E-09 |
| smoking initiation \|\| id:ieu-b-4877 | Major Depressive Disorder \|\| id:ieu-a-1188 | 173005 | rs1050847 | 0.33213023 | 0.05815864 | 1.1247E-08 |
| smoking initiation \|\| id:ieu-b-4877 | Major Depressive Disorder \|\| id:ieu-a-1188 | 173005 | rs10905461 | 0.33619847 | 0.0582599 | 7.8959E-09 |
| smoking initiation \|\| id:ieu-b-4877 | Major Depressive Disorder \|\| id:ieu-a-1188 | 173005 | rs11057005 | 0.33043703 | 0.05812412 | 1.3079E-08 |
| smoking initiation \|\| id:ieu-b-4877 | Major Depressive Disorder \|\| id:ieu-a-1188 | 173005 | rs11078713 | 0.33861313 | 0.05806642 | 5.4938E-09 |
| smoking initiation \|\| id:ieu-b-4877 | Major Depressive Disorder \|\| id:ieu-a-1188 | 173005 | rs1154693 | 0.32953585 | 0.05818583 | 1.4831E-08 |
| smoking initiation \|\| id:ieu-b-4877 | Major Depressive Disorder \|\| id:ieu-a-1188 | 173005 | rs11658881 | 0.33276605 | 0.05824138 | 1.1063E-08 |
| smoking initiation \|\| id:ieu-b-4877 | Major Depressive Disorder \|\| id:ieu-a-1188 | 173005 | rs11712680 | 0.33021398 | 0.05811903 | 1.3337E-08 |
| smoking initiation \|\| id:ieu-b-4877 | Major Depressive Disorder \|\| id:ieu-a-1188 | 173005 | rs117143374 | 0.33052132 | 0.05806781 | 1.2557E-08 |
| smoking initiation \|\| id:ieu-b-4877 | Major Depressive Disorder \|\| id:ieu-a-1188 | 173005 | rs11872397 | 0.33268689 | 0.05828209 | 1.1417E-08 |
| smoking initiation \|\| id:ieu-b-4877 | Major Depressive Disorder \|\| id:ieu-a-1188 | 173005 | rs12042107 | 0.32694018 | 0.05780259 | 1.5481E-08 |
| smoking initiation \|\| id:ieu-b-4877 | Major Depressive Disorder \|\| id:ieu-a-1188 | 173005 | rs12186738 | 0.32319753 | 0.05735242 | 1.7476E-08 |
| smoking initiation \|\| id:ieu-b-4877 | Major Depressive Disorder \|\| id:ieu-a-1188 | 173005 | rs12333760 | 0.3318754 | 0.05815144 | 1.1492E-08 |
| smoking initiation \|\| id:ieu-b-4877 | Major Depressive Disorder \|\| id:ieu-a-1188 | 173005 | rs12441907 | 0.34222712 | 0.05788999 | 3.3863E-09 |
| smoking initiation \|\| id:ieu-b-4877 | Major Depressive Disorder \|\| id:ieu-a-1188 | 173005 | rs12474587 | 0.34041297 | 0.05835479 | 5.4275E-09 |
| smoking initiation \|\| id:ieu-b-4877 | Major Depressive Disorder \|\| id:ieu-a-1188 | 173005 | rs12545053 | 0.32943573 | 0.05796869 | 1.3236E-08 |
| smoking initiation \|\| id:ieu-b-4877 | Major Depressive Disorder \|\| id:ieu-a-1188 | 173005 | rs12632110 | 0.33591098 | 0.05832142 | 8.4289E-09 |
| smoking initiation \|\| id:ieu-b-4877 | Major Depressive Disorder \|\| id:ieu-a-1188 | 173005 | rs13030994 | 0.34236921 | 0.0588353 | 5.9161E-09 |
| smoking initiation \|\| id:ieu-b-4877 | Major Depressive Disorder \|\| id:ieu-a-1188 | 173005 | rs13261666 | 0.32378497 | 0.05781715 | 2.1416E-08 |
| smoking initiation \|\| id:ieu-b-4877 | Major Depressive Disorder \|\| id:ieu-a-1188 | 173005 | rs134529 | 0.3466069 | 0.0563919 | 7.9264E-10 |
| smoking initiation \|\| id:ieu-b-4877 | Major Depressive Disorder \|\| id:ieu-a-1188 | 173005 | rs1385108 | 0.33090681 | 0.05818781 | 1.2939E-08 |
| smoking initiation \|\| id:ieu-b-4877 | Major Depressive Disorder \|\| id:ieu-a-1188 | 173005 | rs1435741 | 0.33402007 | 0.05867373 | 1.2494E-08 |
| smoking initiation \|\| id:ieu-b-4877 | Major Depressive Disorder \|\| id:ieu-a-1188 | 173005 | rs1445649 | 0.3369471 | 0.05838027 | 7.8526E-09 |
| smoking initiation \|\| id:ieu-b-4877 | Major Depressive Disorder \|\| id:ieu-a-1188 | 173005 | rs1869243 | 0.33296321 | 0.05824378 | 1.086E-08 |
| smoking initiation \|\| id:ieu-b-4877 | Major Depressive Disorder \|\| id:ieu-a-1188 | 173005 | rs1899896 | 0.33591783 | 0.05841323 | 8.8867E-09 |
| smoking initiation \|\| id:ieu-b-4877 | Major Depressive Disorder \|\| id:ieu-a-1188 | 173005 | rs1971318 | 0.33355579 | 0.05828022 | 1.0447E-08 |
| smoking initiation \|\| id:ieu-b-4877 | Major Depressive Disorder \|\| id:ieu-a-1188 | 173005 | rs2046850 | 0.33440892 | 0.0582737 | 9.5467E-09 |
| smoking initiation \|\| id:ieu-b-4877 | Major Depressive Disorder \|\| id:ieu-a-1188 | 173005 | rs2140114 | 0.32909996 | 0.05814948 | 1.5177E-08 |
| smoking initiation \|\| id:ieu-b-4877 | Major Depressive Disorder \|\| id:ieu-a-1188 | 173005 | rs2378662 | 0.32875657 | 0.057928 | 1.3848E-08 |
| smoking initiation \|\| id:ieu-b-4877 | Major Depressive Disorder \|\| id:ieu-a-1188 | 173005 | rs240963 | 0.33016547 | 0.05844084 | 1.6085E-08 |
| smoking initiation \|\| id:ieu-b-4877 | Major Depressive Disorder \|\| id:ieu-a-1188 | 173005 | rs2631024 | 0.33871215 | 0.05809509 | 5.5326E-09 |
| smoking initiation \|\| id:ieu-b-4877 | Major Depressive Disorder \|\| id:ieu-a-1188 | 173005 | rs266047 | 0.3486084 | 0.0577391 | 1.5638E-09 |
| smoking initiation \|\| id:ieu-b-4877 | Major Depressive Disorder \|\| id:ieu-a-1188 | 173005 | rs3001723 | 0.33334704 | 0.05873923 | 1.3866E-08 |
| smoking initiation \|\| id:ieu-b-4877 | Major Depressive Disorder \|\| id:ieu-a-1188 | 173005 | rs35702515 | 0.34403217 | 0.05732284 | 1.9531E-09 |
| smoking initiation \|\| id:ieu-b-4877 | Major Depressive Disorder \|\| id:ieu-a-1188 | 173005 | rs3800227 | 0.32990667 | 0.05803706 | 1.3126E-08 |
| smoking initiation \|\| id:ieu-b-4877 | Major Depressive Disorder \|\| id:ieu-a-1188 | 173005 | rs3801289 | 0.33564254 | 0.05828316 | 8.4701E-09 |
| smoking initiation \|\| id:ieu-b-4877 | Major Depressive Disorder \|\| id:ieu-a-1188 | 173005 | rs3904512 | 0.33971972 | 0.05802113 | 4.7671E-09 |
| smoking initiation \|\| id:ieu-b-4877 | Major Depressive Disorder \|\| id:ieu-a-1188 | 173005 | rs4044321 | 0.31444477 | 0.05591253 | 1.8673E-08 |
| smoking initiation \|\| id:ieu-b-4877 | Major Depressive Disorder \|\| id:ieu-a-1188 | 173005 | rs4236259 | 0.3445114 | 0.05750788 | 2.0896E-09 |
| smoking initiation \|\| id:ieu-b-4877 | Major Depressive Disorder \|\| id:ieu-a-1188 | 173005 | rs4352629 | 0.35493341 | 0.05598464 | 2.3001E-10 |
| smoking initiation \|\| id:ieu-b-4877 | Major Depressive Disorder \|\| id:ieu-a-1188 | 173005 | rs4523689 | 0.32940003 | 0.05800085 | 1.353E-08 |
| smoking initiation \|\| id:ieu-b-4877 | Major Depressive Disorder \|\| id:ieu-a-1188 | 173005 | rs4543592 | 0.32797749 | 0.05791724 | 1.4887E-08 |
| smoking initiation \|\| id:ieu-b-4877 | Major Depressive Disorder \|\| id:ieu-a-1188 | 173005 | rs4674993 | 0.3240763 | 0.05696179 | 1.2751E-08 |
| smoking initiation \|\| id:ieu-b-4877 | Major Depressive Disorder \|\| id:ieu-a-1188 | 173005 | rs4781977 | 0.34279987 | 0.05736128 | 2.2847E-09 |
| smoking initiation \|\| id:ieu-b-4877 | Major Depressive Disorder \|\| id:ieu-a-1188 | 173005 | rs6265 | 0.35359911 | 0.05580528 | 2.3535E-10 |
| smoking initiation \|\| id:ieu-b-4877 | Major Depressive Disorder \|\| id:ieu-a-1188 | 173005 | rs6433897 | 0.34039552 | 0.05783735 | 3.9711E-09 |
| smoking initiation \|\| id:ieu-b-4877 | Major Depressive Disorder \|\| id:ieu-a-1188 | 173005 | rs66680800 | 0.34629521 | 0.05654795 | 9.13E-10 |
| smoking initiation \|\| id:ieu-b-4877 | Major Depressive Disorder \|\| id:ieu-a-1188 | 173005 | rs6669839 | 0.33001181 | 0.0580892 | 1.3381E-08 |
| smoking initiation \|\| id:ieu-b-4877 | Major Depressive Disorder \|\| id:ieu-a-1188 | 173005 | rs6728726 | 0.32778236 | 0.05823323 | 1.8148E-08 |
| smoking initiation \|\| id:ieu-b-4877 | Major Depressive Disorder \|\| id:ieu-a-1188 | 173005 | rs6893752 | 0.33788283 | 0.05819074 | 6.3804E-09 |
| smoking initiation \|\| id:ieu-b-4877 | Major Depressive Disorder \|\| id:ieu-a-1188 | 173005 | rs7197072 | 0.33759366 | 0.05812432 | 6.3174E-09 |
| smoking initiation \|\| id:ieu-b-4877 | Major Depressive Disorder \|\| id:ieu-a-1188 | 173005 | rs7224742 | 0.33581874 | 0.05826732 | 8.2429E-09 |
| smoking initiation \|\| id:ieu-b-4877 | Major Depressive Disorder \|\| id:ieu-a-1188 | 173005 | rs72789632 | 0.32713606 | 0.05781601 | 1.5295E-08 |
| smoking initiation \|\| id:ieu-b-4877 | Major Depressive Disorder \|\| id:ieu-a-1188 | 173005 | rs7322872 | 0.3393431 | 0.05800788 | 4.9173E-09 |
| smoking initiation \|\| id:ieu-b-4877 | Major Depressive Disorder \|\| id:ieu-a-1188 | 173005 | rs76214862 | 0.33425984 | 0.05826471 | 9.6415E-09 |
| smoking initiation \|\| id:ieu-b-4877 | Major Depressive Disorder \|\| id:ieu-a-1188 | 173005 | rs76608582 | 0.33550292 | 0.05820627 | 8.2127E-09 |
| smoking initiation \|\| id:ieu-b-4877 | Major Depressive Disorder \|\| id:ieu-a-1188 | 173005 | rs7929518 | 0.33147354 | 0.05817194 | 1.211E-08 |
| smoking initiation \|\| id:ieu-b-4877 | Major Depressive Disorder \|\| id:ieu-a-1188 | 173005 | rs7938812 | 0.32879845 | 0.0594331 | 3.1616E-08 |
| smoking initiation \|\| id:ieu-b-4877 | Major Depressive Disorder \|\| id:ieu-a-1188 | 173005 | rs7969559 | 0.33818689 | 0.05820298 | 6.2296E-09 |
| smoking initiation \|\| id:ieu-b-4877 | Major Depressive Disorder \|\| id:ieu-a-1188 | 173005 | rs9401770 | 0.33921315 | 0.0582998 | 5.9404E-09 |
| smoking initiation \|\| id:ieu-b-4877 | Major Depressive Disorder \|\| id:ieu-a-1188 | 173005 | rs962625 | 0.3393104 | 0.05806048 | 5.0936E-09 |
| smoking initiation \|\| id:ieu-b-4877 | Major Depressive Disorder \|\| id:ieu-a-1188 | 173005 | rs993700 | 0.33104529 | 0.058227 | 1.3049E-08 |
| smoking initiation \|\| id:ieu-b-4877 | Major Depressive Disorder \|\| id:ieu-a-1188 | 173005 | All | 0.33449802 | 0.05750687 | 6.0035E-09 |
| Sleeplessness / insomnia \|\| id:ukb-a-13 | Major Depressive Disorder \|\| id:ieu-a-1188 | 173005 | rs10087341 | 0.69810914 | 0.19457925 | 0.0003335 |
| Sleeplessness / insomnia \|\| id:ukb-a-13 | Major Depressive Disorder \|\| id:ieu-a-1188 | 173005 | rs10156602 | 0.61763903 | 0.18076443 | 0.00063358 |
| Sleeplessness / insomnia \|\| id:ukb-a-13 | Major Depressive Disorder \|\| id:ieu-a-1188 | 173005 | rs11152363 | 0.62867549 | 0.18855625 | 0.00085559 |
| Sleeplessness / insomnia \|\| id:ukb-a-13 | Major Depressive Disorder \|\| id:ieu-a-1188 | 173005 | rs113851554 | 0.7713722 | 0.20253719 | 0.00013979 |
| Sleeplessness / insomnia \|\| id:ukb-a-13 | Major Depressive Disorder \|\| id:ieu-a-1188 | 173005 | rs11635495 | 0.71016986 | 0.19158378 | 0.00020986 |
| Sleeplessness / insomnia \|\| id:ukb-a-13 | Major Depressive Disorder \|\| id:ieu-a-1188 | 173005 | rs11804386 | 0.68544467 | 0.1946782 | 0.00043007 |
| Sleeplessness / insomnia \|\| id:ukb-a-13 | Major Depressive Disorder \|\| id:ieu-a-1188 | 173005 | rs13186678 | 0.70464019 | 0.19303025 | 0.00026182 |
| Sleeplessness / insomnia \|\| id:ukb-a-13 | Major Depressive Disorder \|\| id:ieu-a-1188 | 173005 | rs1547630 | 0.69823456 | 0.19376662 | 0.00031398 |
| Sleeplessness / insomnia \|\| id:ukb-a-13 | Major Depressive Disorder \|\| id:ieu-a-1188 | 173005 | rs2132083 | 0.67593407 | 0.19472382 | 0.00051805 |
| Sleeplessness / insomnia \|\| id:ukb-a-13 | Major Depressive Disorder \|\| id:ieu-a-1188 | 173005 | rs224071 | 0.64878933 | 0.19137349 | 0.00069848 |
| Sleeplessness / insomnia \|\| id:ukb-a-13 | Major Depressive Disorder \|\| id:ieu-a-1188 | 173005 | rs2863957 | 0.75423058 | 0.17910578 | 2.5414E-05 |
| Sleeplessness / insomnia \|\| id:ukb-a-13 | Major Depressive Disorder \|\| id:ieu-a-1188 | 173005 | rs2956278 | 0.69152285 | 0.19438647 | 0.00037445 |
| Sleeplessness / insomnia \|\| id:ukb-a-13 | Major Depressive Disorder \|\| id:ieu-a-1188 | 173005 | rs324017 | 0.69875391 | 0.1944851 | 0.00032709 |
| Sleeplessness / insomnia \|\| id:ukb-a-13 | Major Depressive Disorder \|\| id:ieu-a-1188 | 173005 | rs3808937 | 0.75764058 | 0.17955619 | 2.4483E-05 |
| Sleeplessness / insomnia \|\| id:ukb-a-13 | Major Depressive Disorder \|\| id:ieu-a-1188 | 173005 | rs3817576 | 0.63528439 | 0.18794817 | 0.00072458 |
| Sleeplessness / insomnia \|\| id:ukb-a-13 | Major Depressive Disorder \|\| id:ieu-a-1188 | 173005 | rs4688760 | 0.64963909 | 0.19267914 | 0.0007473 |
| Sleeplessness / insomnia \|\| id:ukb-a-13 | Major Depressive Disorder \|\| id:ieu-a-1188 | 173005 | rs4886140 | 0.66820344 | 0.19491029 | 0.00060746 |
| Sleeplessness / insomnia \|\| id:ukb-a-13 | Major Depressive Disorder \|\| id:ieu-a-1188 | 173005 | rs4943439 | 0.63634141 | 0.18915635 | 0.00076793 |
| Sleeplessness / insomnia \|\| id:ukb-a-13 | Major Depressive Disorder \|\| id:ieu-a-1188 | 173005 | rs6690017 | 0.66808823 | 0.19445303 | 0.00059096 |
| Sleeplessness / insomnia \|\| id:ukb-a-13 | Major Depressive Disorder \|\| id:ieu-a-1188 | 173005 | rs6744461 | 0.67921328 | 0.19510174 | 0.00049893 |
| Sleeplessness / insomnia \|\| id:ukb-a-13 | Major Depressive Disorder \|\| id:ieu-a-1188 | 173005 | rs71373536 | 0.70404878 | 0.19479646 | 0.00030118 |
| Sleeplessness / insomnia \|\| id:ukb-a-13 | Major Depressive Disorder \|\| id:ieu-a-1188 | 173005 | rs7572387 | 0.62862387 | 0.18783971 | 0.0008181 |
| Sleeplessness / insomnia \|\| id:ukb-a-13 | Major Depressive Disorder \|\| id:ieu-a-1188 | 173005 | rs9815484 | 0.63734118 | 0.18926056 | 0.00075844 |
| Sleeplessness / insomnia \|\| id:ukb-a-13 | Major Depressive Disorder \|\| id:ieu-a-1188 | 173005 | rs9878792 | 0.67384784 | 0.19552804 | 0.00056832 |
| Sleeplessness / insomnia \|\| id:ukb-a-13 | Major Depressive Disorder \|\| id:ieu-a-1188 | 173005 | All | 0.67955356 | 0.18745383 | 0.00028876 |
| Neuroticism score \|\| id:ukb-a-230 | Major Depressive Disorder \|\| id:ieu-a-1188 | 173005 | rs10119773 | 0.26353692 | 0.02768273 | 1.7334E-21 |
| Neuroticism score \|\| id:ukb-a-230 | Major Depressive Disorder \|\| id:ieu-a-1188 | 173005 | rs10144845 | 0.26420886 | 0.0278196 | 2.1557E-21 |
| Neuroticism score \|\| id:ukb-a-230 | Major Depressive Disorder \|\| id:ieu-a-1188 | 173005 | rs10455007 | 0.26961451 | 0.02738031 | 7.0605E-23 |
| Neuroticism score \|\| id:ukb-a-230 | Major Depressive Disorder \|\| id:ieu-a-1188 | 173005 | rs10497655 | 0.26667053 | 0.02762459 | 4.7564E-22 |
| Neuroticism score \|\| id:ukb-a-230 | Major Depressive Disorder \|\| id:ieu-a-1188 | 173005 | rs10501696 | 0.26455183 | 0.02768425 | 1.2235E-21 |
| Neuroticism score \|\| id:ukb-a-230 | Major Depressive Disorder \|\| id:ieu-a-1188 | 173005 | rs11090045 | 0.26901568 | 0.02754775 | 1.5844E-22 |
| Neuroticism score \|\| id:ukb-a-230 | Major Depressive Disorder \|\| id:ieu-a-1188 | 173005 | rs11509880 | 0.26088211 | 0.02742036 | 1.8316E-21 |
| Neuroticism score \|\| id:ukb-a-230 | Major Depressive Disorder \|\| id:ieu-a-1188 | 173005 | rs11665070 | 0.26096293 | 0.02781156 | 6.3968E-21 |
| Neuroticism score \|\| id:ukb-a-230 | Major Depressive Disorder \|\| id:ieu-a-1188 | 173005 | rs11682716 | 0.26642516 | 0.02763029 | 5.2883E-22 |
| Neuroticism score \|\| id:ukb-a-230 | Major Depressive Disorder \|\| id:ieu-a-1188 | 173005 | rs117298864 | 0.26247231 | 0.02752454 | 1.4853E-21 |
| Neuroticism score \|\| id:ukb-a-230 | Major Depressive Disorder \|\| id:ieu-a-1188 | 173005 | rs12938775 | 0.26466177 | 0.02770715 | 1.2708E-21 |
| Neuroticism score \|\| id:ukb-a-230 | Major Depressive Disorder \|\| id:ieu-a-1188 | 173005 | rs12969553 | 0.25709234 | 0.02669645 | 5.961E-22 |
| Neuroticism score \|\| id:ukb-a-230 | Major Depressive Disorder \|\| id:ieu-a-1188 | 173005 | rs13226841 | 0.26914131 | 0.02749077 | 1.24E-22 |
| Neuroticism score \|\| id:ukb-a-230 | Major Depressive Disorder \|\| id:ieu-a-1188 | 173005 | rs1442129 | 0.26343885 | 0.02758535 | 1.2975E-21 |
| Neuroticism score \|\| id:ukb-a-230 | Major Depressive Disorder \|\| id:ieu-a-1188 | 173005 | rs147861665 | 0.26609406 | 0.02771125 | 7.8116E-22 |
| Neuroticism score \|\| id:ukb-a-230 | Major Depressive Disorder \|\| id:ieu-a-1188 | 173005 | rs1542212 | 0.27100986 | 0.0270966 | 1.4993E-23 |
| Neuroticism score \|\| id:ukb-a-230 | Major Depressive Disorder \|\| id:ieu-a-1188 | 173005 | rs1673931 | 0.26489394 | 0.02765124 | 9.7212E-22 |
| Neuroticism score \|\| id:ukb-a-230 | Major Depressive Disorder \|\| id:ieu-a-1188 | 173005 | rs1806153 | 0.26192281 | 0.02761667 | 2.4418E-21 |
| Neuroticism score \|\| id:ukb-a-230 | Major Depressive Disorder \|\| id:ieu-a-1188 | 173005 | rs2102341 | 0.26191964 | 0.02763928 | 2.6332E-21 |
| Neuroticism score \|\| id:ukb-a-230 | Major Depressive Disorder \|\| id:ieu-a-1188 | 173005 | rs2206544 | 0.26553586 | 0.02771813 | 9.7173E-22 |
| Neuroticism score \|\| id:ukb-a-230 | Major Depressive Disorder \|\| id:ieu-a-1188 | 173005 | rs2269426 | 0.26151797 | 0.02766268 | 3.2668E-21 |
| Neuroticism score \|\| id:ukb-a-230 | Major Depressive Disorder \|\| id:ieu-a-1188 | 173005 | rs2278609 | 0.26859653 | 0.02745126 | 1.3126E-22 |
| Neuroticism score \|\| id:ukb-a-230 | Major Depressive Disorder \|\| id:ieu-a-1188 | 173005 | rs2715147 | 0.25979127 | 0.02689187 | 4.4331E-22 |
| Neuroticism score \|\| id:ukb-a-230 | Major Depressive Disorder \|\| id:ieu-a-1188 | 173005 | rs2791459 | 0.26680319 | 0.02762542 | 4.5516E-22 |
| Neuroticism score \|\| id:ukb-a-230 | Major Depressive Disorder \|\| id:ieu-a-1188 | 173005 | rs28427480 | 0.26057458 | 0.02744501 | 2.2143E-21 |
| Neuroticism score \|\| id:ukb-a-230 | Major Depressive Disorder \|\| id:ieu-a-1188 | 173005 | rs2921036 | 0.27362089 | 0.02757817 | 3.3517E-23 |
| Neuroticism score \|\| id:ukb-a-230 | Major Depressive Disorder \|\| id:ieu-a-1188 | 173005 | rs297346 | 0.26579857 | 0.02768272 | 7.8707E-22 |
| Neuroticism score \|\| id:ukb-a-230 | Major Depressive Disorder \|\| id:ieu-a-1188 | 173005 | rs34796300 | 0.26830611 | 0.02748738 | 1.6541E-22 |
| Neuroticism score \|\| id:ukb-a-230 | Major Depressive Disorder \|\| id:ieu-a-1188 | 173005 | rs3741475 | 0.26766646 | 0.02762255 | 3.3206E-22 |
| Neuroticism score \|\| id:ukb-a-230 | Major Depressive Disorder \|\| id:ieu-a-1188 | 173005 | rs3811489 | 0.2634738 | 0.02765595 | 1.6214E-21 |
| Neuroticism score \|\| id:ukb-a-230 | Major Depressive Disorder \|\| id:ieu-a-1188 | 173005 | rs3849470 | 0.26192663 | 0.02752024 | 1.7723E-21 |
| Neuroticism score \|\| id:ukb-a-230 | Major Depressive Disorder \|\| id:ieu-a-1188 | 173005 | rs4140799 | 0.26791715 | 0.02762748 | 3.0901E-22 |
| Neuroticism score \|\| id:ukb-a-230 | Major Depressive Disorder \|\| id:ieu-a-1188 | 173005 | rs4632195 | 0.25610335 | 0.02663148 | 6.8066E-22 |
| Neuroticism score \|\| id:ukb-a-230 | Major Depressive Disorder \|\| id:ieu-a-1188 | 173005 | rs4738602 | 0.26857802 | 0.02738204 | 1.0345E-22 |
| Neuroticism score \|\| id:ukb-a-230 | Major Depressive Disorder \|\| id:ieu-a-1188 | 173005 | rs56116032 | 0.2591726 | 0.0269087 | 5.8834E-22 |
| Neuroticism score \|\| id:ukb-a-230 | Major Depressive Disorder \|\| id:ieu-a-1188 | 173005 | rs56226325 | 0.26043936 | 0.02732564 | 1.5585E-21 |
| Neuroticism score \|\| id:ukb-a-230 | Major Depressive Disorder \|\| id:ieu-a-1188 | 173005 | rs57838764 | 0.26467672 | 0.02771041 | 1.278E-21 |
| Neuroticism score \|\| id:ukb-a-230 | Major Depressive Disorder \|\| id:ieu-a-1188 | 173005 | rs59970005 | 0.26631854 | 0.02768436 | 6.5948E-22 |
| Neuroticism score \|\| id:ukb-a-230 | Major Depressive Disorder \|\| id:ieu-a-1188 | 173005 | rs62062288 | 0.26084556 | 0.02701065 | 4.5849E-22 |
| Neuroticism score \|\| id:ukb-a-230 | Major Depressive Disorder \|\| id:ieu-a-1188 | 173005 | rs6743916 | 0.27151166 | 0.0267613 | 3.4635E-24 |
| Neuroticism score \|\| id:ukb-a-230 | Major Depressive Disorder \|\| id:ieu-a-1188 | 173005 | rs6916891 | 0.26950984 | 0.02723806 | 4.3934E-23 |
| Neuroticism score \|\| id:ukb-a-230 | Major Depressive Disorder \|\| id:ieu-a-1188 | 173005 | rs6976111 | 0.26099555 | 0.02742762 | 1.8033E-21 |
| Neuroticism score \|\| id:ukb-a-230 | Major Depressive Disorder \|\| id:ieu-a-1188 | 173005 | rs7107293 | 0.26468565 | 0.02792803 | 2.6065E-21 |
| Neuroticism score \|\| id:ukb-a-230 | Major Depressive Disorder \|\| id:ieu-a-1188 | 173005 | rs7107356 | 0.26231227 | 0.0277206 | 3.0004E-21 |
| Neuroticism score \|\| id:ukb-a-230 | Major Depressive Disorder \|\| id:ieu-a-1188 | 173005 | rs716508 | 0.26142616 | 0.0274247 | 1.5353E-21 |
| Neuroticism score \|\| id:ukb-a-230 | Major Depressive Disorder \|\| id:ieu-a-1188 | 173005 | rs7338774 | 0.27002258 | 0.02728061 | 4.2482E-23 |
| Neuroticism score \|\| id:ukb-a-230 | Major Depressive Disorder \|\| id:ieu-a-1188 | 173005 | rs7502590 | 0.26726625 | 0.02759312 | 3.459E-22 |
| Neuroticism score \|\| id:ukb-a-230 | Major Depressive Disorder \|\| id:ieu-a-1188 | 173005 | rs7567451 | 0.26398439 | 0.02763192 | 1.2526E-21 |
| Neuroticism score \|\| id:ukb-a-230 | Major Depressive Disorder \|\| id:ieu-a-1188 | 173005 | rs7869969 | 0.26126874 | 0.02748327 | 1.9725E-21 |
| Neuroticism score \|\| id:ukb-a-230 | Major Depressive Disorder \|\| id:ieu-a-1188 | 173005 | rs7871494 | 0.26170251 | 0.02748904 | 1.7276E-21 |
| Neuroticism score \|\| id:ukb-a-230 | Major Depressive Disorder \|\| id:ieu-a-1188 | 173005 | rs8053004 | 0.26541999 | 0.02769031 | 9.2187E-22 |
| Neuroticism score \|\| id:ukb-a-230 | Major Depressive Disorder \|\| id:ieu-a-1188 | 173005 | rs8062719 | 0.26583161 | 0.02762554 | 6.414E-22 |
| Neuroticism score \|\| id:ukb-a-230 | Major Depressive Disorder \|\| id:ieu-a-1188 | 173005 | rs836927 | 0.26980107 | 0.02714263 | 2.7849E-23 |
| Neuroticism score \|\| id:ukb-a-230 | Major Depressive Disorder \|\| id:ieu-a-1188 | 173005 | rs9298995 | 0.26856748 | 0.02745824 | 1.3594E-22 |
| Neuroticism score \|\| id:ukb-a-230 | Major Depressive Disorder \|\| id:ieu-a-1188 | 173005 | rs9424100 | 0.26519652 | 0.02769245 | 1.004E-21 |
| Neuroticism score \|\| id:ukb-a-230 | Major Depressive Disorder \|\| id:ieu-a-1188 | 173005 | rs9462364 | 0.26822019 | 0.0274045 | 1.2747E-22 |
| Neuroticism score \|\| id:ukb-a-230 | Major Depressive Disorder \|\| id:ieu-a-1188 | 173005 | All | 0.26488789 | 0.02723573 | 2.3417E-22 |
| Body mass index (BMI) \|\| id:ukb-a-248 | Major Depressive Disorder \|\| id:ieu-a-1188 | 173005 | rs10100245 | 0.19983482 | 0.03501431 | 1.1483E-08 |
| Body mass index (BMI) \|\| id:ukb-a-248 | Major Depressive Disorder \|\| id:ieu-a-1188 | 173005 | rs10185199 | 0.20564444 | 0.03481186 | 3.4774E-09 |
| Body mass index (BMI) \|\| id:ukb-a-248 | Major Depressive Disorder \|\| id:ieu-a-1188 | 173005 | rs10187101 | 0.20382791 | 0.03495973 | 5.5307E-09 |
| Body mass index (BMI) \|\| id:ukb-a-248 | Major Depressive Disorder \|\| id:ieu-a-1188 | 173005 | rs10404726 | 0.19881791 | 0.03491772 | 1.2417E-08 |
| Body mass index (BMI) \|\| id:ukb-a-248 | Major Depressive Disorder \|\| id:ieu-a-1188 | 173005 | rs10465231 | 0.20395331 | 0.03497722 | 5.5085E-09 |
| Body mass index (BMI) \|\| id:ukb-a-248 | Major Depressive Disorder \|\| id:ieu-a-1188 | 173005 | rs1064213 | 0.20597974 | 0.03475135 | 3.0806E-09 |
| Body mass index (BMI) \|\| id:ukb-a-248 | Major Depressive Disorder \|\| id:ieu-a-1188 | 173005 | rs10788493 | 0.20207983 | 0.03501713 | 7.8856E-09 |
| Body mass index (BMI) \|\| id:ukb-a-248 | Major Depressive Disorder \|\| id:ieu-a-1188 | 173005 | rs10803762 | 0.2011192 | 0.03500351 | 9.1549E-09 |
| Body mass index (BMI) \|\| id:ukb-a-248 | Major Depressive Disorder \|\| id:ieu-a-1188 | 173005 | rs10805383 | 0.20190744 | 0.03503841 | 8.2897E-09 |
| Body mass index (BMI) \|\| id:ukb-a-248 | Major Depressive Disorder \|\| id:ieu-a-1188 | 173005 | rs10865612 | 0.20522862 | 0.03497867 | 4.4308E-09 |
| Body mass index (BMI) \|\| id:ukb-a-248 | Major Depressive Disorder \|\| id:ieu-a-1188 | 173005 | rs10898330 | 0.20146567 | 0.03501322 | 8.7162E-09 |
| Body mass index (BMI) \|\| id:ukb-a-248 | Major Depressive Disorder \|\| id:ieu-a-1188 | 173005 | rs10938397 | 0.20057357 | 0.03513592 | 1.1397E-08 |
| Body mass index (BMI) \|\| id:ukb-a-248 | Major Depressive Disorder \|\| id:ieu-a-1188 | 173005 | rs10995427 | 0.19968305 | 0.03492267 | 1.0787E-08 |
| Body mass index (BMI) \|\| id:ukb-a-248 | Major Depressive Disorder \|\| id:ieu-a-1188 | 173005 | rs11012732 | 0.20143314 | 0.03507977 | 9.3485E-09 |
| Body mass index (BMI) \|\| id:ukb-a-248 | Major Depressive Disorder \|\| id:ieu-a-1188 | 173005 | rs11084554 | 0.2009617 | 0.03500694 | 9.4328E-09 |
| Body mass index (BMI) \|\| id:ukb-a-248 | Major Depressive Disorder \|\| id:ieu-a-1188 | 173005 | rs11099020 | 0.20080372 | 0.03499047 | 9.5343E-09 |
| Body mass index (BMI) \|\| id:ukb-a-248 | Major Depressive Disorder \|\| id:ieu-a-1188 | 173005 | rs11150745 | 0.20439084 | 0.03498838 | 5.1678E-09 |
| Body mass index (BMI) \|\| id:ukb-a-248 | Major Depressive Disorder \|\| id:ieu-a-1188 | 173005 | rs11223641 | 0.20256351 | 0.03499772 | 7.1269E-09 |
| Body mass index (BMI) \|\| id:ukb-a-248 | Major Depressive Disorder \|\| id:ieu-a-1188 | 173005 | rs112520079 | 0.20191591 | 0.03502902 | 8.2024E-09 |
| Body mass index (BMI) \|\| id:ukb-a-248 | Major Depressive Disorder \|\| id:ieu-a-1188 | 173005 | rs11264489 | 0.2010089 | 0.03498817 | 9.1892E-09 |
| Body mass index (BMI) \|\| id:ukb-a-248 | Major Depressive Disorder \|\| id:ieu-a-1188 | 173005 | rs112693590 | 0.20258638 | 0.03501706 | 7.2356E-09 |
| Body mass index (BMI) \|\| id:ukb-a-248 | Major Depressive Disorder \|\| id:ieu-a-1188 | 173005 | rs1127100 | 0.19973026 | 0.03491898 | 1.0664E-08 |
| Body mass index (BMI) \|\| id:ukb-a-248 | Major Depressive Disorder \|\| id:ieu-a-1188 | 173005 | rs113182412 | 0.20193493 | 0.03501714 | 8.0817E-09 |
| Body mass index (BMI) \|\| id:ukb-a-248 | Major Depressive Disorder \|\| id:ieu-a-1188 | 173005 | rs113230003 | 0.200399 | 0.03498105 | 1.0115E-08 |
| Body mass index (BMI) \|\| id:ukb-a-248 | Major Depressive Disorder \|\| id:ieu-a-1188 | 173005 | rs113603865 | 0.20236654 | 0.0350295 | 7.603E-09 |
| Body mass index (BMI) \|\| id:ukb-a-248 | Major Depressive Disorder \|\| id:ieu-a-1188 | 173005 | rs11515071 | 0.20214288 | 0.03507402 | 8.2474E-09 |
| Body mass index (BMI) \|\| id:ukb-a-248 | Major Depressive Disorder \|\| id:ieu-a-1188 | 173005 | rs11642015 | 0.19432609 | 0.03601876 | 6.8472E-08 |
| Body mass index (BMI) \|\| id:ukb-a-248 | Major Depressive Disorder \|\| id:ieu-a-1188 | 173005 | rs11655587 | 0.20391256 | 0.03500328 | 5.6933E-09 |
| Body mass index (BMI) \|\| id:ukb-a-248 | Major Depressive Disorder \|\| id:ieu-a-1188 | 173005 | rs11742930 | 0.20156286 | 0.03501569 | 8.5947E-09 |
| Body mass index (BMI) \|\| id:ukb-a-248 | Major Depressive Disorder \|\| id:ieu-a-1188 | 173005 | rs11757278 | 0.20126739 | 0.03499955 | 8.8943E-09 |
| Body mass index (BMI) \|\| id:ukb-a-248 | Major Depressive Disorder \|\| id:ieu-a-1188 | 173005 | rs11761411 | 0.20066616 | 0.03496667 | 9.5359E-09 |
| Body mass index (BMI) \|\| id:ukb-a-248 | Major Depressive Disorder \|\| id:ieu-a-1188 | 173005 | rs117632017 | 0.20091574 | 0.03496324 | 9.1117E-09 |
| Body mass index (BMI) \|\| id:ukb-a-248 | Major Depressive Disorder \|\| id:ieu-a-1188 | 173005 | rs11782074 | 0.20352001 | 0.03495874 | 5.8245E-09 |
| Body mass index (BMI) \|\| id:ukb-a-248 | Major Depressive Disorder \|\| id:ieu-a-1188 | 173005 | rs11856579 | 0.20065902 | 0.03501065 | 9.9622E-09 |
| Body mass index (BMI) \|\| id:ukb-a-248 | Major Depressive Disorder \|\| id:ieu-a-1188 | 173005 | rs12024554 | 0.20258343 | 0.03501112 | 7.1971E-09 |
| Body mass index (BMI) \|\| id:ukb-a-248 | Major Depressive Disorder \|\| id:ieu-a-1188 | 173005 | rs12042959 | 0.20224831 | 0.03502249 | 7.7039E-09 |
| Body mass index (BMI) \|\| id:ukb-a-248 | Major Depressive Disorder \|\| id:ieu-a-1188 | 173005 | rs12049202 | 0.19986875 | 0.03496742 | 1.0915E-08 |
| Body mass index (BMI) \|\| id:ukb-a-248 | Major Depressive Disorder \|\| id:ieu-a-1188 | 173005 | rs12140153 | 0.19884146 | 0.03489505 | 1.2103E-08 |
| Body mass index (BMI) \|\| id:ukb-a-248 | Major Depressive Disorder \|\| id:ieu-a-1188 | 173005 | rs12144626 | 0.19948609 | 0.03493009 | 1.1231E-08 |
| Body mass index (BMI) \|\| id:ukb-a-248 | Major Depressive Disorder \|\| id:ieu-a-1188 | 173005 | rs12477385 | 0.20127018 | 0.03500672 | 8.9522E-09 |
| Body mass index (BMI) \|\| id:ukb-a-248 | Major Depressive Disorder \|\| id:ieu-a-1188 | 173005 | rs12479357 | 0.19986214 | 0.03496307 | 1.0882E-08 |
| Body mass index (BMI) \|\| id:ukb-a-248 | Major Depressive Disorder \|\| id:ieu-a-1188 | 173005 | rs12614861 | 0.2011954 | 0.03500754 | 9.0732E-09 |
| Body mass index (BMI) \|\| id:ukb-a-248 | Major Depressive Disorder \|\| id:ieu-a-1188 | 173005 | rs12622280 | 0.20307534 | 0.03498541 | 6.4531E-09 |
| Body mass index (BMI) \|\| id:ukb-a-248 | Major Depressive Disorder \|\| id:ieu-a-1188 | 173005 | rs12679106 | 0.20598979 | 0.03487037 | 3.4776E-09 |
| Body mass index (BMI) \|\| id:ukb-a-248 | Major Depressive Disorder \|\| id:ieu-a-1188 | 173005 | rs1286138 | 0.20053158 | 0.03499809 | 1.0056E-08 |
| Body mass index (BMI) \|\| id:ukb-a-248 | Major Depressive Disorder \|\| id:ieu-a-1188 | 173005 | rs12877270 | 0.20227672 | 0.03503579 | 7.7674E-09 |
| Body mass index (BMI) \|\| id:ukb-a-248 | Major Depressive Disorder \|\| id:ieu-a-1188 | 173005 | rs12881629 | 0.20224794 | 0.03500832 | 7.5981E-09 |
| Body mass index (BMI) \|\| id:ukb-a-248 | Major Depressive Disorder \|\| id:ieu-a-1188 | 173005 | rs12885458 | 0.19874272 | 0.03482156 | 1.1467E-08 |
| Body mass index (BMI) \|\| id:ukb-a-248 | Major Depressive Disorder \|\| id:ieu-a-1188 | 173005 | rs1296328 | 0.2026009 | 0.0350454 | 7.4215E-09 |
| Body mass index (BMI) \|\| id:ukb-a-248 | Major Depressive Disorder \|\| id:ieu-a-1188 | 173005 | rs12977259 | 0.20222384 | 0.03501213 | 7.6578E-09 |
| Body mass index (BMI) \|\| id:ukb-a-248 | Major Depressive Disorder \|\| id:ieu-a-1188 | 173005 | rs12992672 | 0.19869132 | 0.03523983 | 1.7178E-08 |
| Body mass index (BMI) \|\| id:ukb-a-248 | Major Depressive Disorder \|\| id:ieu-a-1188 | 173005 | rs13062093 | 0.20166911 | 0.03503438 | 8.5967E-09 |
| Body mass index (BMI) \|\| id:ukb-a-248 | Major Depressive Disorder \|\| id:ieu-a-1188 | 173005 | rs13135092 | 0.20594144 | 0.03498703 | 3.9512E-09 |
| Body mass index (BMI) \|\| id:ukb-a-248 | Major Depressive Disorder \|\| id:ieu-a-1188 | 173005 | rs13174863 | 0.20301222 | 0.03502579 | 6.7884E-09 |
| Body mass index (BMI) \|\| id:ukb-a-248 | Major Depressive Disorder \|\| id:ieu-a-1188 | 173005 | rs1320903 | 0.20658188 | 0.03483136 | 3.0125E-09 |
| Body mass index (BMI) \|\| id:ukb-a-248 | Major Depressive Disorder \|\| id:ieu-a-1188 | 173005 | rs1327259 | 0.20266789 | 0.03499676 | 6.9949E-09 |
| Body mass index (BMI) \|\| id:ukb-a-248 | Major Depressive Disorder \|\| id:ieu-a-1188 | 173005 | rs1342391 | 0.20282843 | 0.03501167 | 6.9071E-09 |
| Body mass index (BMI) \|\| id:ukb-a-248 | Major Depressive Disorder \|\| id:ieu-a-1188 | 173005 | rs13427822 | 0.19994237 | 0.03497558 | 1.0866E-08 |
| Body mass index (BMI) \|\| id:ukb-a-248 | Major Depressive Disorder \|\| id:ieu-a-1188 | 173005 | rs1411432 | 0.19982758 | 0.03498767 | 1.1207E-08 |
| Body mass index (BMI) \|\| id:ukb-a-248 | Major Depressive Disorder \|\| id:ieu-a-1188 | 173005 | rs1441264 | 0.19927315 | 0.03497091 | 1.2104E-08 |
| Body mass index (BMI) \|\| id:ukb-a-248 | Major Depressive Disorder \|\| id:ieu-a-1188 | 173005 | rs1446585 | 0.20088238 | 0.03497283 | 9.249E-09 |
| Body mass index (BMI) \|\| id:ukb-a-248 | Major Depressive Disorder \|\| id:ieu-a-1188 | 173005 | rs1458156 | 0.20326208 | 0.03498062 | 6.221E-09 |
| Body mass index (BMI) \|\| id:ukb-a-248 | Major Depressive Disorder \|\| id:ieu-a-1188 | 173005 | rs1477290 | 0.19746129 | 0.03490428 | 1.5384E-08 |
| Body mass index (BMI) \|\| id:ukb-a-248 | Major Depressive Disorder \|\| id:ieu-a-1188 | 173005 | rs147730268 | 0.20303096 | 0.03503401 | 6.8218E-09 |
| Body mass index (BMI) \|\| id:ukb-a-248 | Major Depressive Disorder \|\| id:ieu-a-1188 | 173005 | rs1582931 | 0.20353696 | 0.03497615 | 5.9092E-09 |
| Body mass index (BMI) \|\| id:ukb-a-248 | Major Depressive Disorder \|\| id:ieu-a-1188 | 173005 | rs16846140 | 0.19897802 | 0.03484441 | 1.1266E-08 |
| Body mass index (BMI) \|\| id:ukb-a-248 | Major Depressive Disorder \|\| id:ieu-a-1188 | 173005 | rs16916303 | 0.20378219 | 0.03493173 | 5.4203E-09 |
| Body mass index (BMI) \|\| id:ukb-a-248 | Major Depressive Disorder \|\| id:ieu-a-1188 | 173005 | rs16975459 | 0.20223854 | 0.03502822 | 7.76E-09 |
| Body mass index (BMI) \|\| id:ukb-a-248 | Major Depressive Disorder \|\| id:ieu-a-1188 | 173005 | rs17014332 | 0.20139785 | 0.03501543 | 8.8356E-09 |
| Body mass index (BMI) \|\| id:ukb-a-248 | Major Depressive Disorder \|\| id:ieu-a-1188 | 173005 | rs17024393 | 0.19888068 | 0.03499875 | 1.3273E-08 |
| Body mass index (BMI) \|\| id:ukb-a-248 | Major Depressive Disorder \|\| id:ieu-a-1188 | 173005 | rs17058884 | 0.20313935 | 0.0349912 | 6.4197E-09 |
| Body mass index (BMI) \|\| id:ukb-a-248 | Major Depressive Disorder \|\| id:ieu-a-1188 | 173005 | rs17085463 | 0.20330865 | 0.03497563 | 6.1412E-09 |
| Body mass index (BMI) \|\| id:ukb-a-248 | Major Depressive Disorder \|\| id:ieu-a-1188 | 173005 | rs17149254 | 0.20319568 | 0.03489254 | 5.7638E-09 |
| Body mass index (BMI) \|\| id:ukb-a-248 | Major Depressive Disorder \|\| id:ieu-a-1188 | 173005 | rs17342242 | 0.20128279 | 0.03500361 | 8.9062E-09 |
| Body mass index (BMI) \|\| id:ukb-a-248 | Major Depressive Disorder \|\| id:ieu-a-1188 | 173005 | rs17399739 | 0.20493256 | 0.03479287 | 3.8602E-09 |
| Body mass index (BMI) \|\| id:ukb-a-248 | Major Depressive Disorder \|\| id:ieu-a-1188 | 173005 | rs17716502 | 0.20466506 | 0.03493742 | 4.6834E-09 |
| Body mass index (BMI) \|\| id:ukb-a-248 | Major Depressive Disorder \|\| id:ieu-a-1188 | 173005 | rs1788808 | 0.20683114 | 0.03472294 | 2.5752E-09 |
| Body mass index (BMI) \|\| id:ukb-a-248 | Major Depressive Disorder \|\| id:ieu-a-1188 | 173005 | rs1805123 | 0.20256607 | 0.0350194 | 7.2774E-09 |
| Body mass index (BMI) \|\| id:ukb-a-248 | Major Depressive Disorder \|\| id:ieu-a-1188 | 173005 | rs1884897 | 0.20430161 | 0.03500851 | 5.3543E-09 |
| Body mass index (BMI) \|\| id:ukb-a-248 | Major Depressive Disorder \|\| id:ieu-a-1188 | 173005 | rs1901241 | 0.20298785 | 0.03500089 | 6.6511E-09 |
| Body mass index (BMI) \|\| id:ukb-a-248 | Major Depressive Disorder \|\| id:ieu-a-1188 | 173005 | rs1919243 | 0.20393738 | 0.03492616 | 5.2483E-09 |
| Body mass index (BMI) \|\| id:ukb-a-248 | Major Depressive Disorder \|\| id:ieu-a-1188 | 173005 | rs1941706 | 0.1999201 | 0.03491246 | 1.0263E-08 |
| Body mass index (BMI) \|\| id:ukb-a-248 | Major Depressive Disorder \|\| id:ieu-a-1188 | 173005 | rs1949204 | 0.20069511 | 0.03497934 | 9.6065E-09 |
| Body mass index (BMI) \|\| id:ukb-a-248 | Major Depressive Disorder \|\| id:ieu-a-1188 | 173005 | rs2035806 | 0.20050908 | 0.03497709 | 9.8913E-09 |
| Body mass index (BMI) \|\| id:ukb-a-248 | Major Depressive Disorder \|\| id:ieu-a-1188 | 173005 | rs2046002 | 0.2017759 | 0.03501868 | 8.3148E-09 |
| Body mass index (BMI) \|\| id:ukb-a-248 | Major Depressive Disorder \|\| id:ieu-a-1188 | 173005 | rs2121058 | 0.20095623 | 0.03505064 | 9.8489E-09 |
| Body mass index (BMI) \|\| id:ukb-a-248 | Major Depressive Disorder \|\| id:ieu-a-1188 | 173005 | rs2155869 | 0.20078127 | 0.03498457 | 9.5159E-09 |
| Body mass index (BMI) \|\| id:ukb-a-248 | Major Depressive Disorder \|\| id:ieu-a-1188 | 173005 | rs215634 | 0.19905247 | 0.0348157 | 1.0822E-08 |
| Body mass index (BMI) \|\| id:ukb-a-248 | Major Depressive Disorder \|\| id:ieu-a-1188 | 173005 | rs217672 | 0.20113361 | 0.03499777 | 9.0819E-09 |
| Body mass index (BMI) \|\| id:ukb-a-248 | Major Depressive Disorder \|\| id:ieu-a-1188 | 173005 | rs2192649 | 0.2002923 | 0.03491416 | 9.6535E-09 |
| Body mass index (BMI) \|\| id:ukb-a-248 | Major Depressive Disorder \|\| id:ieu-a-1188 | 173005 | rs2234458 | 0.20245368 | 0.03505664 | 7.6932E-09 |
| Body mass index (BMI) \|\| id:ukb-a-248 | Major Depressive Disorder \|\| id:ieu-a-1188 | 173005 | rs2292238 | 0.201997 | 0.03504081 | 8.1842E-09 |
| Body mass index (BMI) \|\| id:ukb-a-248 | Major Depressive Disorder \|\| id:ieu-a-1188 | 173005 | rs2307111 | 0.20193816 | 0.03514789 | 9.1712E-09 |
| Body mass index (BMI) \|\| id:ukb-a-248 | Major Depressive Disorder \|\| id:ieu-a-1188 | 173005 | rs2318543 | 0.19974706 | 0.03493949 | 1.0846E-08 |
| Body mass index (BMI) \|\| id:ukb-a-248 | Major Depressive Disorder \|\| id:ieu-a-1188 | 173005 | rs2384054 | 0.2031326 | 0.03522837 | 8.1095E-09 |
| Body mass index (BMI) \|\| id:ukb-a-248 | Major Depressive Disorder \|\| id:ieu-a-1188 | 173005 | rs2398861 | 0.19864231 | 0.03491379 | 1.274E-08 |
| Body mass index (BMI) \|\| id:ukb-a-248 | Major Depressive Disorder \|\| id:ieu-a-1188 | 173005 | rs241460 | 0.20188715 | 0.03505423 | 8.4473E-09 |
| Body mass index (BMI) \|\| id:ukb-a-248 | Major Depressive Disorder \|\| id:ieu-a-1188 | 173005 | rs2425857 | 0.1992936 | 0.03482762 | 1.051E-08 |
| Body mass index (BMI) \|\| id:ukb-a-248 | Major Depressive Disorder \|\| id:ieu-a-1188 | 173005 | rs2439823 | 0.20295604 | 0.03506444 | 7.1192E-09 |
| Body mass index (BMI) \|\| id:ukb-a-248 | Major Depressive Disorder \|\| id:ieu-a-1188 | 173005 | rs2450445 | 0.19992633 | 0.0349201 | 1.0328E-08 |
| Body mass index (BMI) \|\| id:ukb-a-248 | Major Depressive Disorder \|\| id:ieu-a-1188 | 173005 | rs245775 | 0.20172389 | 0.03504523 | 8.6079E-09 |
| Body mass index (BMI) \|\| id:ukb-a-248 | Major Depressive Disorder \|\| id:ieu-a-1188 | 173005 | rs2470392 | 0.20112274 | 0.03499501 | 9.0743E-09 |
| Body mass index (BMI) \|\| id:ukb-a-248 | Major Depressive Disorder \|\| id:ieu-a-1188 | 173005 | rs2474898 | 0.20385771 | 0.03493734 | 5.3806E-09 |
| Body mass index (BMI) \|\| id:ukb-a-248 | Major Depressive Disorder \|\| id:ieu-a-1188 | 173005 | rs2482704 | 0.20131178 | 0.03500521 | 8.8765E-09 |
| Body mass index (BMI) \|\| id:ukb-a-248 | Major Depressive Disorder \|\| id:ieu-a-1188 | 173005 | rs2616192 | 0.19961056 | 0.03485921 | 1.0271E-08 |
| Body mass index (BMI) \|\| id:ukb-a-248 | Major Depressive Disorder \|\| id:ieu-a-1188 | 173005 | rs2678204 | 0.1987389 | 0.03505066 | 1.4276E-08 |
| Body mass index (BMI) \|\| id:ukb-a-248 | Major Depressive Disorder \|\| id:ieu-a-1188 | 173005 | rs2711111 | 0.20433368 | 0.03488754 | 4.7152E-09 |
| Body mass index (BMI) \|\| id:ukb-a-248 | Major Depressive Disorder \|\| id:ieu-a-1188 | 173005 | rs2725371 | 0.20147324 | 0.03502979 | 8.8464E-09 |
| Body mass index (BMI) \|\| id:ukb-a-248 | Major Depressive Disorder \|\| id:ieu-a-1188 | 173005 | rs273505 | 0.20237579 | 0.03504239 | 7.6875E-09 |
| Body mass index (BMI) \|\| id:ukb-a-248 | Major Depressive Disorder \|\| id:ieu-a-1188 | 173005 | rs28447555 | 0.20417295 | 0.03490709 | 4.9442E-09 |
| Body mass index (BMI) \|\| id:ukb-a-248 | Major Depressive Disorder \|\| id:ieu-a-1188 | 173005 | rs28489620 | 0.20168773 | 0.03501088 | 8.3758E-09 |
| Body mass index (BMI) \|\| id:ukb-a-248 | Major Depressive Disorder \|\| id:ieu-a-1188 | 173005 | rs2861685 | 0.20309731 | 0.03502212 | 6.6666E-09 |
| Body mass index (BMI) \|\| id:ukb-a-248 | Major Depressive Disorder \|\| id:ieu-a-1188 | 173005 | rs2962082 | 0.19990544 | 0.03490212 | 1.0186E-08 |
| Body mass index (BMI) \|\| id:ukb-a-248 | Major Depressive Disorder \|\| id:ieu-a-1188 | 173005 | rs2975693 | 0.20312208 | 0.03499664 | 6.4734E-09 |
| Body mass index (BMI) \|\| id:ukb-a-248 | Major Depressive Disorder \|\| id:ieu-a-1188 | 173005 | rs34045288 | 0.20498211 | 0.0350337 | 4.8863E-09 |
| Body mass index (BMI) \|\| id:ukb-a-248 | Major Depressive Disorder \|\| id:ieu-a-1188 | 173005 | rs34095326 | 0.20101502 | 0.03499365 | 9.2286E-09 |
| Body mass index (BMI) \|\| id:ukb-a-248 | Major Depressive Disorder \|\| id:ieu-a-1188 | 173005 | rs34236292 | 0.20202853 | 0.03501494 | 7.9375E-09 |
| Body mass index (BMI) \|\| id:ukb-a-248 | Major Depressive Disorder \|\| id:ieu-a-1188 | 173005 | rs34373881 | 0.2022148 | 0.03502608 | 7.7751E-09 |
| Body mass index (BMI) \|\| id:ukb-a-248 | Major Depressive Disorder \|\| id:ieu-a-1188 | 173005 | rs34774377 | 0.20250616 | 0.0350034 | 7.2371E-09 |
| Body mass index (BMI) \|\| id:ukb-a-248 | Major Depressive Disorder \|\| id:ieu-a-1188 | 173005 | rs34811474 | 0.20244746 | 0.03510596 | 8.0812E-09 |
| Body mass index (BMI) \|\| id:ukb-a-248 | Major Depressive Disorder \|\| id:ieu-a-1188 | 173005 | rs34966008 | 0.20423774 | 0.0349994 | 5.3641E-09 |
| Body mass index (BMI) \|\| id:ukb-a-248 | Major Depressive Disorder \|\| id:ieu-a-1188 | 173005 | rs35193668 | 0.20187177 | 0.03503224 | 8.2898E-09 |
| Body mass index (BMI) \|\| id:ukb-a-248 | Major Depressive Disorder \|\| id:ieu-a-1188 | 173005 | rs35483388 | 0.20331275 | 0.03497486 | 6.1322E-09 |
| Body mass index (BMI) \|\| id:ukb-a-248 | Major Depressive Disorder \|\| id:ieu-a-1188 | 173005 | rs35626515 | 0.20622025 | 0.034988 | 3.7689E-09 |
| Body mass index (BMI) \|\| id:ukb-a-248 | Major Depressive Disorder \|\| id:ieu-a-1188 | 173005 | rs35722922 | 0.20295748 | 0.03501494 | 6.779E-09 |
| Body mass index (BMI) \|\| id:ukb-a-248 | Major Depressive Disorder \|\| id:ieu-a-1188 | 173005 | rs357501 | 0.20107006 | 0.03500552 | 9.2492E-09 |
| Body mass index (BMI) \|\| id:ukb-a-248 | Major Depressive Disorder \|\| id:ieu-a-1188 | 173005 | rs35851183 | 0.20312146 | 0.03500958 | 6.5575E-09 |
| Body mass index (BMI) \|\| id:ukb-a-248 | Major Depressive Disorder \|\| id:ieu-a-1188 | 173005 | rs35882248 | 0.20410295 | 0.03498149 | 5.392E-09 |
| Body mass index (BMI) \|\| id:ukb-a-248 | Major Depressive Disorder \|\| id:ieu-a-1188 | 173005 | rs36007635 | 0.20080043 | 0.03498122 | 9.4545E-09 |
| Body mass index (BMI) \|\| id:ukb-a-248 | Major Depressive Disorder \|\| id:ieu-a-1188 | 173005 | rs362307 | 0.20015533 | 0.03497089 | 1.0436E-08 |
| Body mass index (BMI) \|\| id:ukb-a-248 | Major Depressive Disorder \|\| id:ieu-a-1188 | 173005 | rs3759584 | 0.20623567 | 0.03469417 | 2.7749E-09 |
| Body mass index (BMI) \|\| id:ukb-a-248 | Major Depressive Disorder \|\| id:ieu-a-1188 | 173005 | rs3802858 | 0.20300587 | 0.03501677 | 6.7355E-09 |
| Body mass index (BMI) \|\| id:ukb-a-248 | Major Depressive Disorder \|\| id:ieu-a-1188 | 173005 | rs3803286 | 0.20178014 | 0.03505936 | 8.6444E-09 |
| Body mass index (BMI) \|\| id:ukb-a-248 | Major Depressive Disorder \|\| id:ieu-a-1188 | 173005 | rs3810291 | 0.20490286 | 0.03507555 | 5.1653E-09 |
| Body mass index (BMI) \|\| id:ukb-a-248 | Major Depressive Disorder \|\| id:ieu-a-1188 | 173005 | rs3843540 | 0.20015118 | 0.03495508 | 1.0285E-08 |
| Body mass index (BMI) \|\| id:ukb-a-248 | Major Depressive Disorder \|\| id:ieu-a-1188 | 173005 | rs3861879 | 0.20209617 | 0.03501731 | 7.8652E-09 |
| Body mass index (BMI) \|\| id:ukb-a-248 | Major Depressive Disorder \|\| id:ieu-a-1188 | 173005 | rs3897102 | 0.2031921 | 0.03498548 | 6.3262E-09 |
| Body mass index (BMI) \|\| id:ukb-a-248 | Major Depressive Disorder \|\| id:ieu-a-1188 | 173005 | rs390192 | 0.20181397 | 0.03502652 | 8.3248E-09 |
| Body mass index (BMI) \|\| id:ukb-a-248 | Major Depressive Disorder \|\| id:ieu-a-1188 | 173005 | rs4246657 | 0.20200986 | 0.03503344 | 8.1078E-09 |
| Body mass index (BMI) \|\| id:ukb-a-248 | Major Depressive Disorder \|\| id:ieu-a-1188 | 173005 | rs4261944 | 0.20279873 | 0.03501124 | 6.9392E-09 |
| Body mass index (BMI) \|\| id:ukb-a-248 | Major Depressive Disorder \|\| id:ieu-a-1188 | 173005 | rs4402589 | 0.20956913 | 0.03481431 | 1.7482E-09 |
| Body mass index (BMI) \|\| id:ukb-a-248 | Major Depressive Disorder \|\| id:ieu-a-1188 | 173005 | rs4467770 | 0.20463207 | 0.03485338 | 4.3258E-09 |
| Body mass index (BMI) \|\| id:ukb-a-248 | Major Depressive Disorder \|\| id:ieu-a-1188 | 173005 | rs4474229 | 0.20124572 | 0.03501707 | 9.08E-09 |
| Body mass index (BMI) \|\| id:ukb-a-248 | Major Depressive Disorder \|\| id:ieu-a-1188 | 173005 | rs4482463 | 0.20518197 | 0.03490843 | 4.1595E-09 |
| Body mass index (BMI) \|\| id:ukb-a-248 | Major Depressive Disorder \|\| id:ieu-a-1188 | 173005 | rs4502882 | 0.19936577 | 0.03485043 | 1.0614E-08 |
| Body mass index (BMI) \|\| id:ukb-a-248 | Major Depressive Disorder \|\| id:ieu-a-1188 | 173005 | rs4595495 | 0.2009531 | 0.03498825 | 9.2769E-09 |
| Body mass index (BMI) \|\| id:ukb-a-248 | Major Depressive Disorder \|\| id:ieu-a-1188 | 173005 | rs4648450 | 0.20497145 | 0.034834 | 3.999E-09 |
| Body mass index (BMI) \|\| id:ukb-a-248 | Major Depressive Disorder \|\| id:ieu-a-1188 | 173005 | rs4671328 | 0.20614924 | 0.03488772 | 3.4433E-09 |
| Body mass index (BMI) \|\| id:ukb-a-248 | Major Depressive Disorder \|\| id:ieu-a-1188 | 173005 | rs4687770 | 0.20341895 | 0.03496235 | 5.9475E-09 |
| Body mass index (BMI) \|\| id:ukb-a-248 | Major Depressive Disorder \|\| id:ieu-a-1188 | 173005 | rs4718964 | 0.20045629 | 0.03496391 | 9.8533E-09 |
| Body mass index (BMI) \|\| id:ukb-a-248 | Major Depressive Disorder \|\| id:ieu-a-1188 | 173005 | rs4757144 | 0.20359444 | 0.0349882 | 5.922E-09 |
| Body mass index (BMI) \|\| id:ukb-a-248 | Major Depressive Disorder \|\| id:ieu-a-1188 | 173005 | rs4777541 | 0.20128486 | 0.0350249 | 9.0891E-09 |
| Body mass index (BMI) \|\| id:ukb-a-248 | Major Depressive Disorder \|\| id:ieu-a-1188 | 173005 | rs4790841 | 0.20369289 | 0.03503636 | 6.1086E-09 |
| Body mass index (BMI) \|\| id:ukb-a-248 | Major Depressive Disorder \|\| id:ieu-a-1188 | 173005 | rs4911382 | 0.1998624 | 0.03493508 | 1.0592E-08 |
| Body mass index (BMI) \|\| id:ukb-a-248 | Major Depressive Disorder \|\| id:ieu-a-1188 | 173005 | rs491711 | 0.20249606 | 0.03501704 | 7.3473E-09 |
| Body mass index (BMI) \|\| id:ukb-a-248 | Major Depressive Disorder \|\| id:ieu-a-1188 | 173005 | rs4921301 | 0.20144911 | 0.03501578 | 8.7624E-09 |
| Body mass index (BMI) \|\| id:ukb-a-248 | Major Depressive Disorder \|\| id:ieu-a-1188 | 173005 | rs4929923 | 0.20015344 | 0.03497867 | 1.0518E-08 |
| Body mass index (BMI) \|\| id:ukb-a-248 | Major Depressive Disorder \|\| id:ieu-a-1188 | 173005 | rs525101 | 0.19946602 | 0.03490118 | 1.096E-08 |
| Body mass index (BMI) \|\| id:ukb-a-248 | Major Depressive Disorder \|\| id:ieu-a-1188 | 173005 | rs539515 | 0.20146945 | 0.03528197 | 1.128E-08 |
| Body mass index (BMI) \|\| id:ukb-a-248 | Major Depressive Disorder \|\| id:ieu-a-1188 | 173005 | rs55689274 | 0.2033225 | 0.03497121 | 6.0999E-09 |
| Body mass index (BMI) \|\| id:ukb-a-248 | Major Depressive Disorder \|\| id:ieu-a-1188 | 173005 | rs55726687 | 0.20275337 | 0.03504884 | 7.2564E-09 |
| Body mass index (BMI) \|\| id:ukb-a-248 | Major Depressive Disorder \|\| id:ieu-a-1188 | 173005 | rs55938344 | 0.19942202 | 0.0348655 | 1.0668E-08 |
| Body mass index (BMI) \|\| id:ukb-a-248 | Major Depressive Disorder \|\| id:ieu-a-1188 | 173005 | rs56212061 | 0.20195741 | 0.0350243 | 8.1075E-09 |
| Body mass index (BMI) \|\| id:ukb-a-248 | Major Depressive Disorder \|\| id:ieu-a-1188 | 173005 | rs56803094 | 0.19882271 | 0.0348397 | 1.1513E-08 |
| Body mass index (BMI) \|\| id:ukb-a-248 | Major Depressive Disorder \|\| id:ieu-a-1188 | 173005 | rs57636386 | 0.20711106 | 0.03481114 | 2.6886E-09 |
| Body mass index (BMI) \|\| id:ukb-a-248 | Major Depressive Disorder \|\| id:ieu-a-1188 | 173005 | rs58862095 | 0.20011926 | 0.03504878 | 1.1315E-08 |
| Body mass index (BMI) \|\| id:ukb-a-248 | Major Depressive Disorder \|\| id:ieu-a-1188 | 173005 | rs588660 | 0.20449023 | 0.03495895 | 4.9323E-09 |
| Body mass index (BMI) \|\| id:ukb-a-248 | Major Depressive Disorder \|\| id:ieu-a-1188 | 173005 | rs59104534 | 0.2034847 | 0.03496323 | 5.8861E-09 |
| Body mass index (BMI) \|\| id:ukb-a-248 | Major Depressive Disorder \|\| id:ieu-a-1188 | 173005 | rs5995843 | 0.20555431 | 0.03482927 | 3.5962E-09 |
| Body mass index (BMI) \|\| id:ukb-a-248 | Major Depressive Disorder \|\| id:ieu-a-1188 | 173005 | rs6050446 | 0.20096712 | 0.03499515 | 9.3171E-09 |
| Body mass index (BMI) \|\| id:ukb-a-248 | Major Depressive Disorder \|\| id:ieu-a-1188 | 173005 | rs60654199 | 0.20441932 | 0.03487451 | 4.5851E-09 |
| Body mass index (BMI) \|\| id:ukb-a-248 | Major Depressive Disorder \|\| id:ieu-a-1188 | 173005 | rs60764613 | 0.19996529 | 0.03496226 | 1.0686E-08 |
| Body mass index (BMI) \|\| id:ukb-a-248 | Major Depressive Disorder \|\| id:ieu-a-1188 | 173005 | rs61813324 | 0.20235474 | 0.0350419 | 7.7113E-09 |
| Body mass index (BMI) \|\| id:ukb-a-248 | Major Depressive Disorder \|\| id:ieu-a-1188 | 173005 | rs61826867 | 0.20236021 | 0.03502933 | 7.6099E-09 |
| Body mass index (BMI) \|\| id:ukb-a-248 | Major Depressive Disorder \|\| id:ieu-a-1188 | 173005 | rs61871615 | 0.20231847 | 0.03499736 | 7.4274E-09 |
| Body mass index (BMI) \|\| id:ukb-a-248 | Major Depressive Disorder \|\| id:ieu-a-1188 | 173005 | rs61903695 | 0.20311949 | 0.03499299 | 6.4529E-09 |
| Body mass index (BMI) \|\| id:ukb-a-248 | Major Depressive Disorder \|\| id:ieu-a-1188 | 173005 | rs61969510 | 0.20060664 | 0.03497109 | 9.6735E-09 |
| Body mass index (BMI) \|\| id:ukb-a-248 | Major Depressive Disorder \|\| id:ieu-a-1188 | 173005 | rs62106258 | 0.20594009 | 0.03519218 | 4.8607E-09 |
| Body mass index (BMI) \|\| id:ukb-a-248 | Major Depressive Disorder \|\| id:ieu-a-1188 | 173005 | rs62147189 | 0.20273396 | 0.03502001 | 7.0769E-09 |
| Body mass index (BMI) \|\| id:ukb-a-248 | Major Depressive Disorder \|\| id:ieu-a-1188 | 173005 | rs62246314 | 0.20557957 | 0.03470764 | 3.1578E-09 |
| Body mass index (BMI) \|\| id:ukb-a-248 | Major Depressive Disorder \|\| id:ieu-a-1188 | 173005 | rs6265 | 0.21111106 | 0.03481492 | 1.3293E-09 |
| Body mass index (BMI) \|\| id:ukb-a-248 | Major Depressive Disorder \|\| id:ieu-a-1188 | 173005 | rs6536575 | 0.19906611 | 0.0348118 | 1.0756E-08 |
| Body mass index (BMI) \|\| id:ukb-a-248 | Major Depressive Disorder \|\| id:ieu-a-1188 | 173005 | rs6575340 | 0.19910731 | 0.0349928 | 1.2708E-08 |
| Body mass index (BMI) \|\| id:ukb-a-248 | Major Depressive Disorder \|\| id:ieu-a-1188 | 173005 | rs6601527 | 0.20235883 | 0.03507079 | 7.9267E-09 |
| Body mass index (BMI) \|\| id:ukb-a-248 | Major Depressive Disorder \|\| id:ieu-a-1188 | 173005 | rs66679256 | 0.2036576 | 0.03498424 | 5.8354E-09 |
| Body mass index (BMI) \|\| id:ukb-a-248 | Major Depressive Disorder \|\| id:ieu-a-1188 | 173005 | rs6687953 | 0.20149816 | 0.035025 | 8.7683E-09 |
| Body mass index (BMI) \|\| id:ukb-a-248 | Major Depressive Disorder \|\| id:ieu-a-1188 | 173005 | rs66922415 | 0.21378703 | 0.03507241 | 1.0903E-09 |
| Body mass index (BMI) \|\| id:ukb-a-248 | Major Depressive Disorder \|\| id:ieu-a-1188 | 173005 | rs6705567 | 0.20086082 | 0.03497819 | 9.3312E-09 |
| Body mass index (BMI) \|\| id:ukb-a-248 | Major Depressive Disorder \|\| id:ieu-a-1188 | 173005 | rs6722241 | 0.20028919 | 0.03499654 | 1.0459E-08 |
| Body mass index (BMI) \|\| id:ukb-a-248 | Major Depressive Disorder \|\| id:ieu-a-1188 | 173005 | rs6739755 | 0.20156221 | 0.03506309 | 9.0007E-09 |
| Body mass index (BMI) \|\| id:ukb-a-248 | Major Depressive Disorder \|\| id:ieu-a-1188 | 173005 | rs67609008 | 0.19966395 | 0.03483642 | 9.9563E-09 |
| Body mass index (BMI) \|\| id:ukb-a-248 | Major Depressive Disorder \|\| id:ieu-a-1188 | 173005 | rs67844506 | 0.20074788 | 0.03503954 | 1.0092E-08 |
| Body mass index (BMI) \|\| id:ukb-a-248 | Major Depressive Disorder \|\| id:ieu-a-1188 | 173005 | rs6789488 | 0.2008894 | 0.03501982 | 9.6683E-09 |
| Body mass index (BMI) \|\| id:ukb-a-248 | Major Depressive Disorder \|\| id:ieu-a-1188 | 173005 | rs6809307 | 0.20315458 | 0.0349854 | 6.3663E-09 |
| Body mass index (BMI) \|\| id:ukb-a-248 | Major Depressive Disorder \|\| id:ieu-a-1188 | 173005 | rs6831020 | 0.20002843 | 0.03492417 | 1.0192E-08 |
| Body mass index (BMI) \|\| id:ukb-a-248 | Major Depressive Disorder \|\| id:ieu-a-1188 | 173005 | rs6861649 | 0.20123935 | 0.03500515 | 8.9853E-09 |
| Body mass index (BMI) \|\| id:ukb-a-248 | Major Depressive Disorder \|\| id:ieu-a-1188 | 173005 | rs6950388 | 0.20015237 | 0.034878 | 9.5438E-09 |
| Body mass index (BMI) \|\| id:ukb-a-248 | Major Depressive Disorder \|\| id:ieu-a-1188 | 173005 | rs7030732 | 0.20214738 | 0.03502312 | 7.8417E-09 |
| Body mass index (BMI) \|\| id:ukb-a-248 | Major Depressive Disorder \|\| id:ieu-a-1188 | 173005 | rs704061 | 0.19875185 | 0.03483112 | 1.1555E-08 |
| Body mass index (BMI) \|\| id:ukb-a-248 | Major Depressive Disorder \|\| id:ieu-a-1188 | 173005 | rs7094644 | 0.20286581 | 0.03500314 | 6.8059E-09 |
| Body mass index (BMI) \|\| id:ukb-a-248 | Major Depressive Disorder \|\| id:ieu-a-1188 | 173005 | rs7116641 | 0.20562434 | 0.03496706 | 4.0899E-09 |
| Body mass index (BMI) \|\| id:ukb-a-248 | Major Depressive Disorder \|\| id:ieu-a-1188 | 173005 | rs7124681 | 0.20690613 | 0.03496242 | 3.2596E-09 |
| Body mass index (BMI) \|\| id:ukb-a-248 | Major Depressive Disorder \|\| id:ieu-a-1188 | 173005 | rs7132908 | 0.20057285 | 0.03512633 | 1.1295E-08 |
| Body mass index (BMI) \|\| id:ukb-a-248 | Major Depressive Disorder \|\| id:ieu-a-1188 | 173005 | rs7138383 | 0.20225676 | 0.03505123 | 7.9122E-09 |
| Body mass index (BMI) \|\| id:ukb-a-248 | Major Depressive Disorder \|\| id:ieu-a-1188 | 173005 | rs7141420 | 0.20344038 | 0.03504682 | 6.4436E-09 |
| Body mass index (BMI) \|\| id:ukb-a-248 | Major Depressive Disorder \|\| id:ieu-a-1188 | 173005 | rs71495049 | 0.20217232 | 0.03502397 | 7.8151E-09 |
| Body mass index (BMI) \|\| id:ukb-a-248 | Major Depressive Disorder \|\| id:ieu-a-1188 | 173005 | rs7183417 | 0.20030557 | 0.03496005 | 1.0069E-08 |
| Body mass index (BMI) \|\| id:ukb-a-248 | Major Depressive Disorder \|\| id:ieu-a-1188 | 173005 | rs7195386 | 0.20101901 | 0.03501267 | 9.3941E-09 |
| Body mass index (BMI) \|\| id:ukb-a-248 | Major Depressive Disorder \|\| id:ieu-a-1188 | 173005 | rs7201895 | 0.19976704 | 0.03492715 | 1.0682E-08 |
| Body mass index (BMI) \|\| id:ukb-a-248 | Major Depressive Disorder \|\| id:ieu-a-1188 | 173005 | rs7218014 | 0.19882138 | 0.03486339 | 1.1781E-08 |
| Body mass index (BMI) \|\| id:ukb-a-248 | Major Depressive Disorder \|\| id:ieu-a-1188 | 173005 | rs72697614 | 0.19904754 | 0.0347787 | 1.045E-08 |
| Body mass index (BMI) \|\| id:ukb-a-248 | Major Depressive Disorder \|\| id:ieu-a-1188 | 173005 | rs72820274 | 0.20038459 | 0.03498039 | 1.0133E-08 |
| Body mass index (BMI) \|\| id:ukb-a-248 | Major Depressive Disorder \|\| id:ieu-a-1188 | 173005 | rs72892910 | 0.19819526 | 0.03509771 | 1.6331E-08 |
| Body mass index (BMI) \|\| id:ukb-a-248 | Major Depressive Disorder \|\| id:ieu-a-1188 | 173005 | rs72976986 | 0.20198911 | 0.03503666 | 8.1619E-09 |
| Body mass index (BMI) \|\| id:ukb-a-248 | Major Depressive Disorder \|\| id:ieu-a-1188 | 173005 | rs73050254 | 0.20101971 | 0.03498901 | 9.1799E-09 |
| Body mass index (BMI) \|\| id:ukb-a-248 | Major Depressive Disorder \|\| id:ieu-a-1188 | 173005 | rs73144053 | 0.20236397 | 0.03501879 | 7.5269E-09 |
| Body mass index (BMI) \|\| id:ukb-a-248 | Major Depressive Disorder \|\| id:ieu-a-1188 | 173005 | rs73169730 | 0.19915391 | 0.03491112 | 1.1662E-08 |
| Body mass index (BMI) \|\| id:ukb-a-248 | Major Depressive Disorder \|\| id:ieu-a-1188 | 173005 | rs7321331 | 0.20454382 | 0.03489447 | 4.5792E-09 |
| Body mass index (BMI) \|\| id:ukb-a-248 | Major Depressive Disorder \|\| id:ieu-a-1188 | 173005 | rs7331420 | 0.20343938 | 0.03496359 | 5.934E-09 |
| Body mass index (BMI) \|\| id:ukb-a-248 | Major Depressive Disorder \|\| id:ieu-a-1188 | 173005 | rs7498044 | 0.20309131 | 0.03499466 | 6.4947E-09 |
| Body mass index (BMI) \|\| id:ukb-a-248 | Major Depressive Disorder \|\| id:ieu-a-1188 | 173005 | rs750090 | 0.20242011 | 0.03502061 | 7.4688E-09 |
| Body mass index (BMI) \|\| id:ukb-a-248 | Major Depressive Disorder \|\| id:ieu-a-1188 | 173005 | rs75499503 | 0.20483716 | 0.03488509 | 4.3117E-09 |
| Body mass index (BMI) \|\| id:ukb-a-248 | Major Depressive Disorder \|\| id:ieu-a-1188 | 173005 | rs7553158 | 0.2014087 | 0.03503439 | 8.9834E-09 |
| Body mass index (BMI) \|\| id:ukb-a-248 | Major Depressive Disorder \|\| id:ieu-a-1188 | 173005 | rs75557510 | 0.20061057 | 0.03501257 | 1.0062E-08 |
| Body mass index (BMI) \|\| id:ukb-a-248 | Major Depressive Disorder \|\| id:ieu-a-1188 | 173005 | rs756717 | 0.20198537 | 0.03502066 | 8.0406E-09 |
| Body mass index (BMI) \|\| id:ukb-a-248 | Major Depressive Disorder \|\| id:ieu-a-1188 | 173005 | rs76040172 | 0.19901488 | 0.03490341 | 1.185E-08 |
| Body mass index (BMI) \|\| id:ukb-a-248 | Major Depressive Disorder \|\| id:ieu-a-1188 | 173005 | rs7701777 | 0.20079186 | 0.03499716 | 9.6155E-09 |
| Body mass index (BMI) \|\| id:ukb-a-248 | Major Depressive Disorder \|\| id:ieu-a-1188 | 173005 | rs7719067 | 0.20433042 | 0.03493423 | 4.9453E-09 |
| Body mass index (BMI) \|\| id:ukb-a-248 | Major Depressive Disorder \|\| id:ieu-a-1188 | 173005 | rs7723426 | 0.19957226 | 0.03487009 | 1.0447E-08 |
| Body mass index (BMI) \|\| id:ukb-a-248 | Major Depressive Disorder \|\| id:ieu-a-1188 | 173005 | rs7755574 | 0.20292449 | 0.03500098 | 6.7238E-09 |
| Body mass index (BMI) \|\| id:ukb-a-248 | Major Depressive Disorder \|\| id:ieu-a-1188 | 173005 | rs7774 | 0.20171002 | 0.03502232 | 8.4379E-09 |
| Body mass index (BMI) \|\| id:ukb-a-248 | Major Depressive Disorder \|\| id:ieu-a-1188 | 173005 | rs778094 | 0.20453142 | 0.03488593 | 4.5496E-09 |
| Body mass index (BMI) \|\| id:ukb-a-248 | Major Depressive Disorder \|\| id:ieu-a-1188 | 173005 | rs7852189 | 0.20036619 | 0.03497618 | 1.0123E-08 |
| Body mass index (BMI) \|\| id:ukb-a-248 | Major Depressive Disorder \|\| id:ieu-a-1188 | 173005 | rs78565420 | 0.20319521 | 0.03497044 | 6.2292E-09 |
| Body mass index (BMI) \|\| id:ukb-a-248 | Major Depressive Disorder \|\| id:ieu-a-1188 | 173005 | rs79113395 | 0.20267206 | 0.0349985 | 7.002E-09 |
| Body mass index (BMI) \|\| id:ukb-a-248 | Major Depressive Disorder \|\| id:ieu-a-1188 | 173005 | rs7933085 | 0.20169462 | 0.03502716 | 8.4998E-09 |
| Body mass index (BMI) \|\| id:ukb-a-248 | Major Depressive Disorder \|\| id:ieu-a-1188 | 173005 | rs7941828 | 0.20191797 | 0.03502363 | 8.1565E-09 |
| Body mass index (BMI) \|\| id:ukb-a-248 | Major Depressive Disorder \|\| id:ieu-a-1188 | 173005 | rs7952102 | 0.20359039 | 0.03495836 | 5.7526E-09 |
| Body mass index (BMI) \|\| id:ukb-a-248 | Major Depressive Disorder \|\| id:ieu-a-1188 | 173005 | rs7992832 | 0.19989111 | 0.03493837 | 1.0574E-08 |
| Body mass index (BMI) \|\| id:ukb-a-248 | Major Depressive Disorder \|\| id:ieu-a-1188 | 173005 | rs799449 | 0.20535685 | 0.03491807 | 4.0753E-09 |
| Body mass index (BMI) \|\| id:ukb-a-248 | Major Depressive Disorder \|\| id:ieu-a-1188 | 173005 | rs8015400 | 0.20151361 | 0.03505346 | 8.9906E-09 |
| Body mass index (BMI) \|\| id:ukb-a-248 | Major Depressive Disorder \|\| id:ieu-a-1188 | 173005 | rs80330591 | 0.20187542 | 0.03502583 | 8.233E-09 |
| Body mass index (BMI) \|\| id:ukb-a-248 | Major Depressive Disorder \|\| id:ieu-a-1188 | 173005 | rs8078135 | 0.20169523 | 0.03504556 | 8.6524E-09 |
| Body mass index (BMI) \|\| id:ukb-a-248 | Major Depressive Disorder \|\| id:ieu-a-1188 | 173005 | rs8134638 | 0.20638401 | 0.03460204 | 2.4538E-09 |
| Body mass index (BMI) \|\| id:ukb-a-248 | Major Depressive Disorder \|\| id:ieu-a-1188 | 173005 | rs815163 | 0.20347797 | 0.03501561 | 6.2077E-09 |
| Body mass index (BMI) \|\| id:ukb-a-248 | Major Depressive Disorder \|\| id:ieu-a-1188 | 173005 | rs845084 | 0.20261163 | 0.03503997 | 7.3686E-09 |
| Body mass index (BMI) \|\| id:ukb-a-248 | Major Depressive Disorder \|\| id:ieu-a-1188 | 173005 | rs862320 | 0.19781687 | 0.03491999 | 1.4714E-08 |
| Body mass index (BMI) \|\| id:ukb-a-248 | Major Depressive Disorder \|\| id:ieu-a-1188 | 173005 | rs869400 | 0.19964162 | 0.03504904 | 1.226E-08 |
| Body mass index (BMI) \|\| id:ukb-a-248 | Major Depressive Disorder \|\| id:ieu-a-1188 | 173005 | rs879620 | 0.20630742 | 0.03492484 | 3.4801E-09 |
| Body mass index (BMI) \|\| id:ukb-a-248 | Major Depressive Disorder \|\| id:ieu-a-1188 | 173005 | rs9267671 | 0.20078472 | 0.03499155 | 9.575E-09 |
| Body mass index (BMI) \|\| id:ukb-a-248 | Major Depressive Disorder \|\| id:ieu-a-1188 | 173005 | rs9291822 | 0.20392581 | 0.03492236 | 5.2388E-09 |
| Body mass index (BMI) \|\| id:ukb-a-248 | Major Depressive Disorder \|\| id:ieu-a-1188 | 173005 | rs9320823 | 0.20344175 | 0.03502194 | 6.2855E-09 |
| Body mass index (BMI) \|\| id:ukb-a-248 | Major Depressive Disorder \|\| id:ieu-a-1188 | 173005 | rs9342196 | 0.20301664 | 0.03498286 | 6.5016E-09 |
| Body mass index (BMI) \|\| id:ukb-a-248 | Major Depressive Disorder \|\| id:ieu-a-1188 | 173005 | rs935166 | 0.20159817 | 0.03501404 | 8.5299E-09 |
| Body mass index (BMI) \|\| id:ukb-a-248 | Major Depressive Disorder \|\| id:ieu-a-1188 | 173005 | rs9402104 | 0.20116845 | 0.03499604 | 9.0134E-09 |
| Body mass index (BMI) \|\| id:ukb-a-248 | Major Depressive Disorder \|\| id:ieu-a-1188 | 173005 | rs946185 | 0.19877901 | 0.03478003 | 1.095E-08 |
| Body mass index (BMI) \|\| id:ukb-a-248 | Major Depressive Disorder \|\| id:ieu-a-1188 | 173005 | rs9515455 | 0.200406 | 0.03500265 | 1.0316E-08 |
| Body mass index (BMI) \|\| id:ukb-a-248 | Major Depressive Disorder \|\| id:ieu-a-1188 | 173005 | rs9527906 | 0.20216481 | 0.03501713 | 7.7728E-09 |
| Body mass index (BMI) \|\| id:ukb-a-248 | Major Depressive Disorder \|\| id:ieu-a-1188 | 173005 | rs9641499 | 0.19974941 | 0.03495658 | 1.1021E-08 |
| Body mass index (BMI) \|\| id:ukb-a-248 | Major Depressive Disorder \|\| id:ieu-a-1188 | 173005 | rs9688977 | 0.20001557 | 0.03497214 | 1.0697E-08 |
| Body mass index (BMI) \|\| id:ukb-a-248 | Major Depressive Disorder \|\| id:ieu-a-1188 | 173005 | rs9843653 | 0.19519197 | 0.03495876 | 2.3573E-08 |
| Body mass index (BMI) \|\| id:ukb-a-248 | Major Depressive Disorder \|\| id:ieu-a-1188 | 173005 | rs9847186 | 0.19957483 | 0.03487867 | 1.0529E-08 |
| Body mass index (BMI) \|\| id:ukb-a-248 | Major Depressive Disorder \|\| id:ieu-a-1188 | 173005 | All | 0.20202219 | 0.03491275 | 7.1873E-09 |
| Qualifications: College or University degree \|\| id:ukb-a-397 | Major Depressive Disorder \|\| id:ieu-a-1188 | 173005 | rs1007274 | -0.6000568 | 0.1166884 | 2.7127E-07 |
| Qualifications: College or University degree \|\| id:ukb-a-397 | Major Depressive Disorder \|\| id:ieu-a-1188 | 173005 | rs10088132 | -0.6207656 | 0.11749307 | 1.2679E-07 |
| Qualifications: College or University degree \|\| id:ukb-a-397 | Major Depressive Disorder \|\| id:ieu-a-1188 | 173005 | rs10129035 | -0.6053194 | 0.11750854 | 2.5871E-07 |
| Qualifications: College or University degree \|\| id:ukb-a-397 | Major Depressive Disorder \|\| id:ieu-a-1188 | 173005 | rs10138733 | -0.6120431 | 0.11770191 | 1.9935E-07 |
| Qualifications: College or University degree \|\| id:ukb-a-397 | Major Depressive Disorder \|\| id:ieu-a-1188 | 173005 | rs10189857 | -0.6259861 | 0.11742743 | 9.7763E-08 |
| Qualifications: College or University degree \|\| id:ukb-a-397 | Major Depressive Disorder \|\| id:ieu-a-1188 | 173005 | rs10431632 | -0.6094461 | 0.11753845 | 2.1592E-07 |
| Qualifications: College or University degree \|\| id:ukb-a-397 | Major Depressive Disorder \|\| id:ieu-a-1188 | 173005 | rs10515044 | -0.6144533 | 0.11752016 | 1.709E-07 |
| Qualifications: College or University degree \|\| id:ukb-a-397 | Major Depressive Disorder \|\| id:ieu-a-1188 | 173005 | rs10518019 | -0.5925774 | 0.11624179 | 3.4362E-07 |
| Qualifications: College or University degree \|\| id:ukb-a-397 | Major Depressive Disorder \|\| id:ieu-a-1188 | 173005 | rs1073242 | -0.6104484 | 0.11817636 | 2.3971E-07 |
| Qualifications: College or University degree \|\| id:ukb-a-397 | Major Depressive Disorder \|\| id:ieu-a-1188 | 173005 | rs10752262 | -0.6022265 | 0.11704213 | 2.6698E-07 |
| Qualifications: College or University degree \|\| id:ukb-a-397 | Major Depressive Disorder \|\| id:ieu-a-1188 | 173005 | rs10795831 | -0.6141663 | 0.11755631 | 1.7468E-07 |
| Qualifications: College or University degree \|\| id:ukb-a-397 | Major Depressive Disorder \|\| id:ieu-a-1188 | 173005 | rs10801826 | -0.6051087 | 0.1179361 | 2.8849E-07 |
| Qualifications: College or University degree \|\| id:ukb-a-397 | Major Depressive Disorder \|\| id:ieu-a-1188 | 173005 | rs10942580 | -0.6132421 | 0.11755593 | 1.8224E-07 |
| Qualifications: College or University degree \|\| id:ukb-a-397 | Major Depressive Disorder \|\| id:ieu-a-1188 | 173005 | rs10950862 | -0.6049523 | 0.11743484 | 2.5857E-07 |
| Qualifications: College or University degree \|\| id:ukb-a-397 | Major Depressive Disorder \|\| id:ieu-a-1188 | 173005 | rs11080256 | -0.616962 | 0.11743951 | 1.4928E-07 |
| Qualifications: College or University degree \|\| id:ukb-a-397 | Major Depressive Disorder \|\| id:ieu-a-1188 | 173005 | rs11155821 | -0.5913 | 0.11600779 | 3.4495E-07 |
| Qualifications: College or University degree \|\| id:ukb-a-397 | Major Depressive Disorder \|\| id:ieu-a-1188 | 173005 | rs1140892 | -0.6177049 | 0.11687653 | 1.2563E-07 |
| Qualifications: College or University degree \|\| id:ukb-a-397 | Major Depressive Disorder \|\| id:ieu-a-1188 | 173005 | rs114460989 | -0.6179527 | 0.11735083 | 1.3954E-07 |
| Qualifications: College or University degree \|\| id:ukb-a-397 | Major Depressive Disorder \|\| id:ieu-a-1188 | 173005 | rs11678980 | -0.6157165 | 0.11782896 | 1.7368E-07 |
| Qualifications: College or University degree \|\| id:ukb-a-397 | Major Depressive Disorder \|\| id:ieu-a-1188 | 173005 | rs11703948 | -0.6081487 | 0.11765933 | 2.357E-07 |
| Qualifications: College or University degree \|\| id:ukb-a-397 | Major Depressive Disorder \|\| id:ieu-a-1188 | 173005 | rs11711894 | -0.6183912 | 0.11739034 | 1.3805E-07 |
| Qualifications: College or University degree \|\| id:ukb-a-397 | Major Depressive Disorder \|\| id:ieu-a-1188 | 173005 | rs11771370 | -0.6071462 | 0.11752865 | 2.3923E-07 |
| Qualifications: College or University degree \|\| id:ukb-a-397 | Major Depressive Disorder \|\| id:ieu-a-1188 | 173005 | rs11793831 | -0.6249278 | 0.11859561 | 1.3687E-07 |
| Qualifications: College or University degree \|\| id:ukb-a-397 | Major Depressive Disorder \|\| id:ieu-a-1188 | 173005 | rs12028010 | -0.6229453 | 0.11724017 | 1.0759E-07 |
| Qualifications: College or University degree \|\| id:ukb-a-397 | Major Depressive Disorder \|\| id:ieu-a-1188 | 173005 | rs12203182 | -0.6113698 | 0.11762084 | 2.0166E-07 |
| Qualifications: College or University degree \|\| id:ukb-a-397 | Major Depressive Disorder \|\| id:ieu-a-1188 | 173005 | rs12221820 | -0.605566 | 0.11731067 | 2.4423E-07 |
| Qualifications: College or University degree \|\| id:ukb-a-397 | Major Depressive Disorder \|\| id:ieu-a-1188 | 173005 | rs12359372 | -0.6027954 | 0.11714982 | 2.668E-07 |
| Qualifications: College or University degree \|\| id:ukb-a-397 | Major Depressive Disorder \|\| id:ieu-a-1188 | 173005 | rs12375949 | -0.6155183 | 0.11772735 | 1.7104E-07 |
| Qualifications: College or University degree \|\| id:ukb-a-397 | Major Depressive Disorder \|\| id:ieu-a-1188 | 173005 | rs12523278 | -0.6128428 | 0.11802124 | 2.0732E-07 |
| Qualifications: College or University degree \|\| id:ukb-a-397 | Major Depressive Disorder \|\| id:ieu-a-1188 | 173005 | rs12526814 | -0.6159455 | 0.11751641 | 1.594E-07 |
| Qualifications: College or University degree \|\| id:ukb-a-397 | Major Depressive Disorder \|\| id:ieu-a-1188 | 173005 | rs12619354 | -0.607575 | 0.11758504 | 2.3774E-07 |
| Qualifications: College or University degree \|\| id:ukb-a-397 | Major Depressive Disorder \|\| id:ieu-a-1188 | 173005 | rs12735232 | -0.6054748 | 0.1175841 | 2.6147E-07 |
| Qualifications: College or University degree \|\| id:ukb-a-397 | Major Depressive Disorder \|\| id:ieu-a-1188 | 173005 | rs1275202 | -0.6133033 | 0.11762171 | 1.8461E-07 |
| Qualifications: College or University degree \|\| id:ukb-a-397 | Major Depressive Disorder \|\| id:ieu-a-1188 | 173005 | rs12991254 | -0.5991687 | 0.11775396 | 3.6127E-07 |
| Qualifications: College or University degree \|\| id:ukb-a-397 | Major Depressive Disorder \|\| id:ieu-a-1188 | 173005 | rs13033324 | -0.6140454 | 0.11763387 | 1.7895E-07 |
| Qualifications: College or University degree \|\| id:ukb-a-397 | Major Depressive Disorder \|\| id:ieu-a-1188 | 173005 | rs1325604 | -0.5998224 | 0.11688361 | 2.87E-07 |
| Qualifications: College or University degree \|\| id:ukb-a-397 | Major Depressive Disorder \|\| id:ieu-a-1188 | 173005 | rs1333380 | -0.6150151 | 0.1175251 | 1.6673E-07 |
| Qualifications: College or University degree \|\| id:ukb-a-397 | Major Depressive Disorder \|\| id:ieu-a-1188 | 173005 | rs13397208 | -0.6110837 | 0.11756918 | 2.0182E-07 |
| Qualifications: College or University degree \|\| id:ukb-a-397 | Major Depressive Disorder \|\| id:ieu-a-1188 | 173005 | rs13426183 | -0.608535 | 0.11758249 | 2.2744E-07 |
| Qualifications: College or University degree \|\| id:ukb-a-397 | Major Depressive Disorder \|\| id:ieu-a-1188 | 173005 | rs1391441 | -0.6319337 | 0.11678311 | 6.2613E-08 |
| Qualifications: College or University degree \|\| id:ukb-a-397 | Major Depressive Disorder \|\| id:ieu-a-1188 | 173005 | rs1455349 | -0.6128246 | 0.11758448 | 1.8704E-07 |
| Qualifications: College or University degree \|\| id:ukb-a-397 | Major Depressive Disorder \|\| id:ieu-a-1188 | 173005 | rs1536057 | -0.632325 | 0.11522232 | 4.0681E-08 |
| Qualifications: College or University degree \|\| id:ukb-a-397 | Major Depressive Disorder \|\| id:ieu-a-1188 | 173005 | rs1612548 | -0.6044716 | 0.11739337 | 2.6172E-07 |
| Qualifications: College or University degree \|\| id:ukb-a-397 | Major Depressive Disorder \|\| id:ieu-a-1188 | 173005 | rs16828793 | -0.6187795 | 0.11732231 | 1.3335E-07 |
| Qualifications: College or University degree \|\| id:ukb-a-397 | Major Depressive Disorder \|\| id:ieu-a-1188 | 173005 | rs17235639 | -0.6139916 | 0.11744415 | 1.714E-07 |
| Qualifications: College or University degree \|\| id:ukb-a-397 | Major Depressive Disorder \|\| id:ieu-a-1188 | 173005 | rs1727302 | -0.6028825 | 0.11742421 | 2.8331E-07 |
| Qualifications: College or University degree \|\| id:ukb-a-397 | Major Depressive Disorder \|\| id:ieu-a-1188 | 173005 | rs17328524 | -0.5928414 | 0.11554547 | 2.885E-07 |
| Qualifications: College or University degree \|\| id:ukb-a-397 | Major Depressive Disorder \|\| id:ieu-a-1188 | 173005 | rs17563464 | -0.6288448 | 0.11634679 | 6.4838E-08 |
| Qualifications: College or University degree \|\| id:ukb-a-397 | Major Depressive Disorder \|\| id:ieu-a-1188 | 173005 | rs1812587 | -0.6078929 | 0.11757663 | 2.3386E-07 |
| Qualifications: College or University degree \|\| id:ukb-a-397 | Major Depressive Disorder \|\| id:ieu-a-1188 | 173005 | rs1846228 | -0.6217103 | 0.11714907 | 1.1144E-07 |
| Qualifications: College or University degree \|\| id:ukb-a-397 | Major Depressive Disorder \|\| id:ieu-a-1188 | 173005 | rs1914391 | -0.6084905 | 0.11765777 | 2.3198E-07 |
| Qualifications: College or University degree \|\| id:ukb-a-397 | Major Depressive Disorder \|\| id:ieu-a-1188 | 173005 | rs1919489 | -0.6137643 | 0.11750204 | 1.7563E-07 |
| Qualifications: College or University degree \|\| id:ukb-a-397 | Major Depressive Disorder \|\| id:ieu-a-1188 | 173005 | rs1998086 | -0.6090456 | 0.11762887 | 2.2465E-07 |
| Qualifications: College or University degree \|\| id:ukb-a-397 | Major Depressive Disorder \|\| id:ieu-a-1188 | 173005 | rs2061245 | -0.6208493 | 0.11713813 | 1.1571E-07 |
| Qualifications: College or University degree \|\| id:ukb-a-397 | Major Depressive Disorder \|\| id:ieu-a-1188 | 173005 | rs2083440 | -0.6128402 | 0.11762233 | 1.886E-07 |
| Qualifications: College or University degree \|\| id:ukb-a-397 | Major Depressive Disorder \|\| id:ieu-a-1188 | 173005 | rs2109661 | -0.6223089 | 0.11692481 | 1.0246E-07 |
| Qualifications: College or University degree \|\| id:ukb-a-397 | Major Depressive Disorder \|\| id:ieu-a-1188 | 173005 | rs2268991 | -0.6074695 | 0.1174407 | 2.309E-07 |
| Qualifications: College or University degree \|\| id:ukb-a-397 | Major Depressive Disorder \|\| id:ieu-a-1188 | 173005 | rs2271386 | -0.615251 | 0.11753311 | 1.6525E-07 |
| Qualifications: College or University degree \|\| id:ukb-a-397 | Major Depressive Disorder \|\| id:ieu-a-1188 | 173005 | rs2341336 | -0.5994131 | 0.11662386 | 2.7516E-07 |
| Qualifications: College or University degree \|\| id:ukb-a-397 | Major Depressive Disorder \|\| id:ieu-a-1188 | 173005 | rs2447535 | -0.6119882 | 0.11763146 | 1.9653E-07 |
| Qualifications: College or University degree \|\| id:ukb-a-397 | Major Depressive Disorder \|\| id:ieu-a-1188 | 173005 | rs2545795 | -0.6061636 | 0.1173651 | 2.4074E-07 |
| Qualifications: College or University degree \|\| id:ukb-a-397 | Major Depressive Disorder \|\| id:ieu-a-1188 | 173005 | rs2661876 | -0.6050047 | 0.11730744 | 2.5036E-07 |
| Qualifications: College or University degree \|\| id:ukb-a-397 | Major Depressive Disorder \|\| id:ieu-a-1188 | 173005 | rs2670012 | -0.6183551 | 0.11741491 | 1.3912E-07 |
| Qualifications: College or University degree \|\| id:ukb-a-397 | Major Depressive Disorder \|\| id:ieu-a-1188 | 173005 | rs2725377 | -0.6112328 | 0.11763446 | 2.0358E-07 |
| Qualifications: College or University degree \|\| id:ukb-a-397 | Major Depressive Disorder \|\| id:ieu-a-1188 | 173005 | rs2735421 | -0.6248696 | 0.11728194 | 9.934E-08 |
| Qualifications: College or University degree \|\| id:ukb-a-397 | Major Depressive Disorder \|\| id:ieu-a-1188 | 173005 | rs2762546 | -0.6206996 | 0.11744574 | 1.2571E-07 |
| Qualifications: College or University degree \|\| id:ukb-a-397 | Major Depressive Disorder \|\| id:ieu-a-1188 | 173005 | rs2858088 | -0.6004361 | 0.11682346 | 2.752E-07 |
| Qualifications: College or University degree \|\| id:ukb-a-397 | Major Depressive Disorder \|\| id:ieu-a-1188 | 173005 | rs2860049 | -0.5904455 | 0.11602112 | 3.5973E-07 |
| Qualifications: College or University degree \|\| id:ukb-a-397 | Major Depressive Disorder \|\| id:ieu-a-1188 | 173005 | rs28624826 | -0.6179206 | 0.11717224 | 1.3377E-07 |
| Qualifications: College or University degree \|\| id:ukb-a-397 | Major Depressive Disorder \|\| id:ieu-a-1188 | 173005 | rs28807201 | -0.6193037 | 0.11727351 | 1.2859E-07 |
| Qualifications: College or University degree \|\| id:ukb-a-397 | Major Depressive Disorder \|\| id:ieu-a-1188 | 173005 | rs2885198 | -0.6029379 | 0.11719022 | 2.6759E-07 |
| Qualifications: College or University degree \|\| id:ukb-a-397 | Major Depressive Disorder \|\| id:ieu-a-1188 | 173005 | rs2995803 | -0.6143615 | 0.11779791 | 1.8344E-07 |
| Qualifications: College or University degree \|\| id:ukb-a-397 | Major Depressive Disorder \|\| id:ieu-a-1188 | 173005 | rs303752 | -0.6293191 | 0.11630829 | 6.2749E-08 |
| Qualifications: College or University degree \|\| id:ukb-a-397 | Major Depressive Disorder \|\| id:ieu-a-1188 | 173005 | rs34811474 | -0.6122586 | 0.11759935 | 1.9263E-07 |
| Qualifications: College or University degree \|\| id:ukb-a-397 | Major Depressive Disorder \|\| id:ieu-a-1188 | 173005 | rs35617898 | -0.6255293 | 0.11684448 | 8.626E-08 |
| Qualifications: College or University degree \|\| id:ukb-a-397 | Major Depressive Disorder \|\| id:ieu-a-1188 | 173005 | rs35649565 | -0.6160107 | 0.11751366 | 1.5882E-07 |
| Qualifications: College or University degree \|\| id:ukb-a-397 | Major Depressive Disorder \|\| id:ieu-a-1188 | 173005 | rs35811586 | -0.6023578 | 0.11715154 | 2.7227E-07 |
| Qualifications: College or University degree \|\| id:ukb-a-397 | Major Depressive Disorder \|\| id:ieu-a-1188 | 173005 | rs360947 | -0.6149253 | 0.11762729 | 1.7159E-07 |
| Qualifications: College or University degree \|\| id:ukb-a-397 | Major Depressive Disorder \|\| id:ieu-a-1188 | 173005 | rs38857 | -0.6002795 | 0.11677013 | 2.7372E-07 |
| Qualifications: College or University degree \|\| id:ukb-a-397 | Major Depressive Disorder \|\| id:ieu-a-1188 | 173005 | rs3943093 | -0.6108573 | 0.11774971 | 2.1284E-07 |
| Qualifications: College or University degree \|\| id:ukb-a-397 | Major Depressive Disorder \|\| id:ieu-a-1188 | 173005 | rs4567409 | -0.6102404 | 0.11756438 | 2.0951E-07 |
| Qualifications: College or University degree \|\| id:ukb-a-397 | Major Depressive Disorder \|\| id:ieu-a-1188 | 173005 | rs4691576 | -0.6054818 | 0.11757713 | 2.6096E-07 |
| Qualifications: College or University degree \|\| id:ukb-a-397 | Major Depressive Disorder \|\| id:ieu-a-1188 | 173005 | rs4726070 | -0.61069 | 0.11760299 | 2.0714E-07 |
| Qualifications: College or University degree \|\| id:ukb-a-397 | Major Depressive Disorder \|\| id:ieu-a-1188 | 173005 | rs4731366 | -0.6187234 | 0.11732012 | 1.3362E-07 |
| Qualifications: College or University degree \|\| id:ukb-a-397 | Major Depressive Disorder \|\| id:ieu-a-1188 | 173005 | rs4810227 | -0.6212052 | 0.11750226 | 1.2451E-07 |
| Qualifications: College or University degree \|\| id:ukb-a-397 | Major Depressive Disorder \|\| id:ieu-a-1188 | 173005 | rs4877151 | -0.5982551 | 0.11643882 | 2.7777E-07 |
| Qualifications: College or University degree \|\| id:ukb-a-397 | Major Depressive Disorder \|\| id:ieu-a-1188 | 173005 | rs4960987 | -0.6034328 | 0.11741445 | 2.757E-07 |
| Qualifications: College or University degree \|\| id:ukb-a-397 | Major Depressive Disorder \|\| id:ieu-a-1188 | 173005 | rs4983187 | -0.6347971 | 0.11614675 | 4.6167E-08 |
| Qualifications: College or University degree \|\| id:ukb-a-397 | Major Depressive Disorder \|\| id:ieu-a-1188 | 173005 | rs549845 | -0.6048655 | 0.11802399 | 2.9764E-07 |
| Qualifications: College or University degree \|\| id:ukb-a-397 | Major Depressive Disorder \|\| id:ieu-a-1188 | 173005 | rs55677194 | -0.6201508 | 0.11719364 | 1.212E-07 |
| Qualifications: College or University degree \|\| id:ukb-a-397 | Major Depressive Disorder \|\| id:ieu-a-1188 | 173005 | rs56099375 | -0.6108791 | 0.11754411 | 2.0251E-07 |
| Qualifications: College or University degree \|\| id:ukb-a-397 | Major Depressive Disorder \|\| id:ieu-a-1188 | 173005 | rs56151069 | -0.6200303 | 0.1169871 | 1.1581E-07 |
| Qualifications: College or University degree \|\| id:ukb-a-397 | Major Depressive Disorder \|\| id:ieu-a-1188 | 173005 | rs567203 | -0.6104593 | 0.11772443 | 2.1544E-07 |
| Qualifications: College or University degree \|\| id:ukb-a-397 | Major Depressive Disorder \|\| id:ieu-a-1188 | 173005 | rs5758922 | -0.6115291 | 0.11755035 | 1.9687E-07 |
| Qualifications: College or University degree \|\| id:ukb-a-397 | Major Depressive Disorder \|\| id:ieu-a-1188 | 173005 | rs580241 | -0.6130632 | 0.11771392 | 1.9081E-07 |
| Qualifications: College or University degree \|\| id:ukb-a-397 | Major Depressive Disorder \|\| id:ieu-a-1188 | 173005 | rs59123361 | -0.6089201 | 0.11758463 | 2.2359E-07 |
| Qualifications: College or University degree \|\| id:ukb-a-397 | Major Depressive Disorder \|\| id:ieu-a-1188 | 173005 | rs613872 | -0.5931988 | 0.11591013 | 3.0921E-07 |
| Qualifications: College or University degree \|\| id:ukb-a-397 | Major Depressive Disorder \|\| id:ieu-a-1188 | 173005 | rs61527214 | -0.6038958 | 0.11720876 | 2.573E-07 |
| Qualifications: College or University degree \|\| id:ukb-a-397 | Major Depressive Disorder \|\| id:ieu-a-1188 | 173005 | rs62036613 | -0.6171918 | 0.11766609 | 1.5604E-07 |
| Qualifications: College or University degree \|\| id:ukb-a-397 | Major Depressive Disorder \|\| id:ieu-a-1188 | 173005 | rs62063281 | -0.6013172 | 0.11730737 | 2.9597E-07 |
| Qualifications: College or University degree \|\| id:ukb-a-397 | Major Depressive Disorder \|\| id:ieu-a-1188 | 173005 | rs62172156 | -0.6082892 | 0.11778032 | 2.4094E-07 |
| Qualifications: College or University degree \|\| id:ukb-a-397 | Major Depressive Disorder \|\| id:ieu-a-1188 | 173005 | rs62182993 | -0.5946068 | 0.11676995 | 3.5408E-07 |
| Qualifications: College or University degree \|\| id:ukb-a-397 | Major Depressive Disorder \|\| id:ieu-a-1188 | 173005 | rs62246015 | -0.5931466 | 0.11681649 | 3.8225E-07 |
| Qualifications: College or University degree \|\| id:ukb-a-397 | Major Depressive Disorder \|\| id:ieu-a-1188 | 173005 | rs62507575 | -0.6325269 | 0.11498076 | 3.7731E-08 |
| Qualifications: College or University degree \|\| id:ukb-a-397 | Major Depressive Disorder \|\| id:ieu-a-1188 | 173005 | rs634546 | -0.6001867 | 0.11691626 | 2.8444E-07 |
| Qualifications: College or University degree \|\| id:ukb-a-397 | Major Depressive Disorder \|\| id:ieu-a-1188 | 173005 | rs6429911 | -0.6060216 | 0.11742839 | 2.4593E-07 |
| Qualifications: College or University degree \|\| id:ukb-a-397 | Major Depressive Disorder \|\| id:ieu-a-1188 | 173005 | rs6556982 | -0.6163978 | 0.11745813 | 1.5392E-07 |
| Qualifications: College or University degree \|\| id:ukb-a-397 | Major Depressive Disorder \|\| id:ieu-a-1188 | 173005 | rs6559365 | -0.6182756 | 0.11729603 | 1.3563E-07 |
| Qualifications: College or University degree \|\| id:ukb-a-397 | Major Depressive Disorder \|\| id:ieu-a-1188 | 173005 | rs6673646 | -0.6028162 | 0.11711061 | 2.6411E-07 |
| Qualifications: College or University degree \|\| id:ukb-a-397 | Major Depressive Disorder \|\| id:ieu-a-1188 | 173005 | rs6676960 | -0.6140597 | 0.11755594 | 1.7552E-07 |
| Qualifications: College or University degree \|\| id:ukb-a-397 | Major Depressive Disorder \|\| id:ieu-a-1188 | 173005 | rs6704703 | -0.6181309 | 0.11736638 | 1.3891E-07 |
| Qualifications: College or University degree \|\| id:ukb-a-397 | Major Depressive Disorder \|\| id:ieu-a-1188 | 173005 | rs6707062 | -0.6245507 | 0.1166691 | 8.6422E-08 |
| Qualifications: College or University degree \|\| id:ukb-a-397 | Major Depressive Disorder \|\| id:ieu-a-1188 | 173005 | rs6735842 | -0.6115349 | 0.11757165 | 1.9781E-07 |
| Qualifications: College or University degree \|\| id:ukb-a-397 | Major Depressive Disorder \|\| id:ieu-a-1188 | 173005 | rs680767 | -0.6243727 | 0.11665532 | 8.685E-08 |
| Qualifications: College or University degree \|\| id:ukb-a-397 | Major Depressive Disorder \|\| id:ieu-a-1188 | 173005 | rs6839304 | -0.6109599 | 0.11765743 | 2.0726E-07 |
| Qualifications: College or University degree \|\| id:ukb-a-397 | Major Depressive Disorder \|\| id:ieu-a-1188 | 173005 | rs723968 | -0.6169238 | 0.11753822 | 1.5317E-07 |
| Qualifications: College or University degree \|\| id:ukb-a-397 | Major Depressive Disorder \|\| id:ieu-a-1188 | 173005 | rs7260359 | -0.6191104 | 0.11765478 | 1.4242E-07 |
| Qualifications: College or University degree \|\| id:ukb-a-397 | Major Depressive Disorder \|\| id:ieu-a-1188 | 173005 | rs72801817 | -0.620448 | 0.11742594 | 1.2657E-07 |
| Qualifications: College or University degree \|\| id:ukb-a-397 | Major Depressive Disorder \|\| id:ieu-a-1188 | 173005 | rs72829857 | -0.6044312 | 0.11758246 | 2.7403E-07 |
| Qualifications: College or University degree \|\| id:ukb-a-397 | Major Depressive Disorder \|\| id:ieu-a-1188 | 173005 | rs7526112 | -0.5967042 | 0.11744575 | 3.7609E-07 |
| Qualifications: College or University degree \|\| id:ukb-a-397 | Major Depressive Disorder \|\| id:ieu-a-1188 | 173005 | rs7561798 | -0.605421 | 0.11747187 | 2.5532E-07 |
| Qualifications: College or University degree \|\| id:ukb-a-397 | Major Depressive Disorder \|\| id:ieu-a-1188 | 173005 | rs7577926 | -0.6154826 | 0.11754767 | 1.6407E-07 |
| Qualifications: College or University degree \|\| id:ukb-a-397 | Major Depressive Disorder \|\| id:ieu-a-1188 | 173005 | rs7582977 | -0.6197888 | 0.11716183 | 1.223E-07 |
| Qualifications: College or University degree \|\| id:ukb-a-397 | Major Depressive Disorder \|\| id:ieu-a-1188 | 173005 | rs76076331 | -0.6084971 | 0.11754295 | 2.2572E-07 |
| Qualifications: College or University degree \|\| id:ukb-a-397 | Major Depressive Disorder \|\| id:ieu-a-1188 | 173005 | rs7613360 | -0.5863166 | 0.11781448 | 6.4708E-07 |
| Qualifications: College or University degree \|\| id:ukb-a-397 | Major Depressive Disorder \|\| id:ieu-a-1188 | 173005 | rs77451029 | -0.6199554 | 0.11726726 | 1.2455E-07 |
| Qualifications: College or University degree \|\| id:ukb-a-397 | Major Depressive Disorder \|\| id:ieu-a-1188 | 173005 | rs7757476 | -0.616285 | 0.11756665 | 1.5884E-07 |
| Qualifications: College or University degree \|\| id:ukb-a-397 | Major Depressive Disorder \|\| id:ieu-a-1188 | 173005 | rs783562 | -0.6185621 | 0.11740618 | 1.3749E-07 |
| Qualifications: College or University degree \|\| id:ukb-a-397 | Major Depressive Disorder \|\| id:ieu-a-1188 | 173005 | rs7862560 | -0.6181647 | 0.11730836 | 1.3674E-07 |
| Qualifications: College or University degree \|\| id:ukb-a-397 | Major Depressive Disorder \|\| id:ieu-a-1188 | 173005 | rs7902 | -0.6101049 | 0.11774211 | 2.1987E-07 |
| Qualifications: College or University degree \|\| id:ukb-a-397 | Major Depressive Disorder \|\| id:ieu-a-1188 | 173005 | rs7921305 | -0.6095658 | 0.11778062 | 2.2738E-07 |
| Qualifications: College or University degree \|\| id:ukb-a-397 | Major Depressive Disorder \|\| id:ieu-a-1188 | 173005 | rs7965989 | -0.6178206 | 0.11751707 | 1.462E-07 |
| Qualifications: College or University degree \|\| id:ukb-a-397 | Major Depressive Disorder \|\| id:ieu-a-1188 | 173005 | rs7966054 | -0.6251447 | 0.11656665 | 8.186E-08 |
| Qualifications: College or University degree \|\| id:ukb-a-397 | Major Depressive Disorder \|\| id:ieu-a-1188 | 173005 | rs7988627 | -0.6169558 | 0.11771375 | 1.5958E-07 |
| Qualifications: College or University degree \|\| id:ukb-a-397 | Major Depressive Disorder \|\| id:ieu-a-1188 | 173005 | rs79918827 | -0.6172926 | 0.11722077 | 1.3938E-07 |
| Qualifications: College or University degree \|\| id:ukb-a-397 | Major Depressive Disorder \|\| id:ieu-a-1188 | 173005 | rs8051058 | -0.6141753 | 0.1176035 | 1.766E-07 |
| Qualifications: College or University degree \|\| id:ukb-a-397 | Major Depressive Disorder \|\| id:ieu-a-1188 | 173005 | rs8112975 | -0.6122128 | 0.11762579 | 1.9426E-07 |
| Qualifications: College or University degree \|\| id:ukb-a-397 | Major Depressive Disorder \|\| id:ieu-a-1188 | 173005 | rs818414 | -0.6169531 | 0.11746258 | 1.5018E-07 |
| Qualifications: College or University degree \|\| id:ukb-a-397 | Major Depressive Disorder \|\| id:ieu-a-1188 | 173005 | rs860815 | -0.6288826 | 0.11653997 | 6.8036E-08 |
| Qualifications: College or University degree \|\| id:ukb-a-397 | Major Depressive Disorder \|\| id:ieu-a-1188 | 173005 | rs9357004 | -0.6337305 | 0.11677557 | 5.7338E-08 |
| Qualifications: College or University degree \|\| id:ukb-a-397 | Major Depressive Disorder \|\| id:ieu-a-1188 | 173005 | rs9401593 | -0.6407786 | 0.11783122 | 5.385E-08 |
| Qualifications: College or University degree \|\| id:ukb-a-397 | Major Depressive Disorder \|\| id:ieu-a-1188 | 173005 | rs9556958 | -0.6137487 | 0.1176335 | 1.8139E-07 |
| Qualifications: College or University degree \|\| id:ukb-a-397 | Major Depressive Disorder \|\| id:ieu-a-1188 | 173005 | rs9557427 | -0.6063852 | 0.11746767 | 2.4414E-07 |
| Qualifications: College or University degree \|\| id:ukb-a-397 | Major Depressive Disorder \|\| id:ieu-a-1188 | 173005 | rs9608438 | -0.6120084 | 0.11760064 | 1.9491E-07 |
| Qualifications: College or University degree \|\| id:ukb-a-397 | Major Depressive Disorder \|\| id:ieu-a-1188 | 173005 | rs962490 | -0.5922994 | 0.11552978 | 2.9469E-07 |
| Qualifications: College or University degree \|\| id:ukb-a-397 | Major Depressive Disorder \|\| id:ieu-a-1188 | 173005 | rs9826269 | -0.6196387 | 0.11726319 | 1.2628E-07 |
| Qualifications: College or University degree \|\| id:ukb-a-397 | Major Depressive Disorder \|\| id:ieu-a-1188 | 173005 | rs9891803 | -0.6021971 | 0.11716512 | 2.7513E-07 |
| Qualifications: College or University degree \|\| id:ukb-a-397 | Major Depressive Disorder \|\| id:ieu-a-1188 | 173005 | rs9934839 | -0.6119578 | 0.11754403 | 1.9275E-07 |
| Qualifications: College or University degree \|\| id:ukb-a-397 | Major Depressive Disorder \|\| id:ieu-a-1188 | 173005 | rs9960416 | -0.6038416 | 0.11725074 | 2.6048E-07 |
| Qualifications: College or University degree \|\| id:ukb-a-397 | Major Depressive Disorder \|\| id:ieu-a-1188 | 173005 | rs9964724 | -0.5856768 | 0.11704671 | 5.6215E-07 |
| Qualifications: College or University degree \|\| id:ukb-a-397 | Major Depressive Disorder \|\| id:ieu-a-1188 | 173005 | rs997779 | -0.6089505 | 0.11760273 | 2.2424E-07 |
| Qualifications: College or University degree \|\| id:ukb-a-397 | Major Depressive Disorder \|\| id:ieu-a-1188 | 173005 | All | -0.6119465 | 0.11690881 | 1.6553E-07 |
| Qualifications: A levels/AS levels or equivalent \|\| id:ukb-a-399 | Major Depressive Disorder \|\| id:ieu-a-1188 | 173005 | rs10180461 | -0.8612794 | 0.23249051 | 0.00021174 |
| Qualifications: A levels/AS levels or equivalent \|\| id:ukb-a-399 | Major Depressive Disorder \|\| id:ieu-a-1188 | 173005 | rs10189857 | -0.9354665 | 0.23055321 | 4.9604E-05 |
| Qualifications: A levels/AS levels or equivalent \|\| id:ukb-a-399 | Major Depressive Disorder \|\| id:ieu-a-1188 | 173005 | rs10402747 | -0.902382 | 0.23143885 | 9.6587E-05 |
| Qualifications: A levels/AS levels or equivalent \|\| id:ukb-a-399 | Major Depressive Disorder \|\| id:ieu-a-1188 | 173005 | rs10761254 | -0.8409571 | 0.228115 | 0.00022732 |
| Qualifications: A levels/AS levels or equivalent \|\| id:ukb-a-399 | Major Depressive Disorder \|\| id:ieu-a-1188 | 173005 | rs11600296 | -0.8804309 | 0.23303592 | 0.00015804 |
| Qualifications: A levels/AS levels or equivalent \|\| id:ukb-a-399 | Major Depressive Disorder \|\| id:ieu-a-1188 | 173005 | rs11793831 | -0.9056774 | 0.2339365 | 0.00010818 |
| Qualifications: A levels/AS levels or equivalent \|\| id:ukb-a-399 | Major Depressive Disorder \|\| id:ieu-a-1188 | 173005 | rs12089622 | -0.8504008 | 0.23258839 | 0.00025593 |
| Qualifications: A levels/AS levels or equivalent \|\| id:ukb-a-399 | Major Depressive Disorder \|\| id:ieu-a-1188 | 173005 | rs12511976 | -0.9146473 | 0.22925465 | 6.6169E-05 |
| Qualifications: A levels/AS levels or equivalent \|\| id:ukb-a-399 | Major Depressive Disorder \|\| id:ieu-a-1188 | 173005 | rs12546203 | -0.8746415 | 0.23290063 | 0.00017305 |
| Qualifications: A levels/AS levels or equivalent \|\| id:ukb-a-399 | Major Depressive Disorder \|\| id:ieu-a-1188 | 173005 | rs12614212 | -0.891429 | 0.23293598 | 0.00012975 |
| Qualifications: A levels/AS levels or equivalent \|\| id:ukb-a-399 | Major Depressive Disorder \|\| id:ieu-a-1188 | 173005 | rs1291871 | -0.9231255 | 0.22813231 | 5.2001E-05 |
| Qualifications: A levels/AS levels or equivalent \|\| id:ukb-a-399 | Major Depressive Disorder \|\| id:ieu-a-1188 | 173005 | rs13034294 | -0.8773153 | 0.23306138 | 0.00016701 |
| Qualifications: A levels/AS levels or equivalent \|\| id:ukb-a-399 | Major Depressive Disorder \|\| id:ieu-a-1188 | 173005 | rs13240401 | -0.8471079 | 0.22963218 | 0.00022516 |
| Qualifications: A levels/AS levels or equivalent \|\| id:ukb-a-399 | Major Depressive Disorder \|\| id:ieu-a-1188 | 173005 | rs1424532 | -0.8987598 | 0.23232365 | 0.00010948 |
| Qualifications: A levels/AS levels or equivalent \|\| id:ukb-a-399 | Major Depressive Disorder \|\| id:ieu-a-1188 | 173005 | rs178165 | -0.9430677 | 0.22265684 | 2.2802E-05 |
| Qualifications: A levels/AS levels or equivalent \|\| id:ukb-a-399 | Major Depressive Disorder \|\| id:ieu-a-1188 | 173005 | rs17881016 | -0.8666828 | 0.23211768 | 0.00018861 |
| Qualifications: A levels/AS levels or equivalent \|\| id:ukb-a-399 | Major Depressive Disorder \|\| id:ieu-a-1188 | 173005 | rs187600727 | -0.874629 | 0.23251683 | 0.00016885 |
| Qualifications: A levels/AS levels or equivalent \|\| id:ukb-a-399 | Major Depressive Disorder \|\| id:ieu-a-1188 | 173005 | rs1881975 | -0.9185521 | 0.22827648 | 5.7253E-05 |
| Qualifications: A levels/AS levels or equivalent \|\| id:ukb-a-399 | Major Depressive Disorder \|\| id:ieu-a-1188 | 173005 | rs1931263 | -0.8143455 | 0.22287836 | 0.00025842 |
| Qualifications: A levels/AS levels or equivalent \|\| id:ukb-a-399 | Major Depressive Disorder \|\| id:ieu-a-1188 | 173005 | rs1966191 | -0.8870231 | 0.23251094 | 0.0001362 |
| Qualifications: A levels/AS levels or equivalent \|\| id:ukb-a-399 | Major Depressive Disorder \|\| id:ieu-a-1188 | 173005 | rs2179152 | -0.8612752 | 0.23406056 | 0.0002335 |
| Qualifications: A levels/AS levels or equivalent \|\| id:ukb-a-399 | Major Depressive Disorder \|\| id:ieu-a-1188 | 173005 | rs2325037 | -0.9168226 | 0.22905254 | 6.2631E-05 |
| Qualifications: A levels/AS levels or equivalent \|\| id:ukb-a-399 | Major Depressive Disorder \|\| id:ieu-a-1188 | 173005 | rs2352974 | -0.8025897 | 0.2310563 | 0.00051359 |
| Qualifications: A levels/AS levels or equivalent \|\| id:ukb-a-399 | Major Depressive Disorder \|\| id:ieu-a-1188 | 173005 | rs2479552 | -0.8968414 | 0.23234433 | 0.0001134 |
| Qualifications: A levels/AS levels or equivalent \|\| id:ukb-a-399 | Major Depressive Disorder \|\| id:ieu-a-1188 | 173005 | rs2568816 | -0.9148372 | 0.22990422 | 6.9145E-05 |
| Qualifications: A levels/AS levels or equivalent \|\| id:ukb-a-399 | Major Depressive Disorder \|\| id:ieu-a-1188 | 173005 | rs28704868 | -0.8402384 | 0.22816229 | 0.00023084 |
| Qualifications: A levels/AS levels or equivalent \|\| id:ukb-a-399 | Major Depressive Disorder \|\| id:ieu-a-1188 | 173005 | rs34334167 | -0.89214 | 0.23217149 | 0.00012174 |
| Qualifications: A levels/AS levels or equivalent \|\| id:ukb-a-399 | Major Depressive Disorder \|\| id:ieu-a-1188 | 173005 | rs34954834 | -0.8547532 | 0.23156938 | 0.00022326 |
| Qualifications: A levels/AS levels or equivalent \|\| id:ukb-a-399 | Major Depressive Disorder \|\| id:ieu-a-1188 | 173005 | rs3943093 | -0.8791014 | 0.23266667 | 0.00015786 |
| Qualifications: A levels/AS levels or equivalent \|\| id:ukb-a-399 | Major Depressive Disorder \|\| id:ieu-a-1188 | 173005 | rs421853 | -0.8956482 | 0.23213708 | 0.00011419 |
| Qualifications: A levels/AS levels or equivalent \|\| id:ukb-a-399 | Major Depressive Disorder \|\| id:ieu-a-1188 | 173005 | rs4457002 | -0.8183982 | 0.22766693 | 0.00032474 |
| Qualifications: A levels/AS levels or equivalent \|\| id:ukb-a-399 | Major Depressive Disorder \|\| id:ieu-a-1188 | 173005 | rs4675248 | -0.8801795 | 0.23279538 | 0.00015625 |
| Qualifications: A levels/AS levels or equivalent \|\| id:ukb-a-399 | Major Depressive Disorder \|\| id:ieu-a-1188 | 173005 | rs4886901 | -0.8680633 | 0.23209937 | 0.00018398 |
| Qualifications: A levels/AS levels or equivalent \|\| id:ukb-a-399 | Major Depressive Disorder \|\| id:ieu-a-1188 | 173005 | rs4981692 | -0.8856145 | 0.2327234 | 0.00014155 |
| Qualifications: A levels/AS levels or equivalent \|\| id:ukb-a-399 | Major Depressive Disorder \|\| id:ieu-a-1188 | 173005 | rs518709 | -0.8863273 | 0.23307475 | 0.00014309 |
| Qualifications: A levels/AS levels or equivalent \|\| id:ukb-a-399 | Major Depressive Disorder \|\| id:ieu-a-1188 | 173005 | rs55853834 | -0.915837 | 0.23042665 | 7.0519E-05 |
| Qualifications: A levels/AS levels or equivalent \|\| id:ukb-a-399 | Major Depressive Disorder \|\| id:ieu-a-1188 | 173005 | rs56219078 | -0.9029967 | 0.23169924 | 9.7279E-05 |
| Qualifications: A levels/AS levels or equivalent \|\| id:ukb-a-399 | Major Depressive Disorder \|\| id:ieu-a-1188 | 173005 | rs578096 | -0.8961367 | 0.23175953 | 0.00011033 |
| Qualifications: A levels/AS levels or equivalent \|\| id:ukb-a-399 | Major Depressive Disorder \|\| id:ieu-a-1188 | 173005 | rs58503743 | -0.8895945 | 0.23234621 | 0.0001288 |
| Qualifications: A levels/AS levels or equivalent \|\| id:ukb-a-399 | Major Depressive Disorder \|\| id:ieu-a-1188 | 173005 | rs604781 | -0.8820689 | 0.23259702 | 0.00014928 |
| Qualifications: A levels/AS levels or equivalent \|\| id:ukb-a-399 | Major Depressive Disorder \|\| id:ieu-a-1188 | 173005 | rs62139974 | -0.905597 | 0.23134729 | 9.0611E-05 |
| Qualifications: A levels/AS levels or equivalent \|\| id:ukb-a-399 | Major Depressive Disorder \|\| id:ieu-a-1188 | 173005 | rs62183028 | -0.852081 | 0.23044197 | 0.00021765 |
| Qualifications: A levels/AS levels or equivalent \|\| id:ukb-a-399 | Major Depressive Disorder \|\| id:ieu-a-1188 | 173005 | rs6714628 | -0.8393407 | 0.23017425 | 0.00026579 |
| Qualifications: A levels/AS levels or equivalent \|\| id:ukb-a-399 | Major Depressive Disorder \|\| id:ieu-a-1188 | 173005 | rs67589009 | -0.957239 | 0.22018571 | 1.3775E-05 |
| Qualifications: A levels/AS levels or equivalent \|\| id:ukb-a-399 | Major Depressive Disorder \|\| id:ieu-a-1188 | 173005 | rs7203315 | -0.9423649 | 0.2258877 | 3.0216E-05 |
| Qualifications: A levels/AS levels or equivalent \|\| id:ukb-a-399 | Major Depressive Disorder \|\| id:ieu-a-1188 | 173005 | rs7242967 | -0.8464086 | 0.23065866 | 0.000243 |
| Qualifications: A levels/AS levels or equivalent \|\| id:ukb-a-399 | Major Depressive Disorder \|\| id:ieu-a-1188 | 173005 | rs72829857 | -0.8608456 | 0.23206804 | 0.00020771 |
| Qualifications: A levels/AS levels or equivalent \|\| id:ukb-a-399 | Major Depressive Disorder \|\| id:ieu-a-1188 | 173005 | rs7325960 | -0.833879 | 0.22968081 | 0.00028276 |
| Qualifications: A levels/AS levels or equivalent \|\| id:ukb-a-399 | Major Depressive Disorder \|\| id:ieu-a-1188 | 173005 | rs74619517 | -0.8555944 | 0.23060908 | 0.00020714 |
| Qualifications: A levels/AS levels or equivalent \|\| id:ukb-a-399 | Major Depressive Disorder \|\| id:ieu-a-1188 | 173005 | rs7896518 | -0.8580056 | 0.23169706 | 0.00021295 |
| Qualifications: A levels/AS levels or equivalent \|\| id:ukb-a-399 | Major Depressive Disorder \|\| id:ieu-a-1188 | 173005 | All | -0.8808318 | 0.22838949 | 0.00011492 |
| Types of physical activity in last 4 weeks: Heavy DIY (eg: weeding, lawn mowing, carpentry, digging) \|\| id:ukb-b-13184 | Major Depressive Disorder \|\| id:ieu-a-1188 | 173005 | rs10200379 | -0.7991938 | 0.3693655 | 0.03048786 |
| Types of physical activity in last 4 weeks: Heavy DIY (eg: weeding, lawn mowing, carpentry, digging) \|\| id:ukb-b-13184 | Major Depressive Disorder \|\| id:ieu-a-1188 | 173005 | rs11076320 | -0.8963638 | 0.36937785 | 0.01523746 |
| Types of physical activity in last 4 weeks: Heavy DIY (eg: weeding, lawn mowing, carpentry, digging) \|\| id:ukb-b-13184 | Major Depressive Disorder \|\| id:ieu-a-1188 | 173005 | rs11116540 | -0.9784587 | 0.3700831 | 0.00819595 |
| Types of physical activity in last 4 weeks: Heavy DIY (eg: weeding, lawn mowing, carpentry, digging) \|\| id:ukb-b-13184 | Major Depressive Disorder \|\| id:ieu-a-1188 | 173005 | rs11984231 | -1.0414854 | 0.37087344 | 0.00498199 |
| Types of physical activity in last 4 weeks: Heavy DIY (eg: weeding, lawn mowing, carpentry, digging) \|\| id:ukb-b-13184 | Major Depressive Disorder \|\| id:ieu-a-1188 | 173005 | rs12203592 | -0.916691 | 0.36616858 | 0.01229831 |
| Types of physical activity in last 4 weeks: Heavy DIY (eg: weeding, lawn mowing, carpentry, digging) \|\| id:ukb-b-13184 | Major Depressive Disorder \|\| id:ieu-a-1188 | 173005 | rs1288124 | -0.9153864 | 0.36859223 | 0.01301107 |
| Types of physical activity in last 4 weeks: Heavy DIY (eg: weeding, lawn mowing, carpentry, digging) \|\| id:ukb-b-13184 | Major Depressive Disorder \|\| id:ieu-a-1188 | 173005 | rs17157586 | -0.8318243 | 0.36893533 | 0.02415457 |
| Types of physical activity in last 4 weeks: Heavy DIY (eg: weeding, lawn mowing, carpentry, digging) \|\| id:ukb-b-13184 | Major Depressive Disorder \|\| id:ieu-a-1188 | 173005 | rs19573 | -0.9585755 | 0.37009898 | 0.00959615 |
| Types of physical activity in last 4 weeks: Heavy DIY (eg: weeding, lawn mowing, carpentry, digging) \|\| id:ukb-b-13184 | Major Depressive Disorder \|\| id:ieu-a-1188 | 173005 | rs2529676 | -0.9651611 | 0.3697687 | 0.00904958 |
| Types of physical activity in last 4 weeks: Heavy DIY (eg: weeding, lawn mowing, carpentry, digging) \|\| id:ukb-b-13184 | Major Depressive Disorder \|\| id:ieu-a-1188 | 173005 | rs2661990 | -0.7386646 | 0.36914475 | 0.04539064 |
| Types of physical activity in last 4 weeks: Heavy DIY (eg: weeding, lawn mowing, carpentry, digging) \|\| id:ukb-b-13184 | Major Depressive Disorder \|\| id:ieu-a-1188 | 173005 | rs292026 | -0.9623884 | 0.36874231 | 0.0090563 |
| Types of physical activity in last 4 weeks: Heavy DIY (eg: weeding, lawn mowing, carpentry, digging) \|\| id:ukb-b-13184 | Major Depressive Disorder \|\| id:ieu-a-1188 | 173005 | rs35683183 | -0.8271357 | 0.36908807 | 0.02502443 |
| Types of physical activity in last 4 weeks: Heavy DIY (eg: weeding, lawn mowing, carpentry, digging) \|\| id:ukb-b-13184 | Major Depressive Disorder \|\| id:ieu-a-1188 | 173005 | rs62523388 | -0.9574434 | 0.37084434 | 0.00982885 |
| Types of physical activity in last 4 weeks: Heavy DIY (eg: weeding, lawn mowing, carpentry, digging) \|\| id:ukb-b-13184 | Major Depressive Disorder \|\| id:ieu-a-1188 | 173005 | rs6481128 | -0.9536873 | 0.37017502 | 0.00998598 |
| Types of physical activity in last 4 weeks: Heavy DIY (eg: weeding, lawn mowing, carpentry, digging) \|\| id:ukb-b-13184 | Major Depressive Disorder \|\| id:ieu-a-1188 | 173005 | rs79313673 | -0.9148075 | 0.36949238 | 0.01329196 |
| Types of physical activity in last 4 weeks: Heavy DIY (eg: weeding, lawn mowing, carpentry, digging) \|\| id:ukb-b-13184 | Major Depressive Disorder \|\| id:ieu-a-1188 | 173005 | All | -0.9103238 | 0.35685354 | 0.01074226 |
| Past tobacco smoking \|\| id:ukb-b-2134 | Major Depressive Disorder \|\| id:ieu-a-1188 | 173005 | rs1017998 | -0.3340693 | 0.0727061 | 4.3318E-06 |
| Past tobacco smoking \|\| id:ukb-b-2134 | Major Depressive Disorder \|\| id:ieu-a-1188 | 173005 | rs10474278 | -0.3232655 | 0.07358086 | 1.1162E-05 |
| Past tobacco smoking \|\| id:ukb-b-2134 | Major Depressive Disorder \|\| id:ieu-a-1188 | 173005 | rs10914684 | -0.3193094 | 0.07336336 | 1.3463E-05 |
| Past tobacco smoking \|\| id:ukb-b-2134 | Major Depressive Disorder \|\| id:ieu-a-1188 | 173005 | rs10956808 | -0.3231431 | 0.07373988 | 1.1748E-05 |
| Past tobacco smoking \|\| id:ukb-b-2134 | Major Depressive Disorder \|\| id:ieu-a-1188 | 173005 | rs10959442 | -0.3113136 | 0.07260293 | 1.8038E-05 |
| Past tobacco smoking \|\| id:ukb-b-2134 | Major Depressive Disorder \|\| id:ieu-a-1188 | 173005 | rs1109480 | -0.3250197 | 0.07355589 | 9.9307E-06 |
| Past tobacco smoking \|\| id:ukb-b-2134 | Major Depressive Disorder \|\| id:ieu-a-1188 | 173005 | rs11165623 | -0.3255586 | 0.07346648 | 9.3628E-06 |
| Past tobacco smoking \|\| id:ukb-b-2134 | Major Depressive Disorder \|\| id:ieu-a-1188 | 173005 | rs11613961 | -0.3197417 | 0.07342163 | 1.3315E-05 |
| Past tobacco smoking \|\| id:ukb-b-2134 | Major Depressive Disorder \|\| id:ieu-a-1188 | 173005 | rs1174864 | -0.3099805 | 0.07193803 | 1.64E-05 |
| Past tobacco smoking \|\| id:ukb-b-2134 | Major Depressive Disorder \|\| id:ieu-a-1188 | 173005 | rs12209519 | -0.322151 | 0.07352324 | 1.178E-05 |
| Past tobacco smoking \|\| id:ukb-b-2134 | Major Depressive Disorder \|\| id:ieu-a-1188 | 173005 | rs12333760 | -0.3199236 | 0.07329334 | 1.2714E-05 |
| Past tobacco smoking \|\| id:ukb-b-2134 | Major Depressive Disorder \|\| id:ieu-a-1188 | 173005 | rs12450028 | -0.3240318 | 0.07356353 | 1.0589E-05 |
| Past tobacco smoking \|\| id:ukb-b-2134 | Major Depressive Disorder \|\| id:ieu-a-1188 | 173005 | rs1246265 | -0.3207236 | 0.0737203 | 1.358E-05 |
| Past tobacco smoking \|\| id:ukb-b-2134 | Major Depressive Disorder \|\| id:ieu-a-1188 | 173005 | rs12487411 | -0.3188933 | 0.07344228 | 1.4113E-05 |
| Past tobacco smoking \|\| id:ukb-b-2134 | Major Depressive Disorder \|\| id:ieu-a-1188 | 173005 | rs12608052 | -0.3225083 | 0.07353539 | 1.1558E-05 |
| Past tobacco smoking \|\| id:ukb-b-2134 | Major Depressive Disorder \|\| id:ieu-a-1188 | 173005 | rs12895462 | -0.3314275 | 0.07316039 | 5.8942E-06 |
| Past tobacco smoking \|\| id:ukb-b-2134 | Major Depressive Disorder \|\| id:ieu-a-1188 | 173005 | rs13009008 | -0.3338401 | 0.0727442 | 4.4488E-06 |
| Past tobacco smoking \|\| id:ukb-b-2134 | Major Depressive Disorder \|\| id:ieu-a-1188 | 173005 | rs139896 | -0.3319766 | 0.07308991 | 5.5715E-06 |
| Past tobacco smoking \|\| id:ukb-b-2134 | Major Depressive Disorder \|\| id:ieu-a-1188 | 173005 | rs147052174 | -0.3281645 | 0.07342215 | 7.8382E-06 |
| Past tobacco smoking \|\| id:ukb-b-2134 | Major Depressive Disorder \|\| id:ieu-a-1188 | 173005 | rs1499300 | -0.3294468 | 0.07339742 | 7.1715E-06 |
| Past tobacco smoking \|\| id:ukb-b-2134 | Major Depressive Disorder \|\| id:ieu-a-1188 | 173005 | rs1499976 | -0.3167263 | 0.07380425 | 1.7752E-05 |
| Past tobacco smoking \|\| id:ukb-b-2134 | Major Depressive Disorder \|\| id:ieu-a-1188 | 173005 | rs1559278 | -0.3325215 | 0.07295743 | 5.1705E-06 |
| Past tobacco smoking \|\| id:ukb-b-2134 | Major Depressive Disorder \|\| id:ieu-a-1188 | 173005 | rs1623003 | -0.3165978 | 0.07330583 | 1.5684E-05 |
| Past tobacco smoking \|\| id:ukb-b-2134 | Major Depressive Disorder \|\| id:ieu-a-1188 | 173005 | rs17503369 | -0.3064709 | 0.07120656 | 1.6776E-05 |
| Past tobacco smoking \|\| id:ukb-b-2134 | Major Depressive Disorder \|\| id:ieu-a-1188 | 173005 | rs1899689 | -0.3232179 | 0.07352458 | 1.1024E-05 |
| Past tobacco smoking \|\| id:ukb-b-2134 | Major Depressive Disorder \|\| id:ieu-a-1188 | 173005 | rs2289791 | -0.3295065 | 0.07340271 | 7.155E-06 |
| Past tobacco smoking \|\| id:ukb-b-2134 | Major Depressive Disorder \|\| id:ieu-a-1188 | 173005 | rs2367724 | -0.3240497 | 0.07353368 | 1.049E-05 |
| Past tobacco smoking \|\| id:ukb-b-2134 | Major Depressive Disorder \|\| id:ieu-a-1188 | 173005 | rs2433055 | -0.3170691 | 0.07308833 | 1.4368E-05 |
| Past tobacco smoking \|\| id:ukb-b-2134 | Major Depressive Disorder \|\| id:ieu-a-1188 | 173005 | rs2587507 | -0.3274795 | 0.0734352 | 8.2176E-06 |
| Past tobacco smoking \|\| id:ukb-b-2134 | Major Depressive Disorder \|\| id:ieu-a-1188 | 173005 | rs2797793 | -0.3170122 | 0.07306746 | 1.4338E-05 |
| Past tobacco smoking \|\| id:ukb-b-2134 | Major Depressive Disorder \|\| id:ieu-a-1188 | 173005 | rs2862465 | -0.3093495 | 0.07187002 | 1.6752E-05 |
| Past tobacco smoking \|\| id:ukb-b-2134 | Major Depressive Disorder \|\| id:ieu-a-1188 | 173005 | rs28647734 | -0.3137452 | 0.07254075 | 1.5247E-05 |
| Past tobacco smoking \|\| id:ukb-b-2134 | Major Depressive Disorder \|\| id:ieu-a-1188 | 173005 | rs2866724 | -0.3119414 | 0.07261362 | 1.7398E-05 |
| Past tobacco smoking \|\| id:ukb-b-2134 | Major Depressive Disorder \|\| id:ieu-a-1188 | 173005 | rs2917670 | -0.3145972 | 0.07292941 | 1.6053E-05 |
| Past tobacco smoking \|\| id:ukb-b-2134 | Major Depressive Disorder \|\| id:ieu-a-1188 | 173005 | rs2952251 | -0.339773 | 0.07164821 | 2.1137E-06 |
| Past tobacco smoking \|\| id:ukb-b-2134 | Major Depressive Disorder \|\| id:ieu-a-1188 | 173005 | rs35445224 | -0.3325594 | 0.07293801 | 5.1281E-06 |
| Past tobacco smoking \|\| id:ukb-b-2134 | Major Depressive Disorder \|\| id:ieu-a-1188 | 173005 | rs35761479 | -0.3128208 | 0.07237532 | 1.5448E-05 |
| Past tobacco smoking \|\| id:ukb-b-2134 | Major Depressive Disorder \|\| id:ieu-a-1188 | 173005 | rs3808937 | -0.3196938 | 0.07345227 | 1.3465E-05 |
| Past tobacco smoking \|\| id:ukb-b-2134 | Major Depressive Disorder \|\| id:ieu-a-1188 | 173005 | rs3811038 | -0.3215518 | 0.07347956 | 1.2084E-05 |
| Past tobacco smoking \|\| id:ukb-b-2134 | Major Depressive Disorder \|\| id:ieu-a-1188 | 173005 | rs3827592 | -0.3248167 | 0.07363236 | 1.0274E-05 |
| Past tobacco smoking \|\| id:ukb-b-2134 | Major Depressive Disorder \|\| id:ieu-a-1188 | 173005 | rs3857914 | -0.3279837 | 0.073661 | 8.4833E-06 |
| Past tobacco smoking \|\| id:ukb-b-2134 | Major Depressive Disorder \|\| id:ieu-a-1188 | 173005 | rs3935790 | -0.3349934 | 0.07262656 | 3.9777E-06 |
| Past tobacco smoking \|\| id:ukb-b-2134 | Major Depressive Disorder \|\| id:ieu-a-1188 | 173005 | rs4708899 | -0.3306421 | 0.07313959 | 6.1636E-06 |
| Past tobacco smoking \|\| id:ukb-b-2134 | Major Depressive Disorder \|\| id:ieu-a-1188 | 173005 | rs528301 | -0.3377131 | 0.07301063 | 3.7364E-06 |
| Past tobacco smoking \|\| id:ukb-b-2134 | Major Depressive Disorder \|\| id:ieu-a-1188 | 173005 | rs540356 | -0.3201693 | 0.07360754 | 1.3634E-05 |
| Past tobacco smoking \|\| id:ukb-b-2134 | Major Depressive Disorder \|\| id:ieu-a-1188 | 173005 | rs56081685 | -0.3245991 | 0.07352068 | 1.0098E-05 |
| Past tobacco smoking \|\| id:ukb-b-2134 | Major Depressive Disorder \|\| id:ieu-a-1188 | 173005 | rs56760958 | -0.341726 | 0.07155669 | 1.7917E-06 |
| Past tobacco smoking \|\| id:ukb-b-2134 | Major Depressive Disorder \|\| id:ieu-a-1188 | 173005 | rs58400863 | -0.317435 | 0.07328615 | 1.4813E-05 |
| Past tobacco smoking \|\| id:ukb-b-2134 | Major Depressive Disorder \|\| id:ieu-a-1188 | 173005 | rs597808 | -0.3382226 | 0.0728002 | 3.3859E-06 |
| Past tobacco smoking \|\| id:ukb-b-2134 | Major Depressive Disorder \|\| id:ieu-a-1188 | 173005 | rs6141314 | -0.3145448 | 0.07294088 | 1.6155E-05 |
| Past tobacco smoking \|\| id:ukb-b-2134 | Major Depressive Disorder \|\| id:ieu-a-1188 | 173005 | rs62022627 | -0.3445986 | 0.07125346 | 1.3232E-06 |
| Past tobacco smoking \|\| id:ukb-b-2134 | Major Depressive Disorder \|\| id:ieu-a-1188 | 173005 | rs6265 | -0.3483064 | 0.07141327 | 1.0753E-06 |
| Past tobacco smoking \|\| id:ukb-b-2134 | Major Depressive Disorder \|\| id:ieu-a-1188 | 173005 | rs6464024 | -0.3350603 | 0.07284926 | 4.2378E-06 |
| Past tobacco smoking \|\| id:ukb-b-2134 | Major Depressive Disorder \|\| id:ieu-a-1188 | 173005 | rs6588376 | -0.316646 | 0.07307555 | 1.47E-05 |
| Past tobacco smoking \|\| id:ukb-b-2134 | Major Depressive Disorder \|\| id:ieu-a-1188 | 173005 | rs67174662 | -0.3249263 | 0.07359544 | 1.0099E-05 |
| Past tobacco smoking \|\| id:ukb-b-2134 | Major Depressive Disorder \|\| id:ieu-a-1188 | 173005 | rs6751705 | -0.3377435 | 0.07335614 | 4.1413E-06 |
| Past tobacco smoking \|\| id:ukb-b-2134 | Major Depressive Disorder \|\| id:ieu-a-1188 | 173005 | rs71491831 | -0.3250495 | 0.07360484 | 1.0048E-05 |
| Past tobacco smoking \|\| id:ukb-b-2134 | Major Depressive Disorder \|\| id:ieu-a-1188 | 173005 | rs73229090 | -0.3212188 | 0.07364831 | 1.2916E-05 |
| Past tobacco smoking \|\| id:ukb-b-2134 | Major Depressive Disorder \|\| id:ieu-a-1188 | 173005 | rs7333559 | -0.3302776 | 0.07327483 | 6.5632E-06 |
| Past tobacco smoking \|\| id:ukb-b-2134 | Major Depressive Disorder \|\| id:ieu-a-1188 | 173005 | rs74676797 | -0.3237431 | 0.07324806 | 9.8784E-06 |
| Past tobacco smoking \|\| id:ukb-b-2134 | Major Depressive Disorder \|\| id:ieu-a-1188 | 173005 | rs7582445 | -0.3113251 | 0.07252948 | 1.7676E-05 |
| Past tobacco smoking \|\| id:ukb-b-2134 | Major Depressive Disorder \|\| id:ieu-a-1188 | 173005 | rs7600005 | -0.3204336 | 0.0734015 | 1.2684E-05 |
| Past tobacco smoking \|\| id:ukb-b-2134 | Major Depressive Disorder \|\| id:ieu-a-1188 | 173005 | rs7609050 | -0.323295 | 0.07356508 | 1.1094E-05 |
| Past tobacco smoking \|\| id:ukb-b-2134 | Major Depressive Disorder \|\| id:ieu-a-1188 | 173005 | rs762995 | -0.3275597 | 0.07343321 | 8.1712E-06 |
| Past tobacco smoking \|\| id:ukb-b-2134 | Major Depressive Disorder \|\| id:ieu-a-1188 | 173005 | rs763053 | -0.3290552 | 0.07334888 | 7.2517E-06 |
| Past tobacco smoking \|\| id:ukb-b-2134 | Major Depressive Disorder \|\| id:ieu-a-1188 | 173005 | rs76608582 | -0.3249656 | 0.07356552 | 9.9913E-06 |
| Past tobacco smoking \|\| id:ukb-b-2134 | Major Depressive Disorder \|\| id:ieu-a-1188 | 173005 | rs77068442 | -0.3197282 | 0.07319108 | 1.2516E-05 |
| Past tobacco smoking \|\| id:ukb-b-2134 | Major Depressive Disorder \|\| id:ieu-a-1188 | 173005 | rs77304846 | -0.324898 | 0.07356493 | 1.0032E-05 |
| Past tobacco smoking \|\| id:ukb-b-2134 | Major Depressive Disorder \|\| id:ieu-a-1188 | 173005 | rs7733542 | -0.3244472 | 0.0735453 | 1.0264E-05 |
| Past tobacco smoking \|\| id:ukb-b-2134 | Major Depressive Disorder \|\| id:ieu-a-1188 | 173005 | rs7901348 | -0.3209144 | 0.0737148 | 1.3401E-05 |
| Past tobacco smoking \|\| id:ukb-b-2134 | Major Depressive Disorder \|\| id:ieu-a-1188 | 173005 | rs7969559 | -0.326908 | 0.07349692 | 8.6708E-06 |
| Past tobacco smoking \|\| id:ukb-b-2134 | Major Depressive Disorder \|\| id:ieu-a-1188 | 173005 | rs8034783 | -0.3184734 | 0.07345394 | 1.4531E-05 |
| Past tobacco smoking \|\| id:ukb-b-2134 | Major Depressive Disorder \|\| id:ieu-a-1188 | 173005 | rs8071295 | -0.3149859 | 0.07304384 | 1.6158E-05 |
| Past tobacco smoking \|\| id:ukb-b-2134 | Major Depressive Disorder \|\| id:ieu-a-1188 | 173005 | rs885011 | -0.3329242 | 0.0732736 | 5.5308E-06 |
| Past tobacco smoking \|\| id:ukb-b-2134 | Major Depressive Disorder \|\| id:ieu-a-1188 | 173005 | rs899631 | -0.3249841 | 0.0736517 | 1.0221E-05 |
| Past tobacco smoking \|\| id:ukb-b-2134 | Major Depressive Disorder \|\| id:ieu-a-1188 | 173005 | rs911773 | -0.3163011 | 0.07301814 | 1.4788E-05 |
| Past tobacco smoking \|\| id:ukb-b-2134 | Major Depressive Disorder \|\| id:ieu-a-1188 | 173005 | rs9299331 | -0.3282819 | 0.07357403 | 8.1226E-06 |
| Past tobacco smoking \|\| id:ukb-b-2134 | Major Depressive Disorder \|\| id:ieu-a-1188 | 173005 | rs9375371 | -0.3280101 | 0.07361307 | 8.3554E-06 |
| Past tobacco smoking \|\| id:ukb-b-2134 | Major Depressive Disorder \|\| id:ieu-a-1188 | 173005 | rs9381919 | -0.3137478 | 0.07283425 | 1.6496E-05 |
| Past tobacco smoking \|\| id:ukb-b-2134 | Major Depressive Disorder \|\| id:ieu-a-1188 | 173005 | rs9542750 | -0.3208015 | 0.07341907 | 1.2456E-05 |
| Past tobacco smoking \|\| id:ukb-b-2134 | Major Depressive Disorder \|\| id:ieu-a-1188 | 173005 | rs963354 | -0.3313151 | 0.0732643 | 6.1203E-06 |
| Past tobacco smoking \|\| id:ukb-b-2134 | Major Depressive Disorder \|\| id:ieu-a-1188 | 173005 | All | -0.3242529 | 0.07268611 | 8.1577E-06 |
| Nap during day \|\| id:ukb-b-4616 | Major Depressive Disorder \|\| id:ieu-a-1188 | 173005 | rs1001817 | 0.44266256 | 0.1416342 | 0.00177568 |
| Nap during day \|\| id:ukb-b-4616 | Major Depressive Disorder \|\| id:ieu-a-1188 | 173005 | rs1011024 | 0.44123131 | 0.14102962 | 0.00175616 |
| Nap during day \|\| id:ukb-b-4616 | Major Depressive Disorder \|\| id:ieu-a-1188 | 173005 | rs10150432 | 0.46949441 | 0.14132128 | 0.00089318 |
| Nap during day \|\| id:ukb-b-4616 | Major Depressive Disorder \|\| id:ieu-a-1188 | 173005 | rs10757347 | 0.44453044 | 0.14147409 | 0.00167722 |
| Nap during day \|\| id:ukb-b-4616 | Major Depressive Disorder \|\| id:ieu-a-1188 | 173005 | rs10764260 | 0.42420036 | 0.13783112 | 0.00208617 |
| Nap during day \|\| id:ukb-b-4616 | Major Depressive Disorder \|\| id:ieu-a-1188 | 173005 | rs10840017 | 0.44153231 | 0.14107253 | 0.0017491 |
| Nap during day \|\| id:ukb-b-4616 | Major Depressive Disorder \|\| id:ieu-a-1188 | 173005 | rs10868046 | 0.44651996 | 0.14173553 | 0.00163061 |
| Nap during day \|\| id:ukb-b-4616 | Major Depressive Disorder \|\| id:ieu-a-1188 | 173005 | rs10875622 | 0.44293587 | 0.1424833 | 0.00187921 |
| Nap during day \|\| id:ukb-b-4616 | Major Depressive Disorder \|\| id:ieu-a-1188 | 173005 | rs11071755 | 0.43152455 | 0.13949067 | 0.00197762 |
| Nap during day \|\| id:ukb-b-4616 | Major Depressive Disorder \|\| id:ieu-a-1188 | 173005 | rs11121194 | 0.48037232 | 0.13808545 | 0.00050366 |
| Nap during day \|\| id:ukb-b-4616 | Major Depressive Disorder \|\| id:ieu-a-1188 | 173005 | rs11125776 | 0.4354018 | 0.14114238 | 0.00203661 |
| Nap during day \|\| id:ukb-b-4616 | Major Depressive Disorder \|\| id:ieu-a-1188 | 173005 | rs11224896 | 0.45319088 | 0.14192226 | 0.00140689 |
| Nap during day \|\| id:ukb-b-4616 | Major Depressive Disorder \|\| id:ieu-a-1188 | 173005 | rs11252681 | 0.45944135 | 0.14159503 | 0.00117551 |
| Nap during day \|\| id:ukb-b-4616 | Major Depressive Disorder \|\| id:ieu-a-1188 | 173005 | rs11258652 | 0.45084486 | 0.14228101 | 0.00153126 |
| Nap during day \|\| id:ukb-b-4616 | Major Depressive Disorder \|\| id:ieu-a-1188 | 173005 | rs113886333 | 0.44399657 | 0.14139438 | 0.00168874 |
| Nap during day \|\| id:ukb-b-4616 | Major Depressive Disorder \|\| id:ieu-a-1188 | 173005 | rs11615756 | 0.49373713 | 0.14474098 | 0.00064683 |
| Nap during day \|\| id:ukb-b-4616 | Major Depressive Disorder \|\| id:ieu-a-1188 | 173005 | rs12042846 | 0.45655973 | 0.14187579 | 0.00129077 |
| Nap during day \|\| id:ukb-b-4616 | Major Depressive Disorder \|\| id:ieu-a-1188 | 173005 | rs12140153 | 0.41818923 | 0.14235002 | 0.003306 |
| Nap during day \|\| id:ukb-b-4616 | Major Depressive Disorder \|\| id:ieu-a-1188 | 173005 | rs12346996 | 0.44774376 | 0.14188486 | 0.00160122 |
| Nap during day \|\| id:ukb-b-4616 | Major Depressive Disorder \|\| id:ieu-a-1188 | 173005 | rs12451365 | 0.45897638 | 0.14224782 | 0.00125272 |
| Nap during day \|\| id:ukb-b-4616 | Major Depressive Disorder \|\| id:ieu-a-1188 | 173005 | rs12615434 | 0.43267661 | 0.13994803 | 0.00199017 |
| Nap during day \|\| id:ukb-b-4616 | Major Depressive Disorder \|\| id:ieu-a-1188 | 173005 | rs12657723 | 0.48325671 | 0.13853586 | 0.00048608 |
| Nap during day \|\| id:ukb-b-4616 | Major Depressive Disorder \|\| id:ieu-a-1188 | 173005 | rs12992648 | 0.46061291 | 0.14168865 | 0.00115048 |
| Nap during day \|\| id:ukb-b-4616 | Major Depressive Disorder \|\| id:ieu-a-1188 | 173005 | rs13033444 | 0.45173037 | 0.14226339 | 0.00149677 |
| Nap during day \|\| id:ukb-b-4616 | Major Depressive Disorder \|\| id:ieu-a-1188 | 173005 | rs13266972 | 0.46410731 | 0.14126649 | 0.00101862 |
| Nap during day \|\| id:ukb-b-4616 | Major Depressive Disorder \|\| id:ieu-a-1188 | 173005 | rs13284688 | 0.43960261 | 0.14292006 | 0.00209894 |
| Nap during day \|\| id:ukb-b-4616 | Major Depressive Disorder \|\| id:ieu-a-1188 | 173005 | rs1479116 | 0.45548841 | 0.14203831 | 0.0013422 |
| Nap during day \|\| id:ukb-b-4616 | Major Depressive Disorder \|\| id:ieu-a-1188 | 173005 | rs17158413 | 0.44598615 | 0.14177046 | 0.00165615 |
| Nap during day \|\| id:ukb-b-4616 | Major Depressive Disorder \|\| id:ieu-a-1188 | 173005 | rs17265513 | 0.44996076 | 0.14192915 | 0.00152271 |
| Nap during day \|\| id:ukb-b-4616 | Major Depressive Disorder \|\| id:ieu-a-1188 | 173005 | rs174541 | 0.42678755 | 0.14080435 | 0.0024369 |
| Nap during day \|\| id:ukb-b-4616 | Major Depressive Disorder \|\| id:ieu-a-1188 | 173005 | rs1883048 | 0.47141784 | 0.14075684 | 0.00081056 |
| Nap during day \|\| id:ukb-b-4616 | Major Depressive Disorder \|\| id:ieu-a-1188 | 173005 | rs2033103 | 0.45613374 | 0.14205489 | 0.00132289 |
| Nap during day \|\| id:ukb-b-4616 | Major Depressive Disorder \|\| id:ieu-a-1188 | 173005 | rs2099810 | 0.46940457 | 0.14116084 | 0.00088318 |
| Nap during day \|\| id:ukb-b-4616 | Major Depressive Disorder \|\| id:ieu-a-1188 | 173005 | rs224111 | 0.43814905 | 0.14071642 | 0.00184756 |
| Nap during day \|\| id:ukb-b-4616 | Major Depressive Disorder \|\| id:ieu-a-1188 | 173005 | rs2284016 | 0.43807885 | 0.14081279 | 0.00186409 |
| Nap during day \|\| id:ukb-b-4616 | Major Depressive Disorder \|\| id:ieu-a-1188 | 173005 | rs2370926 | 0.48921565 | 0.13736429 | 0.00036882 |
| Nap during day \|\| id:ukb-b-4616 | Major Depressive Disorder \|\| id:ieu-a-1188 | 173005 | rs2390669 | 0.47218762 | 0.14010039 | 0.00075072 |
| Nap during day \|\| id:ukb-b-4616 | Major Depressive Disorder \|\| id:ieu-a-1188 | 173005 | rs2653349 | 0.44401012 | 0.14375104 | 0.00201005 |
| Nap during day \|\| id:ukb-b-4616 | Major Depressive Disorder \|\| id:ieu-a-1188 | 173005 | rs2769916 | 0.45469067 | 0.14219451 | 0.00138544 |
| Nap during day \|\| id:ukb-b-4616 | Major Depressive Disorder \|\| id:ieu-a-1188 | 173005 | rs2786547 | 0.47985058 | 0.14009103 | 0.00061417 |
| Nap during day \|\| id:ukb-b-4616 | Major Depressive Disorder \|\| id:ieu-a-1188 | 173005 | rs34262487 | 0.42742483 | 0.13932492 | 0.00215621 |
| Nap during day \|\| id:ukb-b-4616 | Major Depressive Disorder \|\| id:ieu-a-1188 | 173005 | rs35011311 | 0.46665527 | 0.14096428 | 0.00093146 |
| Nap during day \|\| id:ukb-b-4616 | Major Depressive Disorder \|\| id:ieu-a-1188 | 173005 | rs351776 | 0.44812849 | 0.14198301 | 0.00159833 |
| Nap during day \|\| id:ukb-b-4616 | Major Depressive Disorder \|\| id:ieu-a-1188 | 173005 | rs35851551 | 0.45614165 | 0.1418681 | 0.0013033 |
| Nap during day \|\| id:ukb-b-4616 | Major Depressive Disorder \|\| id:ieu-a-1188 | 173005 | rs3810484 | 0.467667 | 0.14044809 | 0.00086902 |
| Nap during day \|\| id:ukb-b-4616 | Major Depressive Disorder \|\| id:ieu-a-1188 | 173005 | rs3935190 | 0.46840535 | 0.14110537 | 0.00090165 |
| Nap during day \|\| id:ukb-b-4616 | Major Depressive Disorder \|\| id:ieu-a-1188 | 173005 | rs40005 | 0.45524429 | 0.14192977 | 0.00133879 |
| Nap during day \|\| id:ukb-b-4616 | Major Depressive Disorder \|\| id:ieu-a-1188 | 173005 | rs4402351 | 0.44973394 | 0.14226271 | 0.00157071 |
| Nap during day \|\| id:ukb-b-4616 | Major Depressive Disorder \|\| id:ieu-a-1188 | 173005 | rs4587762 | 0.42492195 | 0.13749412 | 0.00199838 |
| Nap during day \|\| id:ukb-b-4616 | Major Depressive Disorder \|\| id:ieu-a-1188 | 173005 | rs467897 | 0.46541227 | 0.14185894 | 0.00103509 |
| Nap during day \|\| id:ukb-b-4616 | Major Depressive Disorder \|\| id:ieu-a-1188 | 173005 | rs4692709 | 0.44525157 | 0.14163945 | 0.00166908 |
| Nap during day \|\| id:ukb-b-4616 | Major Depressive Disorder \|\| id:ieu-a-1188 | 173005 | rs4856536 | 0.46520334 | 0.14148734 | 0.0010092 |
| Nap during day \|\| id:ukb-b-4616 | Major Depressive Disorder \|\| id:ieu-a-1188 | 173005 | rs60920123 | 0.46556608 | 0.14140968 | 0.00099364 |
| Nap during day \|\| id:ukb-b-4616 | Major Depressive Disorder \|\| id:ieu-a-1188 | 173005 | rs614987 | 0.45845659 | 0.14279448 | 0.00132456 |
| Nap during day \|\| id:ukb-b-4616 | Major Depressive Disorder \|\| id:ieu-a-1188 | 173005 | rs62425620 | 0.46033352 | 0.14175914 | 0.00116508 |
| Nap during day \|\| id:ukb-b-4616 | Major Depressive Disorder \|\| id:ieu-a-1188 | 173005 | rs62560863 | 0.45914585 | 0.14181576 | 0.0012053 |
| Nap during day \|\| id:ukb-b-4616 | Major Depressive Disorder \|\| id:ieu-a-1188 | 173005 | rs6452787 | 0.45257816 | 0.14141556 | 0.00137265 |
| Nap during day \|\| id:ukb-b-4616 | Major Depressive Disorder \|\| id:ieu-a-1188 | 173005 | rs6483215 | 0.43838922 | 0.14093341 | 0.001867 |
| Nap during day \|\| id:ukb-b-4616 | Major Depressive Disorder \|\| id:ieu-a-1188 | 173005 | rs6919087 | 0.42680322 | 0.14077622 | 0.00243112 |
| Nap during day \|\| id:ukb-b-4616 | Major Depressive Disorder \|\| id:ieu-a-1188 | 173005 | rs6942927 | 0.45509216 | 0.14170331 | 0.00132005 |
| Nap during day \|\| id:ukb-b-4616 | Major Depressive Disorder \|\| id:ieu-a-1188 | 173005 | rs7038206 | 0.45720941 | 0.14197121 | 0.00127995 |
| Nap during day \|\| id:ukb-b-4616 | Major Depressive Disorder \|\| id:ieu-a-1188 | 173005 | rs7191614 | 0.46158092 | 0.14163329 | 0.00111811 |
| Nap during day \|\| id:ukb-b-4616 | Major Depressive Disorder \|\| id:ieu-a-1188 | 173005 | rs7198121 | 0.4656131 | 0.14115594 | 0.00097178 |
| Nap during day \|\| id:ukb-b-4616 | Major Depressive Disorder \|\| id:ieu-a-1188 | 173005 | rs72781017 | 0.43956607 | 0.14123807 | 0.00185677 |
| Nap during day \|\| id:ukb-b-4616 | Major Depressive Disorder \|\| id:ieu-a-1188 | 173005 | rs7422655 | 0.44333641 | 0.1415178 | 0.00173191 |
| Nap during day \|\| id:ukb-b-4616 | Major Depressive Disorder \|\| id:ieu-a-1188 | 173005 | rs75022160 | 0.48364273 | 0.13704984 | 0.0004172 |
| Nap during day \|\| id:ukb-b-4616 | Major Depressive Disorder \|\| id:ieu-a-1188 | 173005 | rs75411336 | 0.46455477 | 0.14063222 | 0.00095544 |
| Nap during day \|\| id:ukb-b-4616 | Major Depressive Disorder \|\| id:ieu-a-1188 | 173005 | rs7555990 | 0.47376569 | 0.13998014 | 0.00071303 |
| Nap during day \|\| id:ukb-b-4616 | Major Depressive Disorder \|\| id:ieu-a-1188 | 173005 | rs77154532 | 0.4511662 | 0.14192326 | 0.00147812 |
| Nap during day \|\| id:ukb-b-4616 | Major Depressive Disorder \|\| id:ieu-a-1188 | 173005 | rs7752899 | 0.45141191 | 0.14228357 | 0.00151069 |
| Nap during day \|\| id:ukb-b-4616 | Major Depressive Disorder \|\| id:ieu-a-1188 | 173005 | rs7814873 | 0.45953532 | 0.14163143 | 0.00117622 |
| Nap during day \|\| id:ukb-b-4616 | Major Depressive Disorder \|\| id:ieu-a-1188 | 173005 | rs785145 | 0.44310482 | 0.14146665 | 0.00173489 |
| Nap during day \|\| id:ukb-b-4616 | Major Depressive Disorder \|\| id:ieu-a-1188 | 173005 | rs8050478 | 0.46659332 | 0.14104566 | 0.00093931 |
| Nap during day \|\| id:ukb-b-4616 | Major Depressive Disorder \|\| id:ieu-a-1188 | 173005 | rs903678 | 0.42683291 | 0.14262676 | 0.00276561 |
| Nap during day \|\| id:ukb-b-4616 | Major Depressive Disorder \|\| id:ieu-a-1188 | 173005 | rs962247 | 0.4409298 | 0.14162466 | 0.00184965 |
| Nap during day \|\| id:ukb-b-4616 | Major Depressive Disorder \|\| id:ieu-a-1188 | 173005 | rs971415 | 0.46698802 | 0.14123288 | 0.00094466 |
| Nap during day \|\| id:ukb-b-4616 | Major Depressive Disorder \|\| id:ieu-a-1188 | 173005 | rs9965170 | 0.46387547 | 0.14406147 | 0.00128198 |
| Nap during day \|\| id:ukb-b-4616 | Major Depressive Disorder \|\| id:ieu-a-1188 | 173005 | All | 0.45354142 | 0.14034992 | 0.0012314 |
| Age completed full time education \|\| id:ukb-b-6134 | Major Depressive Disorder \|\| id:ieu-a-1188 | 173005 | rs10189857 | -0.4892696 | 0.15381102 | 0.00146778 |
| Age completed full time education \|\| id:ukb-b-6134 | Major Depressive Disorder \|\| id:ieu-a-1188 | 173005 | rs10200379 | -0.4388715 | 0.15622661 | 0.00496651 |
| Age completed full time education \|\| id:ukb-b-6134 | Major Depressive Disorder \|\| id:ieu-a-1188 | 173005 | rs10760199 | -0.4710576 | 0.15598947 | 0.00252938 |
| Age completed full time education \|\| id:ukb-b-6134 | Major Depressive Disorder \|\| id:ieu-a-1188 | 173005 | rs10953765 | -0.4636357 | 0.15697521 | 0.00314132 |
| Age completed full time education \|\| id:ukb-b-6134 | Major Depressive Disorder \|\| id:ieu-a-1188 | 173005 | rs13064576 | -0.4147155 | 0.15385833 | 0.00702963 |
| Age completed full time education \|\| id:ukb-b-6134 | Major Depressive Disorder \|\| id:ieu-a-1188 | 173005 | rs13238996 | -0.4567972 | 0.15630985 | 0.00347365 |
| Age completed full time education \|\| id:ukb-b-6134 | Major Depressive Disorder \|\| id:ieu-a-1188 | 173005 | rs13274119 | -0.4651767 | 0.15694054 | 0.00303637 |
| Age completed full time education \|\| id:ukb-b-6134 | Major Depressive Disorder \|\| id:ieu-a-1188 | 173005 | rs13394374 | -0.4787303 | 0.15526859 | 0.0020476 |
| Age completed full time education \|\| id:ukb-b-6134 | Major Depressive Disorder \|\| id:ieu-a-1188 | 173005 | rs1462163 | -0.4925279 | 0.15326138 | 0.00131061 |
| Age completed full time education \|\| id:ukb-b-6134 | Major Depressive Disorder \|\| id:ieu-a-1188 | 173005 | rs1557341 | -0.4120256 | 0.14841843 | 0.00550139 |
| Age completed full time education \|\| id:ukb-b-6134 | Major Depressive Disorder \|\| id:ieu-a-1188 | 173005 | rs17563464 | -0.5052133 | 0.14789601 | 0.00063547 |
| Age completed full time education \|\| id:ukb-b-6134 | Major Depressive Disorder \|\| id:ieu-a-1188 | 173005 | rs178217 | -0.5048864 | 0.14753104 | 0.00062108 |
| Age completed full time education \|\| id:ukb-b-6134 | Major Depressive Disorder \|\| id:ieu-a-1188 | 173005 | rs2588962 | -0.4306827 | 0.15596701 | 0.00575594 |
| Age completed full time education \|\| id:ukb-b-6134 | Major Depressive Disorder \|\| id:ieu-a-1188 | 173005 | rs2709814 | -0.4584675 | 0.15639641 | 0.00337389 |
| Age completed full time education \|\| id:ukb-b-6134 | Major Depressive Disorder \|\| id:ieu-a-1188 | 173005 | rs34945223 | -0.4130462 | 0.14857325 | 0.00543448 |
| Age completed full time education \|\| id:ukb-b-6134 | Major Depressive Disorder \|\| id:ieu-a-1188 | 173005 | rs4557720 | -0.4668146 | 0.1563671 | 0.0028323 |
| Age completed full time education \|\| id:ukb-b-6134 | Major Depressive Disorder \|\| id:ieu-a-1188 | 173005 | rs4674403 | -0.4560282 | 0.15623441 | 0.00351301 |
| Age completed full time education \|\| id:ukb-b-6134 | Major Depressive Disorder \|\| id:ieu-a-1188 | 173005 | rs4731951 | -0.4669423 | 0.15658054 | 0.00286258 |
| Age completed full time education \|\| id:ukb-b-6134 | Major Depressive Disorder \|\| id:ieu-a-1188 | 173005 | rs57513571 | -0.4860541 | 0.15381238 | 0.00157744 |
| Age completed full time education \|\| id:ukb-b-6134 | Major Depressive Disorder \|\| id:ieu-a-1188 | 173005 | rs62039529 | -0.4639569 | 0.1565242 | 0.00303548 |
| Age completed full time education \|\| id:ukb-b-6134 | Major Depressive Disorder \|\| id:ieu-a-1188 | 173005 | rs6449503 | -0.4721144 | 0.15563691 | 0.00241787 |
| Age completed full time education \|\| id:ukb-b-6134 | Major Depressive Disorder \|\| id:ieu-a-1188 | 173005 | rs6508344 | -0.4719488 | 0.15590963 | 0.0024694 |
| Age completed full time education \|\| id:ukb-b-6134 | Major Depressive Disorder \|\| id:ieu-a-1188 | 173005 | rs6679399 | -0.4883847 | 0.15145461 | 0.00126137 |
| Age completed full time education \|\| id:ukb-b-6134 | Major Depressive Disorder \|\| id:ieu-a-1188 | 173005 | rs6729586 | -0.423597 | 0.15109993 | 0.00505633 |
| Age completed full time education \|\| id:ukb-b-6134 | Major Depressive Disorder \|\| id:ieu-a-1188 | 173005 | rs6754311 | -0.4563499 | 0.15622908 | 0.00348876 |
| Age completed full time education \|\| id:ukb-b-6134 | Major Depressive Disorder \|\| id:ieu-a-1188 | 173005 | rs68191270 | -0.430755 | 0.15489073 | 0.00541875 |
| Age completed full time education \|\| id:ukb-b-6134 | Major Depressive Disorder \|\| id:ieu-a-1188 | 173005 | rs6931604 | -0.5068885 | 0.15066032 | 0.00076698 |
| Age completed full time education \|\| id:ukb-b-6134 | Major Depressive Disorder \|\| id:ieu-a-1188 | 173005 | rs7110786 | -0.4429359 | 0.15587115 | 0.00448766 |
| Age completed full time education \|\| id:ukb-b-6134 | Major Depressive Disorder \|\| id:ieu-a-1188 | 173005 | rs7768758 | -0.446897 | 0.15606989 | 0.00419066 |
| Age completed full time education \|\| id:ukb-b-6134 | Major Depressive Disorder \|\| id:ieu-a-1188 | 173005 | rs7896518 | -0.4403268 | 0.15650963 | 0.00490181 |
| Age completed full time education \|\| id:ukb-b-6134 | Major Depressive Disorder \|\| id:ieu-a-1188 | 173005 | rs7975763 | -0.4395931 | 0.15586221 | 0.00479645 |
| Age completed full time education \|\| id:ukb-b-6134 | Major Depressive Disorder \|\| id:ieu-a-1188 | 173005 | rs9536961 | -0.4338311 | 0.1549634 | 0.00511705 |
| Age completed full time education \|\| id:ukb-b-6134 | Major Depressive Disorder \|\| id:ieu-a-1188 | 173005 | rs9655780 | -0.4657598 | 0.15705726 | 0.0030215 |
| Age completed full time education \|\| id:ukb-b-6134 | Major Depressive Disorder \|\| id:ieu-a-1188 | 173005 | rs9866630 | -0.4922399 | 0.15356372 | 0.00134854 |
| Age completed full time education \|\| id:ukb-b-6134 | Major Depressive Disorder \|\| id:ieu-a-1188 | 173005 | All | -0.4602687 | 0.15210803 | 0.00247867 |
| Average total household income before tax \|\| id:ukb-b-7408 | Major Depressive Disorder \|\| id:ieu-a-1188 | 173005 | rs10429582 | -0.4145118 | 0.09278292 | 7.9122E-06 |
| Average total household income before tax \|\| id:ukb-b-7408 | Major Depressive Disorder \|\| id:ieu-a-1188 | 173005 | rs10761035 | -0.3857577 | 0.09223061 | 2.8828E-05 |
| Average total household income before tax \|\| id:ukb-b-7408 | Major Depressive Disorder \|\| id:ieu-a-1188 | 173005 | rs11191116 | -0.3857697 | 0.09234192 | 2.9457E-05 |
| Average total household income before tax \|\| id:ukb-b-7408 | Major Depressive Disorder \|\| id:ieu-a-1188 | 173005 | rs11588857 | -0.3947502 | 0.09239465 | 1.9335E-05 |
| Average total household income before tax \|\| id:ukb-b-7408 | Major Depressive Disorder \|\| id:ieu-a-1188 | 173005 | rs11678501 | -0.4066187 | 0.08982229 | 5.9848E-06 |
| Average total household income before tax \|\| id:ukb-b-7408 | Major Depressive Disorder \|\| id:ieu-a-1188 | 173005 | rs11714337 | -0.3866695 | 0.09233026 | 2.8155E-05 |
| Average total household income before tax \|\| id:ukb-b-7408 | Major Depressive Disorder \|\| id:ieu-a-1188 | 173005 | rs11877758 | -0.3537128 | 0.08763565 | 5.433E-05 |
| Average total household income before tax \|\| id:ukb-b-7408 | Major Depressive Disorder \|\| id:ieu-a-1188 | 173005 | rs11917431 | -0.3623617 | 0.09076206 | 6.5398E-05 |
| Average total household income before tax \|\| id:ukb-b-7408 | Major Depressive Disorder \|\| id:ieu-a-1188 | 173005 | rs1229984 | -0.3987496 | 0.09151107 | 1.3162E-05 |
| Average total household income before tax \|\| id:ukb-b-7408 | Major Depressive Disorder \|\| id:ieu-a-1188 | 173005 | rs12531825 | -0.3970607 | 0.09206599 | 1.6121E-05 |
| Average total household income before tax \|\| id:ukb-b-7408 | Major Depressive Disorder \|\| id:ieu-a-1188 | 173005 | rs12692596 | -0.3995214 | 0.09131017 | 1.2119E-05 |
| Average total household income before tax \|\| id:ukb-b-7408 | Major Depressive Disorder \|\| id:ieu-a-1188 | 173005 | rs12883788 | -0.386741 | 0.09272532 | 3.035E-05 |
| Average total household income before tax \|\| id:ukb-b-7408 | Major Depressive Disorder \|\| id:ieu-a-1188 | 173005 | rs1421334 | -0.3881402 | 0.09243208 | 2.6787E-05 |
| Average total household income before tax \|\| id:ukb-b-7408 | Major Depressive Disorder \|\| id:ieu-a-1188 | 173005 | rs2068428 | -0.4097661 | 0.08850664 | 3.6606E-06 |
| Average total household income before tax \|\| id:ukb-b-7408 | Major Depressive Disorder \|\| id:ieu-a-1188 | 173005 | rs2332719 | -0.3923633 | 0.09235849 | 2.1543E-05 |
| Average total household income before tax \|\| id:ukb-b-7408 | Major Depressive Disorder \|\| id:ieu-a-1188 | 173005 | rs2362523 | -0.3926824 | 0.09213802 | 2.027E-05 |
| Average total household income before tax \|\| id:ukb-b-7408 | Major Depressive Disorder \|\| id:ieu-a-1188 | 173005 | rs2422859 | -0.3783137 | 0.09192243 | 3.8622E-05 |
| Average total household income before tax \|\| id:ukb-b-7408 | Major Depressive Disorder \|\| id:ieu-a-1188 | 173005 | rs2515919 | -0.3819539 | 0.09210006 | 3.3662E-05 |
| Average total household income before tax \|\| id:ukb-b-7408 | Major Depressive Disorder \|\| id:ieu-a-1188 | 173005 | rs2820314 | -0.3762342 | 0.09151126 | 3.9336E-05 |
| Average total household income before tax \|\| id:ukb-b-7408 | Major Depressive Disorder \|\| id:ieu-a-1188 | 173005 | rs32940 | -0.4121355 | 0.09028471 | 4.9987E-06 |
| Average total household income before tax \|\| id:ukb-b-7408 | Major Depressive Disorder \|\| id:ieu-a-1188 | 173005 | rs34473884 | -0.3836848 | 0.09214214 | 3.1265E-05 |
| Average total household income before tax \|\| id:ukb-b-7408 | Major Depressive Disorder \|\| id:ieu-a-1188 | 173005 | rs387780 | -0.380789 | 0.09172515 | 3.3043E-05 |
| Average total household income before tax \|\| id:ukb-b-7408 | Major Depressive Disorder \|\| id:ieu-a-1188 | 173005 | rs488786 | -0.4016732 | 0.09104396 | 1.0249E-05 |
| Average total household income before tax \|\| id:ukb-b-7408 | Major Depressive Disorder \|\| id:ieu-a-1188 | 173005 | rs5754738 | -0.4003587 | 0.09108898 | 1.1065E-05 |
| Average total household income before tax \|\| id:ukb-b-7408 | Major Depressive Disorder \|\| id:ieu-a-1188 | 173005 | rs6035877 | -0.4041516 | 0.09023277 | 7.4997E-06 |
| Average total household income before tax \|\| id:ukb-b-7408 | Major Depressive Disorder \|\| id:ieu-a-1188 | 173005 | rs62183028 | -0.3730584 | 0.09151743 | 4.5745E-05 |
| Average total household income before tax \|\| id:ukb-b-7408 | Major Depressive Disorder \|\| id:ieu-a-1188 | 173005 | rs6429636 | -0.3628527 | 0.08929916 | 4.8376E-05 |
| Average total household income before tax \|\| id:ukb-b-7408 | Major Depressive Disorder \|\| id:ieu-a-1188 | 173005 | rs6699397 | -0.3799078 | 0.09250605 | 4.011E-05 |
| Average total household income before tax \|\| id:ukb-b-7408 | Major Depressive Disorder \|\| id:ieu-a-1188 | 173005 | rs6868457 | -0.4030801 | 0.09241355 | 1.2906E-05 |
| Average total household income before tax \|\| id:ukb-b-7408 | Major Depressive Disorder \|\| id:ieu-a-1188 | 173005 | rs71576284 | -0.3926061 | 0.09099099 | 1.5976E-05 |
| Average total household income before tax \|\| id:ukb-b-7408 | Major Depressive Disorder \|\| id:ieu-a-1188 | 173005 | rs73015322 | -0.3929851 | 0.09206903 | 1.969E-05 |
| Average total household income before tax \|\| id:ukb-b-7408 | Major Depressive Disorder \|\| id:ieu-a-1188 | 173005 | rs75413320 | -0.3859491 | 0.09242441 | 2.969E-05 |
| Average total household income before tax \|\| id:ukb-b-7408 | Major Depressive Disorder \|\| id:ieu-a-1188 | 173005 | rs7700107 | -0.369838 | 0.09019327 | 4.1225E-05 |
| Average total household income before tax \|\| id:ukb-b-7408 | Major Depressive Disorder \|\| id:ieu-a-1188 | 173005 | rs77126132 | -0.3855332 | 0.09229073 | 2.9489E-05 |
| Average total household income before tax \|\| id:ukb-b-7408 | Major Depressive Disorder \|\| id:ieu-a-1188 | 173005 | rs784256 | -0.3621836 | 0.09012722 | 5.8549E-05 |
| Average total household income before tax \|\| id:ukb-b-7408 | Major Depressive Disorder \|\| id:ieu-a-1188 | 173005 | rs7896518 | -0.3772559 | 0.09139578 | 3.6638E-05 |
| Average total household income before tax \|\| id:ukb-b-7408 | Major Depressive Disorder \|\| id:ieu-a-1188 | 173005 | rs9388490 | -0.3683709 | 0.08957638 | 3.9162E-05 |
| Average total household income before tax \|\| id:ukb-b-7408 | Major Depressive Disorder \|\| id:ieu-a-1188 | 173005 | rs9556958 | -0.3929999 | 0.09215011 | 2.001E-05 |
| Average total household income before tax \|\| id:ukb-b-7408 | Major Depressive Disorder \|\| id:ieu-a-1188 | 173005 | rs968050 | -0.4228999 | 0.08960123 | 2.3608E-06 |
| Average total household income before tax \|\| id:ukb-b-7408 | Major Depressive Disorder \|\| id:ieu-a-1188 | 173005 | rs9891103 | -0.3699274 | 0.0912842 | 5.0678E-05 |
| Average total household income before tax \|\| id:ukb-b-7408 | Major Depressive Disorder \|\| id:ieu-a-1188 | 173005 | All | -0.3878278 | 0.09019423 | 1.7086E-05 |
| Smoking status: Never \|\| id:ukb-d-20116_0 | Major Depressive Disorder \|\| id:ieu-a-1188 | 173005 | rs10193706 | -1.0887816 | 0.190421 | 1.0794E-08 |
| Smoking status: Never \|\| id:ukb-d-20116_0 | Major Depressive Disorder \|\| id:ieu-a-1188 | 173005 | rs10233018 | -1.011793 | 0.17872741 | 1.5041E-08 |
| Smoking status: Never \|\| id:ukb-d-20116_0 | Major Depressive Disorder \|\| id:ieu-a-1188 | 173005 | rs10274594 | -1.0685766 | 0.18826602 | 1.3797E-08 |
| Smoking status: Never \|\| id:ukb-d-20116_0 | Major Depressive Disorder \|\| id:ieu-a-1188 | 173005 | rs1029986 | -1.0557079 | 0.18653983 | 1.5188E-08 |
| Smoking status: Never \|\| id:ukb-d-20116_0 | Major Depressive Disorder \|\| id:ieu-a-1188 | 173005 | rs10774625 | -1.1092914 | 0.18396811 | 1.6416E-09 |
| Smoking status: Never \|\| id:ukb-d-20116_0 | Major Depressive Disorder \|\| id:ieu-a-1188 | 173005 | rs10813628 | -1.0697783 | 0.18804179 | 1.2775E-08 |
| Smoking status: Never \|\| id:ukb-d-20116_0 | Major Depressive Disorder \|\| id:ieu-a-1188 | 173005 | rs10897561 | -1.0676239 | 0.18771896 | 1.2902E-08 |
| Smoking status: Never \|\| id:ukb-d-20116_0 | Major Depressive Disorder \|\| id:ieu-a-1188 | 173005 | rs10905461 | -1.0794412 | 0.18772523 | 8.9184E-09 |
| Smoking status: Never \|\| id:ukb-d-20116_0 | Major Depressive Disorder \|\| id:ieu-a-1188 | 173005 | rs10914684 | -1.0648964 | 0.18799374 | 1.4743E-08 |
| Smoking status: Never \|\| id:ukb-d-20116_0 | Major Depressive Disorder \|\| id:ieu-a-1188 | 173005 | rs10956808 | -1.0759963 | 0.18837013 | 1.1157E-08 |
| Smoking status: Never \|\| id:ukb-d-20116_0 | Major Depressive Disorder \|\| id:ieu-a-1188 | 173005 | rs11103667 | -1.0528477 | 0.18628095 | 1.5865E-08 |
| Smoking status: Never \|\| id:ukb-d-20116_0 | Major Depressive Disorder \|\| id:ieu-a-1188 | 173005 | rs11127913 | -1.1377498 | 0.17902001 | 2.0784E-10 |
| Smoking status: Never \|\| id:ukb-d-20116_0 | Major Depressive Disorder \|\| id:ieu-a-1188 | 173005 | rs11611651 | -1.0735691 | 0.18794416 | 1.1154E-08 |
| Smoking status: Never \|\| id:ukb-d-20116_0 | Major Depressive Disorder \|\| id:ieu-a-1188 | 173005 | rs11631530 | -1.1214378 | 0.18013173 | 4.7955E-10 |
| Smoking status: Never \|\| id:ukb-d-20116_0 | Major Depressive Disorder \|\| id:ieu-a-1188 | 173005 | rs11646575 | -1.0679336 | 0.18858178 | 1.4877E-08 |
| Smoking status: Never \|\| id:ukb-d-20116_0 | Major Depressive Disorder \|\| id:ieu-a-1188 | 173005 | rs12042107 | -1.0481462 | 0.1862547 | 1.8285E-08 |
| Smoking status: Never \|\| id:ukb-d-20116_0 | Major Depressive Disorder \|\| id:ieu-a-1188 | 173005 | rs12450028 | -1.0778462 | 0.18796157 | 9.786E-09 |
| Smoking status: Never \|\| id:ukb-d-20116_0 | Major Depressive Disorder \|\| id:ieu-a-1188 | 173005 | rs12479064 | -1.0710747 | 0.18778944 | 1.1731E-08 |
| Smoking status: Never \|\| id:ukb-d-20116_0 | Major Depressive Disorder \|\| id:ieu-a-1188 | 173005 | rs12487411 | -1.0643302 | 0.18812357 | 1.5349E-08 |
| Smoking status: Never \|\| id:ukb-d-20116_0 | Major Depressive Disorder \|\| id:ieu-a-1188 | 173005 | rs12608052 | -1.0732712 | 0.18795079 | 1.1272E-08 |
| Smoking status: Never \|\| id:ukb-d-20116_0 | Major Depressive Disorder \|\| id:ieu-a-1188 | 173005 | rs12910916 | -1.0182104 | 0.18024721 | 1.6142E-08 |
| Smoking status: Never \|\| id:ukb-d-20116_0 | Major Depressive Disorder \|\| id:ieu-a-1188 | 173005 | rs1499982 | -1.0550423 | 0.18789139 | 1.9637E-08 |
| Smoking status: Never \|\| id:ukb-d-20116_0 | Major Depressive Disorder \|\| id:ieu-a-1188 | 173005 | rs1561195 | -1.0743281 | 0.18706706 | 9.3008E-09 |
| Smoking status: Never \|\| id:ukb-d-20116_0 | Major Depressive Disorder \|\| id:ieu-a-1188 | 173005 | rs16951001 | -1.0764976 | 0.18790886 | 1.0113E-08 |
| Smoking status: Never \|\| id:ukb-d-20116_0 | Major Depressive Disorder \|\| id:ieu-a-1188 | 173005 | rs17003752 | -1.0680571 | 0.18789253 | 1.3127E-08 |
| Smoking status: Never \|\| id:ukb-d-20116_0 | Major Depressive Disorder \|\| id:ieu-a-1188 | 173005 | rs17151637 | -1.1117047 | 0.18369519 | 1.4315E-09 |
| Smoking status: Never \|\| id:ukb-d-20116_0 | Major Depressive Disorder \|\| id:ieu-a-1188 | 173005 | rs1899896 | -1.0782778 | 0.18821319 | 1.0101E-08 |
| Smoking status: Never \|\| id:ukb-d-20116_0 | Major Depressive Disorder \|\| id:ieu-a-1188 | 173005 | rs2416770 | -1.0835942 | 0.18756691 | 7.5996E-09 |
| Smoking status: Never \|\| id:ukb-d-20116_0 | Major Depressive Disorder \|\| id:ieu-a-1188 | 173005 | rs2675609 | -1.0671111 | 0.18841611 | 1.4824E-08 |
| Smoking status: Never \|\| id:ukb-d-20116_0 | Major Depressive Disorder \|\| id:ieu-a-1188 | 173005 | rs2797116 | -1.0718593 | 0.18799236 | 1.1868E-08 |
| Smoking status: Never \|\| id:ukb-d-20116_0 | Major Depressive Disorder \|\| id:ieu-a-1188 | 173005 | rs2867749 | -1.0788564 | 0.18782687 | 9.2527E-09 |
| Smoking status: Never \|\| id:ukb-d-20116_0 | Major Depressive Disorder \|\| id:ieu-a-1188 | 173005 | rs299688 | -1.0893174 | 0.18705263 | 5.7598E-09 |
| Smoking status: Never \|\| id:ukb-d-20116_0 | Major Depressive Disorder \|\| id:ieu-a-1188 | 173005 | rs326341 | -1.0807099 | 0.18783977 | 8.7487E-09 |
| Smoking status: Never \|\| id:ukb-d-20116_0 | Major Depressive Disorder \|\| id:ieu-a-1188 | 173005 | rs35891966 | -1.0438579 | 0.18549562 | 1.8295E-08 |
| Smoking status: Never \|\| id:ukb-d-20116_0 | Major Depressive Disorder \|\| id:ieu-a-1188 | 173005 | rs379525 | -1.0818356 | 0.18751496 | 7.9588E-09 |
| Smoking status: Never \|\| id:ukb-d-20116_0 | Major Depressive Disorder \|\| id:ieu-a-1188 | 173005 | rs42417 | -1.0872415 | 0.18726426 | 6.4013E-09 |
| Smoking status: Never \|\| id:ukb-d-20116_0 | Major Depressive Disorder \|\| id:ieu-a-1188 | 173005 | rs4566215 | -1.0648461 | 0.18736494 | 1.3214E-08 |
| Smoking status: Never \|\| id:ukb-d-20116_0 | Major Depressive Disorder \|\| id:ieu-a-1188 | 173005 | rs4910656 | -1.0950942 | 0.18647234 | 4.2878E-09 |
| Smoking status: Never \|\| id:ukb-d-20116_0 | Major Depressive Disorder \|\| id:ieu-a-1188 | 173005 | rs4957528 | -1.0644746 | 0.1877613 | 1.4338E-08 |
| Smoking status: Never \|\| id:ukb-d-20116_0 | Major Depressive Disorder \|\| id:ieu-a-1188 | 173005 | rs523528 | -1.0743277 | 0.1885821 | 1.2203E-08 |
| Smoking status: Never \|\| id:ukb-d-20116_0 | Major Depressive Disorder \|\| id:ieu-a-1188 | 173005 | rs528301 | -1.1192281 | 0.18478888 | 1.3886E-09 |
| Smoking status: Never \|\| id:ukb-d-20116_0 | Major Depressive Disorder \|\| id:ieu-a-1188 | 173005 | rs55921136 | -1.0558857 | 0.18717625 | 1.6894E-08 |
| Smoking status: Never \|\| id:ukb-d-20116_0 | Major Depressive Disorder \|\| id:ieu-a-1188 | 173005 | rs6141314 | -1.0513725 | 0.1865017 | 1.727E-08 |
| Smoking status: Never \|\| id:ukb-d-20116_0 | Major Depressive Disorder \|\| id:ieu-a-1188 | 173005 | rs6265 | -1.1413717 | 0.17858998 | 1.6479E-10 |
| Smoking status: Never \|\| id:ukb-d-20116_0 | Major Depressive Disorder \|\| id:ieu-a-1188 | 173005 | rs6433897 | -1.0953347 | 0.18623418 | 4.0659E-09 |
| Smoking status: Never \|\| id:ukb-d-20116_0 | Major Depressive Disorder \|\| id:ieu-a-1188 | 173005 | rs6676022 | -1.0425949 | 0.18569331 | 1.9701E-08 |
| Smoking status: Never \|\| id:ukb-d-20116_0 | Major Depressive Disorder \|\| id:ieu-a-1188 | 173005 | rs6690680 | -1.0942795 | 0.186499 | 4.4246E-09 |
| Smoking status: Never \|\| id:ukb-d-20116_0 | Major Depressive Disorder \|\| id:ieu-a-1188 | 173005 | rs72505558 | -1.0829853 | 0.18573501 | 5.5159E-09 |
| Smoking status: Never \|\| id:ukb-d-20116_0 | Major Depressive Disorder \|\| id:ieu-a-1188 | 173005 | rs72678864 | -1.0687744 | 0.18809677 | 1.3309E-08 |
| Smoking status: Never \|\| id:ukb-d-20116_0 | Major Depressive Disorder \|\| id:ieu-a-1188 | 173005 | rs7333559 | -1.093639 | 0.18644962 | 4.4751E-09 |
| Smoking status: Never \|\| id:ukb-d-20116_0 | Major Depressive Disorder \|\| id:ieu-a-1188 | 173005 | rs748828 | -1.0655264 | 0.18834265 | 1.537E-08 |
| Smoking status: Never \|\| id:ukb-d-20116_0 | Major Depressive Disorder \|\| id:ieu-a-1188 | 173005 | rs7528604 | -1.0325759 | 0.18261177 | 1.5631E-08 |
| Smoking status: Never \|\| id:ukb-d-20116_0 | Major Depressive Disorder \|\| id:ieu-a-1188 | 173005 | rs7567570 | -1.0570652 | 0.18721264 | 1.6391E-08 |
| Smoking status: Never \|\| id:ukb-d-20116_0 | Major Depressive Disorder \|\| id:ieu-a-1188 | 173005 | rs763053 | -1.0909471 | 0.18681631 | 5.2302E-09 |
| Smoking status: Never \|\| id:ukb-d-20116_0 | Major Depressive Disorder \|\| id:ieu-a-1188 | 173005 | rs76608582 | -1.0805675 | 0.18789272 | 8.8727E-09 |
| Smoking status: Never \|\| id:ukb-d-20116_0 | Major Depressive Disorder \|\| id:ieu-a-1188 | 173005 | rs772921 | -1.0992792 | 0.18614639 | 3.5167E-09 |
| Smoking status: Never \|\| id:ukb-d-20116_0 | Major Depressive Disorder \|\| id:ieu-a-1188 | 173005 | rs7870475 | -1.0546994 | 0.18718331 | 1.7548E-08 |
| Smoking status: Never \|\| id:ukb-d-20116_0 | Major Depressive Disorder \|\| id:ieu-a-1188 | 173005 | rs7948789 | -1.0589083 | 0.19403601 | 4.8349E-08 |
| Smoking status: Never \|\| id:ukb-d-20116_0 | Major Depressive Disorder \|\| id:ieu-a-1188 | 173005 | rs883403 | -1.0594168 | 0.1869046 | 1.4429E-08 |
| Smoking status: Never \|\| id:ukb-d-20116_0 | Major Depressive Disorder \|\| id:ieu-a-1188 | 173005 | rs9375371 | -1.0862322 | 0.18753106 | 6.9438E-09 |
| Smoking status: Never \|\| id:ukb-d-20116_0 | Major Depressive Disorder \|\| id:ieu-a-1188 | 173005 | rs9381917 | -1.0481151 | 0.18595175 | 1.7355E-08 |
| Smoking status: Never \|\| id:ukb-d-20116_0 | Major Depressive Disorder \|\| id:ieu-a-1188 | 173005 | rs9487626 | -1.0722848 | 0.18982873 | 1.6167E-08 |
| Smoking status: Never \|\| id:ukb-d-20116_0 | Major Depressive Disorder \|\| id:ieu-a-1188 | 173005 | All | -1.0741474 | 0.18517807 | 6.6071E-09 |
